# Supplementary material for: Long-term safety and effectiveness of mRNA-1273 vaccine in adults: COVE trial open-label and booster phases
Source: Nat Commun. 2024 Aug 29;15:7469. doi: 10.1038/s41467-024-50376-z (PMC11362294; doi:10.1038/s41467-024-50376-z)
Supplement: Supplementary file 1 — Supplementary Information [file 41467_2024_50376_MOESM1_ESM.pdf]

Supplementary Appendix

Table of Contents

Table of Contents 1
COVE Trial Consortium (PubMed Listed, and Ordered Alphabetically by Institution Affiliation).. 4
List of COVE Trial Investigators (Consortium) and Study Teams (PubMed Listed, and Ordered Alphabetically by Institution Affiliation) 8
Supplementary Methods 20
Statistical analysis.. 20
Analysis of the Primary Effectiveness Endpoint for Parts B and C, and Long-term Analyses 20
Analysis approach for effectiveness of vaccine 21
Subgroup Analysis 22
Analysis of Other Effectiveness Endpoints 22
Immunogenicity Analysis... 23
Exploratory Endpoints... 25
Immunogenicity Assays 27
Figure S1. Parts B and C Analysis Set Populations 28
Figure S2. Part C Per-Protocol Immunogenicity Set... 30
Figure S3. Severe COVID-19 Events Based on Adjudication Committee Assessments Starting 14 Days After Booster Across Subgroups (Part C Per-Protocol Set).................. 31
Figure S4. Neutralizing Antibodies Against Ancestral SARS-CoV-2 (D614G) After mRNA-1273 Primary Series and Booster, Part C 32
Figure S5. Distribution of Pseudovirus Neutralizing Antibody Against SARS-CoV-2 (D614G), Part C PPIS and by Pre-Booster SARS-CoV-2 Status...................................... 33
Figure S6. Neutralizing Antibodies against Ancestral SARS-CoV-2 (D614G) After mRNA-1273 Primary Series and Booster, By Pre-Booster Infection Status and Age Group, PPIS.. 35
Figure S7. Distribution of Binding Antibody Against Ancestral SARS-CoV-2, Part C PPIS and by Pre-booster SARS-CoV-2 Status 36
Figure S8. Neutralizing Antibody Pre-Booster and Fold-rise (BD-1 to BD-29) Against Ancestral SARS-CoV-2 (D614G) Vs. Dosing Interval, mRNA-1273 Participants........... 38
Figure S9. Neutralizing Antibody Against Ancestral SARS-CoV-2 (D614G) Strain Pre-Booster Vs. Dosing Interval, PPIS-Negative mRNA-1273 and Placebo-mRNA-1273 Subset 39
Figure S10. Design of the COVE Exploratory Booster Analysis 40
Figure S11. Cumulative Proportion of Boosted Participants... 41
Figure S12. Exploratory Analysis of Booster Effectiveness Against Delta and Omicron (BA.1) Over Time 42
Figure S13. Exploratory Analysis of Booster Effectiveness Against Omicron (BA.1) Over Time by Age Groups... 43
Figure S14. COVE Study Design and Modifications.. 44
Table S1. Baseline Demographics Prior to Primary Vaccination, Part C Safety Set....... 45

Table S2. Summary of Unsolicited Adverse Events after 1st Injection in the Primary Series Safety Set 47
Table S3. Unsolicited Adverse Events Reported by ≥1% of Participants in Any Treatment Group, Primary Series Safety Set 48
Table S4. Serious Unsolicited Treatment-related Adverse Events of Participants in Any Treatment Group, Primary Series Safety Set 49
Table S5. Summary of Unsolicited Adverse Events ≤28 Days After Booster, Part C Safety Set 50
Table S6. Treatment-related Unsolicited AEs ≤28 Days after Booster Reported by ≥1% of Participants in Any Treatment Group, Part C Safety Set 51
Table S7. Serious Treatment-related Unsolicited Adverse Events ≤28 Days after Booster, Part C Safety Set 52
Table S8. Summary of Unsolicited Adverse Events ≤28 Days After Booster by Pre-booster SARS-CoV-2 Infection Status, Part C Safety Set 53
Table S9. Summary of Unsolicited Adverse Events ≤28 Days After Booster by Sex Status, Part C Safety Set 54
Table S10. Summary of Unsolicited Adverse Events ≤28 Days After Booster by Race, Part C Safety Set 55
Table S11. Summary of Unsolicited Adverse Events ≤28 Days After Booster by Ethnicity, Part C Safety Set 56
Table S12. Summary of Unsolicited Adverse Events ≤28 Days After Booster by Age Groups, Part C Safety Set 57
Table S13. Summary of Unsolicited Adverse Events up to Study-end After Booster, Part C Safety Set 58
Table S14. Genotypic Analysis of Adjudicated Cases 59
Table S15. Adjudicated COVID-19 Incidence Starting 14 days After Injection 2 of mRNA-1273 Primary Series, by Calendar Quarters, Per-Protocol Primary Series Set . 60
Table S16. Incidences of Secondary Endpoints Starting 14 days After Injection 2 of mRNA-1273 Primary Series, Per-Protocol Primary Series Set 61
Table S17. Summary of Incidence Rates for Other Effectiveness Endpoints (Part C Per-Protocol Set) 62
Table S18. Analysis of Incidence Rates of Severe COVID-19 Based on Adjudication Committee Assessments Starting 14 Days After Booster, by Pre-booster SARS-CoV-2 Status (Part C Safety Set) 63
Table S19. Pseudovirus Neutralizing Antibody Against SARS-CoV-2 (D614G) from Pre-vaccination Baseline, Part C Per-Protocol Immunogenicity Subset by SARS-CoV-2 Status 64
Table S20. Pseudovirus Neutralizing Antibody Against SARS-CoV-2 (D614G) from Pre-booster Baseline, Part C Per-Protocol Immunogenicity Subset by SARS-CoV-2 Status 65
Table S21. Pseudovirus Neutralizing Antibody Against SARS-CoV-2 (D614G) from Pre-booster Baseline, Part C PPIS by SARS-CoV-2-status and Age 66
Table S22. Pseudovirus Neutralizing Antibody Against Ancestral SARS-CoV-2 (D614G) from Pre-booster Baseline, Part C PPIS by SARS-CoV-2-status and Sex 67
Table S23. Spike Binding Antibody Response Against Ancestral SARS-CoV-2 (D614G) from Pre-vaccination Baseline, Part C PPIS by SARS-CoV-2 Status 68

Table S24. Spike Binding Antibody Response Against Ancestral SARS-CoV-2 (D614G) from Pre-booster Baseline Part C Per-Protocol Immunogenicity Subset 69
Table S25. Characteristics of Participants Included in the Exploratory Analysis of Time Intervals 70
Table S26. Baseline Demographics and Characteristics of Participants in Exploratory Effectiveness Analysis by Booster Timing 71
Table S27. Hazard Ratios and 95% Confidence Intervals for Delta and Omicron Cases by Boost Period 72
Table S28. Analysis Sets Definitions 73
Table S29. Analysis Periods for Analyses of Part B, Part C, and Primary Series 74
Table S30. Participants in Analysis Sets 76
Table S31. Criteria for mRNA-1273 Booster (Part C) as Compared With mRNA-1273 Primary Series (Part A) Superiority/Non-Inferiority Analysis 77
Supplement References 78
COVE Protocol 79
COVE Statistical Analysis Plan 272

COVE Trial Consortium (PubMed Listed, and Ordered Alphabetically by Institution Affiliation)

Affiliation/Funding*	Study Group	Location
AB Clinical Trials	Atoya Adams, MD, MBA, Eric Miller	Las Vegas, NV
Accel Research Sites	Bruce G. Rankin DO, John Hill MD, Steven Shinn MD, Marshall Nash MD	DeLand, FL
Advanced Clinical Research	Sinikka L. Green MD, Colleen Jacobsen, Jayasree Krishnankutty, Sikhongi Phungwayo	Cedar Park, TX
Alliance for Multispecialty Research	Richard M. Glover, II MD, Drs. Stacy Slechta, Troy Holdeman, Robyn Hartvickson, Amber Grant	Newton, KS
Alliance for Multispecialty Research	Terry L. Poling MD, Terry D. Klein MD, Thomas C. Klein MD, Tracy R. Klein MD	Wichita, KS
Alliance for Multispecialty Research	William B. Smith MD, Richard L. Gibson MD, Jennifer Winbigler MD, Elizabeth Parker PA	Knoxville, TN
Baptist Health Center for Clinical Research	Priyantha N. Wijewardane, MD, Eric Bravo MD, Jeffrey Thessing MD, Michelle Maxwell APRN, Amanda Horn APRN	Little Rock, AR
Baylor College of Medicine, NIAID 1UM1AI148575-01S2	Hana M. El Sahly MD, Jennifer Whitaker MD, Catherine Mary Healy MD, Christine Akamine MD	Houston, TX
Benchmark Research	Laurence Chu, MD, R. Michelle Chouteau, MD	Austin, TX
Benchmark Research	Michael J. Cotugno MD, George H. Bauer, Jr. MD	Metairie, LA
Benchmark Research	Greg Hachigian MD, Masaru Oshita MD, Michael Cancilla NP, Deborah Murray NP, Kristen Kiersey NP	Sacramento, CA
Benchmark Research	William Seger MD, Mohammed Antwi, Allison Green, Anthony Kim	Fort Worth, TX
Brigham and Women's Hospital, NIAID UM1AI069412, NCATS UL1RR025758	Lindsey R Baden MD, Michael Desjardins MD, Jennifer A Johnson MD, Michael Seaman, PhD, Amy Sherman MD, Stephen R Walsh MD	Boston, MA
Carolina Institute for Clinical Research	Judith Borger DO, Ryan Starr DO, Scott Syndergaard DO, Nafisa Saleem MD	Fayetteville, NC
Centex Studies	Joel Solis MD, Martha Carmen Medina PA-C, Westly Keating PA-C, Edgar Garcia PA-C, Cynthia Bueno PA-C	McAllen, TX
Clinical Research Atlanta	Nathan Segall MD, Nathan Segall, Jon Finley, Mildred Stull	Stockbridge, GA
Clinical Trials of Texas	Douglas Scott Denham DO, Thomas Weiss MD, Ayoade Avworo DNP, Parke Hedges MD	San Antonio, TX
Coastal Carolina Research Center	Cynthia Becher Strout MD, Rica Santiago, Yvonne Davis, Patty Howenstine, Alison Bondell	Mount Pleasant, SC
Cornell Clinical Trials Unit – Weill Cornell Uptown & Weill Cornell Chelsea, NIAID UM1AI068619, NCAT UL1TR002384	Kristin Marks MS MD, Grant Ellsworth, MS, MD, Tina Wang, MD, Timothy Wilkin, MD, MPH, Mary Vogler, MD, Carrie Johnston, MD, MS	New York, NY
COVID-19 Prevention Network (CoVPN, NIAID-NIH)	Michele P Andrasik, Jessica G Andriesen, Gail Broder, Lawrence Corey, Niles Eaton, Kathleen M Neuzil, Huub G Gelderblom, James G Kublin, Rachael McClennen, Nelson Michael, Merlin Robb, Carrie Sopher	Seattle, WA
DM Clinical Research	Vicki E. Miller MD MPH, Monica Murray MSN APRN FNP-C, Fredric Santiago MD, Blanca Gomez FNP-C, Shiela Varghese MSN, APRN, FNP-C, Insiya Valika PA-C, Amy Starr FNP-C	Tomball, TX
Emory University – Ponce de Leon Clinical Research Site, NIAID 3UM1AI068614-14S1	Colleen Kelley MD MPH, Valeria D Cantos MD, Sheetal Kandiah MD MPH, Carlos del Rio MD	Atlanta, GA
Emory University – Hope Clinic, NIAID 1UM1AI148576-01	Nadine Rouphael MD, Paulina Rebolledo, Srilatha Edupuganti, Daniel Sans Graciaa	Decatur, GA
Emory University School of Medicine, NIAID 1UM1AI148576-01	Evan J Anderson MD, Andres Camacho-Gonzalez MD, Satoshi Kamidani MD, Christiana A Rostad MD, Meghan Teherani MD	Atlanta, GA
George Washington University, NIAID UM1AI068619	David Joseph Diemert MD, Elissa Malkin, Marc Siegel, Afsoon Roberts, Gary Simon	Washington, DC
Hackensack University Medical Center	Bindu Balani MD, Carolene Stephenson, Steven Sperber, Cristina Cicogna	Hackensack, NJ

Affiliation/Funding*	Study Group	Location
Henry Ford Health System	Marcus J. Zervos MD, Paul Kilgore MD, MPH, Mayur Ramesh MD, Erica Herc MD, Kate Zenlea MPH	Detroit, MI
Hope Research Institute	Abram Burgher MD, Ann Marie Milliken	Phoenix, AZ
Hope Research Institute	Joseph D. Davis MD, Brendan Levy, Sandra Kelman	Chandler, AZ
Hope Research Institute	Matthew W. Doust MD, Denise Sample, Sandra Erickson	Phoenix, AZ
J. Lewis Research	Shane Glade Christensen MD, Christopher Matich, James Longe, John Witbeck	Salt Lake City, UT
J. Lewis Research	James Todd Peterson MD, Alexander Clark, Gerald Kelty, Issac Pena-Renteria	Salt Lake City, UT
Jacksonville Center for Clinical Research	Michael J. Koren MD, Darlene Bartilucci MD, Jeffery Jacqmein MD, Alpa Patel MD, Carolyn Tran MD	Jacksonville, FL
Javara	Christina Kennelly MD, Robert Brownlee, Jacob Coleman, Hala Webster	Charlotte, NC
Johnson County Clin-Trials	Carlos A. Fierro MD, Natalia Leistner, Amy Thompson, Celia Gonzalez	Lenexa, KS
Kaiser Permanente Washington Health Research Institute, NIAID 1UM1AI148373-01	Lisa A Jackson MD MPH, Janice Suyehira MD	Seattle, WA
Laguna Clinical Research Associates	Milton Haber MD, Maria M. Regalado MD, Veronica Procasky RN JD, Alisha Lutat	Laredo, TX
Lynn Health Science Institute	Carl P. Griffin MD, Raymond Cornelison, William Schnitz, Shanda Gower	Oklahoma City, OK
Lynn Institute of the Rockies	Ripley R. Hollister MD, Jeremy Brown DO, Melody Ronk PA-C	Colorado Springs, CO
M3 Wake Research	Wayne Lee Harper MD, Lisa Cohen DO, Lynn Eckert PA-C, Matthew Hong MD	Raleigh, NC
MediSync Clinical Research Hattiesburg Clinic	Rambod Rouhbakhsh MD, MBA, Elizabeth Danford MD, John Johnson MD, Richard Calderone MD	Petal, MS
Meridian Clinical Research	Shishir Kumar Khetan MD, Oyebisi Olanrewaju AC-CRNP, Nan Zhai NP-C, Kimberly Nieves AC-CRNP, Allison O'Brien AC-CRNP	Rockville, MD
Meridian Clinical Research	Paul Simon Bradley MD, Amanda Lilienthal MSN NP-C, Jim Callis PA-C	Savannah, GA
Meridian Clinical Research	Adam Benson Brosz MD, Andrea Clement PA, Whitney West APRN, Luke Friesen PA, Paul Cramer APRN	Grand Island, NE
Meridian Clinical Research	Frank Steven Eder MD, Ryan Little FNP, Victoria Engler FNP, John Tarbox FNP, Heather Rattenbury-Shaw DO	Binghamton, NY
Meridian Clinical Research	David Jon Ensz MD, Tavane Harrison, Allie Oplinger	Dakota Dunes, SD
Meridian Clinical Research	Brandon James Essink MD, Jay Meyer MD, Frederick Raiser, III MD, Kimberly Mueller APRN, Roni Gray PA	Omaha, NE
Meridian Clinical Research	Keith William Vrbicky MD, Charles Harper MD, Chelsie Nutsch MD, Wendell Lewis III MD, Cathy Laflan MD	Norfolk, NE
Meridian Clinical Research	Jordan L. Whatley MD, Nicole Harrell MD, Amie Shannon MD, Crystal Rowell APRN, FNP-C, Christopher Dedon APRN, FNP-C	Baton Rouge, LA
NIH	Mamodikoe Makhene MD MPH	Bethesda, MD
New Horizons Clinical Research	Gregory Mark Gottschlich MD, Kate Harden PA-C, Melissa Gottschlich PA-C, Mary Smith MSN, FNP-C, Richard Powell MD	Cincinnati, OH
Optimal Research	Murray A. Kimmel DO, Simmy Pinto MD	Melbourne, FL
Optimal Research	Timothy P. Vachris MD, Mark Hutchens MD, Stephen Daniels DO, Margaret Wells MD	Austin, TX
Optimal Research	Mimi Van Der Leden MD, PhD, Peta Gay Jackson Booth MD	Rockville, MD
Palm Beach Research Center	Mira Baron MD, Pamela Kane DO, Shannen Seversen PA-C, Mara Kryvicky PA-C, Julia Lord PA-C	West Palm Beach, FL
Paradigm Clinical Research Center	Jamshid Saleh MD, Matthew Miles, Rafael Lupercio	Redding, CA
Quality of Life Medical & Research Centers	John W. McGettigan Jr. MD, Walter Patton MD, Riemke Brakema MD, Karin Choquette MSN, ABNP-C, Jonlyn McGettigan MSN, RN	Tucson, AZ
Rancho Paseo Medical Group	Judith L. Kirstein MD, Marcia Bernard NP	Banning, CA
Rapid Medical Research	Mary Beth Manning MD, Joan Rothenberg MD, Toby Briskin MD, Denise Roadman PAC, Sharita Tedder-Edwards FNP	Cleveland, OH
Research Centers of America	Howard I. Schwartz MD, Surisday Mederos, Barbara Corral, Jennifer Schwartz, Nelia Sanchez-Crespo	Hollywood, FL

Affiliation/Funding*	Study Group	Location
Rutgers New Jersey Medical School, NIAID UM1AI068619	Shobha Swaminathan MD, Amesika Nyaku MD MS, Tilly Varughese MD, Michelle DallaPiazza MD	Newark, NJ
Saint Louis University, NIAID 1UM1AI148685-01	Sharon E Frey MD, Irene Graham MD, Getahun Abate MD PhD MSc, Daniel Hoft MD PhD	St. Louis, MO
St. Vincent's Health System	Leland N. Allen III MD, Leslie Anne Edwards MSN, CRNP, William Simpson Davis Jr., MS PA-C, Jessica Maria Mena, PA	Birmingham, AL
Suncoast Research Group	Mark E. Kutner MD, Jorge Caso MD, CPI, Maria Hernandez Moran APRN, Marianela Carvajal APRN, Janet Mendez APRN	Miami, FL
Sundance Clinical Research	Larkin T. Wadsworth III MD, Horacio Marafioti, Lyly Dang, Jennifer Berry, Lauren Clement	St. Louis, MO
Synexus Clinical Research	Michael Ryan Adams MD, Leslie Iverson PA	Murray, UT
Synexus Clinical Research	Joseph Lee Newberg MD, Laura Pearlman MS, MD, MBA	Chicago, IL
Synexus Clinical Research	Paul Joseph Nugent DO, Leonard Singer	Cincinnati, OH
Synexus Clinical Research	Michele Diane Reynolds MD, Jennifer Bashour MD, Robert Schmidt MD	Dallas, TX
Synexus Clinical Research	Neil Parmanand Sheth MD, Kenneth Steil DO	Glendale, AZ
Synexus Clinical Research	Ramy Joseph Toma MD, William Kirby MD, Pink Folmar MD, Samantha Williams NP	Birmingham, AL
Synexus Clinical Research	Judith White MD, Robert Meyer MD, Sejal Patel MD, Prity Patel APRN	Orlando, FL
Tekton Research	Paul Pickrell MD, Stefanie Mott FNP-C, Carol Ann Linebarger MD, Hussain Malbari MD, David Pampe MD	Austin, TX
Texas Center for Drug Development	Veronica G. Fragoso MD, Lisa Holloway MD, Cecilia McKeown-Bragas MD, Teresa Becker MD, Vicki Miller MD	Houston, TX
Trial Management Associates	Barton G. Williams MD, William H. Jones MD	Wilmington, NC
VA Greater Los Angeles Healthcare System	Michael Lewis MD, Elham Ghadishah, Joseph Yusin, Mai Pham	Los Angeles, CA
University of California Los Angeles, NIAID UM1AI068619	Jesse L Clark MD, Steven Shoptaw PhD, Michele Vertucci PA, NP, Will Hernandez NP	Los Angeles, CA
University of California San Diego, NIAID UM1AI068636	Stephen A. Spector MD, Amaran Moodley MD, Jill Blumenthal MD, Lisa Stangl NP, Karen Deutsch NP	La Jolla, CA
University of Chicago	Kathleen M. Mullane DO PharmD, David Pitrak MD, Cheryl Nuss FNP, Judy Pi PharmD	Chicago, IL
University of Cincinnati, NIAID UN1AI068619	Carl Fichtenbaum MD, Margaret Powers-Fletcher PhD, Michelle Saemann RN, Sharon Kohrs RN	Cincinnati, OH
University of Colorado Denver, Anschutz Medical Campus, NIAID UM1AI068636	Thomas B. Campbell MD, Andrew Lauria, Jose Castillo Mancilla, Hillary Dunlevy	Aurora, CO
University of Illinois at Chicago – Project WISH, NIAID UM1AI068619	Richard M Novak MD, Andrea Wendrow, Scott Borgetti, Ben Ladner	Chicago, IL
University of Maryland School of Medicine, NIAID 1UM1AI148689-01	Karen L Kotloff MD, Matthew Laurens, Milagritos Tapia, Lisa Chrisley, Cheryl Young	Baltimore, MD
University of Miami, NIAID 3UM1AI068614-14S1	Susanne Doblecki-Lewis MD, Maria Luisa Alcaide, Jose Gonzales-Zamora, Stephen Morris	Miami, FL
University of North Carolina at Chapel Hill, NIAID UM1AI068619	Cynthia Gay MD MPH, David Wohl MD, Joseph Eron, Jr. MD	Chapel Hill, NC
University of Pennsylvania, NIAID 3UM1AI068614-14S1	Ian Frank MD, Debora Dunbar, David Metzger, Florence Momplaisir	Philadelphia, PA
University of Pittsburgh Medical Center, NIAID 1UM1AI148452-01	Judith Martin MD, Alejandro Hoberman MD, Timothy Shope MD MPH, Gysella Muniz MD	Pittsburgh, PA
University of Texas Medical Branch, NIAID 1UM1AI148575-01	Richard Rupp MD, Amber Stanford PA-C, Megan Berman MD, Laura Porterfield MD	Galveston, TX
VA Greater Los Angeles Healthcare System	Michael Lewis MD, Elham Ghadishah, Joseph Yusin, Mai Pham	Los Angeles, CA

Affiliation/Funding*	Study Group	Location
Vanderbilt University Medical Center, NIAID 1UM1AI148452-01	Clarence Buddy Creech II MD, Shannon Walker MD, Stephanie Rolsma MD PhD, Robert Samuels, Isaac Thomsen MD	Nashville, TN
Vanderbilt University Medical Center, NIAID 3UM1AI068614-14S1	Spyros Andrews Kalams MD, Greg Wilson MD	Nashville, TN
Velocity Clinical Research	Gregg H. Lucksinger MD, Kevin Parks MD, Ryan Israelsen MD, Jaleh Ostovar FNP-C, Kary Kelly FNP-C	Medford, OR
Velocity Clinical Research, San Diego	Jeffrey Scott Overcash MD, Hanh Chu, Kia Lee, Karla Zepeda	La Mesa, CA
VitaLink Research	Luis I. De La Cruz MD, Steve Clemons, Elizabeth Everette, Suzanna Studdard	Greenville, SC
VitaLink Research	Gowdhami Mohan MD, Stefanie Tyson, Alyssa-Kay Peay, Danyel Johnson	Anderson, SC
VitaLink Research-Spartanburg	Gregory J. Feldman MD, May-Yin Suen, Jacqueline Muenzner, Joseph Boscia, Farhan Siddiqui	Spartanburg, SC
Wake Forest University Health Sciences	John Sanders MD, PhD, James Peacock MD, Julio Nasim MD	Winston Salem, NC
WR-Clinical Research Center of Nevada	Michael L. Levin MD, Julie Hussey MSN APRN FNP-C, Marcy Kulic MD	Las Vegas, NV
WR-ClinSearch	Mark Montgomery McKenzie MD, Teresa Deese, Erica Osmundsen, Christy Sweet	Chattanooga, TN
WR-Global Medical Research	Valentine Mbepson Ebuh MD, MA, MSc, Elwaleed Elnagar MD, Georgette Ebuh DNP APRN FNP-C, Genevieve Iwuala FNP	Dallas, TX
WR-Medical Center for Clinical Research	Laurie J. Han-Conrad MD, Todd Simmons MD, Denis Tarakjian MD	San Diego, CA

*Funding of institutions by the National Institute of Allergy and Infectious Diseases (NIAID) and/or research support by the National Center for Advancing Translational Science (NCATS) as indicated. All other institutions were funded by Office of the Assistant Secretary for Preparedness and Response, Biomedical Advanced Research and Development Authority. The content of this publication is solely the responsibility of the authors and does not necessarily represent the official views of the funding sources.

List of COVE Trial Investigators (Consortium) and Study Teams (PubMed Listed, and Ordered Alphabetically by Institution Affiliation)

Principal Investigator	Study Team	Institution	Location
Atoya Adams, MD, MBA	Miriah Campbell, Eric Miller, Daisy Langarica, Alia Bober, Diana Giraldo	AB Clinical Trials	Las Vegas, NV
Michael Ryan Adams, MD	Leslie Iverson, Andryelle Toledo, Melinda Bullington, Alicia Hanten, Carolyn Taylor, Shannon Wright, Chase Carnahan, Rachel Law, Natalie Smith, Julie Taylor, Jared-Robert Blake, Stefanie Vasconez, Courtney Jensen	Synexus Clinical Research	Murray, UT
Leland N. Allen III, MD	Leslie Anne Edwards, William Simpson Davis, Jr., Ronald Meza, Jordan Stauffer, John Farringer, Faith Holmes, Rhonda Buzbee, Cristina Velez, Huse Lisa, Lisa Huse, Camelia Speegle, Gregory Prestage, Mary Perez, Jessica Space, Matthew Todd, Jessica McDowell, Marha Bunnell-Pollak, Jackie Ziegler, Jasmine Ali, Dumitru Sirbu, Kellie Williams, Logan Sawyer, Richelle Chambliss, Samantha Blackmon, Stephanie Brennan, Tiffany Gibbs, Alexandria Anderson, Caitlin Roll, Candace Robinson, Zachary McCoy, Jessica Bartlett, Kimberly Cornelison, Chris Bovell, Vincent Baglini, Christy Greenhalgh, Jessica Maria Mena, David House, Matt Honold, Esteban Zurita	St. Vincent's Health System	Birmingham, AL
Evan J. Anderson, MD	Kathleen Stephens, Francine Dyer, Maya Stagg, Aaliyah Carron, Austin Lu, Julia Barton, Sy Tran, Leisa Bower, Esther Park, Jianguo Xu, Rebecca Gonzalez, Vy Ngo, Mike Shepard, Lezly Roxxette Zepeda, Karen Sytsma, Sandra Rojas-Honan, Felicia Glover, Susan Rogers, Theda Gibson, Christina A. Rostad, Andres Camacho-Gonzalez, Teresa Ball, Satoshi Kamidani, Mehgan Farah Teherani, Vikash Patel, Etza Peters, Peggy Kettle, Lisa Macoy, Cindy Lubbers, Amber Samuel, Laila Hussaini, Kathryn Zaks, Caroline Ciric, Meg Taylor, Oliver Smith, Amy Muchinsky, Sydney Biccum, Laura Clegg, Dean Kleinhenz, Angelle Ijeoma, Hannah Huston	Emory University School of Medicine	Atlanta, GA
Lindsey Baden, MD	Xhoi Mitre, Jon Gothing, Bruce Bausk, Jessica Cauley, Natalie Izaguirre, Lewis Novack, Michael Seaman, Katherine Yanosick, Henry Rutherford, Junghyun Kim, Dominique Betterbed, Kathleen Garvey, Lauren Clore, Alexander Mills, Deepesh Duwadi, Alessandra Setaro, Kyl Bowman, Kevin McManus, Sidali Beriane, Fadi Ghantous, Christy Lavine, Jasper Ophel, Joseph Sapiente, Jessica Dorning, Tessa Speidel, Lauren Garneau, Robert Dannemiller, Kirquenique Rolle, Mulika Chhorn, Bailey McCarthy, Hana Flaxman, Milenko Tanasijevic, Cameron Nutt, Javier Barria, Andre Avila-Paz, Buteau Malhaika, Tong Alexandra, Tenaizus Woods, Bethany Evans, Hannah Jin, LaKeisha Gandy, Stephanie St. Pierre, Carolyn Darcy, Michael Corrado, James Maguire, Adetoun Okenla, Tamara Roldon Sevilla, David Kubiak, Cassandre Titus, Movita Harrigan, Maria Alvarado, Rose Theodat, Amy Sherman, Laura Platt, Kirsten Goodman, Laura Nicholson, Wilfredo Matias, Emily Koleske, Ruth Rodriguez, Nicole Taikeff, Jun Bai Park Chang, Julia Klopfer, Phoebe Cunningham, Elizabeth Sampson, Karen Magsipoc, Maureen Macgowan, Lauren Donahue, Haley Schram, Noah Abasciano, Megan Powell, Janet Morgan, Yazed Alsowaida, Olivia Riccardi, Neha Limaye, Virginia Loudermilk, Austin Kim, Kevin Zinchuk, Caitlin Grant, Charles Kelly, David Mellace, Jamie Myers, Erika Gribb, Jose Licona, Monica Feeley, Stephen R Walsh, Jennifer A Johnson, Ann Woolley, Alexis Liakos, Jane Kleinjan, Jon Gothing, Nicolas Issa, Michael Desjardins, Raphael Dolin, Alka Patel, Opeyemi Talabi, Christin Price, Paulette Chandler, Elizabeth W Karlson, Allison P Moriarty	Brigham and Women's Hospital	Boston, MA
Bindu Balani, MD	Smith Kerowyn, Sergio Garcia, Charo Valdez, Shelly Chin, Caitlin DiBello, Silvia Lara, Chika Ekwegharirí, Abena Roberts, Abimbola Coker, Marie-Therese Estanbouli, Greg Eskinazi, Michael Tortoriello, Jay Elkareh, Meral Karakoc, Olga Spathis, Patrice Hassoun, Carolene Stephenson, Steven Sperber, Kaur Harveen, Cristina Cicogna, Ciaran Mannion	Hackensack University Medical Center	Hackensack, NJ
Mira Baron, MD	Pamela Kane, Maria Bermudez, Shannen Seversen, Mara Kryvicky, Julia Lord, Terri Barr, Daisy Acevedo, Elena Acosta, Delta Anderson, Alexandra Arango, Anne Bauer, Joshua Egbehor, Tim Flanary, Audrey Haber, Carol Henao, Patti Isaacson, Peter Jacob, Sakaiya Jackson, Karen Kodes, Ludovic La-Branche, Kimarie Lee-Russell, Carol Liso, Cristina Liso, Stephanie Morse, Michelle Navarrette, Christy Norcross, Nora Norcross, Annette Pitts, Mary Sergalis, David Scott, Tytiana Spearman, Danielle Theodore, Brian Thomas, Jennifer Torres	Palm Beach Research Center	West Palm Beach, FL

Principal Investigator	Study Team	Institution	Location
Judith Borger, DO	Jennifer Angell, Nicole Austin, Deanna Benz, Lucian Cappoli, Nicole Davis, Lynn Eckert, Kathryn Hostetter, Stephanie Keating, Jeanette Mangual-Coughlin, Avia McClain-Stocker, Ifeanyi Momodu, Cheryl Norris, Brennan Opanasenko, Stacey Saldua, Nafisa Saleem, Amy Sheets, Ryan Starr, Scott Syndergaard, Jennifer Thomas, Michelle Wallace, Jeffery Pemberton, Mitchell Arildsen, Dan Tomita	Carolina Institute for Clinical Research	Fayetteville, NC
Paul Simon Bradley, MD	Taja Adams, Stephanie Ailey, Kira Bell, Shanice Bennett, Vincent Bernades, Jim Callis, Bounphone Chanthavong. Taryn Collett, Anne Crouch, Shannon Davis, Morgan Deal, Mimi Duncan, Brandon Essink, Laura Falcone, Debra Gabrielson, Brooke Halpern, Anyfa Hanna, Cassie Heisey, Dawn Kalloniatis, Andrew Kimball, Jeanette Lee, Amanda Lilienthal, Ginny McNew, Crystal Neely, Kay Lynn Olmsted, Nicole Osborn, Chevon Roberts, Pechoka Sanders, Cynthia Seedorf, Kathryn Stoddard, Jonathan Whelan, Stella Yoon	Meridian Clinical Research	Savannah, GA
Adam Benson Brosz, MD	Rhonda Richter, Debra Gabrielson, Kayla Flege, Ashley Bell, Karen Jo Johnson, Paul Cramer, Jessica Stanton, Andrea Clement, Whitney West, Laura Falcone, Amanda Friesz, Kathy Osborne, Summer Tophoj, Kimber Breeden, Susan Newman, Douglas Herbek, Lindsey Mettenbrink, Luke Friesen, Alison Pierce	Meridian Clinical Research	Grand Island, NE
Abram Burgher, MD	Stephanie Catanzaro, Shauna Harrell, Magen Hess, Nate Alderson, Bettie D'Nise Corcoran, Norma Frederick, Adrian Alejo, Brian DeCraene, Karen Wakefield, Scarlett Hammett, Susan DeCraene, Ann Marie Milliken, Neil Pearson, Donald Terral Harper	Hope Research Institute	Phoenix, AZ
Thomas B. Campbell, MD	Andrew Lauria, Jenelynn Kimble, Steven Johnson, Matin Krsak, Andrew Monte, Patrisha Adkins, Michelle Barron, Suzanne Fiorillo, Amy Harrison, Anderson Victoria, Nga Le, Sara Berech, Jose Castillo-Mancilla, Kristine Erlandson, Laurel Ware, Josie Marshall, Stephen Bartlett, Hillary Dunlevy	University of Colorado Denver, Anschutz Medical Campus	Aurora, CO
Shane Glade Christensen, MD	Christopher Mickelson, Jessica Shaw, Emily Raming, Amy Nelson, Gabrielle Lewis, Jenessa Folsom, Mikaela Jones, Dylan Owen, Rachel Pugmire, Jennifer Bradley, Annjanette Kemp, Krista Marti, Allyson Christensen, Madison Ellis, Holly Anderson, Emily Bloomquist, Ross Brunetti, Thomas Conner. Jr., Gina Cox, Diana Grazulis, Wesley Lewis, James Longe, Christopher Matich, Bryan Nelson, Sarah Scott, John Witbeck, Stephen Wood	J. Lewis Research	Salt Lake City, UT
Laurence Chu, MD	Jennifer Bacchi, Maria Barrientes, Lamar Box, Christian Casas, R. Michelle Chouteau, Katherine Davis, Tambra Dora, Cindy Duran, Pamela Fidler, Ruth Fitch, Brooke Harris, Isaiah Knight, Jennifer Leyva, Michelle Listz, Jennifer Montes, Javier Perez, Jessica Ruff, Dean Skiles, Sean Turnbow, Francesca Vigil, Breana Wade, Kelly Weber	Benchmark Research	Austin, TX
Jesse L. Clark, MD	Sandy MacNicoll, Somaieh Talebi, Timothy Hall, Steven Shoptaw, Emery Chang, Michael Li, David Goodman, Paul Adamson, Oladunni Adeyiga, Inez Bentancourt, Susan Reed, Christopher Blades, Jasmin Tavares, Demetria Villanueva, Simone Riley, Jonathan Veloz, Schuyler Thomas, Will Hernandez, Jennifer Baughman, Mitchell Stern, Michele Vertucci	University of California, Los Angeles	Los Angeles, CA
Michael J. Cotugno, MD	Kyra Lawson, Kim Harper, Edwin Adamson, George H. Bauer Jr., Julie Bilich, Brenda Lawson, Brandon Illickal, Lois Eaglin, Heather Salisbury, Jeff Segner	Benchmark Research	Metairie, LA
Clarence Buddy Creech II, MD	Shanda Phillips, Naomi Kown, Katherine Sokolow, Wendy Winn, Katherine Wright, Shannon Walker, Stephanie Rolsma, Anna Gallion, April Hanlotxomphou, Deborah Myers, Robert Adkisson, Natalia Jimenez, Cindy Trimmer, Roberta Winfrey, Matthew Donio, John Oleis, Donna Torr, Shelly McGehee, Robert Samuels, Sandra Yoder, Eric Brady, Isaac Thomsen, Madeleine Guy, Emma Alexander, Lana Howard, Krisha Alexander, Shane Moore, Tacora Wright, Tara Evans, Ursula Powell, Jenna Caserta, Valerie Mitchell, Meryk Moore, Melissa Lehman, Diane Anders, Constance Dotye, Crystal Rice, Lamar Bowman, Sherri Hails, Monique Bennett, Nicki Soper, Leigh Howard	Vanderbilt University Medical Center	Nashville, TN
Joseph D. Davis, MD	Sandra Kelman, Sandra Braden, Sabrina Bolland, Mia Munoz, Jose Barocio, Brendan Levy, Dhwani Shah, Neil Pearson, Stephanie Catanzaro, Nathan Alderson, Susan DeCraene, Maureen Godfrey, Skyla Clark	Hope Research Institute	Chandler, AZ
Luis I. De La Cruz, MD	Amy Ford, Taylor Wilson, Cindy Smith, Austin Lambert, Erin Zeiler, Kaelyn Rowland, Marlee Smith, Suzanna Studdard, Zandra Hamilton, Meredith Benfield, Sara Poff, David Godwin, Elizabeth Everette, Steven Clemons, Kayla Peay, Stephanie Gilreath	VitaLink Research	Greenville, SC
Douglas Scott Denham, DO	Thomas Weiss, Parke Hedges, Ayoade Avworo, Kay Scroggins, Leisel Koerber, Antonio Gutierrez, Nathan Cortez, Andrea Gomez, Darlington Akahara, Michelle Smith, Kristy Trevino, Beatriz Herrera, Shaiane	Clinical Trials of Texas	San Antonio, TX

Principal Investigator	Study Team	Institution	Location
	Dickerson, Kerry de Jesus, Matthew Korte, Cynthia Ramos, Reanna Martinez, Erica Leal, Shakera Flores, Paul Esparza, Brian Hemming, Melinda Axton, D'Andre White, Terri Perez, Carolina Coronado, Rebecca Many, Clayton Stone, Kimberly Evans, Anshumaan Maharaj, Stephen Brick, Steffanie Barrera, Staci Poettgen, Dawn Killian, Gerardo Pena, Karol Perez, Victoria Hernandez, Kevin Martinez, Amy Griffith, Nolan Payton, Quincey Hogue, Jamie Padilla, Emily Mendez, Lily Hays, Maristelle Co, Nicholas Trinidad, Ismael Rodriguez, Amy Lewis, Cindi Nellis, Lele Simmons, Marissa Johnson		
David Joseph Diemert, MD	Linda Witkin, Aimee Desrosiers, DeEnna Wedding, Bertran Walton, LaKeisha Queen, Ryan Mouton, Caroline Thoreson, Manya Magnus, Jennifer Wald, Erika Faust, Nicholas Heredia, Robbie Kattappuram, Hira Qadir, Chelsea Ware, Hannah Yellin, Kegan Dasher, Daniel Mullen, Jeanne Jordan, Taylor Ladson, Madison Lintner, Kaitlyn Macnair, Bitana Saintilma, Kelly Thomas, Samantha Walker, Neha Rampally, Madhu Balachandran, Elissa Malkin, David Parenti, Hana Akselrod, Marc Siegel, Gary Simon, Afsoon Roberts, Aileen Chang	George Washington University	Washington, DC
Susanne Doblecki-Lewis, MD	Maria Luisa Alcaide, Jose Gonzalez-Zamora, Stephen Morris, Yimy Puerto, Annie Salvarrey, Claudia Balgas, Claudia Santos, Katherine King, Brahian Steven Erazo, Mayra Fernandez, Leopoldo Cordova-Garcia, Elisa Corzo-Sanchez, Edgar Fernandez, Loreta Padron, Stefani Ann Butts, Kenia Moreno, Juan Casuso, Maria de Pilar Valanzasca, Thomas Tanner, Marilyn Fernandez, Mary Aloise, Inza Patton, Vivian Pastrana, Sendy Puerto, Irma Barreto Ojeda, Junlin Long, Barbara Huang, Gilianne Narcisse, Vanessa Perez	University of Miami	Miami, FL
Matthew W. Doust, MD	Denise Sample, Sandra Erickson, Nate Alderson, Adrian Alejo, Stephanie Catanzaro, Susan DeCraene, Cassie Enricco, Sandra Erickson, Alex Guereque, Shauna Harrell, Shana Harshell, Stephanie Junker, Stephanie Laufenberg, Madison Mikulak, Makayla Morra, Nicole Olson, Neil Pearson, Jasmin Redden, Monique Romo, Denise Sample, Dhwani Shah, Sahara Vega, Emma Kar	Hope Research Institute	Phoenix, AZ
Valentine Mbepson Ebuh, MD	Elwaleed Elnagar, Georgette Ebuh, Genevieve Iwuala, Catina Adams, Marissa Cervenka, Ezgar Del Real, Shraddha Dubal, Elwaleed Elnagar, Jenifer Fiatte, Kathy Harrell, Genevieve Iwuala, Vicki Martinez, Robert Miranda, Brennan Opanasenko, Destiny Robinson, Liz Ruiz, Amy Sheets, Shoniece Wallace	WR-Global Medical Research	Dallas, TX
Frank Steven Eder, MD	Ryan Little, Victoria Engler, John Tarbox, Heather Rattenbury-Shaw, Deborah Hubish, Jessie Taylor, Debra Gabrielson, Jessica Fellows, Jennifer Molstead, Kathe Olmstead, Ashley Conover, Tammy Kohn, Chelsea Briar, Corrine Young, Collen McVannan, Kelli Quick, Shaylynne Hubanks, Kimber Breeden, Ann Marie Sampson, Traci Hull, Tarin Gordon, Susan Owen, Kate Macarak, Tonya Rackett, Jacob Blattstein, Partidge Jane Aton, Nicole Croft, Carolyn Grausgruber, Rebecca Miller, Ryan Little, Victoria Engler, John Tarbox, Heather Rattenbury-Shaw, Nathan Kimball, Courtney Heisey, Ginny McNew, Abigail Wine, Cindi VanKuren, Jared Frick, Tammy Dennis, Andrew Kimball	Meridian Clinical Research	Binghamton, NY
Hana M. El Sahly, MD	Jennifer A. Whitaker, C. Mary Healy, Christine Akamine, Wendy A Keitel, Robert L Atmar, Annette Nagel, Sandra Francisco, Thea Marie Cordero, Janet Brown, Jennifer Christensen, Caroline Doughty-Skierski, Connie Rangel, Carrie Kibler, Coni Cheesman, Lisreina Toro, Chanei Henry, Chianti Wade Bowers, Pedro Piedra, Kathy Bosworth, Kayla Burrell, Jesus Banay, Tykel Eddy, Trent Davis, Shetel Anassi, Yvette Rugeley, Olga Rybina-Willis	Baylor College of Medicine	Houston, TX
David Jon Ensz, MD	Pamela Allen, Taylor Bergh, Kimber Breeden, Avery Dunn, Brandon Essink, Debra Gabrielson, Rylea Gulick, Tavane Harrison, Courtney Heisey, Andrew Kimball, Shelby Klaschen, Jessica Knight, Makayla Langston, Meagan Miller, Allie Oplinger, Heather Persinger, Alison Pierce, Kathryn Stoddard, Kayla Sturgeon, Jamie Thompson, Melissa Wiseman	Meridian Clinical Research	Dakota Dunes, SD
Brandon James Essink, MD	Jay Meyer, Frederick Raiser, Kimberly Mueller, Roni Gray, Riley Brockman, Tabitha Campbell, Carrie Essink, Laura Falcone, Roni Gray, Linda Layton, Jay Meyer, Kimberly Mueller, Tiffany Nemecek, Frederick "Fritz" Raiser, III, Jessica Satorie, Chelsea Steinmetz, Nicole Osborn, Cassie Heisey, Maria Nguyen	Meridian Clinical Research	Omaha, NE
Gregory J. Feldman, MD	May-Yin Suen, Brittany Cooksey, Madison Fowler, Sarah Chynoweth, Gary Clemons, Laura Jolly, Charlie Jordan, Heather Allison, Steve Clemons, Amber Brittany Belcher, Allison Kelly, Marsha Gossett, Wendy Taylor,	VitaLink Research-Spartanburg	Spartanburg, SC

Principal Investigator	Study Team	Institution	Location
	Amy Witt, Kendal Nelson, Jeffrey Witt, Jacqueline Muenzner, Elizabeth Everette, Supinder Channa, Allison Ayers, Joseph Boscia, Farhan Siddiqui		
Carl J. Fichtenbaum, MD	Maggie Powers-Fletcher, Michelle Saemann, Sharon Kohrs, Kimberly Mullins, Lindsay Davis, Moises Huaman, Angela Snyder, Kristin Weghorn, Brenda Miller, Elizabeth Costea, Lisa Schira, Romana Saeed, Helen Shelton, Kathleen Ballman, Laura Browning-Cho, Sherry Donaworth, Chris Goddard, Jeanine Goodin, Elizabeth Niederegger, Lisa Hachey, Tamara Maus, Pam Fletcher, Makayla Bishop, Victoria Straughn, Shaina Horner, Carrie Christofield, Dana Burns, Jason Mayes, Kelly Windholtz, Lisa Proffitt, Faizan Qureshi, Michelle O'Neil, Arustamyan Lisa, Sarah Trentman, Eva Whitehead, Jennifer Baer, Linda Hinds, Jaasiel Chapman, D'Vaughn House, Gary Frazier, Judy Houston, Lisa Altenau, Mary Burns, Dorice Smith, Justin Ragle, Eric Mueller, Cynthia Nypaver, Jaime Robertson, Anissa Moussa, Geronimo Feria Garzon, Sierra Bennett, Marlena Petrie	University of Cincinnati	Cincinnati, OH
Carlos A. Fierro, MD	Mazen Zari, Celia Gonzalez, Natalia Leistner, Mary Easley, Mary Provost, Krista Estrada, Ann Geier, Amy Thompson, Heather Barker, Karol Moore, Kelly Moen, Monica Atwood, Amber Wolf, Brandi Dickerson, Manyvohn Rinehart, Dina Hammine, Angela Eichler, Casey Johnson, Nathan Arthur	Johnson County Clin-Trials	Lenexa, KS
Veronica G. Fragoso, MD	Lisa Holloway, Cecilia McKeown-Bragas, Teresa Becker, Vicki Miller, Leena Mir, Elton Oliveira, Moez Talpur, Enya Rentas-Sherman, Gabriela Maria Becerra, Dewayne Hicks, Robert Krbashyan, Shakira Barr, Ashraf Jafri, Herman Ortiz, Zohair Harianawala, Chandra Tobin, Norma Gonzalez, Saji Perinjelil, Khorshid Amirkhosravi, Tracy Kowalski, Biman Goswami, Waheeda Sureshbabu, Amy Anderson, Berenice Ferrero, Simeen Khan, Chen-Ho Yang, Nazanin Zarinkamar, Scott Ward, Crystal Reese, Miyosha Lewis, Olga Konshina, Lorrian Yates, Joel Cano, Quiana Wilson, Kara Sikes, Diana Chehab, Joanna Quezan, Maryam Rabbani, Sadaf Batla, Abbyssinia Moges. Diego Carrington, Matthew Joseph, Laura Grissanty, Dean Jang, Dustin McFadden, Misbah Baloch, Elisa Moralez, Abdeali Dalal, Frances Saubon, William Fernandez, Jenny Toress, Blessing Felix, Zain Rizvi	Texas Center for Drug Development, Inc.	Houston, TX
Ian Frank, MD	Annet Davis, Eileen Donaghy, Nicole Sundo, Juan Ramirez, Laura Schankel, Dana Brown, Katharine Bar, Dana Brown, Christopher Chianese, Gillain Constantino, Dovie Watson, Kathleen Degnan, Helen Koenig, William Short, Petra Alexander, Eileen Mergliano, Jie Ho, Michele Wisniewski, Debora Dunbar, Liani Santini-Lopez, Rosemarie Kappes, Angela Cabassa, Tammy Chen, Berry SotoVega, Deborah Kim, Devon Cliett, Kate Kearns, Jillian Baron, Vivian Leung, Florence Momplaisir, Sarah Wood, Tameka Matthews, David Metzger, Richard Tustin	University of Pennsylvania	Philadelphia, PA
Sharon E. Frey, MD	Irene Graham, Getahun Abate, Daniel Hoft, Heather Douds, Cassandra Zehenny, Joan Siegner, Helay Hassas, Kim Cooper, Shirley Dettlebach, Sabrina DiPiazza, Carol Duane, Linda Eggemeyer-Sharpe, Lauren Foreman, Jerry Hutter, Ryan Kerr, Kate Liefer, Tracy Montauk, Karla Mosby, Janice Tennant, Nicole Purcell, Kiana Wilder, Kathleen Chirco, Sharon Irby-Moore, Kathleen Koehler, Melissa Loyet, Thomas Pacatte, Susan Stewart, Azra Blazevic, Tamara Blevins,<br>Chase Colbert, Christopher Eickhoff, Lainey Mejia Jauregui, Keith Meyer, Krystal Meza, Amanda Nethington, Huan Ning, Brittany Williams, Mei Xia, Yinyi Yu, Stanley Doublin, Mary Pat Eastman, Eric Eggemeyer, Mikayla Frye, Michelle Harris, Aleshia McCoy, Donna Duncan, Gwendolyn Tatum, Nicole Purcell, Kiana Wilder, Tammy Grant, Claudia Castillo Paredes, Rong Hou, Jin Wang, Qian Wang, Sarah George	Saint Louis University	St. Louis, MO
Cynthia Gay, MD	David Wohl, Joseph Eron, Jr., Andrew Thorne, Michelle Floris-Moore, Christopher Hurt, David Wohl, Chidinma Okafor, Janette Goins, Ulrike Adam, Ekundayo Nylander-Thompson, Anna Furlong, XinHong Ao, Kathy Guerrero, Melinda Hart, Kathleen Loeven, Rachael Mossey, Esther Speight, Rachel White, Chloe Twomey, Kristen Gray, Miriam Chicurel-Bayard, Susanne Henderson, Patti Vasquez, April Welch, Camille O'Reilly, Maureen Furlong, Noshima Darden-Tabb, Elizabeth DuBose, Marie Oriol, Dynesha Perry, Maria Stetson, Maria Bullis, Shelby Turner, Ebony Harrington, Michael Herce, Suzanne Blevins, Alexander Bradley, Susan Pedersen, Becky Straub, Sandra Barnhart, Felicia Barriga Munante, Nazneen Howerton, Tevnan Keller, Mandy Tipton, Abigail Riddick, Kristi Kirkland, Maggie Harman, Tania Hossain, Centhla Washington, Erin Hoffman, Carolina Pastrana Medina, William Johnson, Samantha Earnhardt, Amy James Loftis, Catherine Kronk, Yaa Ofori-Marfoh, Julie A Nelson, Nicole	University of North Carolina at Chapel Hill	Chapel Hill, NC

Principal Investigator	Study Team	Institution	Location
	Maponga, Lina Rosengren-Hovee, William Zhao, Jennifer Thompson, Sarah Law, Holly Milner, Jonathan Oakes, Rachel Cook, Erin Cardot, Oesa Vinesett, Victoria Rucinski, Joy Wannamaker, Tanailly Giralt Smith, Eliza DuBose, Chidinma Okafor		
Richard M. Glover, II, MD	Stacy Slechta, Troy Holdeman, Robyn Hartvickson, Amber Grant, Jennifer Bennett, Lindsey Brewer, Janelle Brown, Kelsey Burden, Melissa Burton, Brianna Burton, Jordan Danby, Sheri Duncan, Amber Grant, Robyn Hartvickson, Lisa Hemmelgarn, Sherry Henning, Jeri King, Riley King, Colton King, April Kitterman, Shannen Lassiter, Cayla Lawless, Janna Martinez, Ragene Moore, Marissa Mueller, Aaron Nguyen, Justin Phillips, Jordan Reheis, Rebecca Ring, Katherine Saengerhausen, Shannon Thomas, Dylan Thomas, Cindy Thome, Denae Villines, Amber Wenzel, Eilleen Wilbert, Avi Woods, Caressa Presley, Brianna Newport, Olivia Allen, Miranda Santiago, Cheryl Sauerwein, Jill Longstaff, Sadie Allen, Candace Heckart	Alliance for Multispecialty Research	Newton, KS
Gregory Mark Gottschlich, MD	Melissa Gottschlich, Steven Anderson, Gregory Mark Gottschlich II, Mary Woeste, Kate Harden, Cindy Young, Michael Pordy, Audrius Ruksenas, Lacy Baird, Kim Krogman, Lori Stanton, Melissa Fuson, Mason Urban, Christine Watson, Richard Powell, Mary Smith, Jacob Sekinger, Diamond Russell, Nicole Lim, Mylene Asmar-Rios, Yusef Museitif, Craig Mitchell, Tarik Whitham, Zachary Rutledge, Troy Porter, Andrea Newlands, Jami Ramsey, Mary Frances Curry, Nishay Holloman, Crystal Barket, Michelle Spear, Shelley Mahan, Taeleigha Greene, Zachary Eardley, Gen Moussa, Mary Ann Gottschlich	New Horizons Clinical Research	Cincinnati, OH
Sinikka L. Green, MD	Julie Hamilton, Alex Fuller, Jeanette Dickhaus, Colleen Jacobson, Triny Cooper, Michelle Jackson, Taylor Evans, Tabitha Judd, Kathryn Alexander, Megan Rosallo, Sikhongi Phungwayo, Robin Dotson, Dana Finley, Michael Vasquez, Cyndi Foster, Gregg Lucksinger, Sarah Smiley, Jayasree Krishnankutty, Ray Coon, Grishma Dhimmer, Melanie Wilkerson, Tatum Shawver, Marcedes Coffman, Devin Teal, Laura Crenshaw	Advanced Clinical Research	Cedar Park, TX
Carl P. Griffin, MD	William Schnitz, Andrea Romero, Kim Hamilton, Raymond Cornelison, Angela Genovese, Shelly Brunson, April Green, Lacey Dietz, Kim Calloway, Chris Hyatt, Destiny Heinzig-Cartwright, Chalimar Rojo, Sharee Wright, Kathi Shaw, Michael Pojezny, Avery Keller, Krystal Hightower, Dalia Tovar, Shanda Gower	Lynn Health Science Institute	Oklahoma City, OK
Milton Haber, MD	Maria Candelario, Martha Bunnell-Pollak, Lauren Wade, Jackie Ziegler, Deena Ramirez, Perla Avalos, Maria Drada, Jasmine Ali, Jessica McDowell, Kehinde Busari, Patricia Church, Ronald Meza, Marco Vela, Esteban Zurita, Chris Connolly, Ruben Del Bosque, Alisha Lutat, Chelsea Fleming, Brett Potthoff, Anita Suri, Cynthia Priester, Brenda Hernandez, Veronica Procasky, Eva Cerreta, Matt Honold, Melinda Rodriguez, Maria Regalado, Jordan Stauffer	Laguna Clinical Research Associates	Laredo, TX
Greg Hachigian, MD	Michael Cancilla, Ricardo Castellanos, Angela Cuellar, Yaman Darmarathne, Shaila Faulker, Yana Gordeyeva, Michelle Hisey, Ashley Jungsten, Kristin Kiersey, Pawandeep Nagra, Nav Nagra-Kooner, Jazmin Nauta, Masaru Oshita, Kenneth Quick, Julie Raygoza, Amanny Sadek, Melisa Tinder, Jhoana Torres, Deborah Murray, Kristen Kiersey	Benchmark Research	Sacramento, CA
Laurie J. Han-Conrad, MD	Brandon Baldwin, Lucian Cappoli, Tenisha Garcia, Ella Grach, Brenda Grande, Nicolle Mendez, Natalie Moy, Matthew Musikanth, Karen Mylerberg, Brennan Opanasenko, Mark Pulera, Patti Sanchez-Emery, Mireles Sarah, Todd Simmons, Denis Tarakjian	WR-Medical Center for Clinical Research	San Diego, CA
Wayne Lee Harper, MD	Toni Bland, Lori Bridges, Lucian Cappoli, Lisa Cohen, Leah Corts, Annie Craft, James Earnhardt, Lynn Eckert, Aubrey Farray, Laura Hoer, Matthew Hong, Chris Hoyle, Jenee Jiggetts, Brian Joseph, Bradley Killebrew, Kendra Lisec, Lucie Mangala, David Musante, Adnan Nasir, Amanda Olsen, Brennan Opanasenko, Marci Parks, Marion Peoples, Katherine Schuch, Judith Shand, Sabine Ucik, Douglas Wadeson, Barbara Wheeler	M3 Wake Research	Raleigh, NC
Ripley R. Hollister, MD	Jeremy Brown, Brandy Ball, Jeremy Brown, Valerie Dyster, Dalia Jeronimo, Shelby Pickle, Michael Pojezny, Melody Ronk, Kathi Shaw, Bobbi Shofner, Jami Wagner, Meghan York, Jill York	Lynn Institute of the Rockies	Colorado Springs, CO
Lisa A. Jackson, MD, MPH	Marilyn Nguyen, Maya Dunstan, Barbara Carste, Sarah Friend, Diana McFeters, Lynn Gross, Mohamed Ajenah, Jana Ffitch, Audra Mccoy, David Skatula, Susan Lasicka, Kimberly Brinker, Karen Sherwin, Melissa Scheer, Paula Lins, Roger Calvert, Roxanne Erolin, Stella Lee, Vi Tran, Stephanie Pimienta, Bruce Douglas, Lee Barr, Colin Fields, Erika Kiniry, Joe Choe, Janice Suyehira, Joyce Benoit, Michael Witte, Rebecca Lau	Kaiser Permanente Washington Health Research Institute	Seattle, WA

Principal Investigator	Study Team	Institution	Location
Spyros Andrews Kalams, MD	Greg Wilson, Kyle Rybczyk, Katie Crumbo, Carly Griffin, Latoya Hannah, Amy Kerrigan, Valerie Mitchell, Jenna Caserta, Mary Downey, Nicole Swindle, Shonda Sumner, Amber Massey, Trudy Sullivan, Rita Smith, Cindy Nochowicz, Eric Olson, Christian Warren, Josh Simmons, Dana King, Gwendolyn Rees, Matt Donio, Jesse Case, Keith Richardson, Jarissa Greenard	Vanderbilt University Medical Center	Nashville, TN
Colleen Kelley, MD, MPH	Valeria D. Cantos, Sheetal Kandiah, Carlos del Rio, Christina Bacher, Hannah Huston, Juliet Brown, Divya Bhamidipati, Nithin Gopalsamy, Brittany Lynn Speigel, Elizabeth (Betsy) Hall, Brandon Spratt, Kiran Dhillon, Caitlin Moran, Michael Chung, Felecia Wright, Marcia Peters, Rondell Jaggers, Vanessa Soliman, Ron Gaston, Christopher Foster, Sarah Wiatrek, Bezuayehu Mandefro, Pamela Weizel, Pamela Lankford-Turner, Anandi Sheth, John Gharbin, Catherine Abrams, Philip Powers, Paulina Rebolledo, Christin Root, Tiraje Lester, Sha Yi, Damien Swearing, Fred Ede, Isaac Perez, Kelly Likos, Meen Dhir, Aastha KC, Gabriela Georgial, Tucker Colvin, Nabeel Yar Khan, Valarie Hunter, D’Jamel Young, Felecia Atkinson	Emory University Emory University – Ponce de Leon Clinical Research Site	Atlanta, GA
Christina Kennelly, MD	Jacob Coleman, Brittany Bundeff, Melissa C. Hennessey, Kenneth Owen, Caroline Wilds, Jennifer Womack, Susan Martello, Chiedza Hooker, Robert Brownlee, Melissa James, Deborah Wesley-Farrington, Lori Whiteheart, Hala Webster, David Framm, Cortney Fretz, Gwyn Gibson, Susan Donahue, Kelly Woodell, Linda McCarty, Jim Vesely, Scott Chatterton, Andrew Ottesen, Enrico Belgrave, Krishna Shah, James Chester Alexander, Brittain Callahan	Javara	Charlotte, NC
Shishir Kumar Khetan, MD	Taja Adams, Tanya Alexander, Tanya Alexaner, Sydney Barmoy, Jake Bart, Kira Bell, Ira Berger, Jemario Blackwell, Priscilla Buahin, Bounphone Chanthavong, Juliana DeVito, Azure Erskine, Brandon Essink, Laura Falcone, Debra Gabrielson, Beau Garland, Barb Geiger, Tiana Oliver, Courtney Heisey, Sucharita Katikala, Andrew Kimball, Heather Lang, Jeanette Lee, Asefa Mekonnen, Devan Myers, Kimberly Nieves, Allison O’Brien, Oyebisi Olanrewaju, Nicole Osborn, Adunola Oshiyoye, Rahul Patel, Alan Pollack, April Poole, Collin Smith, Kathryn Stoddard, Chao Wang, Sean Whelan, Jonathan Whelan, Graciela Zapata, Nan Zhai	Meridian Clinical Research	Rockville, MD
Murray A. Kimmel, DO	Alexa Diec, Ann Riley, Bette Denmat, Bram Swarr, Christina Raidl, Dania Billman, Denise Dixon, Donald Dawson, Elaine Crudo, James Crowley, Katrina Carlson, Kaylie Worzick, Laura Worth, Lisbeth Gordon, Marion Oliver, Robert Holt, Simmy Pinto, Taylor Atkinson, Traci Mitchell, Lana Ghomrawi, Norma Rokoff	Optimal Research	Melbourne, FL
Judith L. Kirstein, MD	Jared Bradshaw, Krista Forster, Jeanette Dickhaus, Marcia Bernard, Erica Sanchez, Nikki Abels, Cynthia Kunakom, Vanessa Vandergoot, Jessica Fisher, Carol Remigio, Jourdan Manfred, Frederick Lloyd, Tiffany Williams, Clarisse Baudelaire, Lovette Cherelle, Nolan Mackey, Alan Valenzuela, Theodore Wyman, Alyssa Taber, Karen Myers, Craig Koch	Rancho Paseo Medical Group	Banning, CA
Michael J. Koren, MD	Shannon Trull, Amanda Elwood, Mary Strickland, Ivy Gulliermo, Chistopher Ganzhorn, Sonia Gerardo, Taylor Johnson, Victoria Kaposchansky, Cassie Lawler, Laura Little, Amanda Pratt, Sheldon Warren, Andrea West, Emery Noles, Nathanial Grant, Jillian Agnew, Lori Alexander, Brenda Anderson, Deirdre Arrington, Sara Benner, Lisa Carl, Allison Crain, Nafisa Ishaku, Robert Nix, Sharon Smith, Amber Devries, Sandy Salceiro, Opara Chukwudi, Mikaela Karney-Trull, Ramil Castillo, David Graham, Gail Lowe, Alexander Hill, Carolyn Tran, Jeffry Jacqmein, Darlene Bartilucci, Alpa Patel, Janet Garvey, Mitchell Rothstein, Kenneth Aung-Din, Margaret Gannaway, Arman Mughal, Sandra Fuit, Jolenne Wolfer, Erin Schelhorn, Jacob Wolfer, Madison Martinez, Melissa Parks, Patricia Neal	Jacksonville Center for Clinical Research	Jacksonville, FL
Karen L. Kotloff, MD	Matthew Laurens, Milagritos Tapia, Lisa Chrisley, Cheryl Young, Barbara Albert, Robin Barnes, Shernel Barrett, Andrea Berry, Melissa Billington, Shannon Bittner, Colleen Boyce, Faith Pa’Ahana Brown, James Campbell, Regina Carpenter, Jamonie Carter, Ginny Cummings, Brenda Dorsey, Jorge Flores, DeAnna Friedman-Klabanoff, Shirley George, Nancy Greenberg, Hassan Haji, Elizabeth Hammershaimb, Susan Holian, Leslie Howe, Myounghee Lee, Alyson Kwon, Kirsten Lyke, Alma Valle Maldonado, Jennifer Marron, Kaitlin Mason, Monica McArthur, Rosa McBryde, Sherry McCammon, Sandra Molina, Kathleen Neuzil, Daniele Nitkowski , Justin Ortiz, Rekha Rapaka, Mardi Reymann, Toni Robinson, Wanda Somrajit, Mark Travassos	University of Maryland, School of Medicine	Baltimore, MD

Principal Investigator	Study Team	Institution	Location
Mark E. Kutner, MD	Amanda Colina, Isett Caro, Frances Beltran, Jessie Downs, Jonathan Fernandez, Mariete Renden, Mirnaya Mujica-Alabaci, Susel Figueredo, Yanelis Dominguez, Jaime Blandon, Bryan Ruiz, Leidy Montoya, Edgardo Rodriguez, Jessie Downs, Jason Rothscheld, Janett Acle, Yaime Martinez, Soraya Ricardo, Maria Hernandez Moran, Eloisa Guerra, Heidie Perez, Claudia Rodriguez, Victoria Moreno, Vanessa Hechavarria, Saray Carvajal, Daniel Lopez, Carlos Iviricu, Neiner Enriquez, Paola Garcia, Chris Hoyle, Marianela Carvajal, Janet Mendez, Edisleidy Mesa, Marco Ramirez, Dalila Del Valle, Jennifer Ortega, Yeni Hernandez, Jhobana Vargas, Carmen Amador, Juan Delgado, Maury Santos, Meredith Arguelles, Leyanis Coello, Vanessa Ansorena, Jorge Caso, Stacy Machado, Raydel Valdes, Giann Lightbourn, Dayami Dovales, Alain Chang	Suncoast Research Group	Miami, FL
Mimi Van Der Leden, MD, PhD	Chrishea Harvey, Tricia Oyeyemi, Aicha Moutanni, Stephanie Melton, Peta-Gay Jackson Booth, Jennifer Yoon, Gloria Kim, Atanas Filev, Francis Uwandi, Meyling Lopez, Janice Spreitzer, Courtney Gennes, Xiangfei Cheng, Matthew Van Sickle, Nick Bart, Brianne Okunji, Frank Maloba	Optimal Research	Rockville, MD
Michael L. Levin, MD	Brennan Opanasenko, Yajaira Ramos, Shonda Lester, Rebecca Boucher, Shawn Harrell, Shon Boucher, Patti Sanchez, Nina Scharbach, Alex Sanchez, Shyane Raniello, Wendy Guerra, Krystal Tyner, Kimberly Temple, Ruby Ortiz, Daniel Terreault, Amy Kill, Jade Odynski, Adolfo DeLeon, Debbie Carter, Eduardo Rodriguez, Julia Gass, Sara Esparza, Sierra Dansbee, Tammy Harrison, Marcy Kulic, Lucian Cappoli, Mora Kim, Matthew Fenner, Heather Jimenez, Shraddha Dubal, Julie Hussey	WR-Clinical Research Center of Nevada	Las Vegas, NV
Michael Lewis, MD	Nancy Mohler, Mai Pham, Elham Ghadishah, Ruoxiang Wang, Justine Velandria, Benjamin Dreskin, Joseph Yusin, Lauren Vigil, Sara Wong, Suchi Tiwari, Joseph Pisegna, Sunita Dergalust, Wayman Lee, Hadia Al Abdullah, Stephen Sirmay, Sara Broukhim	VA Greater Los Angeles Healthcare System	Los Angeles, CA
Gregg H. Lucksinger, MD	Jaleh Ostovar, Craig Koch, Danuel Hamlin, Kelly Chase, Jeanette Dickhaus, Edward Kerwin, Frederick Forde, Allison Alvord, Dawn Stewart, Dan Hamlin, Kevin Parks, Ryan Israelsen, Kary Kelly, Tiffany Smith, Melissa Myers, Ryan Rackley, Audrey Kuehl, Savannah Peterson, Hannah Hall, Jay Weisbart, Alison Dodenhoff, Emily Kelly	Velocity Clinical Research	Medford, OR
Mary Beth Manning, MD	Carol Salango, Alec Ireland, Lisa Hoagland, Jeanette Dickhaus, Toby Briskin, Joan Rothenberg, Michael Gaston, Sharita Tedder-Edwards, Denise Roadman, Megan Sokolowski, Tina Shickluna, Katherine Bielanski, Samantha Hood, Talia Chandler, Brianna Arman, Melinda DeLong, Naqib Ahmad, Karly Tarase, Jade Svoboda, Lisle Merriman, Melisa Sebera, Emma Landskroner, Amy Maroun, Brooke Glivar, Jennifer Gaston, Sarah Dzigiel, Cassiandra Uminski, Karol Sabol, Devan Patel, Nick Zarbo, Briana Jackson, Brian Sharpe, Nicole, Baitt, Kaitlyn Duffy, Gabrielle Jacobs, Ann Czuprun, Tracee Cash, Diamond Ivey, Kaitlyn RubelI	Rapid Medical Research	Cleveland, OH
Kristen Marks, MD	Grant Ellsworth, Tina Wang, Timothy Wilkin, Mary Vogler, Carrie Johnston, Marshall Glesby, Roy Gulick, Ole Vielemeyer, Rebecca Fry, Todd Stroberg, Caitlin Rhoades, Noah Goss, Shaun Barcavage, Valery Hughes, Jonathan Berardi, Caroline Greene, Sarah Galloway, Caique Mello, Ashley Machado, Mia Crowley, Monique Williams, Katherine Fee, Elizabeth DeJesus, Andrew Yu, Minkyung Lee, Susan Herder, Mary Ann Zweibel, Patrice Weller, Antonio Rivera-Lopez, Edward Kenny, Hetal May, Natella Fridman, Parul Shah, Ruby Lee, Venus Fernandez, Victoria Lesina, Celine Arar, Byron Bullough, Kinge-Ann Marcelin, Brian Mangano, Jessenia Fuentes, Jiamin Li, Genessi Rodriguez, Catherine Jerry, Nadi Islam, Liqun Cai, Wayne Burns, Akinbayo Caulcrick, Andrika Thomas, Barbara Batog, Guoan He, Sara Yoder, Tamara Crowder, Gianna Resso, Sophia Alvarez, Tahera Begum, Elizabeth Connolly, Roxanne Rosario, Paul Kim, Steven Wang, Vasilika Koci	Cornell Clinical Trials Units – Weill Cornell Chelsea and Uptown	New York, NY
Judith Martin, MD	Alejandro Hoberman, Timothy Shope, Gysella Muniz, Sonika Bhatnagar, Kumaravel Rajakumar, Anne-Marie Rick, Peri Unligil, Jennifer Nagg, Melissa Andrasko, Mary Ann Sieber, Jennifer Opal, Lalicia Roman, Spenser Kinsey, Michelle Burke, Matthew Lee, Dominic Kramer, Linette Milkovich, Emily Dougherty, Emily Carney, Shannon Mance, Nader Shaikh, Diana Kearney, Jamie Fries, Lisa Vavro, Shayla Goller	UPMC University Center	Pittsburgh, PA
John W. McGettigan, Jr., MD	Walter Patton, Jennifer Schnider, Riemeka Brakema, Heeten Desai, Mikell Brett Karsten, Patricia Jalomo, Cindy Finch Benoy, Karin Choquette, Jonlyn McGettigan, Yvonne De Los Reyes, Melissa Cozzens, Amanda Hermosillo, Cindy Montgomery, Susan Tarwid, Annette Elzy, Tianna Young, Saysamone Banks, Cristina Fernandez, Damaris Atondo, Zoe Sesma, Norma Barrientos, Maggie Tono, Kisha Adams, JoAnn Wilkins, Arianna Bermudez,	Quality of Life Medical & Research Centers	Tucson, AZ

Principal Investigator	Study Team	Institution	Location
	Carol Sayer, Julie McDowell, Angelina Navarro, Mercedes Sullivan, Crystal Mata, Sheldon Gingrich, Aaliyah Sestiaga, Gia Longo		
David C. Montefiori, PhD	David Beaumont, Rebecca Beerman, Kendall Bradley, Jiayu Chen, Xiaoju Daniell, Thomas Denny, Elizabeth Domin, Amanda Eaton, Kelsey Engel, Wenhong Feng, Juanfei Gao, Hongmei Gao, Kelli Greene, Sarah Hiles, Marianne Jessup-Cumming, Leihua Liu, Kristy Long, Kellen Lund, Kaia Lyons, Charlene McDanal, Marcella Sarzotti-Kelsoe, Francesca Suman, Haili Tang, Jin Tong, Olivia Widman	Duke University Medical Center	Durham, NC
Mark Montgomery McKenzie, MD	Tiffany Jewell, Zackery Harmon, Michael Elizabeth, Christy Sweet, Teresa Deese, Catherine Schon, Misti Earwood, Lou Cappoli, Brennan Opanasenko, Lisa Guider, Michelle Forgey, Justian Jarrett, Rachel Scott, Elizabeth Michael, Erica Osmundsen, Andrew Wood, Shelly Brooks, Gisela Heintz, Lilian Nukuna	WR-ClinSearch	Chattanooga, TN
Vicki E. Miller, MD	Sajjad Naqvi, Soofia Masood, Fredric Santiago, Sonia Guerrero, Subhash Koneru, Nirja Shah, Andrea Torres, Ramani Gali, Talha Baig, Heather Leary, Afifah Ayub, Nayab Goher, Patti Tate, Reagen Reed, Muhammad Irfan, Amy Starr, Alefiyah Motiwala, Julia Kenny, Victoria Aguilar, Jessica Arguijo, Insiya Valika, Victoria Aguilar, Jagruti Patel, Anna Pena, Faryal Mahmood, Blanca Gomez, Nancy Torres, Kristyn Latil, Tarori Mark, Laura Djampou, Lindsey Kueng, Marianne Tadros, Mohammad Millwala, Monica Murray, Murtaza Marvi, Shivani Shah, Vanessa Gonzalez, Zohair Harianawala, Zainab Rizvi, Ambily Dileep, Jaquelyn Gonzales, Ragen Powell, Carolina Deandres, Syed Fahad Ali Kazmi, Sandra Natalia Perez, Shannon Amacker, Shiela Varghese	DM Clinical Research	Tomball, TX
Gowdhami Mohan, MD	Rodolfo Barrera, Emma Partin, Kelly White, Ashley Rochester, Charles Thompson, Stefanie Tyson, Ashten Sheriff, Alyssa-Kay Peay, Kayla Corn, Barbara A. Richardson, Kristin Miller, Steven Clemons, Cameron King, Emma Partin, Gary Clemons, Brianna Starr, Danyel Johnson, Taylor Davis, Niki Tyson	VitaLink Research	Anderson, SC
Kathleen M. Mullane, DO, PharmD	David L. Pitrak, Cheryl Nuss, Karen Cornelius, Randee Estes, Amy Luckett, Michelle Moore, Judi Pi, Stephen Schrantz, Jill Stetkevych	University of Chicago	Chicago, IL
Joseph Lee Newberg, MD	Mary Reyes, Nicole Leahy, Victoria Andriulis, Herbert Whinna, Patricia James, Lana Ghomrawi, Carole Kempfer, Miriam Arroyo, Maria Castro, Anna Maddox, Reuben Martinez, Jacquilyn McCormick-Burks, Laura Pearlman, Rosalinda Vazquez, Shaheera Suleiman, Neha Atal, Rosalind Vazquez	Synexus Clinical Research	Chicago, IL
Richard M. Novak, MD	Regina Harden, Maria Schwarber, Michael Pacini, Rebeca Gansari, Margie Villarreal, Stephanie Martin, Michelle Lee, Richard Morrissy, Taylor Ellis, Samuel Rene, Tara Cobbs, Claudia Preciado, Scott Borgetti, Maximo Brito, Olamide Jarrett, Mahesh Patel, Tracy Cable, Charity Ball, Maryann Holtcamp, Rodrigo Burgos, Sarah Michienzi, Emily Drwiega, Mikayla Johnson, Fischer Herald, Benjamin Ladner, Minseung Chu, Carolyn Dickens, Alfredo Mena Lora, Stockton Mayer, Andrea Wendrow, Habiba Sultana, Nanu Nunwar, David Chan, Marla Schwarber, Khandaker Anwar, Mahmood Ghassemi, Md Ruhul Amin, Doris Carroll, Rosa Valencia, Michelle Agnoli, Elena Llinas, Samuel Rene, Liam Morrissy, Adrian Raygoza, Addis Mekkonnen, Lisa Lindemann, Daniel Meslar, Karen Pacini, Corey Ringhisen, Amy Kennedy-Krage, Claudia Miller, Lorna Sanchez McCann, Gizelle Alvarez, Nia Moragne-Oneal, Nusirat Williams, Ian Feather, Nikki Griffith, Wardrick Nealon, Renyce Powell, Nila Safaeian, Monica Gingell, Diana Bahena, Gerald Beck, Brad Farrington, Rod Reyes, Monica Wilson, Juline Wondrasek, Kimberly Shapiro, Shannon Whitted, Victoria Roehl, Braulio Carrasco, Michael Chen, Olivia Murray, Yasiel Lacalle, Tessa Eckley, Anna Schluckebier, Kevin Cao, Elise DeBruyn	University of Illinois at Chicago – Project WISH	Chicago, IL
Paul Joseph Nugent, DO	Leonard Singer, Jennifer Jones, April Smith, Georgettea 15njec, Lana Ghomrawi, Christine Bennett, Norma Blevins, Linda Brotherton, Michele Byrd, Krista Doss, Victoria Holden, Christine Hull, Jean Montgomery, Nancy Cipollone, Savanah Torline, Brandon Brown, Meagan Thomas, Katie Ziska, Dana Sias, Hannah Wagner	Synexus Clinical Research	Cincinnati, OH
Jeffrey Scott Overcash, MD	Hanh Chu, Kia Lee, Karla Zepeda, John Rodriguez, Adam Prince, Yashveer Dubbula, Elizabeth Tomatsu Michael Voskanian, Crystle Rajania, Stephanie Ramirez, Claudia Camacho, Lauren Arnett, Kecia Darbeau, Ashley Smith, Kimberly Quillin, Cesar Ramirez, Daniel Robitaille, Erica Sanchez, Allie Davis, Michael Waters, Pat Kappen, Valerie Horne, Thao Vuong, Andrew Dennis, Nikki Abels, Dominique Panis, Richard McQuaid, Whitley	Velocity Clinical Research, San Diego	La Mesa, CA

Principal Investigator	Study Team	Institution	Location
	Harbison, Erika Trujillo, Andrea Garcia, Jose Jacob Esparza, Carlos Vera, Raquel Taitingfong, Cathy Meza, He Pu, Jackielynn Smith, Shandel Odom, Zahira Nieves, Ashliegh Lindsay, Ariana Nasatka, Jose Cazarez, Nora Martinez, Angela Hunt, Antonio Delgado, Linda Vega, Angela Anorve, Erica Martinelli, Melania Riordan, Sylvia Lindholm, Gina Ciezkowski, Grecia Perez, Jacob Pineda, Nathan Tyler, Ranya Salem, Amara Yilmaz, Jessica Gonzales, Zabrina Ruiz, Laura Castillo, Yajaira Contreras, Angelica Guzman, Makenna Orel, Jeffery Alvarez, Gordon Bovee, Roxana Ramirez, Joan Esquivel		
James Todd Peterson, MD	Christopher Mickelson, Madeline Maldonado, Alison Charlton, Ashley Bragg, Sean Hansen, Emily Wilcox, Colby Bostock, Megan Henry, Pam Iwasaki, Bradley Young, Katelyn Walker, Joy Nguyen, Lindsey Bevan, Megan Grimmett, Madeline Grote, Heather Littell, Natalie Bee, Alexander Clark, Shana Eborn, Susan Edwards, Dan Henry, Heather Jackson, Gerald Kelty, Issac Pena-Renteria, Jacqueline Rohrer, Jack Taylor, Brooke Barrick, Ty Henry, Anna Dansie, Kenadie Hamblin	J. Lewis Research	Salt Lake City, UT
Paul Pickrell, MD	Susan Bonner, Blaire Graham, Staci Taggart, Hussain Malbari, Tiffany Lemuz, Ethan Shotton, Andrew Bell, Megan Malek, David Pampe, Carol Ann Linebarger, Michelle Peterson, Brandi Chalman, John Luna, Elizabeth Santellanes, Christina Martinez, Lisa Johnson, Lisa Savage, Melissa Winn, Wendi McKenzie, Eileen Euperio, Stefanie Mott, Paul Menefee, Katie Caballero, Darrell O’Brien, Morgan Schulle, Kate Jurek, Olivia Hapanowicz	Tekton Research, Inc.	Austin, TX
Terry L. Poling, MD	Meenakshi (Kavya) Natesan, Patricia Contreras, Denise Hole, Avi Woods, Jill Hiebert, Melissa Burton, Olivia Eagleson, Laura Holz, Terri Ford, Cindy Thome, Terry D Klein, Gregory Greer, Diandra Henriques, Tracy R Klein, Thomas C Klein, Christa Shue, Gina Young, Brenna Sprout	Alliance for Multispecialty Research	Wichita, KS
Bruce G. Rankin, DO	Jennifer Dittman, Lora Parahovnik, Crystal Paccione, Melissa Hodges, Katina Marchione, Matt Maxwell, Any Dominy, Diana Toney, Andrea Marrafino, Laura Isbell, Leandro Fernandez, Claxton Copeland, Michelle Tutt, Adam VanDeusen, Kevin Feldman, Clark Mason, Tifany Huertas, Over Seijas, Jennifer Cline, Christian Beierschmitt, Ryan Hobbick, Jessica Gilliam, Jeanette de Leon, Iman Mencia, Daniel Layish, Vienna Bauer, Shatonia Fields, Albert Garcia, Carrie Rycort, Tasha Brocato, Marshall Nash, Samantha Watts, Amy Houck-Dominy, Angela Hammerle, Teresa Logsdon, Erika Wierzbicki, Taylor Martin, Ranie Hutchins, Fadhel Alyunis, Gail Lavine, Jeffery Hood, Robert Duran, Michelle Jones, Ginny McClanahan, Heather Jackson, Leandra Fernandez, Douglas Winter, Antonio Rivera, Amber Vasquez, Thais Truffa, Daniel Campbell, Grace Newcomb, Elizabeth Orlando, Steven Shinn, John Hill, Christina Isbell, Dhaneshwar Oomrow, Alicia Cevera	Accel Research Sites	DeLand, FL
Michele Diane Reynolds, MD	Jennifer Bashour, Robert Schmidt, Cynthia Mayeux, Uvoka Huffman, Lisa Nicholson, Jacklyn Newton, Lynn Yauch, Cathy Monroe, Kathleen Carty, Angelica Banks, Taylor Werner, Pamela Echols, Pauline Jackson, Chana Hines, Lorine Cook, Cristina Puig, Patrick Brooks, Jennifer Ruiz, Deanna Bowman, Ladina Garcia	Synexus Clinical Research	Dallas, TX
Rambod Rouhbakhsh, MD, MBA	John Johnston, Richard Calderone, Tasha Stevenson, Tameka Fortune, Brandi Pace, Adreanna Pou, Jerrica Sullivan, Yolanda Lewis, April Rouse, Tiffany Jefferson, Elizabeth Danford, Jeff Repper, Mason Boutwell, Alexycia Washington, Krista Hirth, Meagan Grabel	MediSync Clinical Research Hattiesburg Clinic	Petal, MS
Nadine Rouphael, MD	Renata Dennis, Tigisty Girmay, Michelle Wiles, Sharon Curate-Ingram, Lauren Hewitt, Alexis Ahonen, Mari Hart, Sarah Bechnak, Erin Carter, Lauren Nolan, Daniel Sans Graciaa, Geoffrey Kamau, Easton Beshears, Sy Tran, Mary Atha, Mary Bower, Ghina Alaaedine, Brandy Johnson, Jacob Usher, Eileen Osinski, Erin Scherer, C. Tae Stallworth, Stephanie Ramer, Rose Pope, Esther Park, Francine Dyer, Laura Clegg, Rebecca Gonzalez, Stacey Wheeler, Susan Rogers, Vy Ngo, Vanessa Soliman, Kristen Unterberger, Bernadine Panganiban, Christopher Huerta, Juton Winston, Ali Alvarez, Jianguo Xu, Colleen Kelley, Paulina Rebolledo, Nicholas Scanlon, Jessica Traenkner, Matthew Collins, Hollie Macenczak, Cassie Grimsely-Ackerley, Tiffany Lee, Amy Anderson, Michele Paine McCullough, Hannah Huston, Daniella Carter, Lisa Harewood, Srilatha Edupuganti, Varun Phadke, Mindee Adamson, Jeanne Allen, Debbie Bartenfeld, Lily Berz, Amy Cromwell, Sergio Cruz, Fred Ede, Monica Godfrey, Evan Gutter, Angelle Ijeoma, Sara Jo Johnson, Vinit Karmali, Dean Kleinhenz, Jennifer Kleinhenz, Alexandra Koumanelis, Maranda Leary, Tiraje Lester, Juliet Alise Morales, Shashi Nagar, Julia Paine, Dilshad Rafi Ahmed, Brittany Robinson, Amanda Rosner, Renee Silver, Trevor William Simon, Talib	Emory University – Hope Clinic	Decatur, GA

Principal Investigator	Study Team	Institution	Location
	Sirajud-Deen, Damien Swearing, Maliya Tolbert, Pamela Turner, Chia Uzuegbunam, Claire Wan, Dongli Wang, Erika Wimberly, Jean Winter, Joy Winters, Yong Xu, Sha Yi		
Richard Rupp, MD	Amber Stanford, Megan Berman, Laura Porterfield, Gerianne Casey, Hala Ghoson, Doreen Jones, Michael Willig, Cori Burkett, Robert Cox, Amy McMahan, Diane Barrett, Kristin Pollock	University of Texas Medical Branch	Galveston, TX
Jamshid Saleh, MD	Matthew Miles, Rafael Lupercio, Vicky Martin, Marla Clark, Matthew Pohlmeyer, Ruba Zanaid, Veronica Blevins, Tara Ulberg, Carlyee Chambers, Marisol Corrales, Emily Crews, Mohamed Yassin, Sarah Sandberg, Frank Chen, Mandy Swanson	Paradigm Clinical Research Center	Redding, CA
John W. Sanders, MD, MPH	Stacy Harpe-Hall, Jesse Hopkins, Ann Schweppe, Jaymous Fayssoux, Kathryn Bender, James Peacock, Katharine Pearsall, Brandy Snyder, Deidre Knox, Megan Thorpe, Melissa Ellingson, Brittany Bundeff, Lisa Ashworth, Meredith Hiatt, Ritu Rathee, Stacy Woodliff, Brian Strittmatter, Amanda Wright, Daisy DeWeese-Gatt, Caryn Morse, John Williamson, Samantha Wheeler, Lori Whiteheart, Susan Donahue, James Lovette, Kaitlyn Van Leuvan, Kelly Ledbetter, Scott Chatterton, Julio Nasim, Amie Sidberry, Ashley Davis, Carter Noecker, Chie Hooker, Johanna Breenan, Sam Cable, Anna Bowman, Stephanie Boothe, Shea Overcash	Wake Forest University Health Sciences	Winston Salem, NC
Howard I. Schwartz, MD	Carlos Valladares, Jocelyn Morrera, Yulexis Amestoy, Tori Wallenburg, Thelma Beltran, Terry Piedra, Monica Garces, Alexandra Galvis, Wanda Delgado, Catherine Casas, Lesly Miguel Sosa, Vivian Rosales, Jose Fernando Henriquez, Mikael Yaniz, Beatriz Rivera, Peter Ventre, Gabriella Huyke, Maria Companioni, Jessie De Vega, Brianna Gamez, Stephanie Diaz, James Jean-Mary, Americo Padilla, Nikita Notise, Yorlina Luquetta, Monifa Wilson-Morris, Kenia Gutierrez, Roilan Garcia, Karla Pentzke, Leyda Valentin, Lazara Novas, Marilein Camacho, Jazmin Henfield, Laymis Alvarez, Myriam Rosado, Maxine Bryant, Maria Pinero, Laura Raucci, Francisco Ramirez, Angelic Gamez, Mailin Perez, Yasmin Baddour, Hary Leon Joseph, Yaquelin De la Cruz, Dunia Torres, Rosaidaliz Carreira, Chanella Garcia, Surisaday Mederos, Jose Muniz, Karenda Plotka, Sara Gomez, Maria Soto, Cathy Cruz, Nelia Sanchez-Crespo, Jennifer Schwartz, Barbara Corral, Matthew Muniz, Dayana Deltejo, Ana Castro, Reem Hassan	Research Centers of America	Hollywood, FL
Nathan Segall, MD	Michelle Sowell, Nancy Levine, Erynn McKinley, Hannah Smith, Karen Hickson, Elizabet West, Patrizia Greene, Jon Finley, Mildred Stull, Susan Jones, Jennifer LeBrun, Pamela Talbott, Kwannda Whatley, Jeffrey Jones, Michelle Binns, Donna Toepfer, Cynthia Steele, Grace Newville, Gillian Waite, Cynthia Pinckney, Karen Yangapatty, Kiara Tyner, Kimberly Cobb, Kourtney Richardson	Clinical Research Atlanta	Stockbridge, GA
William Seger, MD	Kimberly Pullen, Jean Seignon, Anthony Kim, Mohammed Antwi, Allison Green, Lizzy Seger, Elizabeth Boydston, Abdur Rafay Qadri, Deborah Devlin, Tasha Todd, Oluwatosin Akingbala, Alma Guel, Tisha Davis, Melody Dufrene, Samantha Loudermilk, Virginia Loudermilk, Crystal Starr, John Villegas, Ben Seger, Katherine Hollie	Benchmark Research	Fort Worth, TX
Neil Parmanand Sheth, MD	Kenneth Stell, David Beckett, Enitt Gonzalez, Donna McGunigal, Amanda Burns, Nancy Wood, Shelley Miceli, Christina Avila, Rebecca Baker, Laura Vigliotti, Sarah Kading, Samer Salama	Synexus Clinical Research	Glendale, AZ
William B. Smith, MD	Richard L Gibson, Jennifer Winbigler, Elizabeth Parker, Madison Watts, Suzann Cloninger, Talya Thomas	Alliance for Multispecialty Research	Knoxville, TN
Joel Solis, MD	Martha Carmen Medina, Xavier Morales, Hank Heller, Blake Torrence, Joanna Gurrola-Mahoney, Cynthia Bueno, Heather Holloway, Irving Salinas, Joel Perez, Paola Garcia, Erica Canales, Blanca Urbina, Brancisilio Gutierrez, Carolina Cantu, Chelsea Vargas, Cindy Vasquez, Cody McIntire, Gabriela Gutierrez, Hugo Sosa, Irvin Munoz, Jessica Estrada, Jonna Lopez, Kaegan Knox, Mirella Melendez, Natalia Valle, Natalie Echavarria, Nicole Litton, Amber Victor, Nancy Torrence, Madhu Shreya, Mathew Maran, Asfak Alam, Westly Keating, Tara Green, Devora Torrence, Gerardo Sedas, Shruti Konda, Prem Jangam, Mario Echavarria, Alejandro Silva, Anne McNulty, Daniel Contreras, Daniel Gomez, Edgar Garcia, Elizabeth Weber, Luis Lopez, Samuel Ramirez, Kayla Lopez, Pedro Penalo, Angel Salinas, Jaime Solis, Shannon Moyer, Aryana Ibarra, Guadalupe Gurrola, Jenna Anastasiades, Uchechi Ehiemua, Sara Solorzano	Centex Studies, Inc.	McAllen, TX
Stephen A. Spector, MD	Amaran Moodley, Jill Blumenthal, Baharin Abdullah, Christina Addington, Juan Carlos Alcantar, Deyna Arellano, Bernadette Cale, Brendan Costello, Tammelita Cotlon-Pineda, Fanny Delebecque, Karen Deutsch, Aram Dimayuga, Son Do, Yasmeen Esshaki, Aileen Everhart, Cindy Ewing,	University of California, San Diego	La Jolla, CA

Principal Investigator	Study Team	Institution	Location
	Veronica Figueroa, Medardo Gaytan, Crystal Groom, Carolyn Hernandez, Heather Huitema, Benjamin Hull, Sylvia Isaac, Jaclyn Jaskowiak, Cindy Knott, Leander Lazaro, Thuan Le, Megan Loughran, Michelle Madey, Rosalva Martha-Patten, Colleen McLellan, Jeff Ledford-Mills, Asami Mimura, Patty Moraes, Jennifer Morales, Jessica Nasca, Phirum Nguyen, Marielys Padilla-Martinez, Dennis Perpetua, Mike Pizza, Shannon Ransom, Emily Rizo, Carlos Rojas, Thaine Ross, Marie Sagrado, Eugene Sato, Lisa Stangl, Ji Sun, Nancy Tang, Mina Trivedi, Rodney Trout, Donna Voss, Lindsey Woronicz		
Cynthia Becher Strout, MD	Rica Santiago, Yvonne Davis, Patty Howenstine, Alison Bondell, Jaime Robertson, Anissa Moussa, Geronimo Feria Garzon, Sierra Bennett, Marlena Petrie	Coastal Carolina Research Center	Mount Pleasant, SC
Shobha Swaminathan, MD	Amesika Nyaku, Tilly Varughese, Rondalya Deshields, Michelle L DallaPiazza, Elise Lewis, Jennifer Punsal, Mario Portilla, Malithi Desilva, Christina Daliani, Susana Rivera, Aidan Ziobro, Andressa Rebellatto, Brian Murloy, Christina Ninan, Ernest Pianim, Eunice Wang, Merit Henen, Muhammad Usman, Rebecca Kim, Shiao Wang, Gener Eric Cruz, Bethany Birago, Joyell Arscott, Dina Meawad, Christie Lyn Costanza, Francesca Escaleira, Zoraida Cruz-Barahona, Jared Khan, Valeria Cadorett, Jamir Tuten, Travis Love, Eric Asencio, Sukhwinder Singh	Rutgers New Jersey Medical School	Newark, NJ
Ramy Joseph Toma, MD	Olivia Graves, Josiah Robinson, Patricia Hammonds, Lana Ghomrawi, Kara Quinnelly, Shaun O’Conor, Michael Lambe, Rachell Stewart, William Kirby, Pink Folmar, Rachel Culbreth, Heidi Leblanc, Julie McDaniel, Rian Montgomery, Andrea Woodle, Samantha Williams, Hunter Russell, Shereen Lowe, Maureen Mayer, Hollis Ryan, Elaine Reese	Synexus Clinical Research	Birmingham, AL
Timothy P. Vachris, MD	Mark Hutchens, Stephen Daniels, Margaret Wells, Sandra Clancy, Rebecca Martinez, Jessica Buot, Merissa Daugherty, Julie Hamilton, Kimberly Hernandez, Ashli Alejandro, Amy Collins, Monique Gawlik, Patricia Johnson, Maria Moreno, Ashley Washington, Tina Rountree, Daniel Dore, Ravi Davuluri, Ashlee Brunaugh, Jorge Martinez, James Hermon, Vianai Carreno, Mia Rountree, Colleen Coelho	Optimal Research	Austin, TX
Keith William Vrbicky, MD	Charles Harper, Chelsie Nutsch, Wendell Lewis III, Cathy Laflan, Linden DeBoer, Kayla Andal, Misty Appeldorn, Jenniger Grebe, Russell Herstein, Catherine King, Samantha Wieseler, Alisha Kiepke, Christy Lee, Kelsey Kelley, Kelli James, Ashley Frisch, Courney Green, Taysha Hingst, Jeni Hoppe, Kimber Breeden, Debra Gabrielson, Ginny McNew	Meridian Clinical Research	Norfolk, NE
Larkin T. Wadsworth III, MD	Ashley Dale, Christy Schultz, Rebecca Munsch, Anya Penly, Liz Garner, Stephanie Tesson, George Cherniawski, Angie Kean, Dan Reed, Courtney Kubiak, Maureen Dempsey, Heather Cherniawski, Breanna Galibert, Kristin Branson, Laura Hartupee, Karen Knapp, Horacio Marafioti, Lyly Dang, Jennifer Berry, Lauren Clement, Megan Dandurand	Sundance Clinical Research	St. Louis, MO
Jordan L. Whatley, MD	Patricia Whatley, Christopher Dedon, Anika Payne, Amie Shannon, Kristen Losavio, Nicole Harrell, Mary Margaret Dobson, Lindsey Hall, Chaney Bennett, Crystal Rowell, Mimi Dimmick, Amy Thomassie, Kimber Breeden, Cody LaFleur, Makaylea Truitt, Taryn Collett, Emily Best, Alexandra Caillouet,	Meridian Clinical Research	Baton Rouge, LA
Judith White, MD	Amy Edridge, Chelsea Montalvo, Eugenia Clark, Lisa Russell, Zahra Somji, Lesli Leimer, Robert Meyer, Christine Murphy, Prity Patel, Sejal Patel, Ruben Moliere, Samantha Merveillard, Yarnick Mirjah, Bryn Walls, Joey Cruz, Aaron Cooper, Jessica Bienaime, Ashley Gilcrist, Alisa Petit, Tyler Knightly, Kimberly Stokes, Christina Rosario, Talhia Matos, Ilona Boggs, Nicholas Weber, Felix Busot, Linda Colon, Heather Gillenwater, Cristina Kaplun, Melissa Caputi, Shayna Siplin, Daminee Shah, Samuel Martin, Alexis Waldorf, Vihar Upadhyay, Adolfo Henriquez, Saskia Singh, Maria Roberts, John Caporelli, Shirley Salvador, Quevina Scarver, Vanessa Garcia, Taylor Moore, Jayasen Singh, Curshinda Galvin-Burch, Mary Kesner, Jasmin Gil, Shay Gray, Steven Monsegur, Michele Steinmetz, Michael Lambe, Heather Powell, Sandra Torres, Shaban Katbeh, Taylor Wilson	Synexus Clinical Research	Orlando, FL
Priyantha N. Wijewardane, MD	Natalie Johnson, Martha Evans, Sondra Wright, Richard Pellegrino, Lastida Burns, Natasha Williams, Haylee Rowe, Kayla Graham, Amanda Horn, Eric Bravo, Jeffrey Thessing, A. Michele Maxwell, Amy Cooper, Lauren Evans, Tonya Cato, Haylee Tucker, Lesa Gann, Hannah Jones, Amanda May, Tiffany Walker, A. LeiAn Diaz, Laura Khalil, Lydia Purcell, Timothy Campbell, Charlotte Garcia-Velez, Andrea Scarborough, Beatrice A. Miller, Keith Bracy, Aujania Thompson, Cassandra Johnson, Krishana Day, Freddie Hicks, Jamie Pettus	Baptist Health Center for Clinical Research	Little Rock, AR

Principal Investigator	Study Team	Institution	Location
Barton G. Williams, MD	Flo Abbott, Nicole Burton, Alice Cipollini, Madison Croucher, Philip Dattilo, Erin Harrelson, Kelsey Heston, James Ingram, William H Jones, Karla Lane, Brandy Lowman, Evan Lucas, Megan Marles, Morgan Mathis, Angie Northcott, Clyda Pasquantonio, Alyssa Valente, Ciara Winders, Stephanie Graham	Trial Management Associates	Wilmington, NC
Marcus J. Zervos, MD	Paul Kilgore, Mayur Ramesh, Jelena Verkler, Pardeep Pabla, Andrew Clark, Katrina Williams, Dee Dee Wang, Beverley Duthie, Samia Arshad, Alandra White, Anna Kern, Ashley Mattern, Bilqis Mosed, Dana Parke, Doreen Dankerlui, Dragana Spasevska, Hanah Woods, Helina Misikir, Howard Klausner, Janay Scott, Jessica Heinonen, John Zervos, Joseph Miller, Kate Zenlea, Kristin Eis, Marissa Vasquez, Maurice Slaughter, Meaghan Flynn, Michael Garcia, Michelle Sankah, Nina Paeilli, Philip Benson, Robert Devore, Stevanya Baho, Tony Eljallad, Tyler Prentiss, Yaman Ahmed, Sharon Mathys, Linda Kaljee, Jeffrey Van Laere, Claudia Hanni, Hassan Zafar, Mona Desai, Gina Maki, Mary Perri, Dora Vager, Shannon Thomas, Autumn Robinson, Isis Hamilton, Sonia Eliya, Jehan Jazrawi, Biljana Popovic, Sharon Zahul, Joshua Ruzzin, John Laguio, Ali Mathena, Bobby Cook Jr., Marlene Hesler, Rochelle Fleming, Terria Minniefield, John Simons, Sherese Henderson, Ashley Hopkins, Rebecca McFarlane, Raeshell Carson, Jonathan Williams, Katherine Reyes, Erica Herc, Indira Brar, Mayur Ramesh, John McKinnon, Lacquis Duncan, Tim Asmar, Margaret Beyer, Kaleem Chaudhry, Madison Lee, Jo-Ann Rammal, Karthik Sridasyam, Siddesh Veer, Angelique Buluran, Kimberlyn Lott, Jeremiah Rooker, Alayna Wilder, Kathleen Wilson, Allison Weinmann, Hassan Mourtada	Henry Ford Health System	Detroit, MI

Supplementary Methods

Statistical analysis

Direct comparisons of the effectiveness of the primary series between the mRNA-1273 and placebo-mRNA-1273 groups are not provided given the potential variation in background disease incidence for these groups, which precludes meaningful conclusions. Notably, participants from the originally randomized groups in Part A crossed over into other studies (ie, primary series recipients were eligible for the booster dose parts of phase 2 studies P201 and P205); thus, the two groups not only became increasingly de-randomized, but their relative geographical distribution changed and, as a consequence, the timing of increased incidences due to variant waves of SARS CoV-2 was not consistent across these groups. Analysis sets and treatment groups and analysis periods used for effectiveness analyses are provided in Tables S1 and S2.

Analysis of the Primary Effectiveness Endpoint for Parts B and C, and Long-term Analyses

Primary Efficacy Endpoint Definition/Derivation

Vaccine effectiveness was demonstrated based on the primary efficacy endpoint in the blinded phase (Part A) of first occurrence of COVID-19 starting 14 days after the second injection in the blinded phase (Part A). Parts B and C are open-label (OL) and offered participants an opportunity to receive the primary series and booster of mRNA-1273, respectively. Throughout the study, COVID-19 was assessed in the same way as the primary efficacy endpoint in the blinded phase (Part A) through adjudication of potential COVID-19 and severe COVID-19 by an independent external Adjudication Committee. Derivation of COVID-19 cases based on RT-PCR test results and symptoms is the same as used for the primary efficacy endpoint (Part A) requiring at least 2 eligible systemic symptoms (fever ≥38ºC, chills, fatigue, myalgia, headache, new loss of taste or smell, sore throat) or 1 respiratory (cough, shortness of breath or difficulty breathing, clinical or radiographical evidence of pneumonia) symptom, for the primary series and after the booster.[1]

Surveillance for COVID-19 was performed through weekly contact with participants via a combination of telephone calls and completion of an eDiary starting at day 1 through the end of the study. Participants with symptoms of COVID-19 lasting at least 48 hours (except for fever and/or respiratory symptoms) returned to the clinic or were visited at home by medically qualified site staff within 72 hours to collect a nasopharyngeal swab sample for RT-PCR testing for SARS-CoV-2 and other respiratory pathogens. Alternatively, if a clinic or home visit was not possible, the participants themselves submitted a saliva sample for RT-PCR testing.

In the Part B open-label phase of the study, all participants had a scheduled nasopharyngeal (NP) swab for RT-PCR test and blood collected for immunologic analysis at the participant decision visit (PDV). Participants who received mRNA-1273 in the open-label phase had an additional blood sample collected at OL Day 29 or OL Day 57 per the planned number of mRNA-1273 doses received in the OL phase. For Part B analysis and analysis of the primary series, participants with early infection, as detected by positive RT-PCR at scheduled visits (including Day 29 and PDV), or positive bAb against SARS-CoV-2 nucleocapsid protein at scheduled visits, were censored at the time of early infection. If a participant had eligible symptom(s) within 14 days of positive RT-PCR at scheduled visits, the participant is considered to be a case for COVID-19. For analysis of Part B or primary series, participants who had no documented COVID-19 were censored at the earlier date of date of booster, study-end database

lock, or date of last observed in study defined as the earlier of study completion date, early discontinuation date from study, or date of death.

In the Part C booster dose phase of the study, all participants had scheduled blood sample collected at BD Day 1 visit prior to the booster dose. NP swab test at this visit is optional. Participants who received the booster dose of mRNA-1273 in Part C had blood samples collected at BD Day 29 and BD Day 181 (6 months post-booster). For Part C, participants with early infection, as detected by positive RT-PCR at scheduled visits, or positive bAb against SARS-CoV-2 nucleocapsid protein at the scheduled visits, were censored at the time of early infection. If a participant had eligible symptom(s) within 14 days of positive RT-PCR at scheduled visits, the participant is considered to be a case for COVID-19. Participants who had no documented COVID-19 were censored at the earlier date of database lock or data-cutoff date for efficacy, date of last observed in study defined as the earlier of study completion date, early discontinuation date from study, or date of death.

Analysis approach for effectiveness of vaccine

Effectiveness of the primary series

Effectiveness of the primary series of mRNA-1273 was assessed in participants initially randomized to the mRNA-1273 group (mRNA-1273, vaccinated from July-December 2020, early vaccinated) and those initially randomized to the placebo group (placebo-mRNA-1273, vaccinated December 2020-April 2021, late vaccinated) in the per-protocol (PP) primary series set. The PP primary series set is comprised of participants randomized to mRNA-1273 in Part A who had no evidence of infection prior to injection 1 (baseline/pre-Day 1 SARS-CoV-2-negative test) and those in the placebo-mRNA-1273 group who were SARS-CoV-2 negative at PDV/unblinding at the start of Part B. Parts A and B data after injection 1 of mRNA-1273 are included for this analysis, and date of injection 1 of mRNA-1273 is used as the reference.

Incidence is provided over the time from injection 1 of mRNA-1273 to receipt of booster, last study participation, or data-cutoff date and are also provided by calendar quarters, as well as by time intervals including after injection 1 of mRNA-1273, after injection 1 to before injection 2 of mRNA-1273, after injection 2 of mRNA-1273, injection 2 to <14 days after injection 2 and ≥14 days after injection 2 (this interval was further partitioned into shorter intervals). For each interval, person-time was calculated for participants at risk at the beginning of the interval, as the time from the date of the beginning of the time interval to the date of the event/censoring for participants with an event/censor if the event/censor occurs in the interval; or the time of the interval for participants who continue to be at risk at the end of the interval. Incidence rate (IR), and 95% CI for IR were calculated using the exact method adjusting for person-time will be provided by treatment cohort. The number and IRs of COVID-19 starting 14 days after injection 2 of mRNA-1273 are also be provided by calendar quarters and for the periods defined by emergence and circulation of select variant of interest including Delta (September 1, 2021, to November 30, 2021), and Omicron BA.1 (December 1, 2021, to March 31, 2022), Omicron BA.2 (April 1, 2022, to June 30, 2022), BA.4/5 (July 1, 2022, to November 30, 2022) and Omicron BQ.1.1 (December 1, 2023, to April 7, 2023) variants.

Relative Effectiveness of late vs early vaccinated (the Primary Series of mRNA-1273)

Relative effectiveness of recent vaccinated participants (Placebo-mRNA-1273) vs. early vaccinated participants (mRNA-1273) are assessed using the reduction in incidence, calculated as 1- incidence ratio pre-boost. The 95% CI of reduction in IR was calculated using the exact

method (Poisson distribution). The above analysis was performed by calendar quarters and time periods.

Effectiveness of the 50-µg mRNA-1273 booster

For analysis of COVID-19 incidence after the booster, the Part C PP set was used; participants at risk at the start of Part C were defined as participants who did not have COVID-19 and had no SARS-CoV-2 infection prior to Part C. Incidence after booster, as well as by calendar months (if feasible), and by time periods including After Booster, Booster to <14 days after booster, ≥14 days after Booster are provided. Incidence and 95% CI based on the exact method adjusting for person-time are provided by treatment cohort. Number and IRs of COVID-19 starting 14 days after the booster are also provided by calendar months along with 2 additional time intervals associated with Delta (September 1, 2021, to November 30, 2021), and Omicron BA.1 (December 1, 2021, to March 31, 2022), Omicron BA.2 (April 1, 2022, to June 30, 2022), BA.4/5 (July 1, 2022, to November 30, 2022) and Omicron BQ.1.1 (December 1, 2023, to April 7, 2023) variant waves.

Effectiveness of booster was evaluated based on IRs of COVID-19, using the PP primary series set. Number of events and IRs are provided using a dynamic approach for booster and non-booster groups (eg, a participant without COVID-19 or SARS-COV-2 infection was no longer considered as "at risk" in the non-booster group from the time point when this participant received the booster dose, instead, this participant was considered as "at risk" in the booster group from that time point). Incidences were calculated as the number of participants with an event divided by the number of participants at risk adjusted by person-time (months) in each treatment cohort. Number of cases, incidences, and 95% CI are provided for each treatment group, and reduction in incidence (1-incidence).

Subgroup Analysis

Subgroup analyses of the effectiveness endpoints of COVID-19 and severe COVID-19 were performed in select subgroups specified (age groups [≥18 and <65 years, ≥65 years], stratification at randomization [≥18 and <65 years and not at risk, ≥18 and <65 years at risk, ≥65 years], and sex (female, male), ethnicity, race and ethnicity (non-Hispanic white, communities of color) and risk factor for severe COVID-19 illness [for severe COVID-19 endpoint]). The effectiveness endpoints of COVID-19 were analyzed by each of the subgroups using the same methods described for the primary analysis.

Analysis of Other Effectiveness Endpoints

Other effectiveness endpoints were evaluated in the PP primary series set for the primary series with appropriate analysis periods and Part C using the Part C PP set. Derivation of other effectiveness endpoints are as defined in the previously reported primary efficacy analysis with modifications below.[2,3]

Severe COVID-19

Severe COVID-19 was defined per the COVE protocol[2,3] and FDA guidance[4] as the first occurrence of a confirmed case of COVID-19 as per the primary efficacy endpoint starting 14 days after the second dose of vaccine or placebo AND any of the following: 1) clinical signs indicative of severe systemic illness, respiratory rate ≥30 per minute, heart rate ≥125 beats per minute, oxygen saturation ≤93% on room air at sea level or arterial partial pressure of

oxygen/fraction of inspired oxygen <300 mmHg, OR 2) respiratory failure or acute respiratory distress syndrome (defined as needing high-flow oxygen, non-invasive or mechanical ventilation, or extracorporeal membrane oxygenation), evidence of shock (systolic blood pressure <90 mmHg, diastolic BP <60 mmHg or requiring vasopressors), OR 3) significant acute renal, hepatic or neurologic dysfunction, OR 4) admission to an intensive care unit or death.

CDC Definition of COVID-19

The CDC definition of COVID-19 cases was defined as at least one systemic symptom (fever ≥38ºC, or chills, cough, shortness of breath or difficulty breathing, fatigue, muscle aches or body aches, headache, new loss of taste or smell, sore throat, nasal congestion or rhinorrhea, nausea or vomiting, or diarrhea) AND a positive NP swab, nasal swab, or saliva sample (or respiratory sample, if hospitalized) for SARS-CoV-2 by RT-PCR.[1] This definition was derived using the censoring rules for the effectiveness endpoint of COVID-19 cases (participants with early infection as detected by positive RT-PCR at scheduled visits including day 29 and PDV, or positive bAb against SARS-CoV-2 nucleocapsid protein in blood samples at the scheduled visits and scheduled visits in BD phase are censored at the time of early infection. If a participant had eligible symptom(s) within 14 days of positive RT-PCR at scheduled visits, the participant was considered to be a case for the CDC definition of COVID-19.

Asymptomatic SARS-CoV-2 Infection

Asymptomatic SARS-CoV-2 infection was defined as the absence of symptoms and infections detected by seroconversion or positive RT-PCR at scheduled visits applicable to individual treatment cohorts for Parts B and C, and analysis of primary series with corresponding analysis periods as absence of symptoms (no COVID-19 symptoms by either effectiveness endpoint of COVID-19, or CDC definitions of COVID-19), AND at least one post-baseline positive RT-PCR at scheduled visits, or seroconversion (bAb specific to SARS-CoV-2 nucleocapsid) at scheduled visits (PDV, OL Day 29, and OL Day 57 in Part B; booster dose days 1, 29 and 181 (BD-1, BD-29, and BD-181, respectively in Part C), when blood samples for immunogenicity are collected.

Serologically Confirmed SARS-CoV-2 Infection or COVID-19 Regardless of Symptomatology or Severity

SARS-CoV-2 infection regardless of symptoms was a combination of COVID-19 and asymptomatic SARS-CoV-2 infection, as defined above.

Death caused by COVID-19

Death caused by COVID-19 was defined as due to a cause directly attributed to a complication of COVID-19, starting 14 days after the second injection of study vaccine.

Immunogenicity Analysis

The Part C PP immunogenicity set (PPIS) was used for immunogenicity analyses and are summarized by pre-booster SARS-CoV-2 status. The Part C-PPIS consists of a subset of participants who were in the PP random subcohort for immunogenicity in Part A[5], were randomized to mRNA-1273 in Part A, were baseline/pre-day 1 SARS-CoV-2 negative and received booster in Part C.

The Part C PPIS–pre-booster SARS-CoV-2-negative (Part C-PPIS-Neg) set was used as the primary analysis population for comparison of the immune response after the booster and primary series of mRNA-1273. The Part C PPIS-Neg consists of participants who are in the Part

C PPIS and are pre-booster SARS-CoV-2-infection negative, defined as no virologic or serologic evidence of SARS-CoV-2 infection on or before BD-1(pre-booster), i.e. RT-PCR result was not positive if available at BD-1 and a negative bAb specific to SARS-CoV-2 nucleocapsid (as measured by Roche Elecsys Anti-SARS-CoV-2 assay) on or before BD-1. Part C PPIS-Neg was used for analysis of immune response to booster, especially, to be compared with immune response after primary series of mRNA-1273 to infer immunogenicity of a booster. Comparison of immune response was also performed in all participants in the PPIS; due to the limited size of the PPIS-positive group, immune response comparison was not performed and immunogenicity is summarized for this subgroup. Immunogenicity data at days 209 after the primary series and 181 post-booster in the PPIS participants and by SARS-CoV-2-infection pre-booster status as well as by age group and SARS-CoV-2-infection pre-booster status are also presented.

Immunogenicity analyses for neutralizing antibodies (nAb) and binding antibodies (bAb) included the geometric mean concentration (GMC [AU/mL]) for nAb and GM level for bAb with corresponding 95% CI provided at each time point, and geometric mean fold-rise (GMFR) of titer with corresponding 95% CI provided at each post-baseline time point over the corresponding baseline. GMFR was calculated based on pre-vaccination/pre-injection 1 (Day 1) of the primary series, and pre-booster baseline (BD-1 of Part C, where applicable). Seroresponse (SRR), defined as proportion of participants with seroresponse, was provided with 2-sided 95% Clopper-Pearson Cis. SRR to the booster was assessed based on the pre-vaccination baseline (pre-injection 1) at day 1 of the primary series or the pre-booster baseline (BD-1 of Part C) and was defined as a ≥4-fold rise for those with a baseline value ≥LLOQ, or 4 × LLOQ for those with a baseline value <LLOQ. For 4-fold rise definition compared with pre-vaccination (pre-injection 1 of the primary series), a baseline value of <LLOQ at pre-injection 1 of the primary series was assumed if no pre-injection 1 Ab titer data was available and there was evidence that the participant did not have SARS-CoV-2 infection prior to vaccination (ie, negative SARS-CoV-2 status at pre-vaccination/pre-injection 1).

Immunobridging analyses of the booster dose compared with the primary series, was determined by non-inferiority of the nAb response 28 days following the booster dose (BD-29 29) compared with Day 57 of Part A for the mRNA-1273 cohort (28 days following injection 2 of the mRNA-1273 primary series). The key secondary endpoints assessed were the geometric mean ratio (GMR) comparison of the GM booster (Part C BD-29) to the GM of mRNA-1273 primary series (Part A Day 57) and the difference in SRR:SRR of the booster (Part C seroresponse [BD-29 compared with BD-1]) – SRR mRNA-1273 primary series (Part A seroresponse [Day 57 compared with Day 1]) for nAb. The 95% CI of the GMR and 95% CI of SRR difference was used to assess the non-inferior/superior immune response of a 50-µg mRNA-1273 booster (Part C) as compared with the 100-µg mRNA-1273 primary series (Part A) as described in Table S4. Criteria for non-inferiority were considered met when the lower bound of the 95% CI of the GMR was >0.67 and the lower bound of the 95% CI of the SRR difference was >-10%, and for superiority if the lower bound of the 95% CI for GMR was >1 and the lower bound of the 95% CI for SRR difference was >0.

Preliminary analyses of the impact of time intervals between the primary series and booster on antibody responses among PPIS-negative mRNA-1273 participants and in a subset of placebo-mRNA-1273 PPIS-negative participants with non-missing time intervals and immunogenicity results at BD-1 were also performed.

Exploratory Endpoints

Genotypic Analysis of Variants

Genotypic analysis of variants was performed in SARS-CoV-2 PCR-positive NP samples collected from participants following the primary series and the booster in Part C. Samples were collected from July 2020 (start of Part A) through April 2022 (data-cutoff date date). Whole-genome sequencing was performed using residue samples initially collected for the qualitative detection of RNA from SARS-CoV-2. Values reported in the RT-PCR assay for cycle threshold were reviewed to determine which samples would be analyzed for detection and sequencing of SARS-CoV-2 virus genomic RNA. A cutoff cycle threshold value of <28 was applied to ensure successful sequencing. The sequences detected in adjudicated case samples were representative of the circulating strains in the US during the trial, notably delta and omicron SARS-CoV-2 variants.

Exploratory analysis of effectiveness

Pre- and post-boost vaccine effectiveness were explored using a Cox Proportional Hazards Model with a time-varying covariate that estimated the risk of first occurrence of COVID-19 in the Placebo-mRNA-1273 and mRNA-1273 groups (Figure S11).[6] The model was adjusted for baseline factors (sex, stratification factor at randomization, risk score, early unblinding) as potential confounders of the relationship between boosting time and COVID-19 risk. The analysis population (PP-primary set) consisted of 20,404 participants who remained in the study and did not have adjudicated COVID-19 cases as of 23 September 2021 (the first date study participants were boosted) with no evidence of prior SARS-CoV-2-infection. This included 17,025 boosted participants and 3379 un-boosted participants through January 31, 2022. Participants who remained un-boosted through January 31, 2022, were censored on that date for the analysis. Days since September 23, 2021, were used as the time index to adjust for variation in the risk of COVID-19 through data-cutoff date. As boosting was not randomized, the mRNA-1273 and placebo-mRNA-1273 groups were evaluated in terms of boost initiation with a covariate of time since vaccination in the model. Early boosted participants were those in the original mRNA-1273 arm who received booster before October 25, 2021 (median of booster dates) and late boosted participants were those in the mRNA-1273 arm who received booster on or later than October 25, 2021 (median of booster dates). The Cox model with time-varying variables also allowed evaluation of the durability of the boost effect. The pre- and post-boost efficacy against Delta and Omicron COVID-19 cases were evaluated separately.

A simple example was used to illustrate the Cox model-approach, assuming a cohort of 20,000 individuals who were boosted over 4 time periods and at the start of a period and has its entire effect the same day.

	**Number of cases/number at risk**				
**Period**	**Unboosted**	**Boosted Current Period**	**Boosted 1 Period Before**	**Boosted 2 Periods Before**	**Boosted 3 Periods Before**
1	20/15000	1/5000	-	-	-
2	40/9980	2/5000	2/4999	-	-
3	10/4940	1/5000	3/4998	3/4997	-
4	-------	8/4930	1/4999	5/4995	8/4994

An estimate of immediate boost effectiveness averages unboosted and boosted current periods; 1 – (1/5000)/(20/15000), 1 – (2/5000)/(40/9980), and 1-(1/5000)/(10/4940) which equals 0.88. The effectiveness for those estimated one period ago is the average of 1-(2/4999)/(40/9980) and

1-(3/4998)/(10/4940) or 0.80, and for those boosted 2 periods is estimated as 1 – (3/4997)/(10/4940) = 0.70. Note that these estimates do not depend on the assumption of a constant attack rate for the unboosted over the 4 periods and basically follows the above approach but has a large number of periods and assumes that booster effectiveness decays smoothly with time since priming series and allows a bend-point at C.

An illustrative version of the model is given by the hazard function:

$$(t) = h_0(t)\exp\{Z_i(t)[\beta_0 + \beta_1 I(A_i = 1) + \beta_2(t - v_i) + \beta_3(t - v_i - C)I(t - v_i > C)] + \beta_4[1 - Z_i(t)]I(A_i = 1) + X_i\theta\}I(t \in R_i),$$

where *t* is the number of days since 23 September 2021, C is a bend-point, (45 days for Delta, 90 days for omicron), A=1 for the mRNA-1273 arm and A=0 for the Placebo-mRNA-1273 arm, v is the calendar date of boosting, Z(t) is 1 if ≥ 14 days after boosting and 0 before boosting, X is a vector of covariates, I is the indicator function, and R denotes the intervals of risk for a person with II 1 during intervals of risk and 0 otherwise. R includes the interval [0,T] where T is the time to COVID-19, or censoring, but excludes the 14 days post booster, for those who receive a booster. Outside the intervals of risk, an individual is not in the risk set of the partial likelihood. We take C to be approximately the median of the times from boost to COVID-19. The baseline hazard h_0 (t) is the pre-boost hazard for a person with A=0.

Under this model we define booster effectiveness as a function of v=time since boost for the Placebo-mRNA-1273 arm and mRNA-1273 arms, respectively, as given below.

$$\mathrm{BE}(v; A = 0) = 1 - exp\{\beta_0 + \beta_2 v + \beta_3(v - C)\mathrm{I}(v - C > 0)\}$$

$$\mathrm{BE}(v; A = 1) = 1 - exp\{\beta_0 + \beta_1 + \beta_2 v + \beta_3(v - C)\mathrm{I}(v - C > 0) - \beta_4\}$$

We define pre-boost relative effectiveness of the mRNA-1273 arm (A=1) to the Placebo-mRNA-1273 arm (A=0) as

$$\mathrm{REpre} = exp(\beta_4).$$

Post-boost relative effectiveness of the mRNA-1273 arm (A=1) to the Placebo-mRNA-1273 arm (A=0) is defined as

$$\mathrm{REpost} = exp(\beta_1).$$

The above models were estimated separately for Delta and Omicron cases using a Prentice approach to competing risks where for the Delta model, Omicron cases are censored at the time of COVID-19 and vice versa for the omicron model. For cases with missing variant information, SARS-CoV-2 variants were imputed using the GISAID database where for each week and each state (eg, Maryland, 1st week of December) the proportion of Delta and Omicron cases are estimated as p and 1-p, where p is the average for that week. Missing variants were imputed using a Bernoulli(p) model. Because p is nearly 0 or 1 throughout follow-up, multiple imputations give virtually the same estimates as single imputation and a singly imputed dataset was used.

Immunogenicity Assays

Pseudovirus neutralization assays

For the assessment of ancestral SARS-CoV-2 (D614G) neutralizing antibodies, post-vaccination serology samples from participants were tested using a validated reporter virus microneutralization assay (PPD, part of Thermo Fisher Scientific Vaccines Laboratory Services, Richmond, Virginia).[7,8] This cell-based assay measures the SARS-CoV-2 neutralizing antibody inhibition of the infection of 293T-ACE2 cells by SARS-CoV-2 reporter virus particles (RVP: Wuhan-Hu-1 isolate including D614G). The serum antibody concentration is determined by interpolating the mean of the replicate foci forming unit values from the fitted reference standard curve calibrated to the first WHO International Antibody Standard for SARS-CoV-2 Lot 20/136. The interpolated antibody concentrations were dilution corrected, and the final concentration is the antibody concentration associated with the lowest dilution with an antibody concentration within the quantifiable range of the assay. The results are reported as final antibody geometric mean concentration (GMC) in AU/mL.

Meso Scale Discovery (MSD) Multiplex Spike Assay binding antibody assay

The MSD Multiplex assay for the detection of IgG against SARS-CoV-2 spike (S-2P protein; Wuhan-Hu-1 isolate including D614G) was validated (precision, ruggedness, relative accuracy, dilutional linearity, specificity, and selectivity), by the NIH Vaccine Research Center (Rockville, MD), Vaccine Immunology program for the detection of bAb against the S-2P protein, the receptor binding domain (RBD), and Nucleocapsid (NP).

The MSD Platform utilizes multi-spot microtiter plates fitted with a series of electrodes on the bottom of each well. Using an MSD plate imager, an electrical current was placed across the electrodes, causing a series of oxidation-reduction reactions involving Ruthenium and Tripropylamine leading to a luminescent signal. The V-PLEX SARS-CoV-2 Panel 2 (IgG) Kit was used to measure IgG antibodies to three antigens related to SARS-CoV-2, including SARS-CoV-2 N, SARS-CoV-2 S1 RBD, and SARS-CoV-2 S antigens. Plates were provided with antigens on spots in the wells of a 96 well plate. Antibodies in the serum samples bind to the antigen(s) on the spots and anti-human antibody (IgG) conjugated with MSD SULFO-TAG TM are used for detection. The plate was read on an MSD instrument, which measured the light emitted from the MDS SULFO-TAG. Serum samples, collected from individuals (n=84) with confirmed or suspected COVID-19 diagnosis between March 2020 and September 2020 were reference controls. All sera (S2-P and RBD) were collected under an IRB-approved protocol at the National Institutes of Health, Bethesda MD (VRC 200, clintrials.gov NCT00067054). For conversion of the MSD assay AU/ml to Binding Antibody Units/ml (BAU/ml), based on the WHO International Standard for anti-SARS-CoV-2 immunoglobulin the three binding antibody variables CoV-2 S IgG, CoV-2 RBD IgG, and CoV-2 N IgG, based on WHO International Standard for anti-SARS-CoV-2 Immunoglobulin, multiply by conversion factors 0.0090, 0.0272, and 0.0024, respectively.[9]

Figure S1. Parts B and C Analysis Set Populations

A. Part B

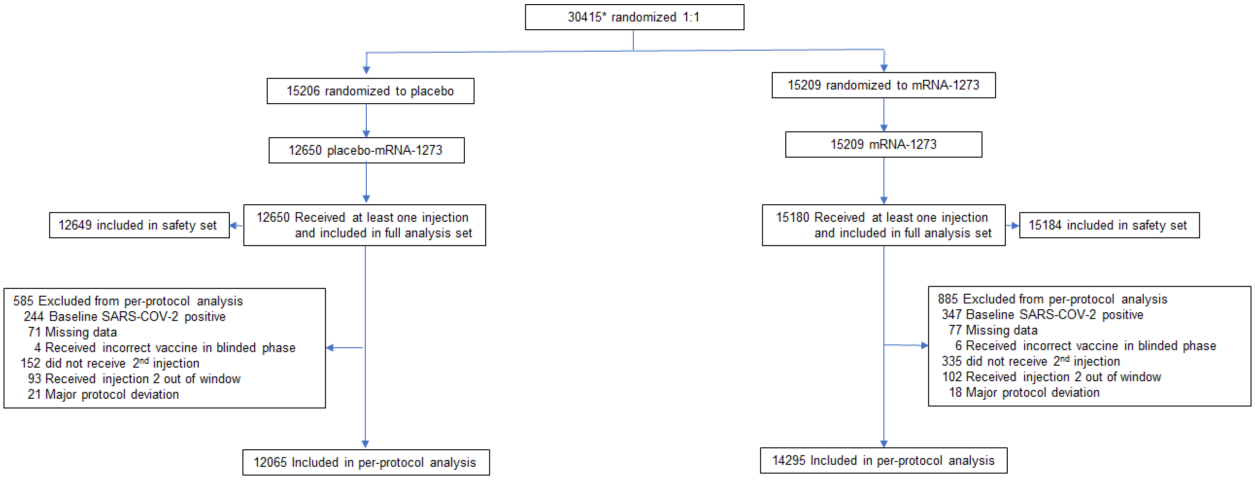

B. Part C

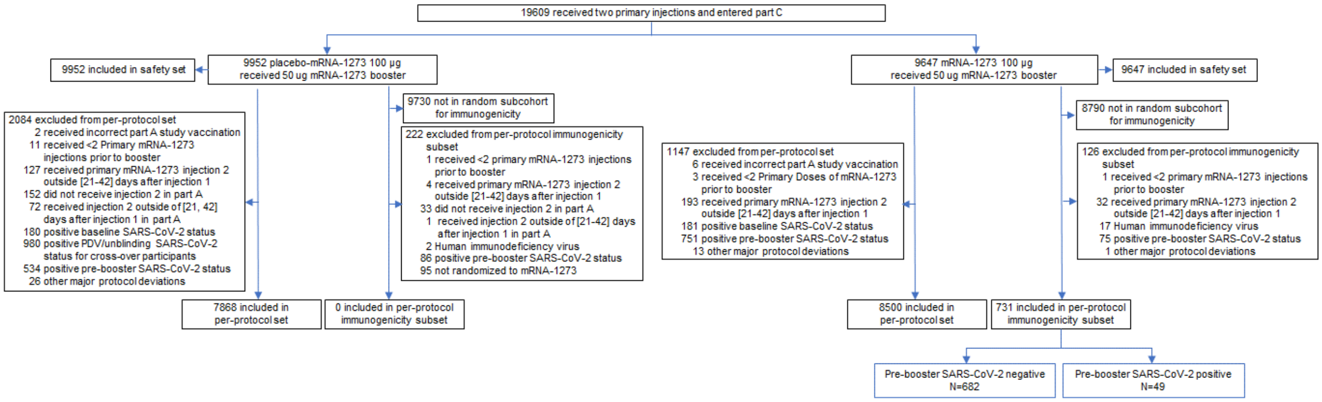

Figure S1. Panel A. Part B Phase includes those who had a participant decision visit or unblinding date. The full analysis set includes all participants who were randomized and received at least one injection of the mRNA-1273 primary series or placebo in Part A and corresponds to the randomized study vaccinations treatment group and study vaccinations. The primary series safety set includes all participants who were randomized and received at least one injection of the mRNA-1273 primary series or placebo in Part A and corresponds to the actual study vaccinations that participants received; the primary series safety set was used for the safety analysis of the mRNA-1273 primary series. The per-protocol primary series set consists of all participants who received two injections of mRNA-1273 primary series per schedule in Parts A or B, with negative pre-injection 1 SARS-CoV-2-infection status, and no major protocol deviations that impacted critical or key study data. The per-protocol primary series set was used to assess effectiveness of the mRNA-1273 primary series as well as effectiveness of participants who received booster and did not receive a booster dose by pre-booster SARS-CoV-2 status. **Panel B**. Ten placebo participants who received a booster dose were included in the safety set and excluded from the per-protocol set. The Part C Safety set includes participants who were randomized in Part A and received an mRNA-1273 50-µg booster in Part C and was used for all analyses of safety in Part C. Part C per-protocol set comprised all participants who were randomized in Part A, received a

mRNA-1273 50-µg booster dose in Part C after the primary series per schedule, were pre-booster SARS-CoV-2 negative, and had no major protocol deviations that impact critical or key data and was used for analysis of the effectiveness of the booster. Participants who did not receive the treatment as randomized in Part A, did not receive 2 injections of the primary series of mRNA-1273, or did not receive injection 2 of mRNA-1273 within [21-42] days after injection 1 were excluded from the Parts B and C per-protocol sets. Part C per-protocol immunogenicity subset (PPIS) consisted of participants in the Part C per-protocol random subset for immunogenicity (PPRSI) in Part A who were randomized to mRNA-1273 in Part A, were baseline/pre-day 1 SARS-CoV-2 negative and received a booster in Part C and is a subset of participants in the PPRSI in Part A and Part C PPRSI and was used to summarize immune response after the booster in Part C. The Part C PPIS-negative groups consisted of participants who were in Part C PPIS and were pre-booster SARS-CoV-2 negative (no evidence of SARS-CoV-2 infection pre-booster on or before BD-1 (ie, negative RT-PCR result if available at BD-1 and a negative bAb specific to SARS-CoV-2 nucleocapsid (Roche Elecsys Anti-SARS-CoV-2 assay) on or before BD-1.

Figure S2. Part C Per-Protocol Immunogenicity Set

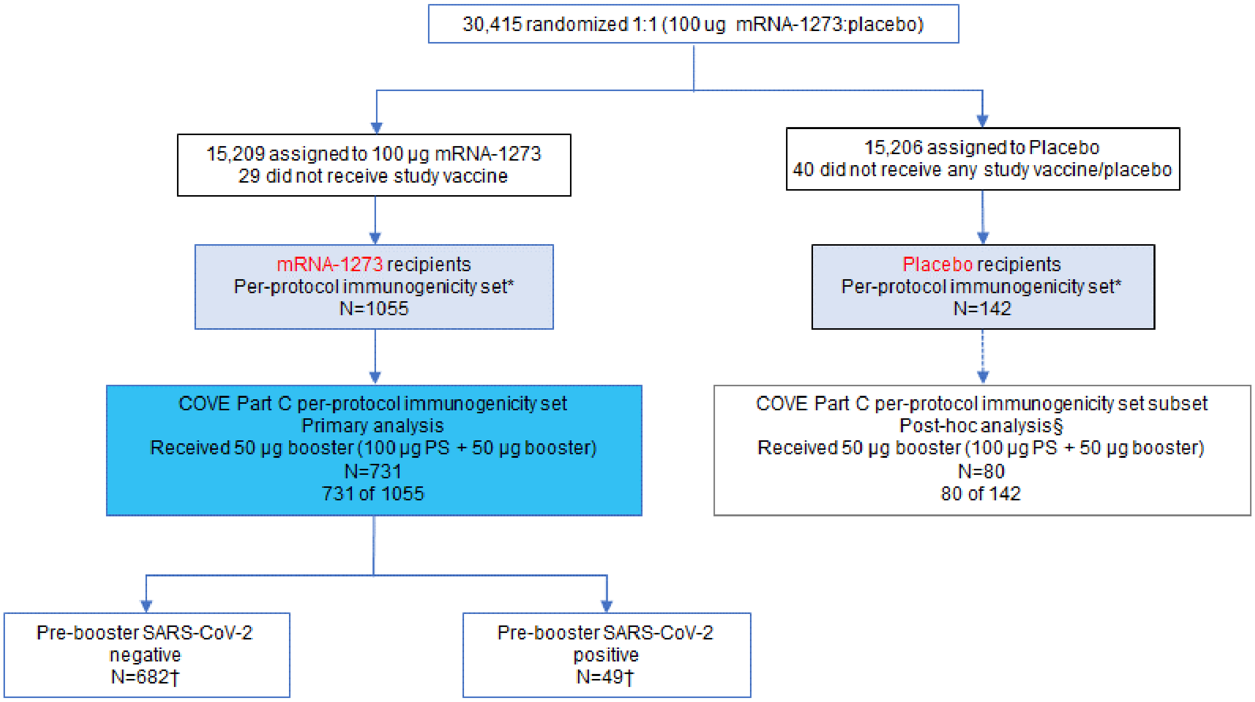

Figure S2. BD-1=booster dose day 1; PS = primary vaccination series of 2 injections 100 µg mRNA-1273. *Per-protocol immunogenicity set (PPIS) of randomly selected immunogenicity subcohort in previously reported immunogenicity analysis of blinded Part A of COVE study.[25] †The Part C PPIS-Neg set consists of participants who are in Part C PPIS and are pre-booster SARS-CoV-2-infection negative, defined as negative virologic (RT-PCR) or serologic (SARS-CoV-2 nucleocapsid measured by Roche Elecsys) tests on or before BD-1 (pre-booster); the PPIS-Neg is the primary analysis set used for the comparison of immune responses after the booster and primary series of mRNA-1273 to infer effectiveness of a booster. Immunogenicity was also assessed in mRNA-1273 participants who were SARS-CoV-2-infection positive at pre-booster (BD-1). §A post-hoc interval analysis was performed in a subset of placebo-mRNA-1273 PPIS-negative participants from the COVE PPIS.

Figure S3. Severe COVID-19 Events Based on Adjudication Committee Assessments Starting 14 Days After Booster Across Subgroups (Part C Per-Protocol Set)

Subgroups	Events/N (%) N=16368	Incidence Rate (95% CI) [1]
Overall	167/16006 (1.0)	1.15 (0.98-1.34)
Age Group		
≥18 and <65 Years	117/11310 (1.0)	1.10 (0.91-1.32)
≥65 Years	50/4696 (1.1)	1.28 (0.95-1.68)
Age & Health Risk for COVID-19 [2]		
≥18 and <65 Years and Not at Risk	92/8835 (1.0)	1.10 (0.89-1.35)
≥18 and <65 Years and at Risk	25/2475 (1.0)	1.10 (0.71-1.62)
≥65 Years	50/4696 (1.1)	1.28 (0.95-1.68)
Sex		
Male	57/8348 (0.7)	0.75 (0.57-0.97)
Female	110/7658 (1.4)	1.59 (1.31-1.92)
Ethnicity		
Hispanic or Latino	28/3053 (0.9)	0.96 (0.64-1.39)
Not Hispanic or Latino	138/12791 (1.1)	1.20 (1.01-1.42)
Not Reported/Unknown	1/162 (0.6)	0.68 (0.02-3.80)
At Risk for Severe COVID-19 at Screening		
Yes	45/3854 (1.2)	1.32 (0.96-1.77)
No	122/12152 (1.0)	1.10 (0.91-1.31)
Race and Ethnicity Group [3]		
White	120/10262 (1.2)	1.32 (1.09-1.58)
Communities of Color	47/5710 (0.8)	0.87 (0.64-1.15)
Missing	0/34	0.00 (NE-11.51)

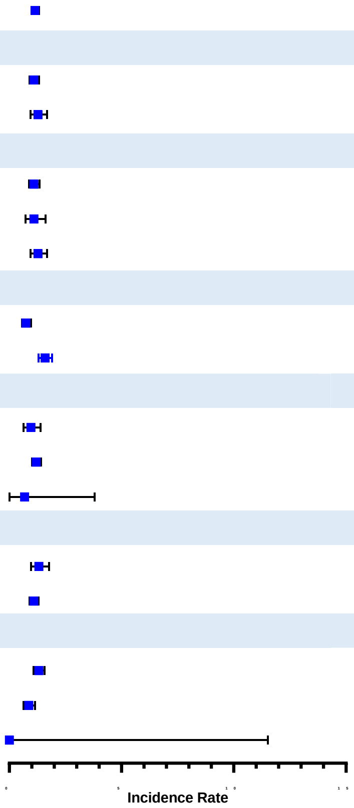

Figure S3. CI = confidence interval; COVID-19 = coronavirus disease 2019; IR = incidence rate; N = number of participants at risk. Incidence rates among subgroups following administration of mRNA-1273 as a 50-µg booster dose. Error bars represent 95% confidence intervals. Incidence is defined as the number of participants with an event starting 14 days after booster by the number of participants at risk at 14 days after booster and adjusted by person-months (total time at risk) in each treatment group. 1 month = 30.4375 days. White is defined as White and non-Hispanic, and communities of color includes all the others whose race or ethnicity is not unknown, unreported, or missing. Participants at risk for severe COVID-19 included those aged ≥65 years and <65 years with at least one of the risk factors (chronic lung disease, significant cardiac disease, severe obesity, diabetes, liver disease, or HIV infection).

Figure S4. Neutralizing Antibodies Against Ancestral SARS-CoV-2 (D614G) After mRNA-1273 Primary Series and Booster, Part C

A. All Partcipants in PPIS

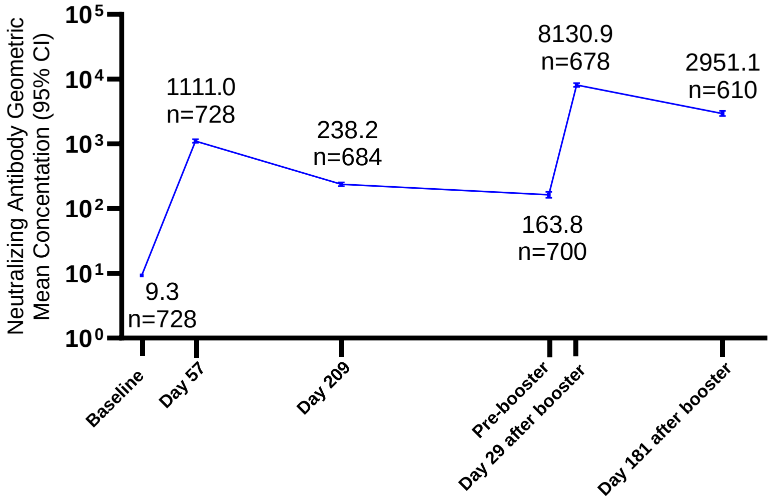

B. SARS-CoV-2 Pre-booster Status

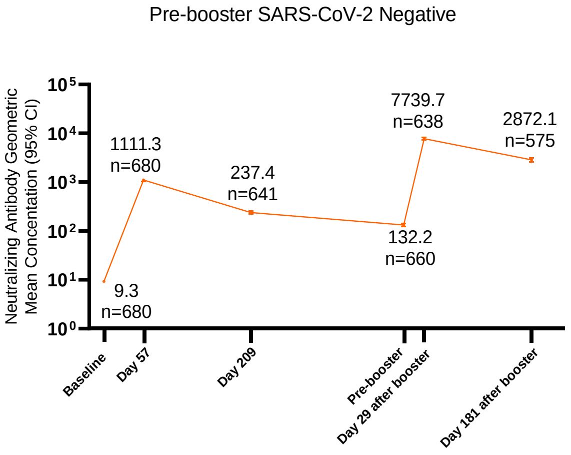

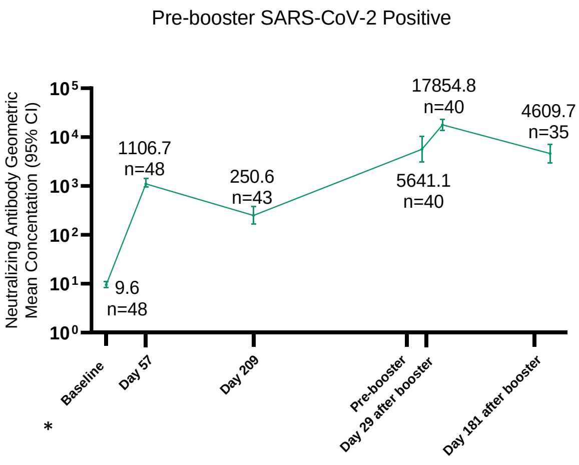

Figure S4. BD = booster dose; CI = confidence interval; LLOQ = lower limit of quantification; PPIS = per-protocol immunogenicity set; SARS-CoV-2 = severe acute respiratory syndrome coronavirus 2; ULOQ = upper limit of quantification. Neutralizing antibody geometric mean concentrations at specified timepoints in all participants (A) and by SARS-CoV-2 pre-booster (negative and positive) status at baseline (B) in the PPIS. Error bars represent 95% confidence intervals. n, number of participants with non-missing data at the corresponding timepoint. Median interval from Day 1 to BD-1: PPIS, 425 days; pre-booster negative subgroup, 424 days; pre-booster positive subgroup, 447 days

Antibody: SARS-CoV-2 (D614G) Neutralizing Antibody (VAC62 assay) (LLOQ: 10, ULOQ: 281600, Log10(LLOQ): 1.00, Log10(ULOQ): 5.45)

Antibody values reported as below the LLOQ are replaced by 0.5 x LLOQ. Values greater than the ULOQ are replaced by the ULOQ if actual values are not available.

Figure S5. Distribution of Pseudovirus Neutralizing Antibody Against SARS-CoV-2 (D614G), Part C PPIS and by Pre-Booster SARS-CoV-2 Status

A. All Participants (PPIS)

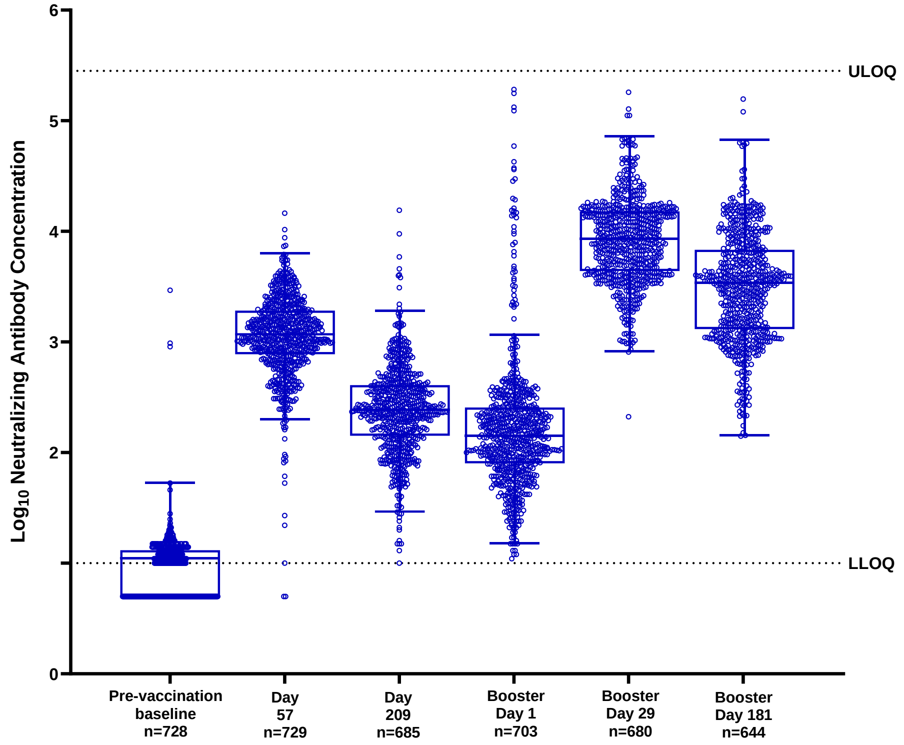

B. PPIS-Negative

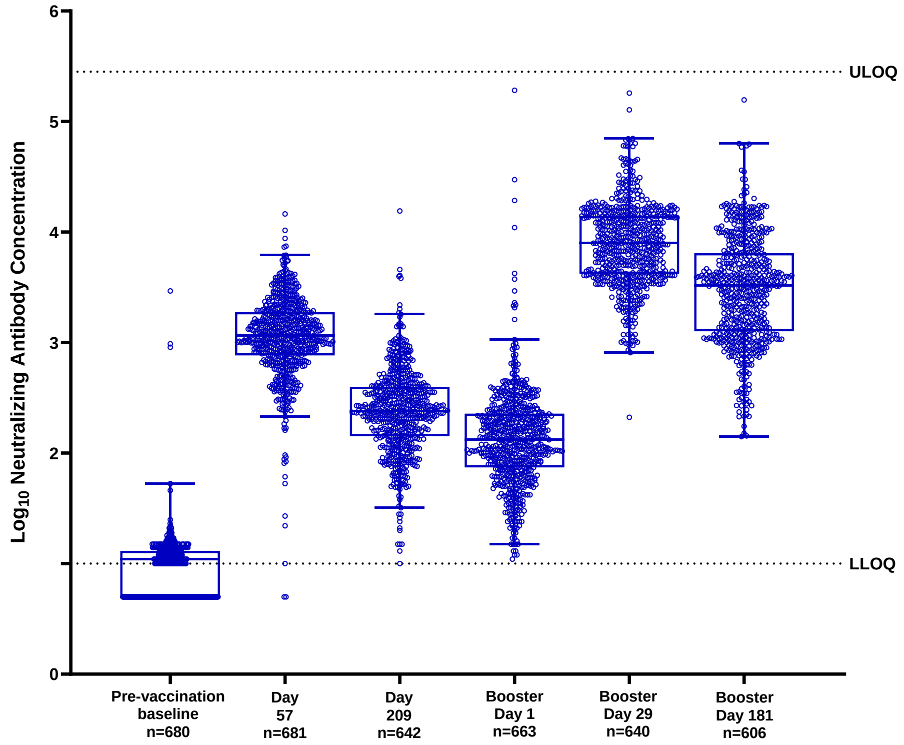

C. PPIS-positive

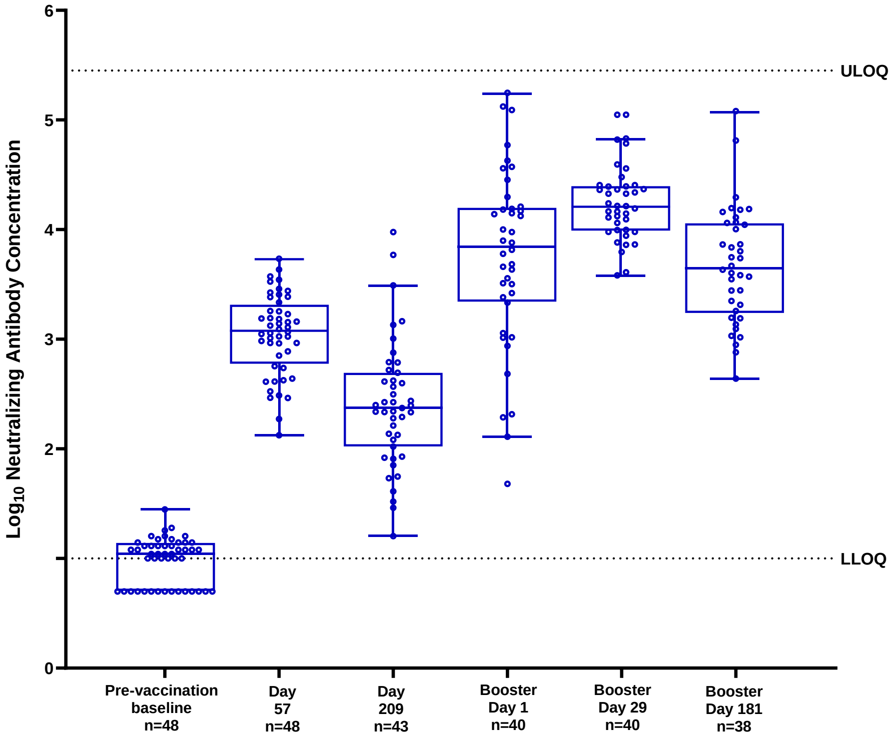

Figure S5. LLOQ = lower limit of quantification; LOG = logarithm; n = number of participants in the group with a result at the corresponding time point; PPIS = per-protocol immunogenicity set SARS-CoV-2 = severe acute respiratory syndrome 2; ULOQ = upper limit of quantification. Distribution of serum neutralizing antibody concentrations among all participants (A), and those pre-booster SARS-CoV-2-negative (B) and -positive (C) in the PPIS. The circles represent values from individual serum samples. Antibody: SARS-CoV-2 (D614G) Neutralizing Antibody (VAC62 assay) (LLOQ: 10, ULOQ: 281600, Log10(LLOQ): 1.00, Log10(ULOQ): 5.45)
Antibody values reported as below the LLOQ are replaced by 0.5 x LLOQ. Values greater than the ULOQ are replaced by the ULOQ if actual values are not available. Boxplot is based on log-transformed value. Boxes and horizontal bars denote interquartile ranges (IQR) and median endpoint titers; whisker endpoints are the maximum and minimum values below or above the median ±1.5 times the IQR.

Figure S6. Neutralizing Antibodies against Ancestral SARS-CoV-2 (D614G) After mRNA-1273 Primary Series and Booster, By Pre-Booster Infection Status and Age Group, PPIS

A. PPIS-Negative

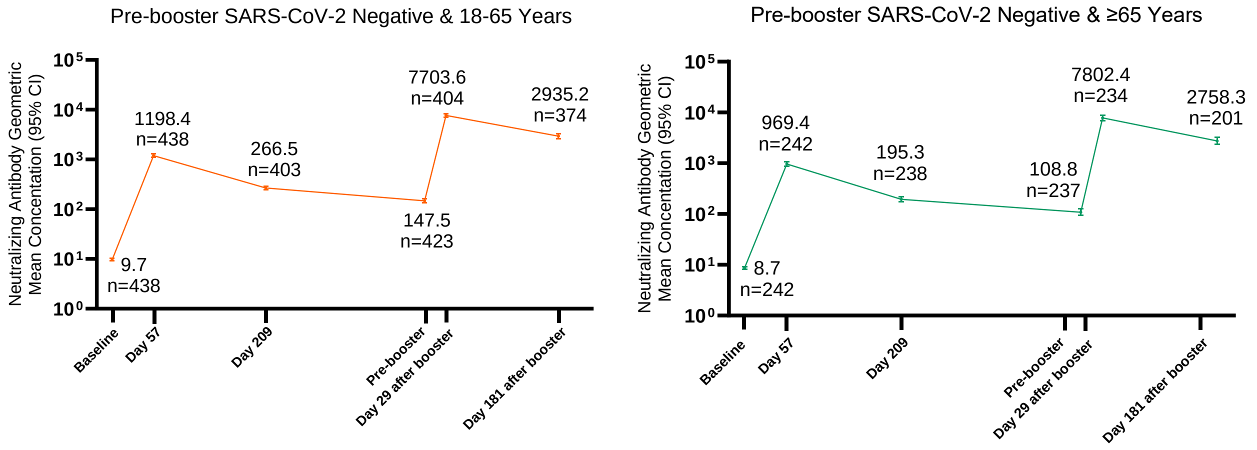

B. PPIS-Positive

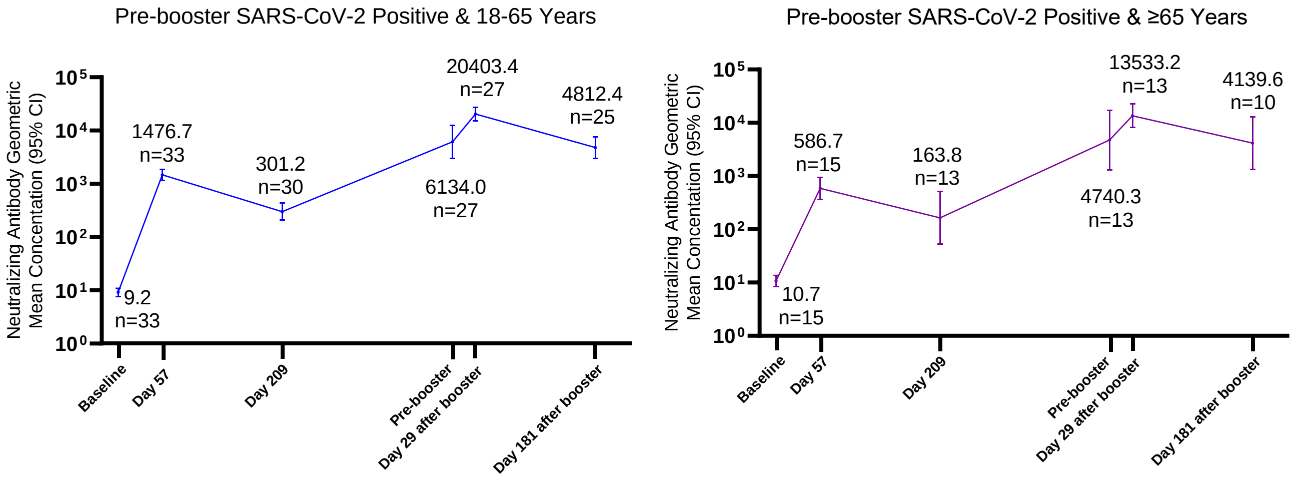

Figure S6. CI = confidence interval; LLOQ = lower limit of quantification; n = number of participants in the group with a result at the corresponding time point; PPIS = per-protocol immunogenicity set; SARS-CoV-2 = severe acute respiratory syndrome coronavirus 2; ULOQ = upper limit of quantification.
Neutralizing antibody geometric mean concentrations at specified timepoints following primary vaccination with 100-µg mRNA-1273 and a 50-µg booster dose in participants SARS-CoV-2 pre-booster-negative (A) and -positive (B) status at baseline in the PPIS. Error bars represent 95% confidence intervals. n, number of participants with non-missing data at the corresponding timepoint. Antibody: SARS-CoV-2 (D614G) Neutralizing Antibody (VAC62 assay) (LLOQ: 10, ULOQ: 281600, Log10(LLOQ): 1.00, Log10(ULOQ): 5.45)
Antibody values reported as below the LLOQ are replaced by 0.5 x LLOQ. Values greater than the ULOQ are replaced by the ULOQ if actual values are not available.

Figure S7. Distribution of Binding Antibody Against Ancestral SARS-CoV-2, Part C PPIS and by Pre-booster SARS-CoV-2 Status

A. All Participants (PPIS)

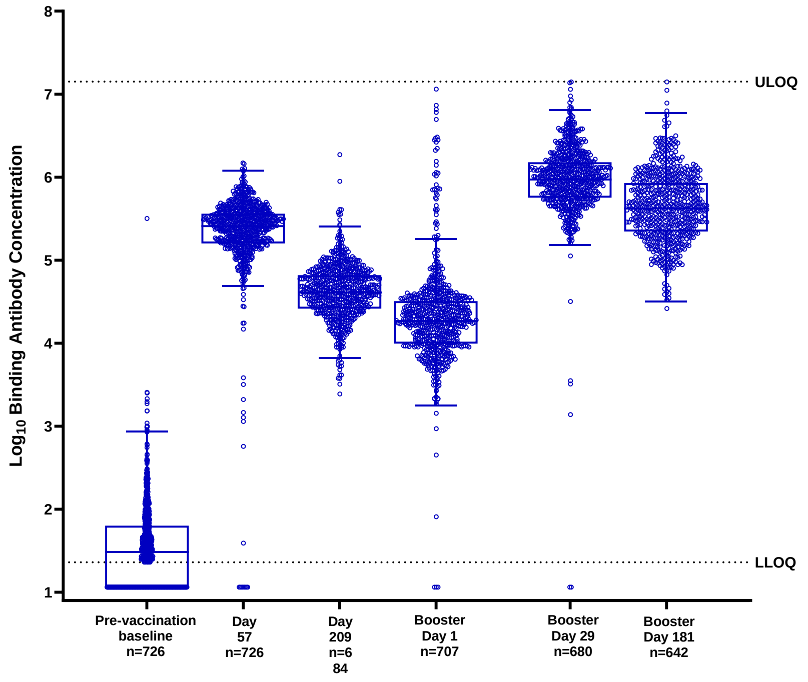

B. PPIS-Negative

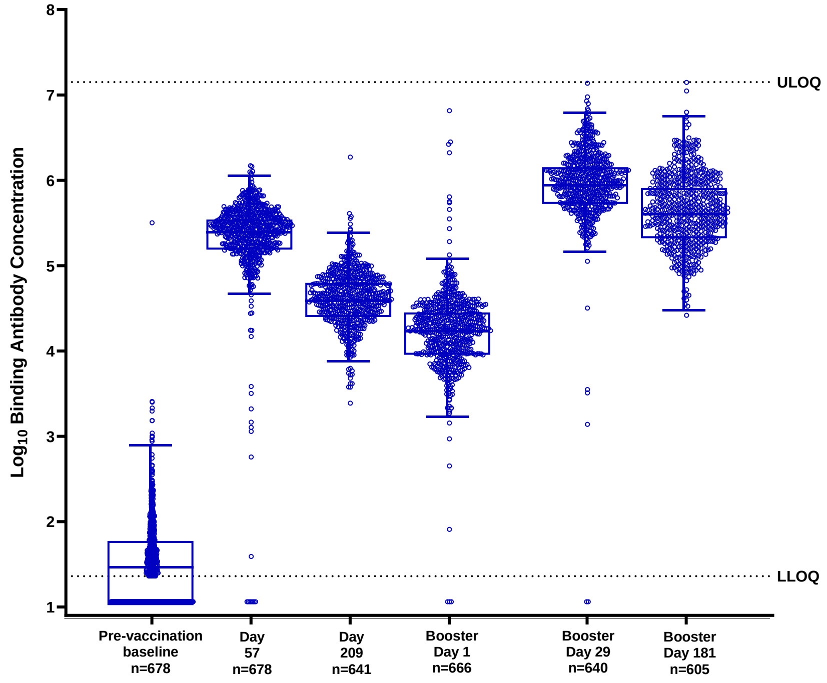

C. PPIS-Positive

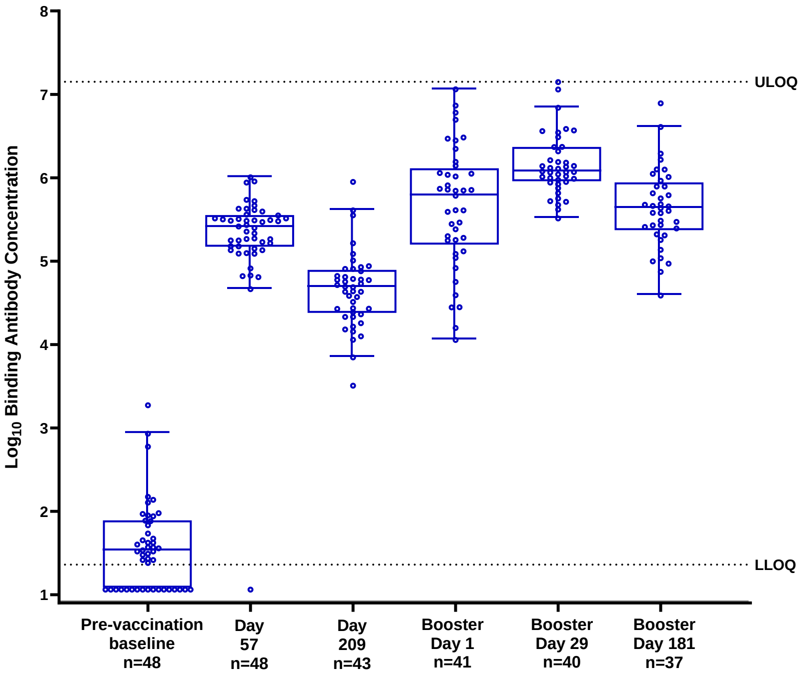

Figure S7. AU = absorbance units; LLOQ = lower limit of quantification; LOG = logarithm; MSD = mesoscale discovery; n = number of participants in the group with a result at the corresponding time point; PPIS = per-protocol immunogenicity set SARS-CoV-2 = severe acute respiratory syndrome 2; ULOQ = upper limit of quantification. Distribution of serum neutralizing antibody concentrations among all participants (A), and those pre-booster SARS-CoV-2-negative (B) and -positive (C) in the PPIS. The circles represent values from individual serum samples. Antibody: SARS-CoV-2S2P IgG Antibody (AU/mL) by MSD MULTIPLEX (VAC72) PPD Vaccine (LLOQ: 23, ULOQ: 14000000, Log10[LLOQ]: 1.36, Log10[ULOQ]: 7.15). Antibody values reported as below the LLOQ are replaced by 0.5 x LLOQ. Values greater than the ULOQ are replaced by the ULOQ if actual values are not available. Boxplot is based on log-transformed values. Boxes and horizontal bars denote interquartile ranges (IQR) and median endpoint titers; whisker endpoints are the maximum and minimum values below or above the median ±1.5 times the IQR.

Figure S8. Neutralizing Antibody Pre-Booster and Fold-rise (BD-1 to BD-29) Against Ancestral SARS-CoV-2 (D614G) Vs. Dosing Interval, mRNA-1273 Participants

A. Neutralizing Antibody Titer

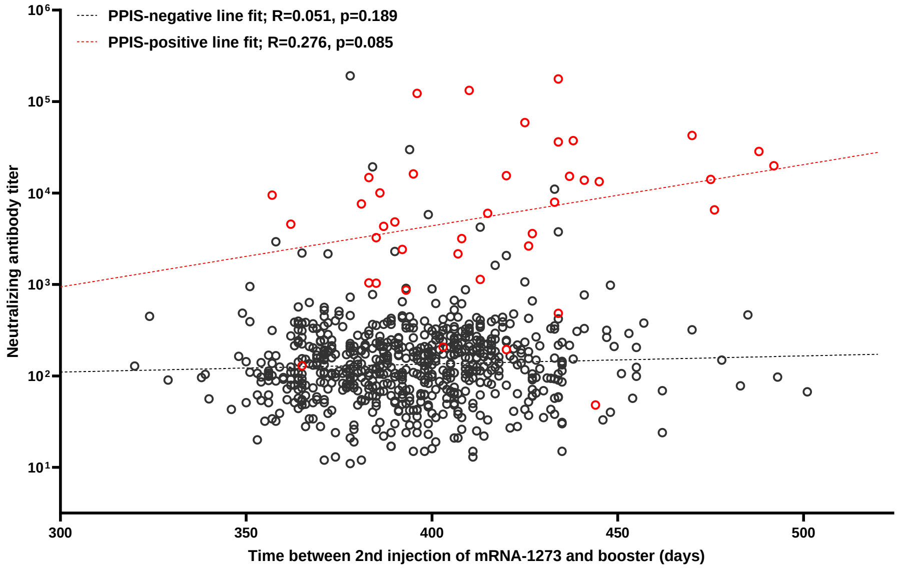

B. Neutralizing Antibody Titer Fold-rise BD-1 to BD-29 (Log-scale)

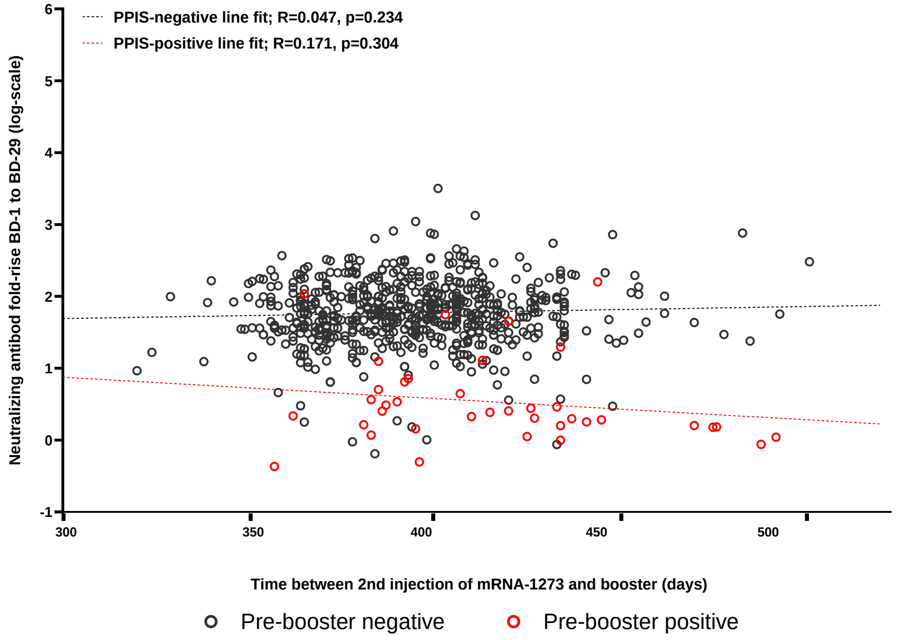

Figure S8. Neutralizing antibody (nAb) titers (A) and fold-rises in nAb from BD-1 to BD-29 (B) among per-protocol immunogencity set (PPIS) pre-booster -negative (n=682) and -postive (n=49) participants across time intervals (days) between 2nd injection of the primary series and booster doses.

Figure S9. Neutralizing Antibody Against Ancestral SARS-CoV-2 (D614G) Strain Pre-Booster Vs. Dosing Interval, PPIS-Negative mRNA-1273 and Placebo-mRNA-1273 Subset

A. Neutralizing Antibody Titer BD-1 **B. Neutralizing Antibody Titer BD-29**

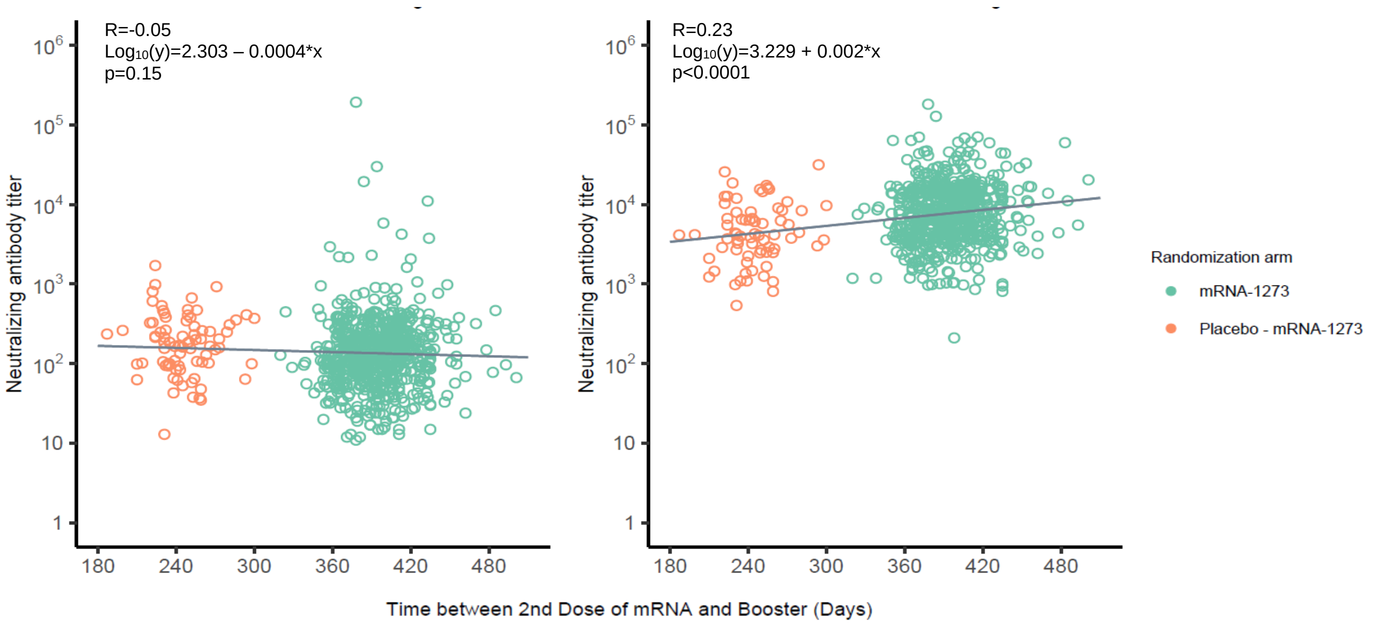

C. Time Intervals in Months

	Interval in months (time between second injection of mRNA-1273 and booster)									
	BD DAY 1					BD DAY 29				
	N	MIN	MAX	MEAN	MEDIAN	N	MIN	MAX	MEAN	MEDIAN
mRNA-1273 100-µG	663	10.5	16.5	13.0	12.9	639	10.5	16.5	13.0	12.9
Placebo-mRNA-1273	80	6.1	9.9	8.1	8.0	71	6.1	9.9	8.0	8.0

Figure S9. Neutralizing antibody (nAb) titers at BD-1 (A) and BD-29 (B) in mRNA-1273 and placebo - mRNA-1273 groups who were pre-booster SARS-CoV-2-negative across time intervals (days) between 2nd injections of the primary series and booster doses as summarized in C.

Figure S10. Design of the COVE Exploratory Booster Analysis

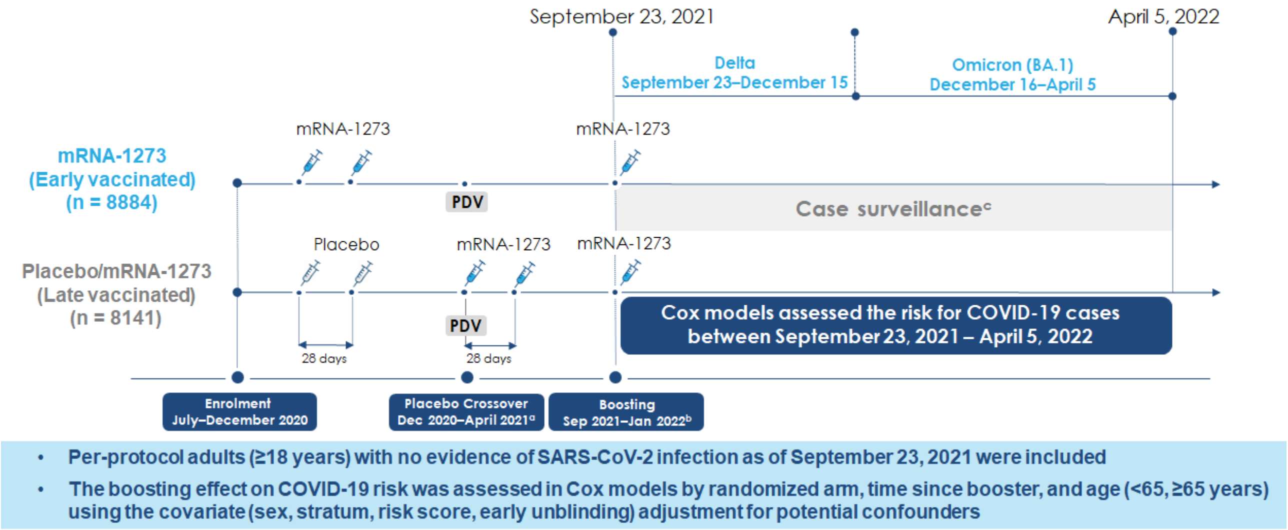

Figure S10. COVID-19 = coronavirus 2019; PDV = participant decision visit; RT-PCR = reverse transcription polymerase chain reaction; SARS-CoV-2 = severe acute respiratory syndrome coronavirus 2. Exploratory analysis of booster effectiveness in 20,404 participants (17,025 boosted; 3,379 unboosted) in the PP-primary set who remained on study and were COVID-19-naïve as of the first booster date (September 23rd, 2021). COVID-19 risk was assessed September 23rd, 2021 to April 5, 2022. Unboosted participants included those who remained unboosted through January 31, 2022, the censor date for the analysis.

[a]Participants in the placebo arm from a blinded phase of a randomized clinical trial (COVE) were unblinded and offered vaccination.

[b]Participants who remained unboosted through January 31, 2022, were censored on that date for the analysis.

[c]COVID-19 cases were based on RT-PCR confirmed with eligible symptoms.

Figure S11. Cumulative Proportion of Boosted Participants

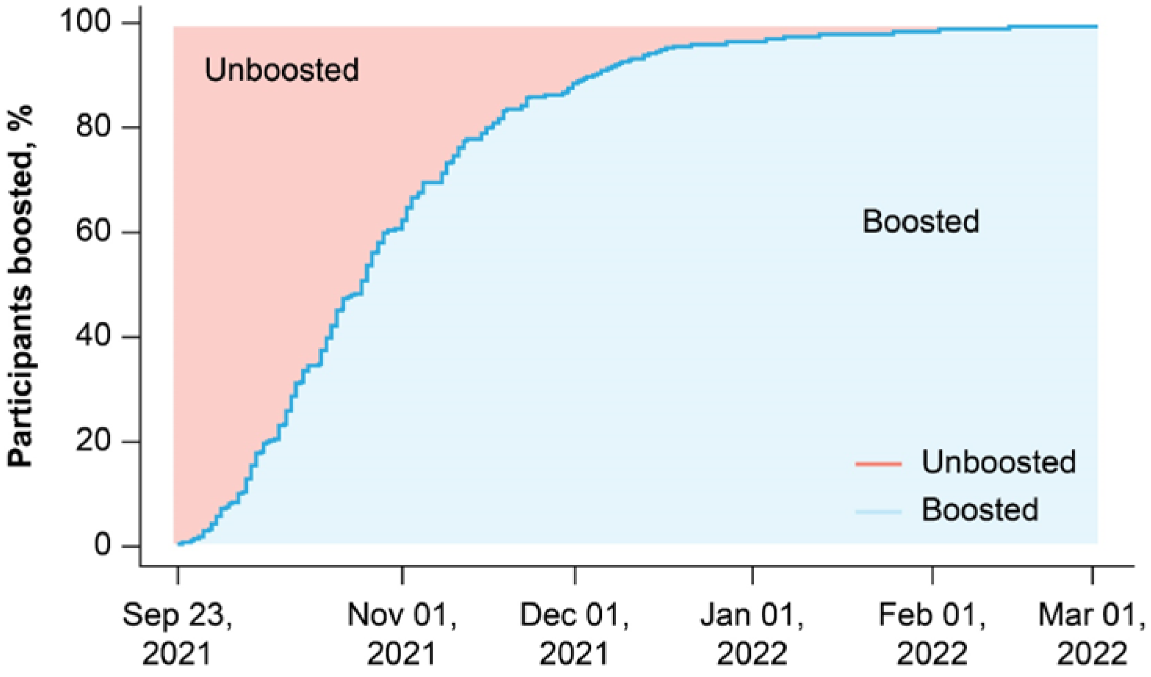

Figure S11. Cumulative proportion of boosted and unboosted participants in the exploratory analysis of booster effectiveness in 20,404 participants in the PP-primary set. 60% of participants received a booster by October 31, 2021; 88% of participants received a booster by November 30, 2021; 97% of participants received a booster by December 31, 2021.Previously presented.[6]

Figure S12. Exploratory Analysis of Booster Effectiveness Against Delta and Omicron (BA.1) Over Time

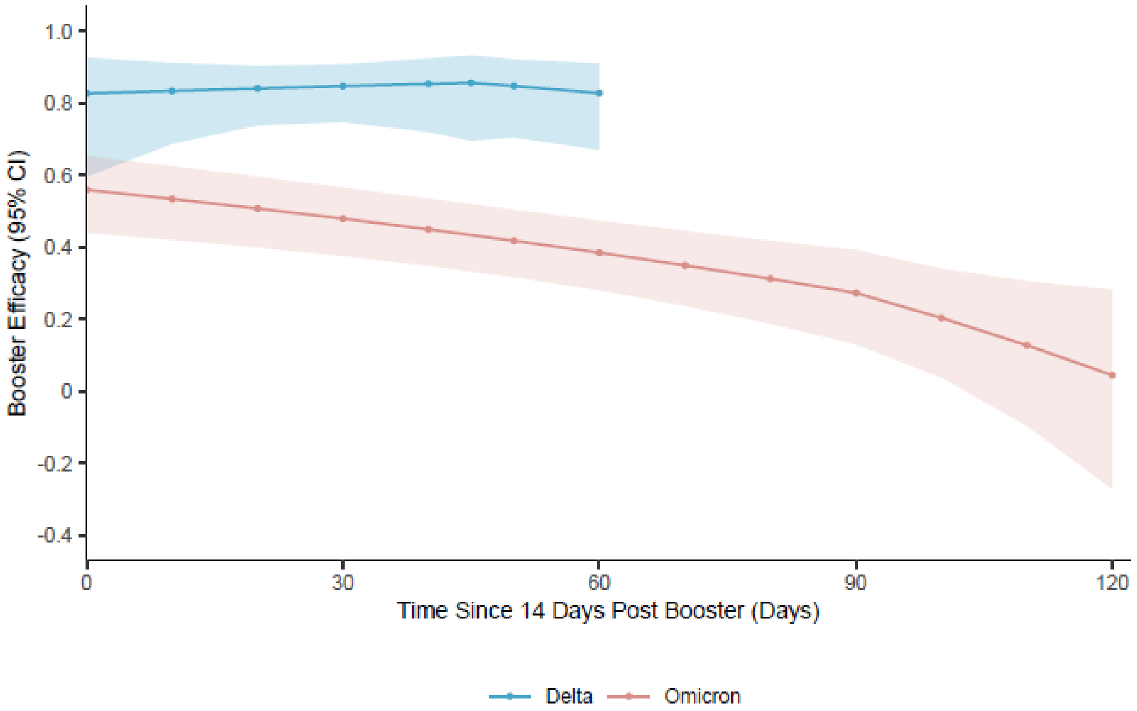

Figure S12. Relative efficacy of booster effectiveness over time following a 50-µg mRNA-1273 booster dose against Delta and Omicron (BA.1) variants. Solid lines represent point estimates of booster efficacy for Delta and Omicron (BA.1); shaded areas represent corresponding 95% confidence intervals. Booster efficacy was calculated as 1 – post-boost/pre-boost hazard ratios. Previously presented.[6]

Figure S13. Exploratory Analysis of Booster Effectiveness Against Omicron (BA.1) Over Time by Age Groups

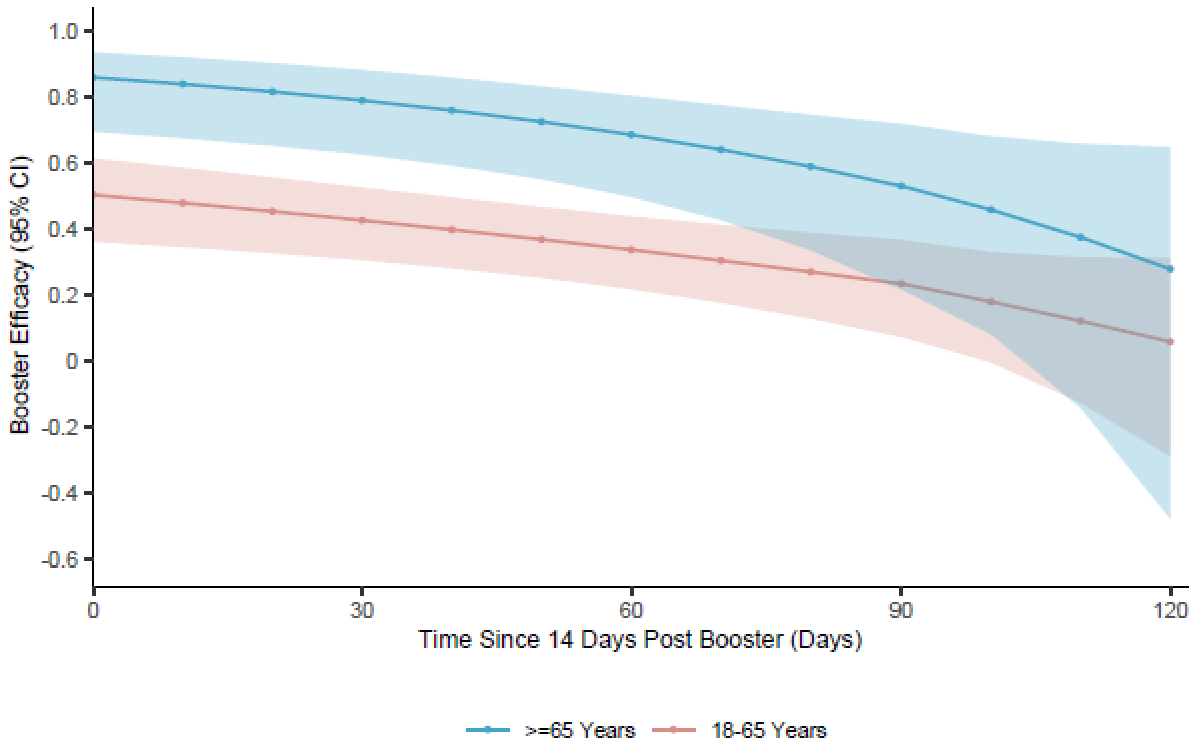

Figure S13. Relative efficacy of booster effectiveness over time following a 50-µg mRNA-1273 booster dose during the Omicron (BA.1) variant wave in participants <65 (n=12,111) and ≥65 (n=4,914) years of age. Solid lines represent point estimates of booster efficacy for Omicron (BA.1) in <65 and ≥65 year age groups; shaded areas represent corresponding 95% confidence intervals. Booster efficacy was calculated as 1 – post-boost/pre-boost hazard ratios. Previously presented.[6]

Figure S14. COVE Study Design and Modifications

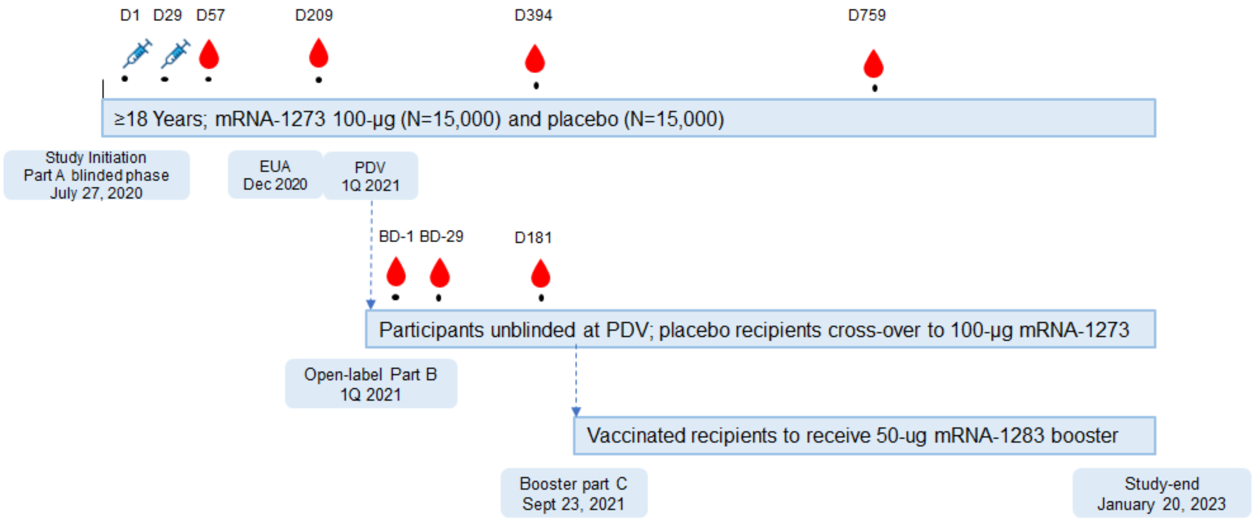

Figure S14. 1Q =1st quarter; BD-1 and BD-29 = booster dose day 1 and day 29; COVE = Coronavirus efficacy; D = day; EUA = Emergency Use Authorization; PDV = participant decision visit (all participants offered unblinding and mRNA-1273 vaccination if randomized to placebo); = vaccination, = blood sample for COVID-19 surveillance and/or immunogenicity analysis. The COVE trial, initiated on July 27, 2020, was designed to evaluate the safety and efficacy of 2-injections of 100-µg mRNA-1273 versus placebo against SARS-CoV-2 infection in adults (≥18 years). Participants in Part A, the randomized, placebo-controlled, blinded phase of this study, were blinded to mRNA-1273 100-µg or placebo. Following EUA of COVID-19 vaccines (Dec 2020), the protocol was amended to add Part B, the Open-Label Observational Phase, to offer all participants the option to unblind to their original treatment assignment and for those who received placebo in Part A of this study and who met EUA eligibility, an option to request open-label 100-µg mRNA-1273 during a PDV. Following EUA for administration of single booster doses of original COVID-19 vaccines to adults who had completed primary series vaccination, participants enrolled in Part B who had received at least one injection of 100-µg mRNA-1273 in the study were eligible to proceed to Part C, the booster Phase of the study to receive a 50-µg mRNA-1273 booster dose.

Table S1. Baseline Demographics Prior to Primary Vaccination, Part C Safety Set

	mRNA-1273 100 µg Primary Series (N=9647)	Placebo-mRNA-1273 100 µg Primary Series (N=9952)	Total (N=19609)
Age at screening (years)			
Mean (range)	52.4 (18-95)	52.9 (18-95)	52.6 (18-95)
Age group at screening, n (%)			
≥18 and <65 years	7027 (72.8)	7176 (72.1)	14212 (72.5)
Mean (range)	45.7 (18-64)	46.0 (18-64)	45.8 (18-64)
≥65 years	2620 (27.2)	2776 (27.9)	5397 (27.5)
Mean (range)	70.5 (65-95)	70.7 (65-95)	70.6 (65-95)
Age subgroup at screening, n (%)			
≥18 and <65 years	7027 (72.8)	7176 (72.1)	14212 (72.5)
≥65 and <75 years	2156 (22.3)	2223 (22.3)	4380 (22.3)
≥75 and <85 years	440 (4.6)	521 (5.2)	961 (4.9)
≥85 years	24 (0.2)	32 (0.3)	56 (0.3)
Age and health risk for severe COVID-19, n (%)[ab]			
≥18 and <65 years and not at risk	5375 (55.7)	5419 (54.5)	10797 (55.1)
≥18 and <65 years and at risk	1656 (17.2)	1759 (17.7)	3421 (17.4)
≥65 years	2616 (27.1)	2774 (27.9)	5391 (27.5)
Sex, n (%)			
Male	5020 (52.0)	5241 (52.7)	10268 (52.4)
Female	4627 (48.0)	4711 (47.3)	9341 (47.6)
Risk factor for severe COVID-19 at screening, n (%)[c]			
Chronic lung disease	489 (5.1)	530 (5.3)	1019 (5.2)
Significant cardiac disease	513 (5.3)	533 (5.4)	1046 (5.3)
Severe obesity	703 (7.3)	758 (7.6)	1463 (7.5)
Diabetes	999 (10.4)	1026 (10.3)	2028 (10.3)
Liver disease	73 (0.8)	68 (0.7)	141 (0.7)
Human immunodeficiency virus infection	71 (0.7)	66 (0.7)	138 (0.7)
At risk for severe COVID-19 at screening, n (%)			
Yes	2321 (24.1)	2438 (24.5)	4764 (24.3)
One risk factor for severe COVID-19	1865 (19.3)	1962 (19.7)	3831 (19.5)
Two or more risk factors for severe COVID-19	456 (4.7)	476 (4.8)	933 (4.8)
No	7326 (75.9)	7514 (75.5)	14845 (75.7)
Race, n (%)			
White	7545 (78.2)	7929 (79.7)	15481 (78.9)
Black or African American	1048 (10.9)	1031 (10.4)	2082 (10.6)
Asian	406 (4.2)	406 (4.1)	812 (4.1)
American Indian or Alaska native	74 (0.8)	78 (0.8)	152 (0.8)
Native Hawaiian or other Pacific islander	27 (0.3)	13 (0.1)	40 (0.2)
Multiracial	218 (2.3)	221 (2.2)	439 (2.2)
Other	212 (2.2)	183 (1.8)	395 (2.0)
Not reported	72 (0.7)	50 (0.5)	122 (0.6)
Unknown	45 (0.5)	41 (0.4)	86 (0.4)
Ethnicity, n (%)			
Hispanic or Latino	2002 (20.8)	1948 (19.6)	3955 (20.2)
Not Hispanic or Latino	7553 (78.3)	7913 (79.5)	15471 (78.9)
Not reported	70 (0.7)	54 (0.5)	124 (0.6)
Unknown	22 (0.2)	37 (0.4)	59 (0.3)
Race and ethnicity group, n (%)[f]			
White	5973 (61.9)	6343 (63.7)	12318 (62.8)
Communities of color	3655 (37.9)	3592 (36.1)	7255 (37.0)
Missing	19 (0.2)	17 (0.2)	36 (0.2)
Time Between second injection Primary Series to Booster			
Median days (IQR)	392.0 (373.0-413.0)	250.0 (236.0-266.0)	316.0 (249.0-392.0)
>35 and ≤44 weeks since Primary Series	91 (0.9)	5236 (52.6)	5327 (27.2)
>44 and ≤53 weeks since Primary Series	2134 (22.1)	358 (3.6)	2492 (12.7)
>53 weeks since Primary Series	7404 (76.7)	33 (0.3)	7437 (37.9)
Follow-Up Time on Study from Booster			
Median days (IQR)	333.0 (309.0-357.0)	325.0 (299.0-351.0)	330.00 (303.0-353.0)
Time on Study from 1st-Injection of mRNA-1273 (Days)			
Median days (IQR)	757.0 (749.0-765.0)	609.0 (586.0-629.0)	655.0 (604.0-757.0)
Baseline RT-PCR results, n (%)			
Negative	9535 (98.8)	9847 (98.9)	19392 (98.9)
Positive	62 (0.6)	52 (0.5)	114 (0.6)
Missing	50 (0.5)	53 (0.5)	103 (0.5)

Baseline Elecsys anti-SARS-CoV-2 results, n (%)			
Negative	9462 (98.1)	9777 (98.2)	19248 (98.2)
Positive	172 (1.8)	168 (1.7)	341 (1.7)
Missing	13 (0.1)	7 (<0.1)	20 (0.1)
Baseline SARS-CoV-2 status, n (%)[d]			
Negative	9397 (97.4)	9712 (97.6)	19118 (97.5)
Positive	198 (2.1)	184 (1.8)	383 (2.0)
By both RT-PCR and Elecsys	36 (0.4)	36 (0.4)	72 (0.4)
By RT-PCR only	26 (0.3)	16 (0.2)	42 (0.2)
By Elecsys only	136 (1.4)	132 (1.3)	269 (1.4)
Missing	52 (0.5)	56 (0.6)	108 (0.6)
PDV/unblinding RT-PCR results, n (%)			
Negative	8382 (86.9)	9388 (94.3)	17776 (90.7)
Positive	134 (1.4)	507 (5.1)	642 (3.3)
Missing	1131 (11.7)	57 (0.6)	1191 (6.1)
PDV/unblinding Elecsys anti-SARS-CoV-2 results, n (%)			
Negative	8304 (86.1)	8803 (88.5)	17114 (87.3)
Positive	230 (2.4)	1077 (10.8)	1308 (6.7)
Missing	1113 (11.5)	72 (0.7)	1187 (6.1)
PDV/unblinding SARS-CoV-2 status, n (%)			
Negative	8102 (84.0)	8625 (86.7)	16733 (85.3)
Positive	343 (3.6)	1225 (12.3)	1569 (8.0)
By both RT-PCR and Elecsys	21 (0.2)	359 (3.6)	381 (1.9)
By RT-PCR only	113 (1.2)	148 (1.5)	261 (1.3)
By Elecsys only	209 (2.2)	718 (7.2)	927 (4.7)
Missing	1202 (12.5)	102 (1.0)	1307 (6.7)
Pre-booster RT-PCR results, n (%) [e]			
Negative	5806 (60.2)	5887 (59.2)	11701 (59.7)
Positive	54 (0.6)	58 (0.6)	112 (0.6)
Missing	3787 (39.3)	4007 (40.3)	7796 (39.8)
Pre-booster Elecsys anti-SARS-CoV-2 results, n (%)			
Negative	8658 (89.7)	8278 (83.2)	16943 (86.4)
Positive	918 (9.5)	1614 (16.2)	2535 (12.9)
Missing	71 (0.7)	60 (0.6)	131 (0.7)
Pre-booster SARS-CoV-2 status, n (%)			
Negative	8705 (90.2)	8305 (83.5)	17017 (86.8)
Positive	941 (9.8)	1647 (16.5)	2591 (13.2)
By both RT-PCR and Elecsys	33 (0.3)	32 (0.3)	65 (0.3)
By RT-PCR only	21 (0.2)	26 (0.3)	47 (0.2)
By Elecsys only	887 (9.2)	1589 (16.0)	2479 (12.6)
Missing	1 (<0.1)	0	1 (<0.1)
Occupational risk, n (%)	7734 (80.2)	7992 (80.3)	15734 (80.2)
Healthcare workers	2258 (23.4)	1895 (19.0)	4154 (21.2)
Others	5476 (56.8)	6097 (61.3)	11580 (59.1)
Location and living circumstances risk, n (%) [b]	8181 (84.8)	8301 (83.4)	16492 (84.1)
Nursing home or assisted living facility	15 (0.2)	24 (0.2)	39 (0.2)
Multi-family dwelling	285 (3.0)	274 (2.8)	561 (2.9)
High density housing	795 (8.2)	783 (7.9)	1580 (8.1)
Low density, multi-family setting	957 (9.9)	999 (10.0)	1958 (10.0)
Single family home	5597 (58.0)	5580 (56.1)	11181 (57.0)
Other	1135 (11.8)	1345 (13.5)	2480 (12.6)
Body Mass Index, n, (weight in Kg/(height in meters^2)	9576	9889	19475
Mean (Standard Deviation)	29.42 (6.87)	29.55 (6.78)	29.49 (6.83)

IQR = interquartile range; IRT = interactive response technology; RT-PCR = reverse transcription polymerase chain reaction; PDV = participant decision visit. Percentages are based on the number of Part C safety participants. Total participants included 10 who received a booster without a primary injection and are not included in the mRNA-1273 and placebo-mRNA-1273 groups. Baseline characteristics were captured at enrollment in part A, prior to primary vaccination.
[a]Based on stratification factor from IRT, participants who are <65 years old are categorized as at risk for severe COVID-19 illness if they have at least 1 of the risk factors specified in the study protocol at Screening.
[b]Age and health risk for severe COVID-19 are derived from age and risk factors collected on case report form (CRF).
[c]Participants could be under ≥1 categories and are counted once at each category.
[d]Baseline SARS-CoV-2 status: Positive if there is immunologic or virologic evidence of prior COVID-19, defined as positive RT-PCR test or positive Elecsys result at day 1. Negative is defined as negative RT-PCR test and negative Elecsys result at day 1.
[e]RT-PCR test at booster dose day 1 is optional per-protocol.
[f]White is defined as White and non-Hispanic, and Communities of Color includes all the others whose race or ethnicity is not unknown, unreported or missing.

Table S2. Summary of Unsolicited Adverse Events after 1st Injection in the Primary Series Safety Set

n (%)	Primary Series Vaccination mRNA-1273 100 µg (N=15184)	Placebo-mRNA-1273 100 µg (N=12649)	Total (N=27833)
**Unsolicited AEs regardless of relationship to study vaccination**			
All	9663 (63.6)	6531 (51.6)	16194 (58.2)
Serious	764 (5.0)	542 (4.3)	1306 (4.7)
Fatal	53 (0.3)	24 (0.2)	77 (0.3)
Medically-attended	7717 (50.8)	5554 (43.9)	13271 (47.7)
Leading to discontinuation of study vaccine	81 (0.5)	12 (<0.1)	93 (0.3)
Leading to study discontinuation	64 (0.4)	31 (0.2)	95 (0.3)
Grade 3 or higher	1218 (8.0)	760 (6.0)	1978 (7.1)
Non-serious*	8899 (58.6)	5989 (47.3)	14888 (53.5)
Grade 3 or higher	588 (3.9)	331 (2.6)	919 (3.3)
Non-serious†	9569 (63.0)	6420 (50.8)	15989 (57.4)
Grade 3 or higher	742 (4.9)	430 (3.4)	1172 (4.2)
**Unsolicited TAEs related to study vaccination**			
All	2503 (16.5)	999 (7.9)	3502 (12.6)
Serious	12 (<0.1)	7 (<0.1)	19 (<0.1)
Fatal	0	0	0
Medically-attended	254 (1.7)	120 (0.9)	374 (1.3)
Leading to discontinuation of study vaccine	22 (0.1)	4 (<0.1)	26 (<0.1)
Leading to study discontinuation	1 (<0.1)	0	1 (<0.1)
Grade 3 or higher	99 (0.7)	32 (0.3)	131 (0.5)
Non-serious*	2491 (16.4)	992 (7.8)	3483 (12.5)
Grade 3 or higher	92 (0.6)	29 (0.2)	121 (0.4)
Non-serious†	2500 (16.5)	996 (7.9)	3496 (12.6)
Grade 3 or higher	93 (0.6)	29 (0.2)	122 (0.4)

Adverse event (AE) is defined as any event not present before exposure to study vaccination or any event already present that worsens in intensity or frequency after exposure. Percentages are based on the number of participants in the primary series safety set.
*Participants without any serious AEs and with any non-serious AE.
†Participants with at least one non-serious AE regardless of reporting any serious AE or not.

Table S3. Unsolicited Adverse Events Reported by ≥1% of Participants in Any Treatment Group, Primary Series Safety Set

System Organ Class Preferred Term n (%)	Primary Vaccination Series			
	mRNA-1273 100 µg (N=15184)		Placebo-mRNA-1273 100 µg (N=12649)	
	Any	Severe	Any	Severe
Number Reporting Unsolicited AEs	2503 (16.5)	97 (0.6)	999 (7.9)	32 (0.3)
Number of Unsolicited AEs	4731	121	2490	43
Blood and lymphatic system disorders	219 (1.4)	1 (<0.1)	29 (0.2)	1 (<0.1)
Lymphadenopathy	209 (1.4)	1 (<0.1)	25 (0.2)	0
Thrombocytopenia	0	0	1 (<0.1)	0
Nervous system disorders	695 (4.6)	18 (0.1)	264 (2.1)	6 (<0.1)
Headache	601 (4.0)	12 (<0.1)	230 (1.8)	5 (<0.1)
Gastrointestinal disorders	179 (1.2)	3 (<0.1)	84 (0.7)	3 (<0.1)
Nausea	104 (0.7)	2 (<0.1)	59 (0.5)	2 (<0.1)
Musculoskeletal and connective tissue disorders	602 (4.0)	14 (<0.1)	164 (1.3)	5 (<0.1)
Myalgia	382 (2.5)	8 (<0.1)	86 (0.7)	2 (<0.1)
Arthralgia	358 (2.4)	3 (<0.1)	35 (0.3)	0
General disorders and administration site conditions	1569 (10.3)	50 (0.3)	784 (6.2)	14 (0.1)
Fatigue	771 (5.1)	21 (0.1)	224 (1.8)	4 (<0.1)
Injection site pain	318 (2.1)	5 (<0.1)	537 (4.2)	3 (<0.1)
Pyrexia	73 (0.5)	0	193 (1.5)	2 (<0.1)
Chills	108 (0.7)	5 (<0.1)	154 (1.2)	1 (<0.1)
Injection site erythema	174 (1.1)	9 (<0.1)	51 (0.4)	3 (<0.1)
Pain	38 (0.3)	0	179 (1.4)	1 (<0.1)

Adverse event (AE) is defined as any event not present before exposure to study vaccination or any event already present that worsens in intensity or frequency after exposure. Percentages are based on the number of participants in the primary series safety set. MedDRA version 23.0.

Table S4. Serious Unsolicited Treatment-related Adverse Events of Participants in Any Treatment Group, Primary Series Safety Set

System Organ Class<br>Preferred Term<br>n (%)	Primary Vaccination Series			
	mRNA-1273<br>100 µg<br>(N=15184)		Placebo-mRNA-1273<br>100 µg<br>(N=12649)	
	Any	Severe	Any	Severe
Number Reporting Unsolicited AEs	12 (<0.1)	6 (<0.1)	7 (<0.1)	3 (<0.1)
Number of Unsolicited AEs	15	7	7	3
Neoplasms benign, malignant and unspecified (incl cysts and polyps)	1 (<0.1)	1 (<0.1)	0	0
B-cell small lymphocytic lymphoma	1 (<0.1)	1 (<0.1)	0	0
Blood and lymphatic system disorders	0	0	1 (<0.1)	1 (<0.1)
Anaemia	0	0	1 (<0.1)	1 (<0.1)
Endocrine disorders	1 (<0.1)	1 (<0.1)	1 (<0.1)	0
Autoimmune thyroiditis	0	0	1 (<0.1)	0
Basedow's disease	1 (<0.1)	1 (<0.1)	0	0
Nervous system disorders	3 (<0.1)	1 (<0.1)	1 (<0.1)	1 (<0.1)
Autonomic nervous system imbalance	1 (<0.1)	0	0	0
Cerebrovascular accident	1 (<0.1)	1 (<0.1)	0	0
Multiple sclerosis	1 (<0.1)	0	0	0
Paraesthesia	0	0	1 (<0.1)	1 (<0.1)
Ear and labyrinth disorders	1 (<0.1)	1 (<0.1)	0	0
Deafness unilateral	1 (<0.1)	1 (<0.1)	0	0
Cardiac disorders	1 (<0.1)	1 (<0.1)	1 (<0.1)	0
Pericardial effusion	1 (<0.1)	1 (<0.1)	1 (<0.1)	0
Pericarditis	1 (<0.1)	0	0	0
Respiratory, thoracic and mediastinal disorders	1 (<0.1)	1 (<0.1)	0	0
Pleural effusion	1 (<0.1)	1 (<0.1)	0	0
Gastrointestinal disorders	1 (<0.1)	0	0	0
Nausea	1 (<0.1)	0	0	0
Vomiting	1 (<0.1)	0	0	0
Skin and subcutaneous tissue disorders	1 (<0.1)	0	0	0
Angioedema	1 (<0.1)	0	0	0
Musculoskeletal and connective tissue disorders	1 (<0.1)	0	2 (<0.1)	1 (<0.1)
Connective tissue disorder	0	0	1 (<0.1)	0
Muscular weakness	0	0	1 (<0.1)	1 (<0.1)
Rheumatoid arthritis	1 (<0.1)	0	0	0
Pregnancy, puerperium and perinatal conditions	0	0	1 (<0.1)	0
Abortion spontaneous	0	0	1 (<0.1)	0
General disorders and administration site conditions	2 (<0.1)	1 (<0.1)	0	0
Swelling face	2 (<0.1)	1 (<0.1)	0	0

Adverse event (AE) is defined as any event not present before exposure to study vaccination or any event already present that worsens in intensity or frequency after exposure.
Percentages are based on the number of participants in the primary series safety set.
MedDRA version 23.0.

Table S5. Summary of Unsolicited Adverse Events ≤28 Days After Booster, Part C Safety Set

n (%)	Part C Booster		
	mRNA-1273 100 µg Primary Series (N=9647)	Placebo-mRNA-1273 100 µg Primary Series (N=9952)	Total (N=19609)
**Unsolicited AEs regardless of relationship to study vaccination**			
All	3303 (34.2)	3140 (31.6)	6443 (32.9)
Serious	42 (0.4)	58 (0.6)	100 (0.5)
Fatal	1 (<0.1)*	5 (<0.1)**	6 (<0.1)
Medically-attended	940 (9.7)	1004 (10.1)	1944 (9.9)
Leading to study discontinuation	1 (<0.1)	5 (<0.1)	6 (<0.1)
Grade 3 or higher	108 (1.1)	110 (1.1)	218 (1.1)
Non-serious[a]	3261 (33.8)	3082 (31.0)	6343 (32.3)
Grade 3 or higher	77 (0.8)	62 (0.6)	139 (0.7)
Non-serious[b]	3286 (34.1)	3114 (31.3)	6400 (32.6)
Grade 3 or higher	82 (0.9)	65 (0.7)	147 (0.7)
**Unsolicited AEs related to study vaccination**			
All	2424 (25.1)	2173 (21.8)	4597 (23.4)
Serious	3 (<0.1)	1 (<0.1)	4 (<0.1)
Fatal	0	0	0
Medically-attended	107 (1.1)	99 (1.0)	206 (1.1)
Leading to study discontinuation	0	0	0
Grade 3 or higher	37 (0.4)	28 (0.3)	65 (0.3)
Non-serious[a]	2421 (25.1)	2172 (21.8)	4593 (23.4)
Grade 3 or higher	35 (0.4)	28 (0.3)	63 (0.3)
Non-serious[b]	2422 (25.1)	2173 (21.8)	4595 (23.4)
Grade 3 or higher	35 (0.4)	28 (0.3)	63 (0.3)

An adverse event (AE) is defined as any event not present before exposure to study vaccination or any event already present that worsens in intensity or frequency after exposure.

Percentages are based on the number of Part C safety participants.

[a]Participants without any SAE and with any non-serious AE.

[b]Participants with at least one non-serious AE regardless of reporting any SAE or not.

*One fatal event due to a road traffic accident. **Fatal events include 2 cardiac arrest, 1 cerebrovascular accident (left-sided stroke M1 occlusion); Pancreatic carcinoma (pancreatic cancer), and ventricular extrasystoles (worsening of premature ventricular contractions).

Table S6. Treatment-related Unsolicited AEs ≤28 Days after Booster Reported by ≥1% of Participants in Any Treatment Group, Part C Safety Set

System Organ Class<br>Preferred Term<br>n (%)	Part C Booster			
	mRNA-1273 100 µg<br>Primary Series<br>(N=9647)		Placebo-mRNA-1273 100 µg<br>Primary Series<br>(N=9952)	
	Any	Severe	Any	Severe
Number Reporting Unsolicited AEs	2424 (25.1)	37 (0.4)	2173 (21.8)	28 (0.3)
Number of Unsolicited AEs	4755	51	4087	44
Nervous system disorders	516 (5.3)	6 (<0.1)	448 (4.5)	5 (<0.1)
Headache	486 (5.0)	6 (<0.1)	419 (4.2)	3 (<0.1)
Gastrointestinal disorders	97 (1.0)	1 (<0.1)	112 (1.1)	2 (<0.1)
Nausea	70 (0.7)	0	81 (0.8)	1 (<0.1)
Musculoskeletal and connective tissue disorders	425 (4.4)	6 (<0.1)	375 (3.8)	12 (0.1)
Myalgia	279 (2.9)	5 (<0.1)	215 (2.2)	7 (<0.1)
General disorders and administration site conditions	2135 (22.1)	21 (0.2)	1930 (19.4)	15 (0.2)
Injection site pain	1330 (13.8)	4 (<0.1)	1247 (12.5)	2 (<0.1)
Fatigue	694 (7.2)	7 (<0.1)	630 (6.3)	7 (<0.1)
Pain	362 (3.8)	2 (<0.1)	277 (2.8)	2 (<0.1)
Chills	327 (3.4)	5 (<0.1)	242 (2.4)	4 (<0.1)
Pyrexia	304 (3.2)	3 (<0.1)	188 (1.9)	1 (<0.1)

Adverse event (AE) is defined as any event not present before exposure to study vaccination or any event already present that worsens in intensity or frequency after exposure. Percentages are based on the number of participants in the Part C safety set.
MedDRA version 23.0.

Table S7. Serious Treatment-related Unsolicited Adverse Events ≤28 Days after Booster, Part C Safety Set

**System Organ Class<br>Preferred Term<br>n (%)**	**Part C Booster**			
	**mRNA-1273 100 µg<br>Primary Series<br>(N=9647)**		**Placebo-mRNA-1273 100 µg<br>Primary Series<br>(N=9952)**	
	**Any**	**Severe**	**Any**	**Severe**
Number reporting unsolicited AEs	3 (<0.1)	2 (<0.1)	1 (<0.1)	0
Number of unsolicited AEs	3	2	1	0
Cardiac disorders	3 (<0.1)	2 (<0.1)	0	0
Arteriospasm coronary	1 (<0.1)	1 (<0.1)	0	0
Cardiac flutter	1 (<0.1)	0	0	0
Myocarditis	1 (<0.1)	1 (<0.1)	0	0
Skin and subcutaneous tissue disorders	0	0	1 (<0.1)	0
Erythema nodosum	0	0	1 (<0.1)	0

Adverse event (AE) is defined as any event not present before exposure to study vaccination or any event already present that worsens in intensity or frequency after exposure. Percentages are based on the number of participants in Part C safety set. One additional SAE of myocarditis considered unrelated to study vaccine ccurred in the Placebo-mRNA-1273 group through the study data cut-off date (Table S12). MedDRA version 23.0. Three SAEs were reported in participants in the mRNA-1273 group. These included one case of myocarditis in a participant >40 years with onset day 1 post-booster the mRNA-1273 group that was considered related to vaccination by the investigator and sponsor. The participant presented with a Rhinovirus/Enterovirus upper respiratory infection one month prior to the onset of symptoms that resolved 3 days prior to the booster dose following treatment with prednisone and albuterol. The participant had elevated levels of troponin and C-reactive protein and was hospitalized to investigate potential MI, treated with intravenous heparin, oral aspirin, transdermal nitroglycerin, and metoprolol then discharged after 3 days with follow-up cardiology. The myocarditis resolved on BD-72 and cardiac MRI performed 6 weeks after the event showed no evidence of myocarditis. The other, a case of severe coronary arteriospasm with onset on Day 11 occurred in a participant >70 years with a history of coronary artery disease, deemed related to vaccination by the investigator that was treated with concomitant medication and resolved on BD-82. A cardiac disorder reported as cardiac flutter “heart fluttering” not verified by EKG occurred on Day 12 in a participant >40 years in the mRNA-1273 group with a history of anxiety who was on treatment with escitalopram citrate that resolved on day 13 without treatment. A case of erythema nodosum was reported in a female >70 years on Day 9 in the placebo-mRNA-1273 group that resolved without treatment on day 30.

Table S8. Summary of Unsolicited Adverse Events ≤28 Days After Booster by Pre-booster SARS-CoV-2 Infection Status, Part C Safety Set

	SARS-CoV-2 Negative		SARS-CoV-2 Positive	
	mRNA-1273 100 µg Primary Series (N=8705)	Placebo-mRNA-1273 100 µg Primary Series (N=8305)	mRNA-1273 100 µg Primary Series (N=941)	Placebo-mRNA-1273 100 µg Primary Series (N=1647)
Unsolicited AEs Regardless of Relationship to Study Vaccination				
All	3032 (34.8)	2677 (32.2)	270 (28.7)	463 (28.1)
Serious	40 (0.5)	48 (0.6)	2 (0.2)	10 (0.6)
Fatal	1 (<0.1)	3 (<0.1)	0	2 (0.1)
Medically-attended	842 (9.7)	839 (10.1)	98 (10.4)	165 (10.0)
Leading to study discontinuation	1 (<0.1)	3 (<0.1)	0	2 (0.1)
Grade 3 or higher	101 (1.2)	90 (1.1)	7 (0.7)	20 (1.2)
Non-serious*	2992 (34.4)	2629 (31.7)	268 (28.5)	453 (27.5)
Grade 3 or higher	72 (0.8)	50 (0.6)	5 (0.5)	12 (0.7)
Non-serious†	3015 (34.6)	2656 (32.0)	270 (28.7)	458 (27.8)
Grade 3 or higher	76 (0.9)	53 (0.6)	6 (0.6)	12 (0.7)
Unsolicited TEAEs related to Study Vaccination				
All	2254 (25.9)	1868 (22.5)	169 (18.0)	305 (18.5)
Serious	3 (<0.1)	1 (<0.1)	0	0
Fatal	0	0	0	0
Medically-attended	98 (1.1)	83 (1.0)	9 (1.0)	16 (1.0)
Leading to study discontinuation	0	0	0	0
Grade 3 or higher	34 (0.4)	23 (0.3)	3 (0.3)	5 (0.3)
Non-serious*	2251 (25.9)	1867 (22.5)	169 (18.0)	305 (18.5)
Grade 3 or higher	32 (0.4)	23 (0.3)	3 (0.3)	5 (0.3)
Non-serious†	2252 (25.9)	1868 (22.5)	169 (18.0)	305 (18.5)
Grade 3 or higher	32 (0.4)	23 (0.3)	3 (0.3)	5 (0.3)

An adverse event (AE) is defined as any event not present before exposure to study vaccination or any event already present that worsens in intensity or frequency after exposure. Percentages are based on the number of Part C safety participants.
*Participants without any SAE and with any non-serious AE.
†Participants with at least one non-serious AE regardless of reporting any SAE or not.

Table S9. Summary of Unsolicited Adverse Events ≤28 Days After Booster by Sex Status, Part C Safety Set

	Male		Female	
	mRNA-1273 100 µg Primary Series (N=5020)	Placebo-mRNA-1273 100 µg Primary Series (N=5241)	mRNA-1273 100 µg Primary Series (N=4627)	Placebo-mRNA-1273 100 µg Primary Series (N=4711)
**Unsolicited AEs Regardless of Relationship to Study Vaccination**				
All	1630 (32.5)	1582 (30.2)	1673 (36.2)	1558 (33.1)
Serious	23 (0.5)	33 (0.6)	19 (0.4)	25 (0.5)
Fatal	0	5 (<0.1)	1 (<0.1)	0
Medically-attended	428 (8.5)	479 (9.1)	512 (11.1)	525 (11.1)
Leading to study discontinuation	0	5 (<0.1)	1 (<0.1)	0
Grade 3 or higher	49 (1.0)	59 (1.1)	59 (1.3)	51 (1.1)
Non-serious*	1607 (32.0)	1549 (29.6)	1654 (35.7)	1533 (32.5)
Grade 3 or higher	31 (0.6)	31 (0.6)	46 (1.0)	31 (0.7)
Non-serious†	1621 (32.3)	1568 (29.9)	1665 (36.0)	1546 (32.8)
Grade 3 or higher	34 (0.7)	32 (0.6)	48 (1.0)	33 (0.7)
**Unsolicited TEAEs related to study vaccination**				
All	1204 (24.0)	1101 (21.0)	1220 (26.4)	1072 (22.8)
Serious	2 (<0.1)	0	1 (<0.1)	1 (<0.1)
Fatal	0	0	0	0
Medically-attended	48 (1.0)	52 (1.0)	59 (1.3)	47 (1.0)
Leading to study discontinuation	0	0	0	0
Grade 3 or higher	9 (0.2)	12 (0.2)	28 (0.6)	16 (0.3)
Non-serious*	1202 (23.9)	1101 (21.0)	1219 (26.3)	1071 (22.7)
Grade 3 or higher	8 (0.2)	12 (0.2)	27 (0.6)	16 (0.3)
Non-serious†	1203 (24.0)	1101 (21.0)	1219 (26.3)	1072 (22.8)
Grade 3 or higher	8 (0.2)	12 (0.2)	27 (0.6)	16 (0.3)

An adverse event (AE) is defined as any event not present before exposure to study vaccination or any event already present that worsens in intensity or frequency after exposure. Percentages are based on the number of Part C safety participants.
*Participants without any SAE and with any non-serious AE.
†Participants with at least one non-serious AE regardless of reporting any SAE or not.

Table S10. Summary of Unsolicited Adverse Events ≤28 Days After Booster by Race, Part C Safety Set

	White		Black or African American		Asian		American Indian or Alaska Native		Native Hawaiian or Other Pacific Islander		Multiracial		Other/Not Reported/Unknown	
	mRNA-1273 (N=7545)	Placebo-mRNA-1273 (N=7929)	mRNA-1273 (N=1048)	Placebo-mRNA-1273 (N=1031)	mRNA-1273 (N=406)	Placebo-mRNA-1273 (N=406)	mRNA-1273 (N=74)	Placebo-mRNA-1273 (N=78)	mRNA-1273 (N=27)	Placebo-mRNA-1273 (N=13)	mRNA-1273 (N=218)	Placebo-mRNA-1273 (N=221)	mRNA-1273 (N=329)	Placebo-mRNA-1273 (N=274)
**Unsolicited AEs Regardless of Relationship to Study Vaccination**														
All	2540 (33.7)	2498 (31.5)	326 (31.1)	277 (26.9)	151 (37.2)	146 (36.0)	34 (45.9)	31 (39.7)	9 (33.3)	2 (15.4)	110 (50.5)	83 (37.6)	133 (40.4)	103 (37.6)
Serious	29 (0.4)	50 (0.6)	6 (0.6)	6 (0.6)	0	0	1 (1.4)	1 (1.3)	0	0	2 (0.9)	0	4 (1.2)	1 (0.4)
Fatal	0	5 (<0.1)	1 (<0.1)	0	0	0	0	0	0	0	0	0	0	0
MAAE	723 (9.6)	825 (10.4)	104 (9.9)	83 (8.1)	36 (8.9)	40 (9.9)	9 (12.2)	8 (10.3)	1 (3.7)	0	32 (14.7)	21 (9.5)	35 (10.6)	27 (9.9)
Leading to study discontinuation	0	5 (<0.1)	1 (<0.1)	0	0	0	0	0	0	0	0	0	0	0
≥Grade 3	77 (1.0)	87 (1.1)	12 (1.1)	11 (1.1)	3 (0.7)	1 (0.2)	0	2 (2.6)	1 (3.7)	0	9 (4.1)	4 (1.8)	6 (1.8)	5 (1.8)
Non-serious*	2511 (33.3)	2448 (30.9)	320 (30.5)	271 (26.3)	151 (37.2)	146 (36.0)	33 (44.6)	30 (38.5)	9 (33.3)	2 (15.4)	108 (49.5)	83 (37.6)	129 (39.2)	102 (37.2)
≥Grade 3	55 (0.7)	45 (0.6)	8 (0.8)	6 (0.6)	3 (0.7)	1 (0.2)	0	2 (2.6)	1 (3.7)	0	7 (3.2)	4 (1.8)	3 (0.9)	4 (1.5)
Non-serious†	2529 (33.5)	2474 (31.2)	322 (30.7)	276 (26.8)	151 (37.2)	146 (36.0)	34 (45.9)	30 (38.5)	9 (33.3)	2 (15.4)	110 (50.5)	83 (37.6)	131 (39.8)	103 (37.6)
≥Grade 3	58 (0.8)	47 (0.6)	9 (0.9)	7 (0.7)	3 (0.7)	1 (0.2)	0	2 (2.6)	1 (3.7)	0	7 (3.2)	4 (1.8)	4 (1.2)	4 (1.5)
**Unsolicited TEAEs Related to Study Vaccination**														
All	1876 (24.9)	1714 (21.6)	223 (21.3)	189 (18.3)	115 (28.3)	98 (24.1)	27 (36.5)	23 (29.5)	8 (29.6)	2 (15.4)	83 (38.1)	66 (29.9)	92 (28.0)	81 (29.6)
Serious	1 (<0.1)	1 (<0.1)	0	0	0	0	0	0	0	0	1 (0.5)	0	1 (0.3)	0
Fatal	0	0	0	0	0	0	0	0	0	0	0	0	0	0
MAAE	87 (1.2)	82 (1.0)	8 (0.8)	6 (0.6)	6 (1.5)	5 (1.2)	1 (1.4)	1 (1.3)	0	0	2 (0.9)	2 (0.9)	3 (0.9)	3 (1.1)
Leading to study discontinuation	0	0	0	0	0	0	0	0	0	0	0	0	0	0
≥Grade 3	27 (0.4)	21 (0.3)	3 (0.3)	3 (0.3)	2 (0.5)	0	0	1 (1.3)	0	0	3 (1.4)	2 (0.9)	2 (0.6)	1 (0.4)
Non-serious*	1875 (24.9)	1713 (21.6)	223 (21.3)	189 (18.3)	115 (28.3)	98 (24.1)	27 (36.5)	23 (29.5)	8 (29.6)	2 (15.4)	82 (37.6)	66 (29.9)	91 (27.7)	81 (29.6)
≥Grade 3	26 (0.3)	21 (0.3)	3 (0.3)	3 (0.3)	2 (0.5)	0	0	1 (1.3)	0	0	2 (0.9)	2 (0.9)	2 (0.6)	1 (0.4)
Non-serious†	1875 (24.9)	1714 (21.6)	223 (21.3)	189 (18.3)	115 (28.3)	98 (24.1)	27 (36.5)	23 (29.5)	8 (29.6)	2 (15.4)	83 (38.1)	66 (29.9)	91 (27.7)	81 (29.6)
≥Grade 3	26 (0.3)	21 (0.3)	3 (0.3)	3 (0.3)	2 (0.5)	0	0	1 (1.3)	0	0	2 (0.9)	2 (0.9)	2 (0.6)	1 (0.4)

An adverse event (AE) is defined as any event not present before exposure to study vaccination or any event already present that worsens in intensity or frequency after exposure. Percentages are based on the number of Part C safety participants.
*Participants without any SAE and with any non-serious AE.
†Participants with at least one non-serious AE regardless of reporting any SAE or not.

Table S11. Summary of Unsolicited Adverse Events ≤28 Days After Booster by Ethnicity, Part C Safety Set

	Hispanic or Latino		Not Hispanic or Latino		Not Reported/Unknown	
	mRNA-1273 100 µg Primary Series (N=2002)	Placebo-mRNA-1273 100 µg Primary Series (N=1948)	mRNA-1273 100 µg Primary Series (N=7553)	Placebo-mRNA-1273 100 µg Primary Series (N=7913)	mRNA-1273 100 µg Primary Series (N=92)	Placebo-mRNA-1273 100 µg Primary Series (N=91)
**Unsolicited AEs Regardless of Relationship to Study Vaccination**						
All	742 (37.1)	656 (33.7)	2527 (33.5)	2446 (30.9)	34 (37.0)	38 (41.8)
Serious	9 (0.4)	15 (0.8)	31 (0.4)	43 (0.5)	2 (2.2)	0
Fatal	1 (<0.1)	2 (0.1)	0	3 (<0.1)	0	0
MAAE	208 (10.4)	201 (10.3)	719 (9.5)	792 (10.0)	13 (14.1)	11 (12.1)
Leading to study discontinuation	1 (<0.1)	2 (0.1)	0	3 (<0.1)	0	0
≥Grade 3	23 (1.1)	26 (1.3)	83 (1.1)	82 (1.0)	2 (2.2)	2 (2.2)
Non-serious*	733 (36.6)	641 (32.9)	2496 (33.0)	2403 (30.4)	32 (34.8)	38 (41.8)
≥Grade 3	14 (0.7)	13 (0.7)	62 (0.8)	47 (0.6)	1 (1.1)	2 (2.2)
Non-serious†	739 (36.9)	651 (33.4)	2515 (33.3)	2425 (30.6)	32 (34.8)	38 (41.8)
≥Grade 3	15 (0.7)	15 (0.8)	66 (0.9)	48 (0.6)	1 (1.1)	2 (2.2)
**Unsolicited AEs Related to Study Vaccination**						
All	541 (27.0)	461 (23.7)	1861 (24.6)	1685 (21.3)	22 (23.9)	27 (29.7)
Serious	1 (<0.1)	1 (<0.1)	1 (<0.1)	0	1 (1.1)	0
Fatal	0	0	0	0	0	0
MAAE	22 (1.1)	15 (0.8)	85 (1.1)	83 (1.0)	0	1 (1.1)
Leading to study discontinuation	0	0	0	0	0	0
≥Grade 3	8 (0.4)	5 (0.3)	29 (0.4)	22 (0.3)	0	1 (1.1)
Non-serious*	540 (27.0)	460 (23.6)	1860 (24.6)	1685 (21.3)	21 (22.8)	27 (29.7)
≥Grade 3	7 (0.3)	5 (0.3)	28 (0.4)	22 (0.3)	0	1 (1.1)
Non-serious†	541 (27.0)	461 (23.7)	1860 (24.6)	1685 (21.3)	21 (22.8)	27 (29.7)
≥Grade 3	7 (0.3)	5 (0.3)	28 (0.4)	22 (0.3)	0	1 (1.1)

An adverse event (AE) is defined as any event not present before exposure to study vaccination or any event already present that worsens in intensity or frequency after exposure. Percentages are based on the number of Part C safety participants.
*Participants without any SAE and with any non-serious AE.
†Participants with at least one non-serious AE regardless of reporting any SAE or not.

Table S12. Summary of Unsolicited Adverse Events ≤28 Days After Booster by Age Groups, Part C Safety Set

	≥18-<65 Years		≥65 Years	
	mRNA-1273 100 µg Primary Series (N=7027)	Placebo-mRNA-1273 100 µg Primary Series (N=7176)	mRNA-1273 100 µg Primary Series (N=2620)	Placebo-mRNA-1273 100 µg Primary Series (N=2776)
**Unsolicited AEs Regardless of Relationship to Study Vaccination**				
All	2488 (35.4)	2296 (32.0)	815 (31.1)	844 (30.4)
Serious	23 (0.3)	31 (0.4)	19 (0.7)	27 (1.0)
Fatal	1 (<0.1)	3 (<0.1)	0	2 (<0.1)
Medically-attended	679 (9.7)	698 (9.7)	261 (10.0)	306 (11.0)
Leading to study discontinuation	1 (<0.1)	3 (<0.1)	0	2 (<0.1)
Grade 3 or higher	70 (1.0)	74 (1.0)	38 (1.5)	36 (1.3)
Non-serious*	2465 (35.1)	2265 (31.6)	796 (30.4)	817 (29.4)
Grade 3 or higher	52 (0.7)	47 (0.7)	25 (1.0)	15 (0.5)
Non-serious†	2475 (35.2)	2285 (31.8)	811 (31.0)	829 (29.9)
Grade 3 or higher	54 (0.8)	50 (0.7)	28 (1.1)	15 (0.5)
**Unsolicited AEs related to study vaccination**				
All	1853 (26.4)	1623 (22.6)	571 (21.8)	550 (19.8)
Serious	2 (<0.1)	0	1 (<0.1)	1 (<0.1)
Fatal	0	0	0	0
Medically-attended	81 (1.2)	75 (1.0)	26 (1.0)	24 (0.9)
Leading to study discontinuation	0	0	0	0
Grade 3 or higher	24 (0.3)	19 (0.3)	13 (0.5)	9 (0.3)
Non-serious*	1851 (26.3)	1623 (22.6)	570 (21.8)	549 (19.8)
Grade 3 or higher	23 (0.3)	19 (0.3)	12 (0.5)	9 (0.3)
Non-serious†	1852 (26.4)	1623 (22.6)	570 (21.8)	550 (19.8)
Grade 3 or higher	23 (0.3)	19 (0.3)	12 (0.5)	9 (0.3)

An adverse event (AE) is defined as any event not present before exposure to study vaccination or any event already present that worsens in intensity or frequency after exposure. Percentages are based on the number of Part C safety participants.
*Participants without any SAE and with any non-serious AE.
†Participants with at least one non-serious AE regardless of reporting any SAE or not.

Table S13. Summary of Unsolicited Adverse Events up to Study-end After Booster, Part C Safety Set

n (%)	Part C Booster mRNA-1273 100 µg Primary Series (N=9647)	Placebo-mRNA-1273 100 µg Primary Series (N=9952)	Total (N=19609)
**Unsolicited AEs regardless of relationship to study vaccination**			
All	6393 (66.3)	6426 (64.6)	12820 (65.4)
Serious	374 (3.9)*	381 (3.8)†	755 (3.9)
Fatal§	23 (0.2)	27 (0.3)	50 (0.3)
Medically-attended	5136 (53.2)	5243 (52.7)	10379 (52.9)
Leading to study discontinuation	23 (0.2)	30 (0.3)	53 (0.3)
Grade 3 or higher	582 (6.0)	552 (5.5)	1134 (5.8)
Non-serious*	6019 (62.4)	6045 (60.7)	12065 (61.5)
Grade 3 or higher	280 (2.9)	240 (2.4)	520 (2.7)
Non-serious†	6336 (65.7)	6359 (63.9)	12696 (64.7)
Grade 3 or higher	338 (3.5)	311 (3.1)	649 (3.3)
**Unsolicited AEs related to study vaccination**			
All	2429 (25.2)	2189 (22.0)	4618 (23.6)
Serious	3 (<0.1)	1 (<0.1)	4 (<0.1)
Fatal	0	0	0
Medically-attended	113 (1.2)	114 (1.1)	227 (1.2)
Leading to study discontinuation	0	0	0
Grade 3 or higher	37 (0.4)	30 (0.3)	67 (0.3)
Non-serious*	2426 (25.1)	2188 (22.0)	4614 (23.5)
Grade 3 or higher	35 (0.4)	30 (0.3)	65 (0.3)
Non-serious†	2427 (25.2)	2189 (22.0)	4616 (23.5)
Grade 3 or higher	35 (0.4)	30 (0.3)	65 (0.3)

Adverse event is defined as any event not present before exposure to study vaccination or any event already present that worsens in intensity or frequency after exposure. Percentages are based on the number of participants in the primary series safety set.

*Participants without any serious AEs and with any non-serious AE.

†Participants with at least one non-serious AE regardless of reporting any serious AE or not.

§Four deaths were associated with COVID-19. Two deaths occurred in participants aged ≥60 years during Part B; one attributed to COVID-19 and pneumonia bacterial in a male on open-label day 293 post-vaccination, and another due to acute respiratory failure on open-label day 115 post-vaccination in a female who had an ongoing life-threatening COVID-19 infection with onset 1 month prior to the fatal event. Two deaths in male participants aged ≥65 years occurred after the booster in Part C, attributed to respiratory failure (updated to COVID-19 after database lock) on study day 98 after the booster dose, and COVID-19 (symptomatic COVID-19) on day 39 after the booster dose, respectively.

Table S14. Genotypic Analysis of Adjudicated Cases

	All Cases	Severe Cases
**Parts A and B: Starting 14 Days After Injection 2 of the Primary Series (Per-Protocol Primary Series Set)**		
Number of participants	24914	24914
Number of participants with COVID-19[a], N1 (N1/N%)	1055 (4.2)	102 (0.4)
Number of participants by variant group, n (n/N1%)		
Delta	486 (46.1)	55 (53.9)
Omicron Variant Lineages	196 (18.6)	12 (11.8)
BA.1	171 (16.2)	11 (10.8)
BA.2	8 (0.8)	1 (1.0)
BA.4	2 (0.2)	0
BA.5	15 (1.4)	0
Alpha	11 (1.0)	3 (2.9)
Epsilon	7 (0.7)	1 (1.0)
Gamma	3 (0.3)	0
Iota	1 (0.1)	0
Lambda	1 0.1)	0
Mu	1 (0.1)	0
Other Variants	21 (2.0)	0
No Sequencing Data Available	328 (31.1)	31 (30.4)
**Part C: Starting 14 Days After Booster (Part C Per-Protocol Set)**		
Number of participants	16368	16368
Number of participants with COVID-19[b], N1 (N1/N%)	3538 (21.6)	167 (1.0)
Number of participants by variant group, n (n/N1%)		
Delta	14 (0.4)	1 (0.6)
Omicron Variant Lineages	2653 (75.0)	116 (69.5)
BA.1	1109 (31.3)	47 (28.1)
BA.2	744 (21.0)	34 (20.4)
BA.4	144 (4.1)	6 (3.6)
BA.5	647 (18.3)	29 (17.4)
Other Omicron Variants	9 (0.3)	0
BQ1.1	3 (0.1)	0
Other Variants	26 (0.7)	2 (1.2)
No Sequencing Data Available	845 (23.9)	48 (28.7)

BD = booster dose; COVID-19 = coronavirus disease 2019; RT-PCR = reverse transcription polymerase chain reaction.
A COVID-19 case was based on eligible symptoms and positive RT-PCR within 14 days (with the censoring rules for effectiveness analyses). If a participant had SARS-CoV-2 infection regardless of symptom defined using positive RT-PCR or Elecsys on or before the date of Dose 1 of mRNA-1273 primary series, the participant was censored at the date of dose 1 of mRNA-1273 primary series.bCOVID-19 case was based on eligible symptoms and positive RT-PCR within 14 days (with the censoring rules for effectiveness analyses). If a participant had SARS-CoV-2 infection regardless of symptom defined using positive RT-PCR or Elecsys on or before the date of booster dose (or BD-1 visit if they did not receive booster), the participant was censored at the date of booster dose (or BD-1 visit if they did not receive booster).

Table S15. Adjudicated COVID-19 Incidence Starting 14 days After Injection 2 of mRNA-1273 Primary Series, by Calendar Quarters, Per-Protocol Primary Series Set

Cases	COVID-19*adjudicated cases		
	mRNA-1273<br>N=14291	Placebo-mRNA-1273<br>N=10623	Total<br>N=24914
≥14 Days After Injection 2			
Event, n (%) [1]	668 (4.7)	387 (3.7)	1055 (4.3)
Person months	169448.3	84092.0	253540.3
Incidence/1000 person months (95% CI)	3.94 (3.65-4.25)	4.60 (4.16-5.08)	4.16 (3.91-4.42)
July 1, 2020-September 30, 2020			
Event, n (%) [1]	0	0	0
Person months	1636.8	0	1636.8
Incidence/1000 person months (95% CI)	0.00 (NE-2.25)	0	0.00 (NE-2.25)
October 1, 2020-December 1, 2020			
Event, n (%) [1]	40 (0.3)	0	40 (0.3)
Person months	36707.1	0	36707.1
Incidence/1000 person months (95% CI)	1.09 (0.78-1.48)	0	1.09 (0.78-1.48)
January 1, 2021-March 31, 2021			
Event, n (%) [1]	44 (0.3)	1 (<0.1)	45 (0.2)
Person months	40794.9	9337.5	50132.4
Incidence/1000 person months (95% CI)	1.08 (0.78-1.45)	0.11 (0.00-0.60)	0.90 (0.66-1.20)
April 1, 2021-June 30, 2021			
Event, n (%) [1]	28 (0.2)	9 (<0.1)	37 (0.2)
Person months	39916.5	30662.7	70579.1
Incidence/1000 person months (95% CI)	0.70 (0.47-1.01)	0.29 (0.13-0.56)	0.52 (0.37-0.72)
July 1, 2021-September 30, 2021			
Event, n (%) [1]	348 (2.7)	204 (2.0)	552 (2.4)
Person months	35523.6	30549.0	66072.7
Incidence/1000 person months (95% CI)	9.80 (8.79-10.88)	6.68 (5.79-7.66)	8.35 (7.67-9.08)
October 1, 2021-December 31, 2021			
Event, n (%) [1]	89 (0.9)	79 (0.9)	168 (0.9)
Person months	10996.5	10957.3	21953.8
Incidence/1000 person months (95% CI)	8.09 (6.50-9.96)	7.21 (5.71-8.99)	7.65 (6.54-8.90)
January 1, 2022-March 31, 2022			
Event, n (%) [1]	97 (11.3)	77 (11.0)	174 (11.2)
Person months	1642.4	1176.7	2819.2
Incidence/1000 person months (95% CI)	59.06 (47.89-72.05)	65.44 (51.64-81.78)	61.72 (52.89-71.60)
April 1, 2022-June 30, 2022			
Event, n (%) [1]	14 (3.5)	5 (1.8)	19 (2.8)
Person months	1120.3	719.8	1840.1
Incidence/1000 person months (95% CI)	12.50 (6.83-20.97)	6.95 (2.26-16.21)	10.33 (6.22-16.12)
July 1, 2022-September 30, 2022			
Event, n (%) [1]	8 (2.3)	12 (5.5)	20 (3.5)
Person months	898.0	565.4	1463.4
Incidence/1000 person months (95% CI)	8.91 (3.85-17.55)	21.22 (10.97-37.08)	13.67 (8.35-21.11)
October 1, 2022-December 31, 2022			
Event, n (%) [1]	0	0	0
Person months	208.0	122.3	330.3
Incidence/1000 person months (95% CI)	0.00 (NE-17.74)	0.00 (NE-30.15)	0.00 (NE-11.17)
January 1, 2023-March 31, 2023			
Event, n (%) [1]	0	0	0
Person months	4.2	1.1	5.4
Incidence/1000 person months (95% CI)	0.00 (NE-870.39)	0.00 (NE-3208.01)	0.00 (NE-684.64)

CI = confidence interval; NE = not estimable.
The per-protocol primary series set includes participants who are in Part A per-protocol set and received two doses of mRNA-1273 primary series in either Part A or Part B per scheduled without major protocol deviations.
[1] Percentages are based on N1.
[2] Person-months for each time period is defined as the total months from the start of each time period or 14 days after the date of Dose 2 of mRNA-1273 100 µg Primary Series, whichever is later, to the date of event, one day before the date of booster if received, the end of each time period, last date of study participation, or effectiveness data cutoff date, whichever is the earliest. 1 month = 30.4375 days.
[3] Incidence for each time period is defined as the number of participants with an event during the time period divided by the number of participants at risk during the time period and adjusted by person-months (total time at risk) in each treatment group. The 95% CI is calculated using the exact method (Poisson distribution) and adjusted by person-months.

Table S16. Incidences of Secondary Endpoints Starting 14 days After Injection 2 of mRNA-1273 Primary Series, Per-Protocol Primary Series Set

Secondary Endpoint<br>Per-Protocol Primary Series Set	mRNA-1273 100 µg<br>Primary Series<br>(N=14291)	Placebo-mRNA-1273 100 µg<br>Primary Series<br>(N=10623)
COVID-19 (primary definition)*		
Cases, n (%)†	683 (4.8)	391 (3.7)
Person-months§	169352.4	84064.4
Incidence/1000 person-months (95% CI)¶	4.03<br>(3.74-4.35)	4.65<br>(4.20-5.14)
COVID-19 (CDC definition)*		
Cases, n (%)†	729 (5.1)	412 (3.9)
Person-months§	169055.7	83973.4
Incidence/1000 person-months (95% CI)¶	4.31 (4.01-4.64)	4.91 (4.44-5.40)
Severe COVID-19		
Cases, n (%)†	77 (0.5)	33 (0.3)
Person-months§	172356.2	85725.7
Incidence/1000 person-months (95% CI)¶	0.45 (0.35-0.56)	0.39 (0.27-0.54)
SARS-CoV-2 infection (regardless of symptoms)		
Cases, n (%)†	1415 (9.9)	802 (7.7)
Person-months§	165722.7	83015.2
Incidence/1000 person-months (95% CI)¶	8.54 (8.10-9.00)	9.66 (9.00-10.35)
COVID-19 Deaths		
Cases, n (%)†	0	0
Person-months§	172672.3	86490.1
Incidence/1000 person-months (95% CI)¶	0.00<br>(NE-0.02)	0.00<br>(NE- 0.04)

CDC = Centers for Disease Control and Prevention; CI = confidence interval; COVID-19 = coronavirus disease 2019; SARS-CoV-2 = severe acute respiratory syndrome coronavirus-2; NE = not estimable.
The per-protocol primary series set includes participants in Part A per-protocol set who received two doses of mRNA-1273 primary series in either Part A or Part B per scheduled without major protocol deviations.
*COVID-19 cases based on 2 symptoms per primary efficacy definition (COVE) or 1 symptom per CDC definition
†Percentages are based on N1.
§Person-months for each time period is defined as the total months from the start of each time period to the date of event, one day before the date of booster if received, the end of each time period, last date of study participation, or effectiveness data cutoff date, whichever is the earliest. 1 month = 30.4375 days.
¶Incidence rate for each time period is defined as the number of participants with an event during the time period divided by the number of participants at risk during the time period and adjusted by person-months (total time at risk) in each treatment group. The 95% CI is calculated using the exact method (Poisson distribution) and adjusted by person-months.

Table S17. Summary of Incidence Rates for Other Effectiveness Endpoints (Part C Per-Protocol Set)

	Part C booster	
	**mRNA-1273 100 µg Primary Series (N=8500)**	**Placebo-mRNA-1273 100 µg Primary Series (N=7868)**
COVID-19 (Adjudicated)		
Number of participants at risk (N1)	8238	7733
Number of participants with event, n (%) [a]	1747 (21.2)	1791 (23.2)
Incidence/1000 person-months (95% CI) [b]	25.43 (24.25-26.65)	29.83 (28.46-31.24)
COVID-19 (Any)		
Number of participants at risk (N1)	8238	7733
Number of participants with event, n (%) [a]	1745 (21.2)	1787 (23.1)
Incidence/1000 person-months (95% CI) [b]	25.40 (24.22-26.62)	29.75 (28.39-31.16)
Severe COVID-19 (adjudicated)		
Number of participants at risk (N1)	8259	7747
Number of participants with event, n (%) [a]	84 (1.0)	83 (1.1)
Incidence/1000 person-months (95% CI) [b]	1.10 (0.87-1.36)	1.21 (0.96-1.50)
Severe COVID-19 (Any)		
Number of participants at risk (N1)	8259	7747
Number of participants with event, n (%) [a]	90 (1.1)	88 (1.1)
Incidence/1000 person-months (95% CI) [b]	1.17 (0.94-1.44)	1.28 (1.03-1.58)
CDC definition of COVID-19		
Number of participants at risk (N1)	8236	7731
Number of participants with event, n (%) [a]	1841 (22.4)	1876 (24.3)
Incidence/1000 person-months (95% CI) [b]	27.02 (25.80-28.28)	31.51 (30.10-32.97)
Death caused by COVID-19		
Number of participants at risk (N1)	8471	7848
Number of participants with event, n (%) [a]	0	0
Incidence/1000 person-months (95% CI) [b]	0.00 (NE-0.05)	0.00 (NE-0.05)
Asymptomatic SARS-CoV-2 infection		
Number of participants at risk (N1)	8238	7733
Number of participants with event, n (%) [a]	1728 (21.0)	1441 (18.6)
Incidence/1000 person-months (95% CI) [b]	27.02 (25.76-28.32)	25.76 (24.45-27.13)
SARS-CoV-2 infection regardless of symptomatology and severity		
Number of participants at risk (N1)	8236	7731
Number of participants with event, n (%) [a]	3539 (43.0)	3289 (42.5)
Incidence/1000 person-months (95% CI) [b]	55.48 (53.67-57.34)	58.98 (56.98-61.03)

CI = confidence interval; NE=not estimable.
a Percentages are based on N1.
b Incidence is defined as the number of participants with an event starting 14 days after booster divided by the number of participants at risk at 14 days after booster and adjusted by person-months (total time at risk) in each treatment group. The 95% CI is calculated using the exact method (Poisson distribution) and adjusted by person-months. 1 month = 30.4375 days.

Table S18. Analysis of Incidence Rates of Severe COVID-19 Based on Adjudication Committee Assessments Starting 14 Days After Booster, by Pre-booster SARS-CoV-2 Status (Part C Safety Set)

	SARS-CoV-2 Negative			SARS-CoV-2 Positive		
	mRNA-1273 100 µg Primary Series[a]	Placebo-mRNA-1273 100 µg Primary Series	Total	mRNA-1273 100 µg Primary Series[a]	Placebo-mRNA-1273 100 µg Primary Series	Total
	N=8705	N=8305	N=17010	N=941	N=1647	N=2588
**≥14 days after booster**						
Number at risk (N1)	8452	8046	16498	120	119	239
Participants with event, n (%)[a]	87 (1.0)	88 (1.1)	175 (1.1)	1 (0.8)	0	1 (0.4)
Person-months[b]	78557.2	71501.9	150059.1	1159.4	1145.5	2304.9
Incidence/1000 person-months (95% CI)[c]	1.11 (0.89-1.37)	1.23 (0.99-1.52)	1.17 (1.00-1.35)	0.86 (0.02-4.81)	0.00 (NE-3.22)	0.43 (0.01- 2.42)
**September 1, 2021-November 30, 2021 (Delta predominant)**						
Number at risk (N1)	7179	6502	13681	92	98	190
Participants with event, n (%)[a]	1 (<0.1)	0	1 (<0.1)	0	0	0
Person-months[b]	6692.0	5847.8	12539.8	78.8	78.7	157.5
Incidence/1000 person-months (95% CI)[c]	0.15 (0.00-0.83)	0.00 (NE-0.63)	0.08 (0.00-0.44)	0.00 (NE-46.80)	0.00 (NE-46.88)	0.00 (NE-23.42)
**December 1, 2021-March 31, 2022 (Omicron BA.1 predominant)**						
Number at risk (N1)	8430	8025	16455	115	119	234
Participants with event, n (%)[a]	38 (0.5)	41 (0.5)	79 (0.5)	1 (0.9)	0	1 (0.4)
Person-months[b]	32188.3	30027.4	62215.7	421.4	438.3	859.7
Incidence/1000 person-months (95% CI)[c]	1.18 (0.84-1.62)	1.37 (0.98-1.85)	1.27 (1.01-1.58)	2.37 (0.06-13.22)	0.00 (NE-8.42)	1.16 (0.03-6.48)
**April 1, 2022-June 30, 2022 (Omicron BA.2 predominant)**						
Number at risk (N1)	8109	7393	15502	114	111	225
Participants with event, n (%)[a]	26 (0.3)	29 (0.4)	55 (0.4)	0	0	0
Person-months[b]	21345.5	19381.7	40727.2	328.6	312.5	641.1
Incidence/1000 person-months (95% CI)[c]	1.22 (0.80-1.79)	1.50 (1.00-2.15)	1.35 (1.02-1.76)	0.00 (NE-11.23)	0.00 (NE-11.80)	0.00 (NE-5.75)
**July 1, 2022-November 30, 2022 (Omicron BA.4/5 predominant)**						
Number at risk (N1)	6510	5845	12355	106	100	206
Participants with event, n (%)[a]	22 (0.3)	18 (0.3)	40 (0.3)	0	0	0
Person-months[b]	18244.2	16185.7	34429.9	327.9	314.1	642.0
Incidence/1000 person-months (95% CI)[c]	1.21 (0.76-1.83)	1.11 (0.66-1.76)	1.16 (0.83-1.58)	0.00 (NE-11.25)	0.00 (NE-11.75)	0.00 (NE-5.75)
**After December 1, 2022 (Omicron BQ.1.1 predominant)**						
Number at risk (N1)	179	128	307	4	3	7
Participants with event, n (%)[a]	0	0	0	0	0	0
Person-months[b]	87.2	59.3	146.5	2.7	1.9	4.6
Incidence/1000 person-months (95% CI)[c]	0.00 (NE-42.31)	0.00 (NE-62.21)	0.00 (NE-25.18)	0.00 (NE-1369.27)	0.00 (NE-1903.06)	0.00 (NE-796.31)

CI = confidence interval ; NE = not estimable.
Part C safety set included all participants in the per-protocol set who received mRNA-1273 primary series (Part A) followed by an mRNA-1273 50-µg booster dose.
[a]One participant from the mRNA-1273 group had a missing pre-booster SARS-CoV-2 status.
[b]Percentages are based on N1.
[c]Person-months for each time period is defined as the total months from the start of each time period to the date of event, the end of each time period, last date of study participation, or effectiveness data cutoff date, whichever is the earliest. 1 month = 30.4375 days.
[d]Incidence for each time period is defined as the number of participants with an event during the time period divided by the number of participants at risk during the time period and adjusted by person-months (total time at risk) in each treatment group. The 95% CI is calculated using the exact method (Poisson distribution) and adjusted by person-months.

Table S19. Pseudovirus Neutralizing Antibody Against SARS-CoV-2 (D614G) from Pre-vaccination Baseline, Part C Per-Protocol Immunogenicity Subset by SARS-CoV-2 Status

Time point	PPIS<br>mRNA-1273 100 µg Primary series N=731	PPIS-negative<br>mRNA-1273 100 µg Primary Series (N=682)	PPIS-positive<br>mRNA-1273 100 µg Primary Series (N=49)
Baseline (pre-vaccination)			
n[a]	728	680	48
GMC	9.3	9.3	9.6
(95% CI)[b]	(8.9-9.7)	(8.9-9.7)	(8.4-11.1)
Day 57			
n [a]	728	680	48
GMC	1111.0	1111.3	1106.7
(95% CI)[b]	(1043.8-1182.5)	(1041.7-1185.5)	(864.4-1416.7)
N1	728	680	48
GMFR (vs. pre-vaccination GMC)	119.1	119.4	114.9
(95% CI)[b]	(110.7-128.1)	(110.7-128.7)	(85.6-154.2)
SRR %, from pre-vaccination[d] (n/N1)[e]	98.9 (720/728)	98.8 (672/680)	100 (48/48)
(95% CI)[f]	(97.8-99.5)	(97.7-99.5)	(92.6-100.0)
Day 209			
n [a]	684	641	43
GMC	238.2	237.4	250.6
(95% CI)[b]	(222.6-255.0)	(221.9-254.0)	(166.5-377.1)
N1	682	639	43
GMFR (vs. pre-vaccination GMC)	25.7	25.8	25.5
(95% CI)[b]	(23.8-27.9)	(23.8-27.9)	(15.7-41.5)
SRR %, from pre-vaccination[d] (n/N1)[e]	95.6 (652/682)	95.9 (613/639)	90.7 (39/43)
(95% CI)[f]	(93.8-97.0)	(94.1-97.3)	(77.9-97.4)
Pre-booster			
n [a]	700	660	40
GMC	163.8	132.2	5641.1
(95% CI)[b]	(148.0-181.4)	(122.6-142.6)	(3079.3-10334.0)
N1	697	658	39
GMFR (vs. pre-vaccination GMC)	18.0	14.6	626.5
(95% CI)[b]	(16.1-20.0)	(13.4-15.8)	(338.4-1159.7)
SRR %, from pre-vaccination[d] (n/N1)[e]	87.8 (612/697)	87.1 (573/658)	100 (39/39)
(95% CI)[f]	(85.1-90.1)	(84.3-89.5)	(91.0-100.0)
BD-29			
n[a]	678	638	40
GMC	8130.9	7739.7	17854.8
(95% CI)[b]	(7611.7-8685.6)	(7240.9-8272.8)	(13854.3-23010.5)
N1	675	636	39
GMFR (vs. pre-vaccination GMC)	882.4	841.7	1907.1
(95% CI)[b]	(817.4-952.6)	(779.0-909.4)	(1374.0- 2647.0)
SRR %, from pre-vaccination[d] (n/N1)[e]	100.0 (675/675)	100.0 (636/636)	100.0 (39/39)
(95% CI)[f]	(99.5-100.0)	(99.4-100.0)	(91.0-100.0)
BD-181			
n[a]	610	575	35
GMC	2951.1	2872.1	4609.7
(95% CI)[b]	(2701.6-3223.6)	(2625.2-3142.2)	(3005.4-7070.3)
N1	608	574	34
GMFR (vs. pre-vaccination GMC)	314.3	305.9	497.2
(95% CI)[b]	(285.4-346.1)	(277.3-337.4)	(305.1-810.0)
SRR %, from pre-vaccination[d] (n/N1)[e]	99.7 (606/608)	99.7 (572/574)	100 (34/34)
(95% CI)[f]	(98.8-100.0)	(98.7-100.0)	(89.7-100.0)

BD-29 and BD-181 = booster doses on days 29 and 181, respectively; CI = confidence interval, GM = geometric mean; GMC = geometric mean concentration [AU/mL]; GMFR = geometric mean fold-rise; LLOQ = lower limit of quantification; N1 = number of participants with non-missing data at baseline and the corresponding time point; NE = not estimable; SRR = seroresponse rate; ULOQ = upper limit of quantification.
Antibody values reported as below the LLOQ are replaced by 0.5 x LLOQ. Values greater than the ULOQ are replaced by the ULOQ if actual values are not available. Pre-vaccination baseline was evaluated prior to primary vaccination injection 1 on day 1 (Part A).
[a]Number of participants with non-missing data at the corresponding time point.
[b]95% CI is calculated based on the t-distribution of the log-transformed values or the difference in the log- transformed values for GM value and GMFR, respectively, then back transformed to the original scale for presentation.
[c]Percentages are based on n in footnote a.
[d]Seroresponse at a partcipant level is defined as a change from below the LLOQ to ≥ 4 x LLOQ, or at least a 4-fold rise if baseline is equal to or above the LLOQ.
[e]Number of participants meeting the criterion at the time point. Percentages are based on N1.
[f]95% CI is calculated using the Clopper-Pearson method.

Table S20. Pseudovirus Neutralizing Antibody Against SARS-CoV-2 (D614G) from Pre-booster Baseline, Part C Per-Protocol Immunogenicity Subset by SARS-CoV-2 Status

Time point	PPIS<br>mRNA-1273 100 µg<br>Primary series<br>N=731	PPIS-negative<br>mRNA-1273 100 µg<br>Primary Series<br>N=682	PPIS-positive<br>mRNA-1273 100 µg<br>Primary Series<br>N=49
Pre-booster (BD Day 1)			
n[a]	700	660	40
GMC	163.8	132.2	5641.1
(95% CI)[b]	(148.0-181.4)	(122.6-142.6)	(3079.3- 10334.0)
BD-29			
n [a]	677	637	40
GMC	8122.2	7730.2	17854.8
(95% CI) [b]	(7603.0-8676.8)	(7231.6-8263.2)	(13854.3- 23010.5)
N1	670	632	38
GMFR (vs. pre-booster GMC)	50.2	59.1	3.2
(95% CI)[b]	(45.6-55.1)	(54.6-64.1)	(2.0-5.2)
SRR, % from pre-booster[d] (n/N1)[e]	94.5 (633/670)	98.4 (622/632)	28.9 (11/38)
(95% CI)[f]	(92.5-96.1)	(97.1-99.2)	(15.4-45.9)
BD-181			
n [a]	610	575	35
GMC	2951.1	2872.1	4609.7
(95% CI) [b]	(2701.6-3223.6)	(2625.2-3142.2)	(3005.4-7070.3)
N1	591	561	30
GMFR (vs. pre-booster GMC)	17.9	21.3	0.7
(95% CI)[b]	(16.0-20.2)	(19.2-23.6)	(0.4-1.1)
SRR, % from pre-booster[d] (n/N1)[e]	89.2 (527/591)	93.6 (525/561)	6.7 (2/30)
(95% CI)[f]	(86.4-91.6)	(91.2-95.5)	(0.8-22.1)

BD-29 and BD-181 = booster doses on days 29 and 181, respectively; CI = confidence interval; GM = geometric mean; GMC = geometric mean concentration [AU/mL]; GMFR = geometric mean fold-rise, LLOQ = lower limit of quantification; N1 = Number of participants with non-missing data at pre-booster and the corresponding time point; PPIS=per protocol immunogenicity set; SRR = seroresponse rate; ULOQ = upper limit of quantification. Antibody values reported as below the LLOQ are replaced by 0.5 x LLOQ. Values greater than the ULOQ are replaced by the ULOQ if actual values are not available. Pre-booster baseline was evaluated prior to booster dose on day (BD-1).

[a]Number of participants with non-missing data at the corresponding time point.

[b]95% CI is calculated based on the t-distribution of the log-transformed values or the difference in the log- transformed values for GM value and GMFR, respectively, then back transformed to the original scale for presentation.

[c]Percentages are based on n in footnote a.

[d]Seroresponse at a participant level is defined as a change from below the LLOQ to ≥ 4 x LLOQ, or at least a 4-fold rise if pre-booster is equal to or above the LLOQ.

[e]Number of participants meeting the criterion at the time point. Percentages are based on N1.

[f]95% CI is calculated using the Clopper-Pearson method.

Table S21. Pseudovirus Neutralizing Antibody Against SARS-CoV-2 (D614G) from Pre-booster Baseline, Part C PPIS by SARS-CoV-2-status and Age

	PPIS-negative		PPIS-positive	
	≥18-<65 years	≥65 years	≥18-<65 years	≥65 years
Time point	mRNA-1273 100 µg Primary series N=439	mRNA-1273 100 µg Primary Series N=243	mRNA-1273 100 µg Primary series N=34	mRNA-1273 100 µg Primary Series N=15
Baseline (pre-vaccination)				
n[a]	438	242	33	15
GMC	9.7	8.7	9.2	10.7
(95% CI)[b]	(9.1-10.3)	(8.2-9.2)	(7.7-11.0)	(8.4-13.7)
Day 57				
n[a]	438	242	33	15
GMC	1198.4	969.4	1476.7	586.7
(95% CI)[b]	(1104.3-1300.4)	(873.6-1075.8)	(1160.9-1878.4)	(363.3-947.4)
N1	438	242	33	15
GMFR (vs pre-vaccination GMC)[c]	123.9	111.6	160.7	54.9
(95% CI)[b]	(112.3-136.6)	(99.1-125.7)	(119.6-216.0)	(31.5-95.7)
SRR % from pre-vaccination[d] (n/N1)[e]	98.2 (430/438)	100.0 (242/242)	100.0 (33/33)	100.0 (15/15)
(95% CI)[b]	(96.4-99.2)	(98.5-100.0)	(89.4-100.0)	(78.2-100.0)
Day 209				
n[a]	403	238	30	13
GMC	266.5	195.3	301.2	163.8
(95% CI)[b]	(246.5-288.2)	(172.9-220.5)	(207.7-436.8)	(52.9-507.2)
N1	402	237	30	13
GMFR (vs pre-vaccination GMC)[c]	27.8	22.6	31.7	15.6
(95% CI)[b]	(25.2-30.6)	(19.7-26.0)	(19.7-50.9)	(4.4-55.5)
SRR, % from pre-vaccination (n/N1)[e]	97.3 (391/402)	93.7 (222/237)	100.0 (30/30)	69.2 (9/13)
(95% CI)[b]	(95.2-98.6)	(89.8-96.4)	(88.4-100.0)	(38.6-90.9)
Pre-booster				
n[a]	423	237	27	13
GMC	147.5	108.8	6134.0	4740.3
(95% CI)[b]	(135.9, 160.0)	(93.7-126.4)	(2997.0-12554.3)	(1316.7-17065.3)
BD-29				
n [a]	403	234	27	13
GMC	7688.6	7802.4	20403.4	13533.2
(95% CI) [b]	(7111.4-8312.7)	(6901.0-8821.5)	(15151.2-27476.4)	(8177.1- 22397.6)
N1	399	233	26	12
GMFR (vs pre-booster GMC[c])	52.1	73.4	3.2	3.3
(95% CI)[b]	(47.5-57.2)	(63.6-84.8)	(1.9-5.5)	(1.1-9.5)
SRR, % from pre-booster[d] (n/N1)[e]	98.7 (394/399)	97.9 (228/233)	26.9 (7/26)	33.3 (4/12)
(95% CI)[f]	(97.1-99.6)	(95.1- 99.3)	(11.6-47.8)	(9.9-65.1)
BD-181				
n [a]	374	201	25	10
GMC	2935.2	2758.3	4812.4	4139.6
(95% CI) [b]	(2628.6-3277.5)	(2359.9-3224.1)	(3037.6-7624.0)	(1327.9-12904.6)
N1	364	197	21	9
GMFR (vs pre-booster GMC)[c]	19.9	24.3	0.6	1.0
(95% CI)[b]	(17.6-22.4)	(20.2-29.3)	(0.4-0.9)	(0.3-4.0)
SRR, % from pre-booster[d] (n/N1)[e]	93.4 (340/364)	93.9 (185/197)	4.8 (1/21)	11.1 (1/9)
(95% CI)[f]	(90.3-95.7)	(89.6-96.8)	(0.1-23.8)	(0.3-48.2)

BD-29 and BD-181 = booster doses on days 29 and 181, respectively; CI = confidence interval; GM = geometric mean; GMC = geometric mean concentration [AU/mL]; GMFR = geometric mean fold-rise, LLOQ = lower limit of quantification; N1 = Number of participants with non-missing data at pre-booster and the corresponding time point; PPIS=per protocol immunogenicity set; SRR = seroresponse rate; ULOQ = upper limit of quantification.
Antibody values reported as below the LLOQ (10) are replaced by 0.5 x LLOQ. Values greater than the ULOQ (281600) are replaced by the ULOQ if actual values are not available.
[a]Number of participants with non-missing data at the corresponding time point.
[b]95% CI is calculated based on the t-distribution of the log-transformed values or the difference in the log- transformed values for GM value and GMFR, respectively, then back transformed to the original scale for presentation.
[c]GMFR calculated versus baseline (pre-vaccination) or pre-booster.
[d]Seroresponse at a participant level is defined as a change from below the LLOQ at baseline (pre-vaccination) or pre-booster to ≥4 x LLOQ, or ≥4-fold rise if baseline or pre-booster is equal to or above the LLOQ. Percentages are based on n in footnote a.
[e]Number of participants meeting the criterion at the time point. Percentages are based on N1.
[f]95% CI is calculated using the Clopper-Pearson method.

Table S22. Pseudovirus Neutralizing Antibody Against Ancestral SARS-CoV-2 (D614G) from Pre-booster Baseline, Part C PPIS by SARS-CoV-2-status and Sex

	PPIS-negative		PPIS-positive	
	Male	Female	Male	Female
Time point	mRNA-1273 100 µg Primary series N=361	mRNA-1273 100 µg Primary Series N=321	mRNA-1273 100 µg Primary series N=28	mRNA-1273 100 µg Primary Series N=21
Pre-booster				
n[a]	351	309	22	18
GMC	129.2	135.8	4790.5	6888.3
(95% CI)[b]	(115.3-144.7)	(123.1-149.8)	(1913.9-11990.8)	(2973.9-15955.0)
BD-29				
n [a]	339	298	23	17
GMC	7697.5	7767.6	17684.9	18087.3
(95% CI) [b]	(7015.9-8445.4)	(7053.7-8553.8)	(12587.4-24846.7)	(11825.2-27665.4)
N1	337	295	21	17
GMFR (vs pre-booster GMC)	60.6	57.5	4.0	2.5
(95% CI)[b]	(54.4-67.6)	(51.1-64.6)	(1.9-8.3)	(1.4-4.4)
SRR, % from pre-booster[d] (n/N1)[e]	98.5 (332/337)	98.3 (290/295)	38.1 (8/21)	17.6 (3/17)
(95% CI)[f]	(96.6-99.5)	(96.1-99.4)	(18.1-61.6)	(3.8- 43.4)
BD-181				
n [a]	305	270	17	18
GMC	3012.6	2721.3	4296.9	4926.0
(95% CI) [b]	(2649.2-3425.8)	(2400.8-3084.6)	(2311.1-7988.9)	(2565.0-9460.5)
N1	298	263	14	16
GMFR (vs pre-booster GMC)	22.5	20.1	0.9	0.6
(95% CI)[b]	(19.4-26.1)	(17.4-23.1)	(0.4-2.5)	(0.4-0.8)
SRR, % from pre-booster[d] (n/N1)[e]	91.9 (274/298)	95.4 (251/263)	14.3 (2/14)	(0/16)
(95% CI)[f]	(88.3-94.8)	(92.2-97.6)	(1.8-42.8)	(0.0-20.6)

BD-29 and BD-181 = booster doses on days 29 and 181, respectively; CI = confidence interval; GM = geometric mean; GMC = geometric mean concentration [AU/mL]; GMFR = geometric mean fold-rise; LLOQ = lower limit of quantification; N1 = Number of participants with non-missing data at pre-booster and the corresponding time point; PPIS=per protocol immunogenicity set; SRR = seroresponse rate; ULOQ = upper limit of quantification.
Antibody values reported as below the LLOQ (10) are replaced by 0.5 x LLOQ. Values greater than the ULOQ (281600) are replaced by the ULOQ if actual values are not available.
[a]Number of participants with non-missing data at the corresponding time point.
[b]95% CI is calculated based on the t-distribution of the log-transformed values or the difference in the log- transformed values for GM value and GM fold-rise, respectively, then back transformed to the original scale for presentation.
[c]Percentages are based on n in footnote a.
[d]Seroresponse at a participant level is defined as a change from below the LLOQ to ≥ 4 x LLOQ, or at least a 4-fold rise if pre-booster is equal to or above the LLOQ.
[e]Number of participants meeting the criterion at the time point. Percentages are based on N1.
[f]95% CI is calculated using the Clopper-Pearson method.

Table S23. Spike Binding Antibody Response Against Ancestral SARS-CoV-2 (D614G) from Pre-vaccination Baseline, Part C PPIS by SARS-CoV-2 Status

Time point	PPIS mRNA-1273 100 µg Primary series N=731	PPIS-negative mRNA-1273 100 µg Primary Series (N=682)	PPIS-positive mRNA-1273 100 µg Primary Series (N=49)
Baseline (pre-vaccination)			
n[a]	726	678	48
GM levels	34.6	34.6	35.9
95% CI [b]	(31.8-37.7)	(31.7-37.7)	(25.4-50.7)
Day 57			
n[a]	726	678	48
GM levels	217200.5	219312.3	189454.8
95% CI[b]	(196835.0-239673.1)	(198323.9-242522.0)	(119600.0-300109.7)
N1	724	676	48
GMFR (vs pre-vaccination GM level)	6263.3	6339.8	5280.1
95% CI[b]	(5524.8-7100.6)	(5572.5-7212.6)	(3054.1-9128.3)
SRR, % from pre-vaccination[d] (n/N1)[e]	98.5 (713/724)	98.5 (666/676)	97.9 (47/48)
95% CI[f]	(97.3-99.2)	97.3-99.3	88.9-99.9
Day 209			
n [a]	683	640	43
GM levels	42319.4	42176.3	44506.8
95% CI [b]	(39993.0-44781.1)	(39850.944637.4)	(32360.9-61211.4)
N1	679	636	43
GMFR (vs pre-vaccination GM level)	1227.6	1231.0	1178.7
95% CI[b]	(1108.0-1360.2)	(1107.7-1368.0)	(754.0-1842.8)
SRR, % from pre-vaccination[d] (n/N1)[e]	99.9 (678/679)	99.8 (635/636)	100 (43/43)
95% CI[f]	(99.2-100.0)	(99.1-100.0)	(91.8-100.0)
Prebooster			
n a	704	663	41
GM levels	21301.3	17661.4	440945.0
95% CI [b]	(19278.6-23536.3)	(16271.8-19169.7)	(258855.3-751124.3)
N1	699	659	40
GMFR (vs pre-vaccination GM level)	629.9	524.5	12864.2
95% CI[b]	(555.8-713.8)	(468.3-587.4)	(6832.9-24219.1)
SRR, % from pre-vaccination[d] (n/N1)[e]	99.3 (694/699)	99.2 (654/659)	100 (40/40)
95% CI[f]	(98.3-99.8)	(98.2-99.8)	(91.2-100.0)
BD-29			
n [a]	678	638	40
GM levels	954301.7	931634.7	1400244.2
95% CI [b]	(881043.6-1033651.0)	(857434.7-1012255.8)	(1071319.1-1830158.8)
N1	673	634	39
GMFR (vs pre-vaccination GM level)	28143.8	27478.2	41529.2
95% CI [b]	(24961.4-31731.9)	(24262.9-31119.6)	(26656.7-64699.5)
SRR, % from pre-vaccination[d] (n/N1)[e]	99.7 (671/673)	99.7 (632/634)	100.0 (39/39)
95% CI[f]	(98.9-100.0)	(98.9-100.0)	(91.0-100.0)
BD-181			
n [a]	608	574	34
GM levels	438319.9	439401.6	420455.3
95% CI [b]	(406730.3-472363.0)	(407232.3-474112.2)	(283026.9-624614.3)
N1	604	571	33
GMFR (vs pre-vaccination GM level)	12357.3	12534.2	9662.2
95% CI [b]	(10912.0-13994.0)	(11030.5-14243.0)	(5500.6-16972.3)
SRR, % from pre-vaccination[d] (n/N1)[e]	99.8 (603/604)	99.8 (570/571)	100 (33/33)
95% CI[f]	(99.1-100.0)	(99.0-100.0)	(89.4-100.0)

BD-29 and BD-181 = booster doses on days 29 and 181, respectively; CI = confidence interval, GM = geometric mean; GMFR = geometric mean fold-rise; LLOQ = lower limit of quantification; N1 = number of participants with non-missing data at baseline and the corresponding time point; SRR = seroresponse rate; ULOQ = upper limit of quantification. Antibody values reported as below the LLOQ are replaced by 0.5 x LLOQ. Values greater than the ULOQ are replaced by the ULOQ if actual values are not available.
[a]Number of participants with non-missing data at the corresponding time point.
[b]95% CI is calculated based on the t-distribution of the log-transformed values or the difference in the log- transformed values for GM value and GMFR, respectively, then back transformed to the original scale for presentation.
[c]Percentages are based on n in footnote a.
[d]Seroresponse at a participant level is defined as a change from below the LLOQ to ≥ 4 x LLOQ, or at least a 4-fold rise if baseline is equal to or above the LLOQ.
[e]Number of participants meeting the criterion at the time point. Percentages are based on N1.
[f]95% CI is calculated using the Clopper-Pearson method.

Table S24. Spike Binding Antibody Response Against Ancestral SARS-CoV-2 (D614G) from Pre-booster Baseline Part C Per-Protocol Immunogenicity Subset

	PPIS	PPIS-negative	PPIS-positive
Time point	mRNA-1273 100 µg Primary series N=731	mRNA-1273 100 µg Primary Series (N=682)	mRNA-1273 100 µg Primary Series (N=49)
Pre-booster			
n[a]	704	663	41
GM levels	21301.3	17661.4	440945.0
95% CI[b]	(19278.6-23536.3)	(16271.8-19169.7)	(258855.3-751124.3)
BD-29			
n[a]	677	637	40
GM levels	952877.4	930122.0	1400244.2
95% CI[b]	(879672.1-1032174.8)	(855983.3-1010682.1)	(1071319.1-1830158.8)
N1	672	633	39
GMFR (vs pre-booster GM level)	45.6	53.5	3.3
95% CI[b]	(40.8-50.9)	(48.4-59.3)	(2.2-4.9)
SRR, % from pre-booster[d] (n/N1)[e]	93.9 (631/672)	97.8 (619/633)	30.8 (12/39)
95% CI[f]	(91.8-95.6)	(96.3-98.8)	(17.0-47.6)
BD-181			
n[a]	608	574	34
GM levels	438319.9	439401.6	420455.3
95% CI[b]	(406730.3-472363.0)	(407232.3-474112.2)	(283026.9-624614.3)
N1	589	560	29
GMFR (vs pre-booster GM level)	21.0	24.6	1.0
95% CI[b]	(18.7-23.5)	(22.2-27.2)	(0.6-1.5)
SRR, % from pre-booster[d] (n/N1)[e]	90.8 (535/589)	95.2 (533/560)	6.9 (2/29)
95% CI[f]	(88.2-93.0)	(93.1-96.8)	(0.8-22.8)

BD-29 and BD-181 = booster doses on days 29 and 181, respectively; CI = confidence interval, GM = geometric mean; GMFR = geometric mean fold-rise; LLOQ = lower limit of quantification; N1 = number of participants with non-missing data at baseline and the corresponding time point; SRR = seroresponse rate; ULOQ = upper limit of quantification.
Antibody values reported as below the LLOQ are replaced by 0.5 x LLOQ. Values greater than the ULOQ are replaced by the ULOQ if actual values are not available.
[a]Number of participants with non-missing data at the corresponding time point.
[b]95% CI is calculated based on the t-distribution of the log-transformed values or the difference in the log- transformed values for GM level and GMFR, respectively, then back transformed to the original scale for presentation.
[c]Percentages are based on n in footnote a.
[d]Seroresponse at a participant level is defined as a change from below the LLOQ to ≥ 4 x LLOQ, or at least a 4-fold rise if baseline is equal to or above the LLOQ.
[e]Number of participants meeting the criterion at the time point. Percentages are based on N1.
[f]95% CI is calculated using the Clopper-Pearson method.

Table S25. Characteristics of Participants Included in the Exploratory Analysis of Time Intervals

	mRNA-1273 PPIS-Negative Subset* N=682	Placebo-mRNA-1273† N=80
**Age at Screening**		
Mean	55.3 (14.9)	56.9 (14.4)
Median	58.0	58.0
Min, Max	18-86	19-84
**Sex**		
Female	321 (47)	40 (50)
Male	361 (53)	40 (50)
**Ethnicity**		
Hispanic or Latino	207 (30)	23 (29)
Not Hispanic or Latino	472 (69)	57 (71)
Not reported/unknown	3 (0.4)	0

Baseline characteristics in subset of placebo-mRNA-1273 participants from the randomly selected per protocol immunogenicity set.

*Exploratory analysis of a pre-defined subset of PPIS-negative participants.

†N=80 participants who are pre-booster negative with non-missing intervals and non-missing nAb results at booster dose day 1 and are not included in a predefined immunogenicity subset.

Table S26. Baseline Demographics and Characteristics of Participants in Exploratory Effectiveness Analysis by Booster Timing

Characteristics n (%)	Early boosted* (N=8298)	Late boosted* (N=8727)
Age at screening (years)		
Mean (range)	54.5 (18-94)	51.8 (18-95)
Age and health risk for severe COVID-19, n (%)[a]		
≥18 and <65 years and not at risk	4237 (51.1)	4987 (57.1)
≥18 and <65 years and at risk	1344 (16.2)	1543 (17.7)
≥65 years	2717 (32.7)	2197 (25.2)
Sex, n (%)		
Male	4331 (52.2)	4563 (52.3)
Female	3967 (47.8)	4164 (47.7)
Race, n (%)		
White	6730 (81.1)	6734 (77.2)
Black or African American	761 (9.2)	989 (11.3)
Asian	324 (3.9)	407 (4.7)
American Indian or Alaska Native	65 (0.8)	67 (0.8)
Native Hawaiian or other Pacific Islander	19 (0.2)	20 (0.2)
Multiracial	179 (2.2)	215 (2.5)
Other	148 (1.8)	193 (2.2)
Not reported or unknown	72 (0.9)	102 (1.2)
Ethnicity, n (%)		
Hispanic or Latino	1320 (15.9)	1973 (22.6)
Not Hispanic or Latino	6885 (83.0)	6681 (76.6)
Not reported or unknown	93 (1.1)	73 (0.8)
Risk factor for severe COVID-19 at screening, n (%)[b]		
At risk	1994 (24.0)	2106 (24.1)
Chronic lung disease	408 (4.9)	467 (5.4)
Significant cardiac disease	476 (5.7)	448 (5.1)
Severe obesity	570 (6.9)	653 (7.5)
Diabetes	837 (10.1)	926 (10.6)
Liver disease	55 (0.7)	70 (0.8)
Human immunodeficiency virus infection	59 (0.7)	52 (0.6)
Occupational Risk	6651 (80.2)	6886 (78.9)
Healthcare Workers	1894 (22.8)	1722 (19.7)
Time Between second injection Primary Series to Booster		
Median days (IQR)	357 (240-384)	347 (265-411)
Follow-Up Time on Study from Booster (Days)		
Median days (IQR)	175 (169-182)	147 (127-156)
Baseline SARS-CoV-2 status, n (%)[c]		
Negative	8298 (100)	8727 (100)
Positive	0	0
Missing	0	0
Pre-booster SARS-CoV-2 status, n (%)		
Negative	8088 (97.5)	8163 (93.5)
Positive	210 (2.5)	564 (6.5)
Missing	0	0
Body Mass Index, kg/m^2		
n	8232	8671
Mean (SD)	29.3 (6.7)	29.4 (6.8)
Standard risk score		
n	8229	8137
Mean (SD)	-0.11 (1.01)	-0.03 (1.01)
Unblind		
Early	4863 (58.6)	3930 (45.0)
Late	3435 (41.4)	4797 (55.0)

*Early boosted participants received booster before 25Oct2021 (median of booster dates) and late boosted participants received booster on or later than October 25, 2021 (median of booster dates). IQR-interquartile range; IRT = interactive response technology; RT-PCR = reverse transcription polymerase chain reaction; PDV = participant decision visit. Percentages are based on the number of participants in the analysis set in the COVE trial who remained in follow-up as of 23 September 2021 with no evidence of prior SARS-CoV-2 infection.

[a]Based on stratification factor from IRT, participants who are <65 years old are categorized as at risk for severe COVID-19 illness if they have at least 1 of the risk factors specified in the study protocol at Screening.

[b]Participants could be under ≥1 categories and are counted once at each category.

[c]Baseline SARS-CoV-2 status: Positive if there is immunologic or virologic evidence of prior COVID-19, defined as positive RT-PCR test or positive Elecsys result at day 1. Negative is defined as negative RT-PCR test and negative Elecsys result at day 1.

Table S27. Hazard Ratios and 95% Confidence Intervals for Delta and Omicron Cases by Boost Period

Variant	Arm	Pre-boost HR	Post-Boost HR	1-post/pre-boost HR (Boost Effectiveness)* (95% CI)
Delta	mRNA-1273	1.24	0.17	0.86 (0.76-0.92)
	Placebo-mRNA-1273	1.00	0.20	0.80 (0.65-0.89)
	Relative effectiveness ratio (95% CI)†	1.24 (0.91-1.70)	0.86 (0.44-1.67)	
Omicron	mRNA-1273	0.91	0.51	0.44 (0.31-0.55)
	Placebo-mRNA-1273	1.00	0.68	0.32 (0.15-0.46)
	Relative effectiveness ratio (95% CI)†	0.91 (0.69-1.22)	0.76 (0.68-0.84)	

HR-hazard ratio. *Ratio of post-booster/prebooster; †Ratio of mRNA-1273/placebo-mRNA-1273. The boosting effectiveness on COVID-19 risk was assessed in Cox models as described in the Methods and Supplementary Methods, by randomized study vaccination groups and boost period using the covariate (sex, stratum, risk score, early unblinding) adjustment for potential confounders.

Table S28. Analysis Sets Definitions

**Primary Series**	Participants randomized to mRNA-1273 in Part A and those who crossed over to mRNA-1273 in Part B
Per-protocol primary series set	The per-protocol primary series set consists of all participants in per-protocol set who received two injections of mRNA-1273 primary series per schedule in Part A or Part B, with negative pre-injection 1 SARS-CoV-2 status and had no major protocol deviations that impact critical or key study data.<br>Participants who did not receive the treatment as randomized in Part A, did not receive 2 doses of the primary series of mRNA-1273, or did not receive Dose 2 of mRNA-1273 within [21, 42] days after Dose 1 of mRNA-1273 will be excluded from the per-protocol primary series set.<br>PP primary series set will be used for the effectiveness analysis of the mRNA-1273 primary series. Per-protocol primary series set will also be used to assess effectiveness of participants received booster dose and those who did not receive booster dose by pre-booster SARS-CoV-2 status.
Primary series safety set	All participants who were randomized in Part A and received at least one dose of mRNA-1273 primary series. The primary series safety set will be used for the safety analysis of the mRNA-1273 primary series.
**Part C**	
Part C safety set	Participants who were randomized in Part A and received mRNA-1273 50 µg booster in Part C. The Part C safety set will be used for all analysis of safety in Part C.
Part C per-protocol set	All participants who were randomized in Part A, received mRNA-1273 50 µg booster in Part C after primary series per schedule, pre-booster SARS-CoV-2 negative, and have no major protocol deviations that impact critical or key data.<br>Participants who did not receive the treatment as randomized in Part A, did not receive 2 doses of the primary series of mRNA-1273, or did not receive Dose 2 of mRNA-1273 within [21, 42] days after Dose 1 will be excluded from the Part C per-protocol set.<br>Part C PP set will be used for analysis of effectiveness of booster.
Part C Per-protocol immunogenicity subset	Participants in the per-protocol random subset for immunogenicity (PPRSI) in Part A who were randomized to mRNA-1273 in Part A, were baseline/pre-Day 1 SARS-CoV-2 negative and received booster in Part C. Part C PPIS is a subset of PPRSI in Part A, and a subset of Part C PPRSI. Part C PPIS will be used to summarize immune response after booster in Part C
Part C per-protocol immunogenicity subset – pre-booster SARS-CoV-2 negative (Part C PPIS-negative)	Participants who are in Part C PPIS and are pre-booster SARS-CoV-2 negative, defined as no virologic or serologic evidence of SARS-CoV-2 infection on or before booster dose (BD)-Day 1 (pre-booster), i.e. RT-PCR result is not positive if available at BD-1 and negative for binding antibodies specific to SARS-CoV-2 nucleocapsid (as measured by Roche Elecsys Anti-SARS-CoV-2 assay) on or before BD-1.<br>Part C PPIS-negative will be used for analysis of immune response to booster, especially, to be compared with immune response after primary series of mRNA-1273 to infer effectiveness of a booster.

Table S29. Analysis Periods for Analyses of Part B, Part C, and Primary Series

Treatment Group/Cohort	Description	Analysis Period
**Part B (**Effectiveness**)**		
mRNA-1273	Participants randomized to mRNA-1273 in the Blinded Phase<br>Date of reference: PDV/unblinding	From PDV/unblinding to earlier date of (BD-1 visit, DBL or data-cutoff date for effectiveness, last observed in study[a])
Placebo	Participants randomized to Placebo in the Blinded Phase and did not request to receive mRNA-1273 in Part B. Date of reference: Dose 1 of mRNA-1273	
Placebo-mRNA-1273	Participants randomized to Placebo in the Blinded phase and requested to receive mRNA-1273 in Part B<br>Date of reference: Dose 1 of mRNA-1273 in Part B	
**Part B (Safety)**		
mRNA-1273	Participants received at least one dose of mRNA-1273 in the Blinded Phase	From PDV/unblinding or 1st dose of mRNA-1273 in Part B to earlier date of (BD- Day 1, DBL or data-cutoff date for safety, last observed in study[a]).
Placebo	Participants only received Placebo in the Blinded Phase and did not receive mRNA-1273 in Part B	
Placebo-mRNA-1273	Participants only received Placebo in the Blinded phase and received mRNA-1273 in Part B	
**Part C (E**ffectiveness**, Immunogenicity, Safety)**		
Booster 50 µg after mRNA-1273 primary series	Participants randomized to/received mRNA-1273 in the Blinded Phase and received booster 50 µg in Part C<br>Date of reference: date of booster	From dose date in Part C to earlier date of (DBL or data-cutoff date for safety, last observed in study [a]).
Booster 50 µg after Placebo-mRNA-1273 Primary Series	Participants randomized to /only received Placebo in the Blinded Phase and received at least one dose of mRNA-1273 in Part B and received booster 50 µg in Part C<br>Date of reference: date of booster	
Booster 50 µg	Participants randomized in the blinded phase and received booster 50 µg in Part C<br>Date of reference: date of booster	From dose date in Part C to earlier date of (DBL or data-cutoff date for safety, last observed in study [a]).
Not received booster	Participants randomized to/received mRNA-1273 in Part	Up to earlier date of (DBL or data-cutoff date for safety, last observed in study [a]).

	A or B and did not receive booster in Part C<br>Date of reference: date of BD-visit 1	
Placebo	Participants randomized to/only received Placebo in Part A or B and did not receive booster in Part C<br>Date of reference: date of BD-visit 1	From BD-visit 1 in Part C to earlier date of (DBL or data-cutoff date for safety, last observed in study [a]).
**Effectiveness, Immunogenicity of mRNA-1273 primary Series**		
mRNA-1273	Participants randomized to mRNA-1273 in the Blinded Phase	From Dose 1 of mRNA-1273 to earlier date of (booster date, DBL or data-cutoff date, last observed in study [a]).
Placebo-mRNA-1273	Participants randomized to Placebo and received mRNA-1273 in Part B	
**Safety of mRNA-1273 primary Series**		
mRNA-1273	Participants received mRNA-1273 in the Blinded Phase	From Dose 1 of mRNA-1273 to earlier date of (booster date, DBL or data-cutoff date, last observed in study [a]).
Placebo-mRNA-1273	Participants received Placebo in the Blinded phase and received mRNA-1273 in Part B	

[a]Last observed in study is the earliest date of study discontinuation, study completion, or death.

All analyses were conducted using SAS Version 9.4 or higher unless otherwise specified.

Table S30. Participants in Analysis Sets

A. Part B

	Placebo	Placebo-mRNA-1273	mRNA-1273	Total
Randomization set*	2556	12650	15209	30415
Full analysis set, n (%)*	2516 (98.4)	12650 (100)	15180 (99.8)	30346 (99.8)
Modified intent-to-treat set, n (%)*	2415 (94.5)	12335 (97.5)	14756 (97.0)	29506 (97.0)
Per-protocol set, n (%)*	2103 (82.3)	12065 (95.4)	14295 (94.0)	28463 (93.6)
Safety set†	2513	12649	15184	30346

Analysis sets refer to the analysis sets in the blinded phase.
*Numbers are based on planned treatment group and percentages are based on the number of randomized participants
†Numbers are based on actual treatment group.

B. Part C

	Part C Booster 50 µg mRNA-1273		
	mRNA-1273 100 µg Primary Series	Placebo-mRNA-1273 100 µg Primary Series	Total
Part C safety set	9647	9952	19609
Part C per-protocol set	8500	7868	16368

Numbers are based on actual treatment group.

Table S31. Criteria for mRNA-1273 Booster (Part C) as Compared With mRNA-1273 Primary Series (Part A) Superiority/Non-Inferiority Analysis

Endpoint	Scenario	Conclusion
GMR	Lower bound of 95% CI of GMR < 0.67	Non-inferiority of a booster as compared with mRNA-1273 primary series based on GMR is not demonstrated
	0.67 ≤ Lower bound of 95% CI of GMR ≤ 1	Non-inferiority of a booster as compared with mRNA-1273 primary series based on GMR is demonstrated
	Lower bound of 95% CI of GMR > 1	Superiority of a booster as compared with mRNA-1273 primary series based on GMR is demonstrated
SRR	Lower bound of 95% CI of SRR difference < -10%	Non-inferiority of a booster as compared with mRNA-1273 primary series based on SRR difference is not demonstrated
	-10% ≤ Lower bound of 95% CI of SRR difference ≤ 0	Non-inferiority of a booster as compared with mRNA-1273 primary series based on SRR difference is demonstrated but superiority is not demonstrated

GMR = geometric mean ratio; SRR = seroresponse.

Supplement References

1. CDC. Symptoms of COVID-19. Vol. 2021 (2021).
2. Baden, L.R.*, et al.* Efficacy and Safety of the mRNA-1273 SARS-CoV-2 Vaccine. *N Engl J Med* **384**, 403-416 (2021).
3. El Sahly, H.M.*, et al.* Efficacy of the mRNA-1273 SARS-CoV-2 Vaccine at Completion of Blinded Phase. *N Engl J Med* **385**, 1774-1785 (2021).
4. US FDA. Development and Licensure of Vaccines to Prevent COVID-19: Guidance for Industry. (https://www.fda.gov/media/139638/download, 2023).
5. El Sahly, H.M.*, et al.* Humoral Immunogenicity of the mRNA-1273 Vaccine in the Phase 3 Coronavirus Efficacy (COVE) Trial. *J Infect Dis* **226**, 1731-1742 (2022).
6. Follmann, D.*, et al. 1939. Who to Boost When: An Analysis of Dosing Interval and Age on COVID-19 Outcomes in the COVE Trial During the Delta and Omicron Waves*, (Open Forum Infect Dis. 2023 Nov 27;10(Suppl 2):ofad500.2470. doi: 10.1093/ofid/ofad500.2470. eCollection 2023 Dec., 2023).
7. Bonhomme, M.E.*, et al.* Robust validation and performance comparison of immunogenicity assays assessing IgG and neutralizing antibodies to SARS-CoV-2. *PLoS One* **17**, e0262922 (2022).
8. PPD Laboratories. PPD Laboratories Assays for COVID-19 Trials.
9. Gilbert, P.B.*, et al.* Immune correlates analysis of the mRNA-1273 COVID-19 vaccine efficacy clinical trial. *Science* **375**, 43-50 (2022).

CLINICAL STUDY PROTOCOL

Protocol Title:	A Phase 3, Randomized, Stratified, Observer-Blind, Placebo-Controlled Study to Evaluate the Efficacy, Safety, and Immunogenicity of mRNA-1273 SARS-CoV-2 Vaccine in Adults Aged 18 Years and Older
Protocol Number:	mRNA-1273-P301
Sponsor Name:	ModernaTX, Inc.
Legal Registered Address:	200 Technology Square<br>Cambridge, MA 02139
Sponsor Contact and Medical Monitor:	Rituparna Das MD, PhD<br>VP, Clinical Development - COVID-19 Vaccines<br>ModernaTX, Inc.<br>200 Technology Square, Cambridge, MA 02139<br>Telephone: 1-617-710-9794<br>e-mail: rituparna.das@modernatx.com
Regulatory Agency Identifier Number(s):	IND: 19745
Amendment Number:	10
Date of Amendment 10:	07 Apr 2022
Date of Amendment 9:	10 Sep 2021
Date of Amendment 8:	23 Mar 2021
Date of Amendment 7:	10 Feb 2021
Date of Amendment 6:	23 Dec 2020
Date of Amendment 5:	11 Nov 2020
Date of Amendment 4:	30 Sep 2020
Date of Amendment 3:	20 Aug 2020
Date of Amendment 2:	31 Jul 2020
Date of Amendment 1:	26 Jun 2020
Date of Original Protocol:	15 Jun 2020

CONFIDENTIAL

All financial and nonfinancial support for this study will be provided by ModernaTX, Inc. The concepts and information contained in this document or generated during the study are considered proprietary and may not be disclosed in whole or in part without the expressed written consent of ModernaTX, Inc. The study will be conducted according to the *International Council for Harmonisation (ICH) Technical Requirements for Registration of Pharmaceuticals for Human Use*, *E6(R2) Good Clinical Practice (GCP) Guidance.*

Protocol Approval – Sponsor Signatory

**Study Title:**	A Phase 3, Randomized, Stratified, Observer-Blind, Placebo-Controlled Study to Evaluate the Efficacy, Safety, and Immunogenicity of mRNA-1273 SARS-CoV-2 Vaccine in Adults Aged 18 Years and Older
**Protocol Number:**	mRNA-1273-P301
**Protocol Version Date:**	07 Apr 2022

Protocol accepted and approved by:

Please see eSignature and date in the last page of the document.

______________________________	____________________
Rituparna Das MD, PhD	Date

VP, Clinical Development –
COVID-19 Vaccines
ModernaTX, Inc.
200 Technology Square
Cambridge, MA 02139
Telephone: 1-617-710-9794

DECLARATION OF INVESTIGATOR

I have read and understood all sections of the protocol entitled "A Phase 3, Randomized, Stratified, Observer-Blind, Placebo-Controlled Study to Evaluate the Efficacy, Safety, and Immunogenicity of mRNA-1273 SARS-CoV-2 Vaccine in Adults Aged 18 Years and Older" and the most recent version of the Investigator's Brochure.

I agree to supervise all aspects of the protocol and to conduct the clinical investigation in accordance with the current protocol, the *International Council for Harmonisation (ICH) Technical Requirements for Registration of Pharmaceuticals for Human Use, E6(R2) Good Clinical Practice (GCP) Guidance*, and all applicable government regulations. I will not make changes to the protocol before consulting with ModernaTX, Inc. or implement protocol changes without institutional review board (IRB)/independent ethics committee (IEC) approval except to eliminate an immediate risk to participants.

I agree to administer study treatment only to participants under my personal supervision or the supervision of a sub-investigator. I will not supply study treatment to any person not authorized to receive it. I also agree that persons debarred from conducting or working on clinical studies by any court or regulatory agency will not be allowed to conduct or work on studies for the Sponsor or a partnership in which the Sponsor is involved. I will immediately disclose it in writing to the Sponsor if any person who is involved in the study is debarred, or if any proceeding for debarment is pending, or, to the best of my knowledge, threatened.

I will not disclose confidential information contained in this document including participant information, to anyone other than the recipient, study staffs, and members of the IRB/IEC. I agree to ensure that this information will not be used for any purpose other than the evaluation or conduct of the clinical investigation without the prior written consent from ModernaTX, Inc. I will not disclose information regarding this clinical investigation or publish results of the investigation without authorization from ModernaTX, Inc.

The signature below provides the necessary assurance that this study will be conducted according to all stipulations of the protocol, including statements regarding confidentiality, and according to local legal and regulatory requirements, US federal regulations, and ICH E6(R2) GCP guidelines.

__________________________________ ____________________

Signature of Principal Investigator Date

____

Printed Name of Principal Investigator

Protocol Amendment Summary of Changes

DOCUMENT HISTORY	
**Document**	**Date**
Amendment 10	07 Apr 2022
Amendment 9	10 Sep 2021
Amendment 8	23 Mar 2021
Amendment 7	10 Feb 2021
Amendment 6	23 Dec 2020
Amendment 5	11 Nov 2020
Amendment 4	30 Sep 2020
Amendment 3	20 Aug 2020
Amendment 2	31 Jul 2020
Amendment 1	26 Jun 2020
Original Protocol	15 Jun 2020

Amendment 10, 07 Apr 2022: Current Amendment

This amendment is considered to be substantial based on the criteria set forth in Article 10(a) of Directive 2001/20/EC of the European Parliament and the Council of the European Union.

Main Rationale for the Amendment:

1. To provide guidance to the investigators in providing a booster dose to participants with breakthrough coronavirus disease 2019 infection after primary series.
2. To include immunogenicity analysis for Part C as a secondary objective of the study.

Summary of Major Changes From Protocol Amendment 9 to Protocol Amendment 10:

Section # and Name	Description of Change	Brief Rationale
Title Page, Protocol Approval Page, Headers, Protocol Amendment Summary of Changes	Updated the protocol version and date.	To reflect the new version and date of the protocol.
Title Page, Protocol Approval Page	Updated Sponsor contact.	To reflect personnel change.
Section 1.2 (Schema)	Updated Figure 1 and Figure 2.	To reflect Part C as described elsewhere in the current protocol.

Section # and Name	Description of Change	Brief Rationale
Section 1.1 (Synopsis), Section 2.3.2 (Risks from Study Participation), Section 2.3.3 (Overall Benefit/Risk Conclusion), Section 4.1.3 (Part C, the Booster Dose Phase)	Text updated to reflect the current benefit-risk profile of a booster dose of mRNA-1273 based on cumulative evidence.	Additional evidence informing the benefit-risk profile of booster dose from the ongoing Moderna Phase 2a study (mRNA-1273-P201) has become available since the previous amendment.
Section 1.1 (Synopsis), Section 2.3.2 (Risks from Study Participation), Section 4.1.3 (Part C, the Booster Dose Phase), Section 4.2 (Scientific Rationale for Study Design)	Updated sections to generally reference variants rather than a specific variant.	To encompass a broad range of emerging variants.
Section 1.1 (Synopsis), Section 3 (Objectives and Endpoints), Section 9.5.3 (Immunogenicity Analyses)	For Part C, immunogenicity analysis of BD vaccine response will be performed using the noninferiority tests of the 2 null hypotheses based on the 2 key secondary endpoints: Ab GMT and seroresponse rate at BD-Day 29 in Part C compared with Ab GMT and seroresponse rate at Day 57 (28 days after the second dose of the primary series of mRNA-1273).	To provide immunogenicity analysis plan relevant to the provision of a booster dose in Part C.
Section 1.1 (Synopsis), Section 4.1.1 (Part A, the Blinded Phase), Section 8.1.3 (Convalescent Period Starting with the Illness Visit), Section 11.1 (Appendix 1: Schedules of Events, Table 19)	Clarified that the duration of the daily telemedicine visits may extend past the 28-day Convalescent Period.	The Convalescent Period duration as presented in the Schedule of Events is 28 days; however, text was added to clarify that telemedicine visits may occur outside of this window depending on the duration of symptoms.

Section # and Name	Description of Change	Brief Rationale
Section 1.1 (Synopsis), Section 4.1.1 (Part A, the Blinded Phase), Section 6.4.2 (Concomitant Medications and Therapies)	Occasional references to parts of the study (eg, "Part A only") in regard to particular procedures, requirements, or prohibitions have been removed from protocol sections that are specific to the study part and/or where these apply to all parts of the study.	To remove redundancies that did not improve clarity.
Section 1.1 (Synopsis) Section 4.1.2 (Part B, the Open-label Observational Phase), Section 4.4 (End of Study Definition), Section 7.2 (Discontinuation of Study Treatment)	Clarified that a participant who did not receive a second dose of IP will have a study follow-up of 25 months after first dose of IP.	To address participants who may not have had the second dose of their original IP assignment following randomization.
Section 1.1 (Synopsis), Section 8.2 (Safety Assessments), Section 8.3.3 (Adverse Events of Special Interest), Section 9.5.2.1 (Adverse Events), Section 11.1 (Appendix 1: (Schedules of Events)	Added AESIs as an assessment for Part C (for participants who receive the BD). Removed reference to AESI in Schedule of Events tables specific for Part A and Part B.	To clarify that AESIs will be collected in Part C for participants who receive the BD.
Section 1.1 (Synopsis), Section 9.5.2.1 (Adverse Events)	Clarified the analysis sets used for safety analyses.	To clarify which analysis set will be used for specific parts of the study.
Section 4.4 (End of Study Definition)	Clarified the timing of the final visit.	To clarify when the final study visit will occur for participants who missed the second dose and for participants who entered Part C and had a BD-3 visit later than Day 759 (Month 25).
Section 5.1.2 (Exclusion Criteria)	Removed text specifying "Part A only" for exclusion criteria #4, #6, #11, #13, and #14; added text clarifying that exclusion criterion #10 is applicable to Part A only.	To account for the addition of Part B and Part C to the study, exclusion criteria were updated to reflect applicability of criteria to Part B and Part C.

Section # and Name	Description of Change	Brief Rationale
Section 6.3 (Study Treatment Compliance), Section 7.2 (Discontinuation of Study Treatment), Section 7.3.1 (Participant Withdrawal)	Added participants who are withdrawn from the study due to receipt of a non-study primary series or first booster COVID-19 vaccine in addition to participants who withdraw consent.	To clarify statements to cover both types of participant withdrawal.
Section 6.4.3 (Concomitant Medications and Vaccines That May Lead to the Elimination of a Participant from Per-protocol Analyses)	Clarified that any non-study COVID-19 may lead to exclusion from the per-protocol analysis in addition to withdrawal from the study (for primary series or first booster only).	To remain consistent with Section 7.3.1 (Participant Withdrawal).
Section 7.1 (Criteria for Delay of Study Treatment)	Additional criteria for delay of study treatment added for Part C, including seasonal influenza vaccine and diagnosed COVID-19.	To allow rescheduling of IP dosing in specified situations.
Section 8.1.3 (Convalescent Period Starting With the Illness Visit), Section 11.1 (Appendix 1: Schedules of Events, Table 19)	Text added to allow participants who have been asymptomatic for more than 72 hours prior to Day 14 to reduce the frequency of telemedicine calls during the convalescent period.	To provide a reduced telemedicine call burden for the sites and participants when certain criteria are met.
Section 9.5.2 (Safety Analyses, Table 12)	Added “Part A” to the table title.	To clarify that this section applies to Part A of the study.
Section 9.5.4 (Exploratory Analyses in Part A)	Added “in Part A” to the section title and removed section paragraph specific to Part A, as this is now specified in the title.	To clarify that this section applies to Part A of the study.
Section 11.1 (Appendix 1: Schedules of Events, Table 17 and Table 18)	Duration of eDiary collection was corrected to Day 759.	The eDiary collection duration was incorrectly listed in the previous version of the schedules of events.
Section 11.1 (Appendix 1: Schedules of Events, Table 18)	Window for safety calls was increased from ±3 days to ±7 days.	The safety call window was increased to allow flexibility for sites and participants.

Section # and Name	Description of Change	Brief Rationale
Section 11.1 (Appendix 1: Schedules of Events, Table 18)	Window for Day 759 Visit was changed from ±14 days to -14/+28 days.	The visit window was increased to allow flexibility for the sites and participants.
Section 11.1 (Appendix 1: Schedules of Events, Table 23)	Window for BD-3 Visit changed from -3/+14 to ±14 days.	The visit window was increased to allow flexibility for the sites and participants.
Section 11.1 (Appendix 1: Schedules of Events, Table 23)	Clarified that not all participants may receive the booster in Part C; added footnote 4 regarding sample collection details for blood for immunologic analysis; clarified that physical examinations and vital sign collection are optional for participants who chose not to receive a BD. Clarified that pregnancy tests at the BD-1 visit are for participants who receive a BD.	Text added for clarity.
Section 11.2.6 (Informed Consent Process)	Clarified that participants who are rescreened or rescheduled are not required to re-sign the same version of the ICF if occurring within 28 days from the previous ICF signature date.	Text added for clarity.
Section 11.5 (Appendix 5: Adverse Events of Special Interest Terms)	Appendix updated to clarify AESI terms.	Text edited for clarity.

IRB and Regulatory Authority Approval

A copy of this amended protocol will be sent to the IRB and regulatory authority.

The changes described in this amended protocol require IRB approval prior to implementation. In addition, if the changes herein affect the informed consent, sites are required to update and submit a revised informed consent for approval that incorporates the changes described in this amended protocol.

1. PROTOCOL SUMMARY

1.1. Synopsis

**Protocol Number:**	**mRNA-1273-P301**
**Title:**	A Phase 3, Randomized, Stratified, Observer-Blind, Placebo-Controlled Study to Evaluate the Efficacy, Safety, and Immunogenicity of mRNA-1273 SARS-CoV-2 Vaccine in Adults Aged 18 Years and Older.
**Study Phase:**	3
**Objectives:**	Primary:

Primary:

- To demonstrate the efficacy of mRNA-1273 to prevent coronavirus disease 2019 (COVID-19).
- To evaluate the safety and reactogenicity of 2 injections of mRNA-1273 given 28 days apart.

Secondary:

- To evaluate the efficacy of mRNA-1273 to prevent severe COVID-19.
- To evaluate the efficacy of mRNA-1273 to prevent serologically confirmed severe acute respiratory syndrome coronavirus 2 (SARS-CoV-2) infection or COVID-19 regardless of symptomatology or severity.
- To evaluate vaccine efficacy (VE) against a secondary definition of COVID-19.
- To evaluate VE to prevent death caused by COVID-19.
- To evaluate the efficacy of mRNA-1273 to prevent COVID-19 after the first dose of investigational product (IP).
- To evaluate the efficacy of mRNA-1273 to prevent COVID-19 in all study participants, regardless of evidence of prior SARS-CoV-2 infection.
- To evaluate the efficacy of mRNA-1273 to prevent asymptomatic SARS-CoV-2 infection.

	Exploratory for Part A:<br>• To evaluate the effect of mRNA-1273 on the viral infection kinetics as measured by viral load at SARS-CoV-2 infection diagnosis by reverse transcriptase polymerase chain reaction (RT-PCR) and number of days from the estimated date of SARS-CoV-2 infection until undetectable SARS-CoV-2 infection by RT-PCR.<br>• To assess VE to reduce the duration of symptoms of COVID-19.<br>• To evaluate VE against all-cause mortality.<br>• To assess VE against burden of disease due to COVID-19.<br>• To evaluate the genetic and/or phenotypic relationships of isolated SARS-CoV-2 strains to the vaccine sequence.<br>• To evaluate immune response markers after dosing with IP as correlates of risk of COVID-19 and as correlates of risk of SARS-CoV-2 infection.<br>• To conduct additional analyses related to furthering the understanding of SARS-CoV-2 infection and COVID-19, including analyses related to the immunology of this or other vaccines, detection of viral infection, and clinical conduct.<br>Exploratory for Part C:<br>• To evaluate the safety of a booster dose (BD) of mRNA-1273<br>• To evaluate the immunogenicity of a BD of mRNA-1273.
**Study Design and Methodology:**	This is a 3-part Phase 3 study, comprising Part A, Part B, and Part C (Figure 1). Participants in Part A, the Blinded Phase of this study, were blinded to their treatment assignment. Given that the primary efficacy endpoint for mRNA-1273 against COVID-19 was met per the protocol-defined interim analysis (IA), Part B, the Open-Label Observational Phase of this study, was designed to offer participants who received placebo in Part A of this study an option to request open-label mRNA-1273 while investigational vaccine was still available. With regard to eligibility for Part B, the US Centers for Disease Control (CDC)-Emergency Use Authorization (EUA) guidance specifications will supersede eligibility criteria in the protocol. Part C is designed to offer eligible participants in Part B the option to request a BD of mRNA-1273. With regard to eligibility for Part C, the CDC-EUA guidance specifications will supersede eligibility criteria in the protocol.

Part A, the Blinded Phase:

The Blinded Phase of this study (Figure 1) is a randomized, stratified, observer-blind, placebo-controlled evaluation of the efficacy, safety, and immunogenicity of mRNA-1273 SARS-CoV-2 vaccine compared to placebo in adults 18 years of age and older who have no known history of SARS-CoV-2 infection but whose locations or circumstances put them at appreciable risk of acquiring COVID-19 and/or SARS-CoV-2 infection.

Participants will be randomly assigned to receive injections of either 100 µg of mRNA-1273 vaccine or a placebo control in a 1:1 randomization ratio. Assignment will be stratified by age and health risk. This is a case-driven study and thus final sample size of the study will depend on the actual attack rate of COVID-19.

All participants will be assessed for efficacy and safety endpoints and provide a nasopharyngeal (NP) swab sample and blood sample before the first and second dose of IP, in addition to a series of post-dose blood samples for immunogenicity through 24 months after the second dose of IP. Efficacy assessments will include surveillance for COVID-19 with RT-PCR confirmation of SARS-CoV-2 infection after the first and second dose of IP.

As noted above, this is a case-driven study: if the prespecified criteria for early efficacy are met at the time of either IA or overall efficacy at the primary analysis, a final study report describing the efficacy and safety of mRNA-1273 will be prepared based on the data available at that time. In the event that success criteria are met either at the time of the IA or when the total number of cases toward the primary endpoint have accrued, participants will continue to be followed in a blinded fashion until Month 25 to enable assessment of long-term safety and durability of VE.

If the study concludes early, all participants will be requested to provide a final blood sample at the time of study conclusion.

Each participant will receive 2 doses of IP by 0.5 mL intramuscular (IM) injection, the first on Day 1 and the second on Day 29. An NP swab sample will be collected prior to the first and second dose of IP, for evaluation by RT-PCR. Participants will be given an electronic diary (eDiary) to report solicited adverse reactions (ARs) for 7 days after each dose of IP and to prompt an unscheduled clinic visit for clinical evaluation and NP swab sample if a participant experiences any symptoms of COVID-19 (throughout study). All participants will receive safety calls on Day 8, Day 15, Day 22, Day 36, and Day 43

that will serve both to monitor for unsolicited adverse events (AEs) and to monitor for symptoms of COVID-19.

Surveillance for COVID-19 will be performed through weekly contacts with the participant via a combination of telephone calls and completion of an eDiary starting at Day 1 through the end of the study.

Participants with symptoms of COVID-19 lasting at least 48 hours (except for fever and/or respiratory symptoms) will return to the clinic or will be visited at home by medically qualified site staff within 72 hours (an “Illness Visit”) to collect an NP swab sample for RT-PCR testing for SARS-CoV-2 and other respiratory pathogens, or alternatively, if a clinic or home visit is not possible, will submit a saliva sample for SARS-CoV-2 RT-PCR testing. Any confirmed COVID-19 occurring in a participant will be captured as a medically attended AE (MAAE) along with relevant concomitant medications and details about severity, seriousness, and outcome. All confirmed serious COVID-19 cases will be reported to the Sponsor or designee within 24 hours.

Starting with the Illness Visit, study participants will be monitored by the study investigator (or appropriately delegated study staff) for a 14-day period (from the Illness Visit or initial COVID-19 contact) or until symptoms resolve, whichever is later, and which may include symptoms persisting longer than the 28-day Convalescent Period. Each participant diagnosed with COVID-19 will monitor their body temperature, oxygen saturation, and symptoms following the diagnosis of COVID-19. In addition to daily follow-up of symptoms, assessments will include the collection of saliva samples during the 28-day period following the diagnosis of SARS-CoV-2 infection. The investigator will determine if medical attention is required due to worsening of COVID-19. Finally, a Convalescent Visit will be scheduled approximately 28 days after the initial Illness Visit. At this visit, a saliva sample will be collected, and a blood sample will be drawn for immunologic assessment of SARS-CoV-2 infection.

The 28-day period following the Illness Visit is referred to as the Convalescent Period. If during the Convalescent Period the participant has a positive result for SARS-CoV-2 from the Illness Visit, the participant will continue the Convalescent Period. If the participant has a negative result for SARS-CoV-2 from the Illness Visit, the participant will exit the Convalescent Period, including

discontinuation of daily telemedicine visits and collection of saliva samples, and will return to their respective study schedule.

At each dosing visit, participants will be instructed (Day 1) or reminded (Day 29) on how to document and report solicited ARs in the eDiary provided. Solicited ARs will be assessed for 7 days after each IP dose and unsolicited AEs will be assessed for 28 days after each IP dose; serious AEs (SAEs), MAAEs, AEs leading to withdrawal, will be assessed throughout the study.

Part B, the Open-Label Observational Phase:

Part B, the Open-Label Observational Phase of the study, is prompted by the authorization of a COVID-19 vaccine under EUA. Transitioning the study to Part B permits all ongoing study participants to be informed of the availability and eligibility criteria of any COVID-19 vaccine made available under an EUA and the option to offer all ongoing study participants who request unblinding, an opportunity to schedule a participant decision visit (PDV) at the study site to learn their original treatment assignment (placebo vs. mRNA-1273 vaccine).

Part B, the Open-Label Observational Phase of the study, also provides the opportunity for eligible study participants who previously received placebo to actively request to receive 2 doses of mRNA-1273 vaccine (subject to investigational vaccine availability).

All study participants will receive a notification letter and will be asked to schedule a PDV. Principal Investigators should consider current local public health guidance for administration of COVID-19 vaccines under EUA when determining the scheduling priority of participants.

All participants will proceed to Part B, the Open-Label Observational Phase of the study, starting with a PDV (Figure 2).

At the PDV, all participants will:

- Be encouraged to remain in the ongoing study,
- Be given the option to be unblinded as to their original group assignment (placebo vs. mRNA-1273 vaccine),
- Be counselled about the importance of continuing other public health measures to limit the spread of disease including social distancing, wearing a mask, and hand-washing,
- Sign a revised informed consent form (ICF),
- Provide a NP swab for RT-PCR for SARS-CoV-2 and a blood sample for serology prior to unblinding.

The following participants will continue with the original study Schedule of Events (SoEs) (Figure 2):

- Participants who request to not be unblinded,
- Participants who request to be unblinded and received mRNA-1273, and
- Participants who request to be unblinded and received placebo and choose to remain on placebo.

Participants who are unblinded, received placebo, are eligible, and request to receive mRNA-1273 (Figure 2) will have the following clinic visits:

- Open-label Dose 1 (OL-D1): To occur at the PDV or at a scheduled subsequent visit.
- Open-label Dose 2 (OL-D29): To occur 28 days after Dose 1 on OL-D1.
- Open-label Clinic Visit (OL-D57): To occur 1 month after Dose 2 on OL-D29.
- In addition, participants will continue to comply with the Original Study SoEs, as applicable.

Participants who are unblinded, received ONLY 1 dose of mRNA-1273, are eligible, and request to receive mRNA-1273 (Figure 2) will have the following clinic visits:

- Their second dose on OL-D1: To occur at the PDV or at a scheduled subsequent visit.
- And return on OL-D29: To occur 1 month after Dose 2 on OL-D1.

- In addition, participants will continue to comply with the Original Study SoEs, as applicable.

All participants remain on their original SoE to complete 24 months of follow-up after Dose 2; study participants who enter the Supplemental/Modified Supplemental SoE as described above do so in addition to their original SoE. Accordingly, all study participants will complete the full study follow-up to 24 months after the second dose of their original inoculation (mRNA-1273 or placebo), or 25 months after the first dose if the second dose is missed.

At a point when the mRNA-1273 vaccine is no longer available for study use, any participant who schedules a PDV after this timepoint will be unblinded (with no option to stay blinded) but will not be offered to receive mRNA-1273 study vaccine. These participants will have all of the same procedures performed as outlined in the SoE table, with the exception of being offered to receive mRNA-1273 vaccine.

After the Biologics License Application database lock (on 04 May 2021), any remaining participants who had not been unblinded were unblinded by the Moderna study team and received their unblinding treatment assignment (placebo vs. messenger RNA [mRNA] vaccine) via certified letter from the study site.

Part C, the Booster Dose Phase

Part C, providing a BD for all eligible participants who choose to receive one, is prompted by the interim results of an ongoing Moderna Phase 2a study (mRNA-1273-P201; NCT04405076), in which participants who, 6 to 8 months prior, received 2 doses of 50 µg or 100 µg of mRNA-1273 were administered a 50 µg booster of mRNA-1273. Results demonstrated enhanced immune responses compared to pre-boost levels and met the noninferiority criteria stipulated in the Food and Drug Administration Guidance on EUA for Vaccines to Prevent COVID-19. In addition, no new safety signals emerged upon administration of the BD in Study mRNA-1273-P201. Based on cumulative evidence, the benefit-risk profile of a BD of mRNA-1273 is favorable, particularly in light of increasing breakthrough disease with the emergence of variants. With the wider availability of boosters under EUA, providing the option for a BD to all eligible participants currently enrolled in Part B is expected to promote retention of participants in the ongoing study and thereby defend the scientific integrity of the

study for the planned 2-year duration of follow-up after the completion of the primary vaccination series regimen.

Each study participant will receive a notification letter and will be asked to schedule a BD-1 visit at their study site. Principal Investigators should consider current local public health guidance for administration of COVID-19 vaccines under EUA and marketing authorization (if any) when determining the scheduling priority of participants.

At the BD-1 visit, each participant will:

- Be encouraged to remain in the ongoing study,
- Sign a revised ICF that includes both updated safety information relevant to the ongoing study and a BD, and the option to receive a BD,
- Be given the option to receive a BD consisting of a 50 µg dose of mRNA-1273,
- Be counselled about the importance of continuing other public health measures to limit the spread of disease including social distancing, wearing a mask, and hand-washing.

After the BD-1 visit, participants who do not receive a BD will continue with the Part C Supplemental SoE for the BD-2 and BD-3 visits but will not have the BD-1a visit or the safety calls.

Participants who request a BD and are eligible and have no contraindications to further dosing will have the following study site visits and complete scheduled activities (subject to investigational vaccine availability) according to the Part C Supplemental SoE:

- BD-1 visit: Participants will receive a single 50 µg dose of mRNA-1273.
- BD-1a visit: Optional, and subject to blood sample kit availability, Day 4, 3 days after BD on Day 1.
- BD-2 visit: Day 29, 28 days after the BD on Day 1.
- BD-3 visit: Day 181, 180 days after the BD on Day 1.

The investigator is responsible for conducting all assessments as specified in the Part C Supplemental SoE, according to the schedule. As this Supplemental SoE is intended to occur in addition to the original SoEs being followed by all participants in Part B, there is a possibility for study visits to overlap. If visits overlap according to

respective visit windows, a single visit may be done with the combined study procedures completed once.

This study will be conducted in compliance with the protocol, Good Clinical Practice, and all applicable regulatory requirements.

Randomization: In Part A, the Blinded Phase of the study, approximately 30,000 participants will be randomly assigned in 1:1 ratio to receive either mRNA-1273 100 µg or placebo. The randomization will be in a blinded manner using a centralized interactive response technology, in accordance with pre-generated randomization schedules.

Stratification: Randomization in Part A, the Blinded Phase of the study, will be stratified based on age and, if they are < 65 years of age, based on the presence or absence of risk factors for severe illness from COVID-19 based on CDC recommendation as of March 2020. There will be 3 strata for randomization: ≥ 65 years, < 65 years and categorized to be at increased risk ("at risk") for the complications of COVID-19, and < 65 years "not at risk". Risk will be defined based on the study participants' relevant past and current medical history. At least 25% of enrolled participants, up to 50%, will be either ≥ 65 years of age or < 65 years of age and "at risk" at Screening.

Participants who are < 65 years old will be categorized as at risk for severe COVID-19 illness if they have at least 1 of the following risk factors at Screening:

- Chronic lung disease (eg, emphysema and chronic bronchitis, idiopathic pulmonary fibrosis, and cystic fibrosis) or moderate to severe asthma
- Significant cardiac disease (eg, heart failure, coronary artery disease, congenital heart disease, cardiomyopathies, and pulmonary hypertension)
- Severe obesity (body mass index ≥ 40 kg/m^2)
- Diabetes (Type 1, Type 2, or gestational)
- Liver disease
- Human immunodeficiency virus infection

Study Population: Participants (males and females 18 years of age or older at time of consent), who are at risk of SARS-CoV-2 infection with no known history of SARS-CoV-2 infection, are a subset of the planned target population. Additionally, potential study participants at increased risk

	of complications from COVID-19 will be included, since it is hypothesized that these participants might derive the greatest benefit from a vaccine. Participants ≥ 65 years of age will be eligible for enrollment with or without underlying medical conditions further increasing their risk of severe COVID-19.<br>Study sites may be selected based on SARS-CoV-2 infection risk of the local population. Approximately 30,000 participants will be enrolled.<br>The full lists of inclusion and exclusion criteria are provided in the body of the protocol.
**Efficacy Assessments:**	**Primary Efficacy Assessment:**<br>To be considered as a case of COVID-19 for the evaluation of the Primary Efficacy Endpoint, the following criteria must be met:<br>• The participant must have experienced at least TWO of the following systemic symptoms: fever (≥ 38ºC), chills, myalgia, headache, sore throat, new olfactory and taste disorder(s), OR<br>• The participant must have experienced at least ONE of the following respiratory signs/symptoms: cough, shortness of breath or difficulty breathing, OR clinical or radiographical evidence of pneumonia; AND<br>• The participant must have at least 1 NP swab, or saliva sample (or respiratory sample, if hospitalized) positive for SARS-CoV-2 by RT-PCR.<br>**Secondary Efficacy Assessments:**<br>To be considered severe COVID-19, the following criteria must be met: a confirmed case of COVID-19 as per the Primary Efficacy Endpoint case definition, plus any of the following:<br>• Clinical signs indicative of severe systemic illness, respiratory rate ≥ 30 per minute, heart rate ≥ 125 beats per minute, SpO_2 ≤ 93% on room air at sea level or PaO_2/FIO_2 < 300 mmHg, OR<br>• Respiratory failure or acute respiratory distress syndrome (defined as needing high-flow oxygen, non-invasive or mechanical ventilation, or extracorporeal membrane oxygenation), evidence of shock (systolic blood pressure [BP] < 90 mmHg, diastolic BP < 60 mmHg or requiring vasopressors), OR<br>• Significant acute renal, hepatic, or neurologic dysfunction, OR

	• Admission to an intensive care unit or death.<br>The secondary case definition of COVID-19 is defined as having any of the following systemic symptoms: fever (temperature ≥ 38ºC) or chills, cough, shortness of breath or difficulty breathing, fatigue, muscle aches or body aches, headache, new loss of taste or smell, sore throat, nasal congestion or rhinorrhea, nausea or vomiting or diarrhea, AND a positive NP swab or saliva sample (or respiratory sample, if hospitalized) for SARS-CoV-2 by RT-PCR.<br>Death attributed to COVID-19 is defined as any participant who dies during the study with a cause directly attributed to a complication of COVID-19.<br>Asymptomatic SARS-CoV-2 infection is determined by seroconversion due to infection assessed by binding antibody (bAb) levels against SARS-CoV-2 as measured by a ligand-binding assay specific to the SARS-CoV-2 nucleocapsid protein and a negative NP swab sample for SARS-CoV-2 at Day 1.
**Immunogenicity Assessments:**	Immunogenicity assessments will include the following:<br>• Serum bAb levels against SARS-CoV-2 as measured by ligand-binding assay specific to the SARS-CoV-2 S protein.<br>• Serum bAb levels against SARS-CoV-2 as measured by ligand-binding assay specific to the SARS-CoV-2 nucleocapsid protein.<br>• Serum neutralizing antibody (nAb) titer against SARS-CoV-2 as measured by pseudovirus and/or live virus neutralization assays.
**Safety Assessments:**	Safety assessments will include monitoring and recording of the following for each participant:<br>• Solicited local and systemic ARs that occur during the 7 days following each injection (ie, the day of injection and 6 subsequent days). Solicited ARs will be recorded daily using eDiaries (Part A).<br>• Unsolicited AEs observed or reported during the 28 days following each injection (ie, the day of injection and 27 subsequent days) (Part A [all participants] and Part C [only those who receive the BD]).<br>• AEs leading to discontinuation from dosing and/or study participation from Day 1 through Day 759 or withdrawal from the study.

- MAAEs from Day 1 through Day 759 or withdrawal from the study.
- SAEs from Day 1 through Day 759 or withdrawal from the study.
- AEs of special interest (AESIs; for participants who receive the BD in Part C) from BD-Day 1 through Day 759 or withdrawal from the study.
- Abnormal vital sign measurements.
- Physical examination findings.
- Pregnancy and accompanying outcomes.
- Concomitant medications and non-study vaccinations.

**Investigational Product, Dosage, and Route of Administration:**	The mRNA-1273 IP is a lipid nanoparticle (LNP) dispersion of an mRNA encoding the prefusion stabilized S protein of SARS-CoV-2 formulated in LNPs composed of 4 lipids (1 proprietary and 3 commercially available): the proprietary ionizable lipid SM-102; cholesterol; 1,2-distearoyl-sn-glycero-3 phosphocholine (DSPC); and 1 monomethoxypolyethyleneglycol-2,3-dimyristylglycerol with polyethylene glycol of average molecular weight 2000 (PEG2000-DMG). The mRNA-1273 vaccine is provided as a sterile liquid for injection and is a white to off- white dispersion in appearance, at a concentration of 0.2 mg/mL in 20 mM Tris buffer containing 87 mg/mL sucrose and 10.7 mM sodium acetate at pH 7.5.<br><br>The placebo is 0.9% sodium chloride (normal saline) injection, which meets the criteria of the United States Pharmacopeia.<br><br>In Part A and Part B, as applicable, IP will be administered as an IM injection into the deltoid muscle on a 2-dose injection schedule on Day 1 and Day 29. For injections administered for Part A and Part B, each injection will have a volume of 0.5 mL and contain mRNA-1273 100 µg or saline placebo. For Part C, each injection will have a volume of 0.25 mL and contain mRNA-1273 50 µg. Preferably, vaccine should be administered into the nondominant arm. The second dose of IP should be administered in the same arm as the first dose (Part A and Part B, as applicable).<br><br>The IP will be prepared for injection as a single 0.5 mL (Part A and Part B) or 0.25 mL (Part C) dose for each participant per protocol, as detailed in the Pharmacy Manual. Unblinded personnel who will not participate in any other aspect of the study during Part A, the Blinded Phase, will perform IP accountability, dose preparation, and IP administration. Study site personnel who were blinded during the Blinded Phase will be unblinded at the participant level at the PDV.
**Sample Size:**	The sample size is driven by the total number of cases to demonstrate VE (mRNA-1273 vs. placebo) to prevent COVID-19 in Part A. Under the assumption of proportional hazards over time and with 1:1 randomization of mRNA-1273 and placebo, a total of 151 COVID-19 cases will provide 90% power to detect a 60% reduction in hazard rate (60% VE), rejecting the null hypothesis H0: VE ≤ 30%, with 2 IAs at 35% and 70% of the target total number of cases using a 1-sided O'Brien-Fleming boundary for efficacy and a log-rank test statistic with a 1-sided false positive error rate of 0.025. The total number of cases pertains to the Per-protocol (PP) Set accruing at least 14 days after the second dose. There are 2 planned

IAs in this study, which will be performed when approximately 35% and 70% of the target total number of cases have been observed. Approximately 30,000 participants will be randomized with the following assumptions:

- The target VE against COVID-19 is 60% (with 95% confidence interval [CI] lower bound ruling out 30%, rejecting the null hypothesis H0: VE ≤ 30%).
- A 6-month COVID-19 incidence rate of 0.75% in the placebo arm.
- An annual dropout rate of 2% (loss of evaluable participants).
- Two IAs at 35% and 70% of total target cases across the 2 treatment groups with O'Brien-Fleming boundaries for efficacy monitoring.
- 3-month uniform accrual.
- Approximately 15% of participants will be excluded from the PP population, and participants are at risk for COVID-19 starting 14 days after the second dose.

Power for Selected Secondary Efficacy Endpoints:

For the secondary objective on VE against virologically confirmed SARS-CoV-2 infection or COVID-19 regardless of symptomatology or severity (COV-INF), the study will have ≥ 90% power to demonstrate the VE is above 30% (to reject null hypothesis VE ≤ 30%) at 1-sided alpha of 2.5% if the true VE to prevent COV-INF is 60%, because every COVID-19 endpoint is necessarily a COV-INF endpoint.

For the secondary objective on VE against severe COVID-19, the power of demonstrating VE based on a total of 30 and 60 events under different scenarios of true VE and VE criteria has been calculated.

**Statistical Methods:**	**Statistical Hypotheses:** For the primary efficacy objective, the null hypothesis of this study is that the VE of mRNA-1273 to prevent first occurrence of COVID-19 is ≤ 30% (ie, $H_0^{efficacy}$: VE ≤ 0.3).<br><br>The study will be considered to meet the primary efficacy objective if the corresponding CI of VE rules out 30% at either one of the IAs or at the primary analysis. In the primary analysis of VE of COVID-19, cases will be counted starting 14 days after the second dose of IP.<br><br>Vaccine efficacy is defined as the percent reduction in the hazard of the primary endpoint (mRNA-1273 vs. placebo). Equivalently, the null hypothesis is:<br><br>• $H_0^{efficacy}$: hazard ratio (HR) ≥ 0.7 (equivalently, proportional hazards VE ≤ 0.3).<br><br>A stratified Cox proportional hazard model will be used to assess the magnitude of the treatment group difference (ie, HR) between mRNA-1273 and placebo at a 1-sided 0.025 significance level.<br><br>**Analysis Populations:** Analysis populations for statistical analyses are Randomization Set, Full Analysis Set (FAS), Modified Intent-to-Treat (mITT) Set, PP Set, Immunogenicity Subset, Solicited Safety Set, and Safety Set, as shown below:<br><br>• Randomization Set: All participants who are randomized, regardless of the participants' treatment status in the study.<br>• FAS: All randomized participants who received at least one dose of IP. Participants will be analyzed according to the group to which they were randomized.<br>• mITT Set: All participants in the FAS who had no immunologic or virologic evidence of prior COVID-19 (ie, negative NP swab test at Day 1 and/or bAb against SARS-CoV-2 nucleocapsid below limit of detection or lower limit of quantification [LLOQ]) at Day 1 before the first dose of IP. Participants will be analyzed according to the group to which they were randomized.<br>• PP Set: All participants in the mITT Set who received planned doses of IP per schedule and have no major protocol deviations, as determined and documented by Sponsor prior to database lock and unblinding, that impact critical or key study data. Participants will be analyzed according to the group to which they were randomized.<br>• Immunogenicity Subset: All participants in the FAS who were sampled into subset for characterizing mRNA-1273 immunogenicity and had a valid immunogenicity test result prior

to the first dose of IP and at least 1 valid result after the first dose of IP. Participants in the subset who had major protocol deviations that impact critical or key immunogenicity or study data may be excluded from the Immunogenicity Subset. The details of the Immunogenicity Subset will be documented prior to analysis of immunogenicity data.

- Solicited Safety Set: The Solicited Safety Set consists of all randomized participants who received at least one dose of IP and contributed any solicited AR data. The Solicited Safety Set will be used for the analyses of solicited ARs and participants will be included in the treatment group corresponding to the IP that they actually received.
- Safety Set: All randomized participants who received at least one dose of IP. The Safety Set will be used for all analyses of safety except for the solicited ARs. Participants will be included in the treatment group corresponding to the IP that they actually received.

Efficacy Analyses: Efficacy analyses will be performed using the FAS, mITT, and PP populations, and participants will be included in the treatment group to which they were randomized. The primary analysis population for efficacy will be the PP Set.

The table below summarizes the analysis approach for primary and secondary efficacy endpoints for Part A, the randomized, observer-blind, and placebo-controlled phase of the study. Sensitivity analysis methods are described for each endpoint as applicable.

Endpoint	Statistical Analysis Methods
**Primary endpoint:**<br>VE of mRNA-1273 to prevent COVID-19	• Primary analysis: VE will be estimated with 1 – HR (mRNA-1273 vs. placebo) using a Cox proportional hazard regression model with treatment group as a fixed effect and adjusting for stratification factor based on the PP Set, with cases counted starting 14 days after the second dose of IP.<br>• Analysis using the same model based on the mITT Set.<br>• Sensitivity analysis using the same model based on the PP Set, with cases counted starting either immediately after the second dose of IP or immediately after the first dose of IP.<br>• Subgroup analysis of the primary efficacy endpoint will be performed to assess consistency of VE, such as in the age groups ≥ 18 and < 65 years and ≥ 65 years.<br>• Supportive analysis of VE to be estimated with 1 – ratio of incidence rates with 95% CI using the exact method conditional upon the total number of cases.<br>• Supportive analysis of cumulative incidence VE.
**Secondary endpoints:**<br>• Vaccine efficacy of mRNA-1273 to prevent severe COVID-19<br>• Vaccine efficacy of mRNA-1273 to prevent serologically	Similar analysis method as for the primary endpoint analysis.<br>For each of the secondary endpoints:<br>• Primary analysis: VE will be estimated with 1 – HR (mRNA-1273 vs. placebo) using a Cox proportional

confirmed SARS-CoV-2 infection or COVID-19 regardless of symptomatology or severity.<br>• Vaccine efficacy of mRNA-1273 to prevent COVID-19 using a secondary definition of symptoms<br>• Vaccine efficacy of mRNA-1273 to prevent death caused by COVID-19<br>• Vaccine efficacy of mRNA-1273 to prevent COVID-19 after the first dose of IP<br>• Vaccine efficacy of mRNA-1273 to prevent asymptomatic SARS-CoV-2 infection	hazard regression model with treatment group as a fixed effect and adjusting for stratification factor based on the PP Set, with cases counted starting 14 days after the second dose of IP.<br>• Analysis using the same model based on the mITT Set.<br>• Sensitivity analyses with cases counted starting immediately after the second dose of IP, 14 days after the first dose of IP, immediately after the first dose of IP, and immediately after randomization.<br>• Vaccine efficacy and 95% CI based on the case incidence will be estimated with 1 – ratio of incidence rates using the exact method conditional upon the total number of cases.
Vaccine efficacy of mRNA-1273 to prevent COVID-19 in all study participants, regardless of evidence of prior SARS-CoV-2 infection	The FAS population will be used for this secondary objective, using similar analysis methods as for the primary endpoint analysis.<br>• Primary analysis: VE will be estimated with 1 – HR (mRNA-1273 vs. placebo) using a Cox proportional hazard regression model with treatment group as a fixed effect and adjusting for stratification factor based on the FAS, with cases counted starting 14 days after the second dose of IP.<br>• Sensitivity analyses with cases counted starting

	immediately after the second dose of IP, 14 days after the first dose of IP, immediately after the first dose of IP, and immediately after randomization.

The table below summarizes the analysis approach for long-term endpoints.

Endpoint	Statistical Analysis Methods
**Long-term endpoints:**<br>Cases starting 14 days after the second injection of IP for participants in the mRNA-1273 Cohort and the Placebo Cohort, or after the second injection of mRNA-1273 for participants in the Placebo-mRNA-1273 Cohort<br>• COVID-19<br>• Severe COVID-19<br>• Either COVID-19 or SARS-CoV-2 infection<br>• Secondary definition of COVID-19<br>• Death caused by COVID-19<br>• COVID-19 regardless of evidence of prior SARS-CoV-2 infection determined by serologic titer against SARS-CoV-2 nucleocapsid<br>Cases starting 14 days after the first injection of IP for participants in the mRNA-1273 Cohort and the Placebo Cohort, or after the first injection of mRNA-1273 for participants in the Placebo-mRNA-1273 Cohort	• Long-term efficacy will be evaluated after the primary analysis of the primary vaccination series with mRNA-1273 by including data collected in the Open-Label Phase.<br>• Long-term efficacy data will be summarized descriptively by treatment cohort without cohort comparison.<br>• In the primary approach, cases will be counted starting 14 days after the second dose of IP for participants in treatment cohorts of mRNA-1273 and placebo or starting 14 days after the second dose of mRNA-1273 for participants in the Placebo-mRNA-1273 Cohort.<br>• Sensitivity analyses with cases starting from the second dose, 14 days after the first dose, or first dose of IP for participants in the cohorts of mRNA-1273 and placebo, or mRNA-1273 for participants in the Placebo-mRNA-1273 Cohort may be provided.<br>• Incidences of cases assessed by numbers, rates, and 2-sided 95% CI based on the exact method adjusting for person-time will be summarized by treatment cohort.<br>• The Kaplan-Meier analysis will be used to estimate cumulative incidences of time

• COVID-19<br>SARS-CoV-2 infection in the absence of symptoms defining COVID-19 starting 14-days after the second injection of:<br>• IP for participants in the mRNA-1273 Cohort and the Placebo Cohort, or mRNA-1273 for participants in the Placebo-mRNA-1273 Cohort	to first cases by treatment cohort.<br>• Long-term efficacy analysis will be performed using the PP Set and mITT Set.<br>• Efficacy analyses of unblinded/open-label phase data collected in Part B will be provided, as measured by the incidence rate of COVID-19 after the BD of mRNA-1273. The details of analysis of long-term efficacy and open-label phase data (Part B and Part C) will be provided in the statistical analysis plan (SAP).

Safety: Safety and reactogenicity will be assessed by clinical review of all relevant parameters. Safety analyses will be provided for Parts A, B, and C separately unless specified otherwise.

All safety analyses will be based on the Safety Set, except summaries of solicited ARs, which will be based on the Solicited Safety Set. All safety analyses will be provided by treatment group, and by treatment cohort as applicable, unless otherwise specified.

The number and percentage of participants with any solicited local AR, with any solicited systemic AR, and with any solicited AR during the 7-day follow-up period after each injection will be provided only for Part A. A 2-sided 95% exact CI using the Clopper-Pearson method will be also provided for the percentage of participants with any solicited AR for each treatment group. Analysis of solicited AR will be provided using the Solicited Safety Set for Part A.

The number and percentage of participants with unsolicited AEs (Part A [all participants] and Part C [only those who receive the BD]), SAEs, MAAEs, AESIs (Part C [only those who receive the BD]), and AEs leading to discontinuation from IP or withdrawal from the study will be summarized. Unsolicited AEs will be presented by Medical Dictionary for Regulatory Activities preferred term and system organ class. Analyses of AEs will be provided for Part A and

Part B using the Safety Set. For Part C, analysis of AEs will be provided in the Part C Safety Set (ie, participants who received booster in Part C).

For all other safety parameters, descriptive summary statistics will be provided.

Further details will be described in the SAP.

Immunogenicity: The secondary immunogenicity endpoints will be analyzed using the Immunogenicity Subset by treatment group, by treatment cohort as applicable, and by baseline SARS-CoV-2 serostatus, unless otherwise specified.

The SAP will describe the complete set of immunogenicity analyses, including the approach to sample individuals into an Immunogenicity Subset for characterizing vaccine immunogenicity and assessing immunological correlates of risk and protection.

Part A

Data from quantitative immunogenicity assays will be summarized for each treatment group using positive response rates and geometric means with 95% CI, for each timepoint for which an assessment is performed. Data from qualitative (ie, yielding a positive or negative result) assays will be summarized by tabulating the frequency of positive responses for each assay by group at each timepoint that an assessment is performed. Analyses will focus on the 2 key immunogenicity timepoints and the change in marker response between them: Day 1 before the first dose of IP and Day 57 (28 days after the second dose of IP). The SAP will describe the complete set of immunogenicity analyses.

Quantitative levels or geometric mean titer (GMT) of specific bAb with corresponding 95% CI at each timepoint and geometric mean fold rise (GMFR) of specific bAb with corresponding 95% CI at each postbaseline timepoint over pre-dose baseline at Day 1 will be provided by study arm. Descriptive summary statistics including median, minimum, and maximum will also be provided.

The GMT of specific nAb with corresponding 95% CI at each timepoint and GMFR of specific nAb with corresponding 95% CI at each post-baseline timepoint over pre-dose baseline at Day 1 will be provided by study arm. Descriptive summary statistics including median, minimum, and maximum will also be provided. For summarizations of group variables values, antibody (Ab) values reported as below the LLOQ will be replaced by 0.5 × LLOQ. Values

that are reported as greater than the upper limit of quantification (ULOQ) without the actual values will be converted to the ULOQ.

The number and percentage of participants with a fold rise ≥ 2, ≥ 3, and ≥ 4 of serum SARS-CoV-2-specific nAb titers and participants with seroresponse from baseline will be provided with 2-sided 95% CI using the Clopper-Pearson method at each post-baseline timepoint. Seroresponse at a participant level may be defined as a change from below the LLOQ to at least 4 times the LLOQ, or at least a 4-fold rise in nAb or vaccine antigen-specific bAb in participants with pre-existing nAb or bAb of at least the LLOQ at baseline/pre-vaccination. Seroresponse may also be defined for each specific assay assessing nAb or bAb. The definition of seroresponse will be finalized and documented in the SAP.

The GMT of specific nAb for each group and the geometric mean ratio (GMR) of mRNA-1273 versus placebo in Part A with corresponding 2-sided 95% CI will be estimated at each study timepoint using an analysis of covariance model with the treatment group and baseline values, if applicable, as explanatory variables, the analysis may adjust for the stratification factor.

Part C

In Part C, the immunogenicity analysis of BD vaccine response will be performed using the noninferiority tests of the 2 null hypotheses based on the 2 key secondary endpoints, respectively.

Part C Key Secondary Endpoint 1: Ab GMT at BD-Day 29

Hypothesis: immunogenicity response to mRNA-1273 BD as measured by Ab GMT at BD-Day 29 in Part C is noninferior compared with Ab GMT at Day 57 (28 days after the second dose) in the primary series of mRNA-1273.

The noninferiority of post-booster GMT at BD-Day 29 in Part C as compared to the primary series at Day 57 is considered to be demonstrated if the lower bound of the 95% CI of the GMR (ratio of GMT at BD-Day 29 vs. GMT at Day 57 [28 days after Dose 2 of the primary series]) is ≥ 0.67 using a noninferiority margin of 1.5 (1.5-fold immuno-bridging margin for the lower bound of the 95% CI for GMT ratio/GMR).

The GMT with 95% CI will be summarized using t-distribution of the log transferred values and then back transformed to the original scale. The GMR with 95% CI to compare post-booster GMT at BD-Day 29 in Part C with the primary series GMT at Day 57 (28 days after the

second dose) will be computed based on the t-distribution of mean difference in the log transferred values and then back transformed to the original scale.

Part C Key Secondary Endpoint 2: Ab Seroresponse Rate at BD-Day 29

Hypothesis: immunogenicity response to mRNA-1273 BD as measured by seroresponse rate (SRR) at BD-Day 29 in Part C is noninferior compared with SRR of the primary series at Day 57 (28 days after the second dose of the primary series of mRNA-1273).

The noninferiority in SRR post-booster at BD-Day 29 in Part C compared with SRR of the primary series at Day 57 (28 days after the second dose of the primary series of mRNA-1273) is considered to be demonstrated if the lower bound of the 95% CI of the SRR difference (SRR of the booster at BD-Day 29 – SRR of the primary series at Day 57) is > -10% (using the noninferiority margin of 10%). The SRR difference is defined as the seroresponse rate at BD-Day 29 in Part C minus the seroresponse rate at Day 57 (28 days after the second dose) following the primary series of mRNA-1273. The seroresponse of a booster or the primary series is defined as a titer change from baseline (pre-Dose 1 in primary series) below the LLOQ to ≥ 4 × LLOQ, or at least a 4-fold rise if baseline is ≥ LLOQ.

The SRR with 95% CI (using Clopper-Pearson method) will be provided. The SRR difference with 95% CI to compare post-booster SRR at BD-Day 29 in Part C with the primary series SRR at Day 57 (28 days after the second dose) will be computed. The method for computing 95% CI of seroresponse rate difference will be provided in the SAP.

The primary immunogenicity objective in Part C is met if noninferiority is demonstrated based on both key secondary endpoints (both GMT and SRR).

1.2. Schema

Figure 1: Study Flow Diagram: Part A, the Blinded Phase Followed by Part B, the Open-Label Phase Including Part C, the Booster Dose Phase

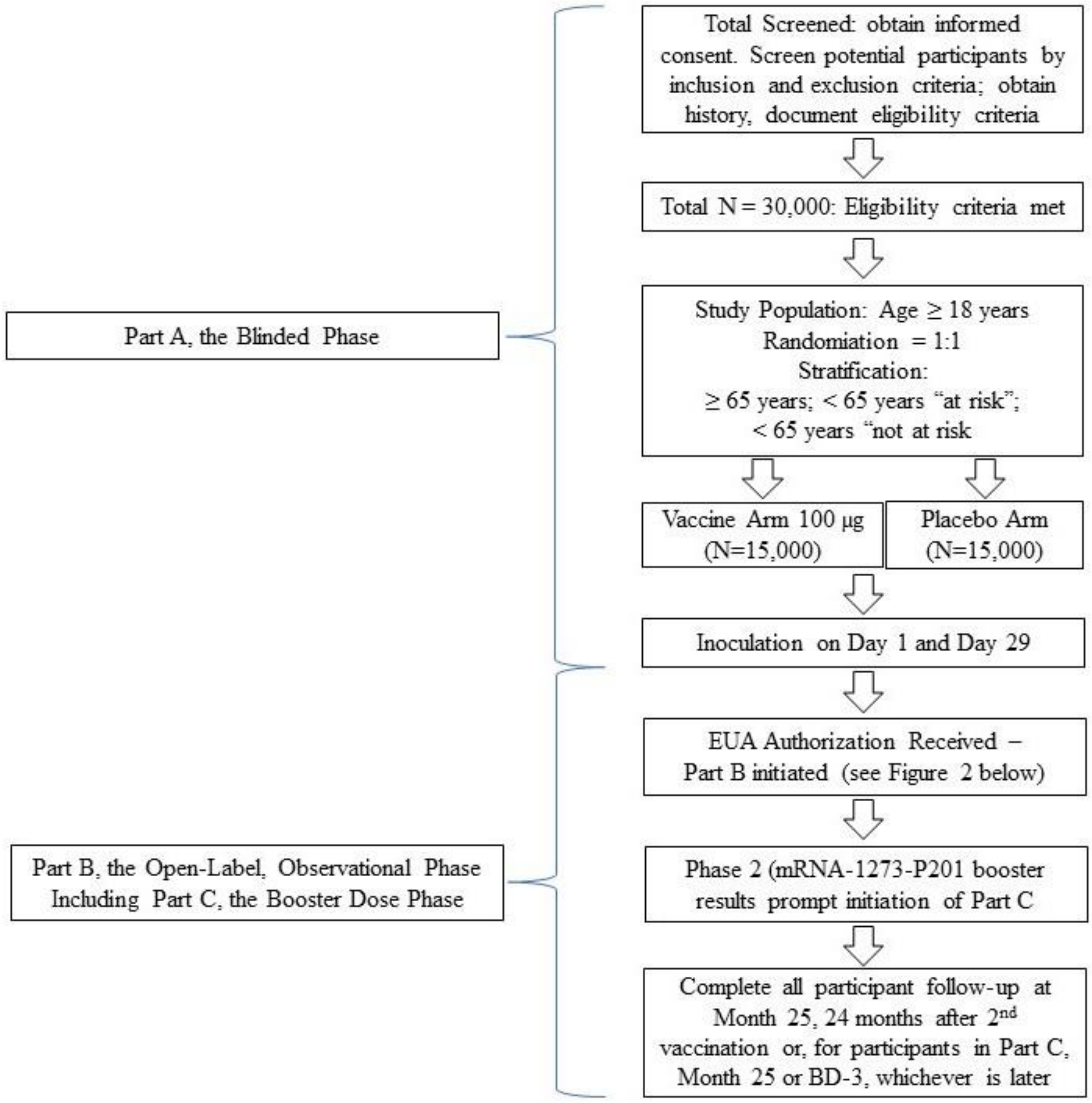

Abbreviations: BD = booster dose visit; EUA = emergency use authorization

Figure 2: Part B, the Open-Label Observational Phase, and Part C, the Booster Dose Phase

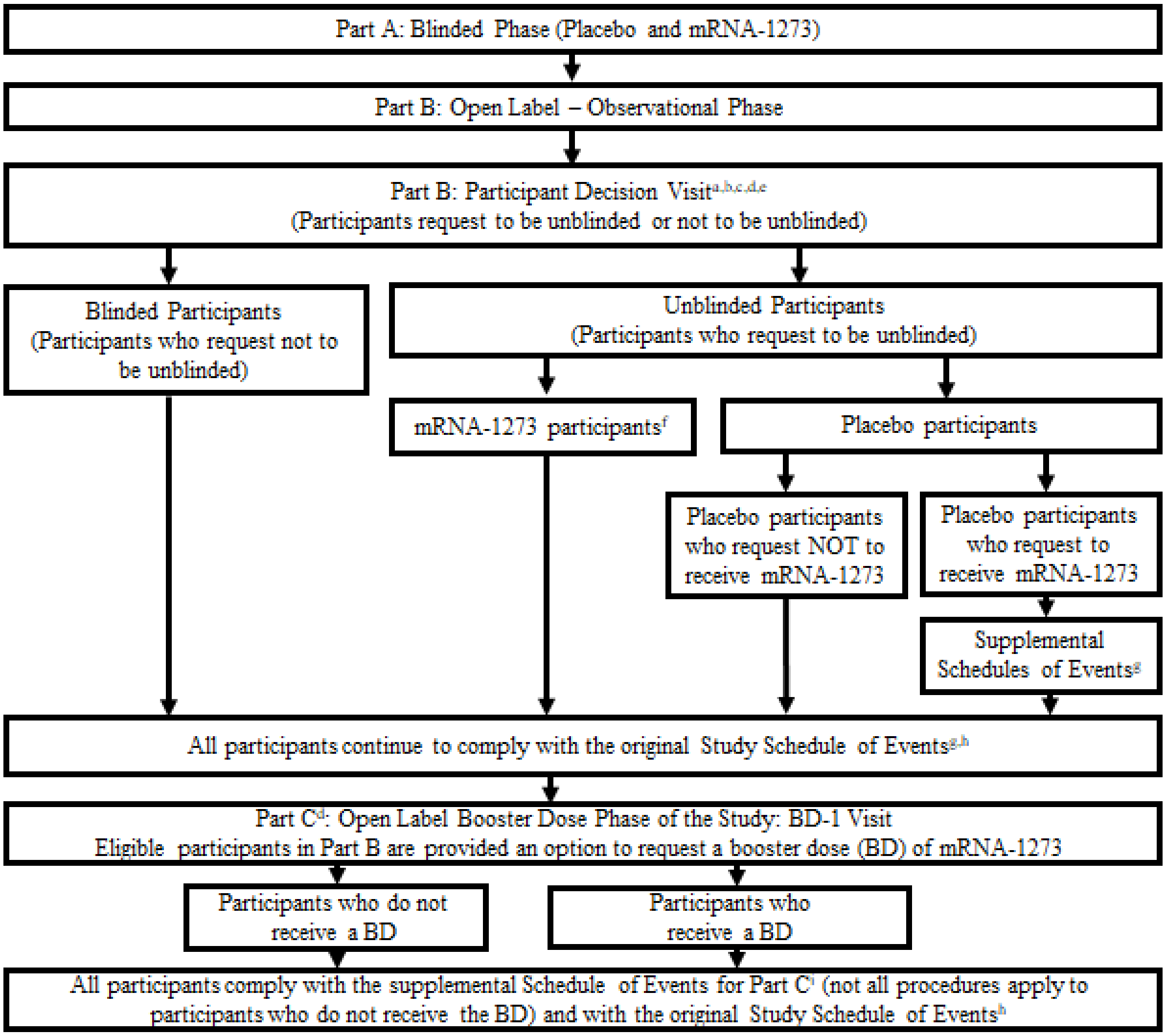

[a] All participants are encouraged to remain in the study.
[b] All participants are given the option to be unblinded to treatment received in Part A: Blinded Phase.
[c] All participants are counselled about the importance of continuing other public health measures to limit the spread of disease including physical-social distancing, wearing a mask, and hand-washing.
[d] All participants sign a revised informed consent form.
[e] All participants consent to provide a nasopharyngeal swab and a blood sample for immunologic analysis.
[f] mRNA-1273 recipients who only received 1 dose of the mRNA-1273 vaccine will receive the second dose of mRNA-1273 vaccine. Additional details provided in the Supplemental Schedule of Events (Table 22).
[g] Placebo recipients who request to receive mRNA-1273 and meet eligibility will comply with a Supplemental Schedule of Events (Table 21) in addition to the Original Study Schedule of Events.
[h] Original Study Schedule of Events (Table 16, Table 17, Table 18, and Table 19).
[i] Procedures required or not required based on receipt of the booster dose are clarified in the Part C Supplemental Schedule of Events (Table 23).

TABLE OF CONTENTS

Clinical Study Protocol 1
Declaration of Investigator 4
Protocol Amendment Summary of Changes 5
IRB and Regulatory Authority Approval 10
1. Protocol Summary 11
1.1. Synopsis 11
1.2. Schema 35
Table of Contents 37
List of Tables 42
List of Figures 43
List of Abbreviations 44
2. Introduction 47
2.1. Study Rationale 47
2.2. Background and Overview 47
2.2.1. Nonclinical Studies 48
2.2.2. Clinical Studies 48
2.3. Benefit/Risk Assessment 51
2.3.1. Potential Benefits of Study Participation 51
2.3.2. Risks From Study Participation 51
2.3.3. Overall Benefit/Risk Conclusion 53
3. Objectives and Endpoints 54
4. Study Design 59
4.1. General Design 59
4.1.1. Part A, the Blinded Phase 59
4.1.2. Part B, the Open-Label Observational Phase 61
4.1.3. Part C, the Booster Dose Phase 64
4.2. Scientific Rationale for Study Design 65
4.3. Choice of Dose and Control Product 66
4.4. End of Study Definition 66
5. Study Population 68
5.1. Inclusion and Exclusion Criteria (Part A, Part B, and Part C) 68
5.1.1. Inclusion Criteria 68
5.1.2. Exclusion Criteria 69

5.2. Participant Restrictions 71
5.3. Screen Failures (Part A, Blinded Phase Only) 71
6. Study Treatment 72
6.1. Investigational Product 72
6.2. Dosing and Management of Investigational Product 72
6.2.1. Method of Randomly Assigning Participants to Treatment Groups (Part A, Blinded Phase Only) 72
6.2.2. Administration of Investigational Product 73
6.2.3. Delivery and Receipt 74
6.2.4. Packaging and Labeling 74
6.2.5. Storage 75
6.2.6. Investigational Product Accountability 75
6.2.7. Handling and Disposal 75
6.2.8. Unblinding 76
6.3. Study Treatment Compliance 78
6.4. Prior and Concomitant Therapy 79
6.4.1. Prior Medications and Therapies 79
6.4.2. Concomitant Medications and Therapies 79
6.4.3. Concomitant Medications and Vaccines That May Lead to the Elimination of a Participant From Per-protocol Analyses 80
7. Delaying or Discontinuing Study Treatment and Participant Withdrawal from the Study 81
7.1. Criteria for Delay of Study Treatment 81
7.2. Discontinuation of Study Treatment 82
7.3. Participant Withdrawal From the Study 83
7.3.1. Participant Withdrawal 83
7.4. Lost to Follow-up 84
8. Study Assessments and Procedures 86
8.1. Efficacy and Immunogenicity Assessments and Procedures 87
8.1.1. Efficacy Assessments Related to COVID-19 and SARS-CoV-2 Infection 87
8.1.2. Surveillance for COVID-19 Symptoms 88
8.1.3. Convalescent Period Starting With the Illness Visit 91
8.1.4. Ancillary Supplies for Participant Use 93

8.1.5. Immunogenicity Assessments 94
8.2. Safety Assessments 94
8.2.1. Safety Phone Calls 95
8.2.2. Use of Electronic Diaries 95
8.2.3. Demographics/Medical History 98
8.2.4. Physical Examination 98
8.2.5. Vital Sign Measurements 98
8.2.6. Blood Sampling 99
8.3. Safety Definitions and Procedures 100
8.3.1. Adverse Event 100
8.3.2. Medically Attended Adverse Events 100
8.3.3. Adverse Events of Special Interest 101
8.3.4. Serious Adverse Events 104
8.3.5. Solicited Adverse Reactions (Part A) 105
8.3.6. Recording and Follow-up of Pregnancy 108
8.3.7. Recording and Follow-up of an AE and/or SAE 109
8.3.8. Time Period and Frequency for Collecting AE and SAE Information 109
8.3.9. Assessment of Intensity 109
8.3.10. Assessment of Causality 110
8.3.11. Reporting Adverse Events 111
8.3.12. Regulatory Reporting Requirements for SAEs 111
8.4. Monitoring Committees 112
8.4.1. Protocol Safety Review Team 112
8.4.2. Data and Safety Monitoring Board 112
8.4.3. Adjudication Committee 113
8.4.4. Independent Cardiac Event Adjudication Committee 113
8.5. Management of Overdose 114
8.6. Pharmacokinetics 114
8.7. Pharmacodynamics 114
8.8. Exploratory Assessments and Biomarkers 114
8.9. Medical Resource Utilization and Health Economics 114
9. Statistical Considerations 115

9.1. Blinding and Responsibility for Analyses 115
9.2. Statistical Hypotheses 115
9.3. Sample Size Determination 116
9.3.1. Power for Selected Secondary Efficacy Endpoints 118
9.4. Analysis Populations 119
9.5. Statistical Analyses 120
9.5.1. Efficacy Analyses 120
9.5.2. Safety Analyses 126
9.5.3. Immunogenicity Analyses 127
9.5.4. Exploratory Analyses in Part A 130
9.5.5. Subgroup Analyses 130
9.6. Interim Analyses 131
10. References 133
11. Supporting Documentation and Operational Considerations 137
11.1. APPENDIX 1: Schedules of Events 138
11.2. APPENDIX 2: Study Governance Considerations 160
11.2.1. Regulatory and Ethical Considerations 160
11.2.2. Study Monitoring 160
11.2.3. Audits and Inspections 162
11.2.4. Financial Disclosure 162
11.2.5. Recruitment Procedures 162
11.2.6. Informed Consent Process 162
11.2.7. Protocol Amendments 163
11.2.8. Protocol Deviations 164
11.2.9. Data Protection 164
11.2.10. Sample Retention and Future Biomedical Research 165
11.2.11. Data Quality Assurance and Quality Control 166
11.2.12. Data Collection and Management 167
11.2.13. Source Documents 167
11.2.14. Retention of Records 168
11.2.15. Study and Site Closure 168
11.2.16. Publication Policy 169

11.3. APPENDIX 3: Contraceptive Guidance 170
11.4. APPENDIX 4: Statistical Appendices 172
11.4.1. Estimands and Estimand Specifications 172
11.4.2. Statistical Methods and Sensitivity Analyses 175
11.5. APPENDIX 5: Adverse Events of Special Interest Terms 176
11.6. APPENDIX 6: Protocol Amendment History 178

LIST OF TABLES

Table 1: Objectives and Endpoints 54
Table 2: Summary of Treatment Groups 72
Table 3: Grading of COVID-19 Symptoms 92
Table 4: Maximum Planned Immunogenicity Blood Sampling Volumes per Participant by Visit 99
Table 5: Solicited Adverse Reactions and Grades 106
Table 6: Conditions and Sample Size to Demonstrate Vaccine Efficacy 117
Table 7: Power of Demonstrating Vaccine Efficacy Against Severe COVID-19 118
Table 8: Populations for Analyses 119
Table 9: Statistical Analysis Methods of Efficacy Endpoints 120
Table 10: Treatment Cohorts for the Long-term Efficacy Analyses 124
Table 11: Long-term Efficacy Endpoints 125
Table 12: Analysis Strategy for Safety Parameters in Part A 127
Table 13: Immunogenicity Endpoints and Statistical Methods 128
Table 14: Burden of Disease Score 130
Table 15: Interim Boundaries Using O’Brien-Fleming Spending Function, Calculation Based on the PP Set for the Primary Efficacy Endpoint 132
Table 16: Schedule of Events (Vaccination Phase, Day 1 – Day 57) 139
Table 17: Schedule of Events (Surveillance Phase, Day 64 – Day 394) 142
Table 18: Schedule of Events (Surveillance Phase, Day 401 – Day 759) 145
Table 19: Schedule of Events (Convalescent Period, Starting With the Illness Visit) 147
Table 20: Part B: Participant Decision Visit 149
Table 21: Supplemental Schedule of Events: Open-Label Observational Phase - Placebo Participants who Request to Receive mRNA-1273 150
Table 22: Modified Supplemental Schedule of Events: Open-Label Observational Phase – mRNA-1273 Participants Who ONLY Received 1 Dose of mRNA-1273 153
Table 23: Part C Supplemental Schedule of Events: Booster Dose Phase 156
Table 24: Intercurrent Event Types 172
Table 25: Primary Objective and Estimands With Rationale for Strategies to Address Intercurrent Events for Per-protocol Analysis 173
Table 26: Summary of Statistical Methods and Sensitivity Analyses 175
Table 27: Adverse Events of Special Interest 176

LIST OF FIGURES

Figure 1: Study Flow Diagram: Part A, the Blinded Phase Followed by Part B, the Open-Label Phase Including Part C, the Booster Dose Phase 35
Figure 2: Part B, the Open-Label Observational Phase, and Part C, the Booster Dose Phase 36
Figure 3: Surveillance for COVID-19 Symptoms and the Corresponding Clinical Data Pathways 90
Figure 4: Boundary Crossing Probabilities by Effect Size 117

LIST OF ABBREVIATIONS

The following abbreviations and terms are used in this study protocol.

Abbreviation	Definition
Ab	antibody
AC	adjudication committee
AE	adverse event
AESI	adverse event of special interest
AR	adverse reaction
bAb	binding antibody
BD	booster dose
BLA	Biologics License Application
BOD	burden of disease
BP	blood pressure
CDC	US Centers for Disease Control and Prevention
CEAC	Cardiac Event Adjudication Committee
CFR	Code of Federal Regulations
CI	confidence interval
CLIA	Clinical Laboratory Improvement Amendments
cMRI	cardiac magnetic resonance imaging
CONSORT	Consolidated Standards of Reporting Trials
CoV	coronavirus
COVID-19	coronavirus disease 2019
COV-INF	virologically confirmed SARS-CoV-2 infection or COVID-19 regardless of symptomatology or severity
CP	convalescent plasma
CRO	contract research organization
CSR	clinical study report
DHHS	Department of Health and Human Services
DMID	Division of Microbiology and Infectious Diseases
DSMB	Data and Safety Monitoring Board
DSPC	1,2-distearoyl-sn-glycero-3-phosphocholine
ECG/EKG	electrocardiogram
eCRF	electronic case report form
eDiary	electronic diary
EUA	Emergency Use Authorization
FAS	full analysis set

Abbreviation	Definition
FDA	Food and Drug Administration
FSH	follicle-stimulating hormone
GCP	Good Clinical Practice
GMFR	geometric mean fold rise
GMT	geometric mean titer
HCP	healthcare practitioner
HIV	human immunodeficiency virus
HR	hazard ratio
HRT	hormonal replacement therapy
IA	interim analysis
IB	investigator’s brochure
ICF	informed consent form
ICH	International Council for Harmonisation
IM	intramuscular
IP	investigational product
IRB	institutional review board
IRT	interactive response technology
LLOQ	lower limit of quantification
LNP	lipid nanoparticle
LOD	limit of detection
LTFU	lost to follow-up
MAAE	medically attended adverse event
mAb	monoclonal antibody
MedDRA	Medical Dictionary for Regulatory Activities
MERS-CoV	Middle East respiratory syndrome coronavirus
mITT	modified intent-to-treat
mRNA	messenger RNA
nAb	neutralizing antibody
NIAID	National Institute of Allergy and Infectious Diseases
NP	nasopharyngeal
PCR	polymerase chain reaction
PDV	participant decision visit
PEG2000-DMG	1-monomethoxypolyethyleneglycol-2,3-dimyristylglycerol with polyethylene glycol of average molecular weight 2000
PP	per-protocol

Abbreviation	Definition
PSRT	Protocol Safety Review Team
RT-PCR	reverse transcriptase polymerase chain reaction
S	Spike
S-2P	spike protein with 2 proline residues introduced for stability in a prefusion conformation
SAE	serious adverse event
SAP	statistical analysis plan
SARS-CoV-2	severe acute respiratory syndrome coronavirus 2
SM-102	heptadecan-9-yl 8-((2-hydroxyethyl) (6-oxo-6-(undecyloxy) hexyl) amino) octanoate
SoE	Schedule of Events
SRR	seroresponse rate
TEAE	treatment-emergent adverse event
ULOQ	upper limit of quantification
USP	United States Pharmacopeia
VE	vaccine efficacy
WHO	World Health Organization

2. INTRODUCTION

2.1. Study Rationale

Coronaviruses (CoVs) are a large family of viruses that cause illness ranging from the common cold to more severe diseases, such as Middle East respiratory syndrome CoV (MERS-CoV) and severe acute respiratory syndrome CoV (SARS-CoV).

An outbreak of the CoV disease 2019 (COVID-19) caused by SARS-CoV-2 began in Wuhan, Hubei Province, China in December 2019 and has spread throughout China and to over 216 other countries and territories, including the United States (WHO 2020). On 11 Mar 2020, the World Health Organization (WHO) officially declared COVID-19 a pandemic. As of 28 May 2020, the WHO reported more than 5,593,631 confirmed cases and 353,334 deaths globally and the US Centers for Disease Control and Prevention (CDC) reported 1,698,523 confirmed and probable cases of COVID-19, with 100,446 deaths in the United States (CDC 2020a). The CDC have reported that the highest risk of disease burden is in older adults (≥ 65 years old) and people of any age who have serious underlying medical conditions, such as chronic lung disease or moderate to severe asthma; serious heart conditions; severe obesity; diabetes; chronic kidney disease requiring dialysis; liver disease; and those who are immunocompromised (CDC 2020b).

At the time the present Phase 3 study was initiated, no vaccine against SARS-CoV-2 had been authorized or approved. Global efforts to evaluate novel antivirals and therapeutic strategies to treat severe SARS-CoV-2 infections have intensified, but no proven therapeutic currently exists. Therefore, there is an urgent public health need for rapid development of novel interventions to prevent the spread of this disease. The primary goal of this Phase 3 study is to evaluate the vaccine efficacy (VE) of mRNA-1273 to prevent COVID-19, compared to placebo.

2.2. Background and Overview

ModernaTX, Inc. (the Sponsor) has developed a rapid response, proprietary vaccine platform based on a messenger RNA (mRNA) delivery system. The platform is based on the principle and observations that cells in vivo can take up mRNA, translate it, and then express protein viral antigen(s) on the cell surface. The delivered mRNA does not enter the cellular nucleus or interact with the genome, is nonreplicating, and is expressed transiently. mRNA vaccines have been used to induce immune responses against infectious pathogens such as cytomegalovirus (NCT03382405), human metapneumovirus and parainfluenza virus type 3 (NCT03392389), Zika virus (NCT03325075), and influenza virus (NCT03076385 and NCT03345043).

The Sponsor is using its mRNA-based platform to develop a novel lipid nanoparticle (LNP)-encapsulated mRNA-based vaccine against SARS-CoV-2 (mRNA-1273). mRNA-1273 encodes for the full-length spike (S) protein of SARS-CoV-2, modified to introduce 2 proline residues to stabilize the S protein (S-2P) in a prefusion conformation. The CoV S protein mediates

attachment and entry of the virus into host cells (by fusion), making it a primary target for neutralizing antibodies (nAbs) that prevent infection (Johnson et al 2016; Wang et al 2015; Wang et al 2018; Chen et al 2017; Corti et al 2015; Yu et al 2015; Kim et al 2019; Widjaja et al 2019). It has been confirmed that the stabilized SARS-CoV-2 S-2P antigen presents in the correct prefusion conformation (Wrapp et al 2020).

The development of the mRNA-1273 vaccine is being accelerated to address the current SARS-CoV-2 outbreak as a result of the uniquely rapid and scalable manufacturing process for mRNA-1273. The primary goal of this Phase 3 study is to evaluate the VE of mRNA-1273 to prevent COVID-19, compared to placebo.

2.2.1. Nonclinical Studies

Nonclinical studies have demonstrated that CoV S proteins are immunogenic and S protein-based vaccines, including those based on mRNA delivery platforms, are protective in animals. Prior clinical studies of vaccines targeting related CoVs and other viruses have demonstrated that mRNA-based vaccines are safe and immunogenic. It is therefore anticipated that mRNA-1273 will generate robust immune responses to the SARS-CoV-2 S protein and will be well tolerated. In addition, mRNA-1273 has shown preliminary evidence of protection against SARS-CoV-2 in a murine model of infection (data on file).

In support of development of mRNA-1273 for prophylaxis against SARS-CoV-2 infection, nonclinical immunogenicity, biodistribution, and safety studies have been completed with similar mRNA-based vaccines formulated in LNPs containing SM-102 (heptadecan-9-yl 8-((2 hydroxyethyl)(6 oxo 6-(undecyloxy)hexyl)amino)octanoate), the novel proprietary lipid used in the mRNA-1273 LNP formulation.

A detailed review of nonclinical experience with mRNA-1273 vaccine is provided in the investigator's brochure (IB).

2.2.2. Clinical Studies

The mRNA-1273 vaccine is currently being evaluated for safety and immunogenicity in a dose-ranging Phase 1 study (NCT04283461) sponsored and conducted by the Division of Microbiology and Infectious Diseases (DMID) of the National Institute of Allergy and Infectious Diseases (NIAID). The Phase 1 DMID study is an open-label dose-ranging study of mRNA-1273 in healthy adult male and non-pregnant female participants in 3 age groups: age 18 to 55 years, inclusive (45 participants); age 56 to 70 years, inclusive (30 participants); and ≥ 71 years (30 participants). Each participant received an intramuscular (IM) injection (0.5 mL) of mRNA-1273 on Days 1 and 29 in the deltoid muscle and will be followed for 13 months after the second injection. Sera obtained from 41 participants who were convalescing from COVID-19 were included as a comparative control.

A total of 120 participants were enrolled, and 116 participants received the second injection. Three mRNA-1273 dose levels (25, 50, and 100 µg) administered 28 days apart have been assessed in participants 18 to 55 years, 56 to 70 years, and ≥ 71 years of age. The mRNA-1273 250-µg dose was not evaluated in participants 56 to 70 years and ≥ 71 years of age due to reactogenicity observed in 4 participants in the 250 µg (18 to 55 years) dose cohort. Across all age groups, solicited adverse reactions (ARs) were predominantly mild or moderate in severity and most commonly included fatigue, chills, headache, myalgia, and pain at the injection site. The incidence and severity of solicited ARs were generally dose dependent and increases in incidence and severity were generally observed after the second injection. The incidence of systemic reactions increased after the second injection, particularly at the highest (250 µg) dose. Four (27%) participants in the 250 µg dose cohort reported at least 1 severe solicited AR after the second injection, including feverishness, fatigue, fever, headache, myalgia, nausea, and erythema/redness. No serious adverse events (SAEs) were reported through Day 119, and no pause rules were triggered during the study. mRNA-1273 induced robust binding antibody (bAb) responses after 2 injections. Notably, 100 µg of mRNA-1273 resulted in numerically higher S-2P-specific binding titers than 25 µg and 50 µg of mRNA-1273 in all age groups. Two injections of mRNA-1273 also induced robust nAb responses. The nAb response was marginal after the first injection but increased substantially after the second injection. The nAb response assessed by a pseudovirus neutralization assay at the 100 µg mRNA-1273 dose level was similar to that at the 250 µg mRNA-1273 dose level in the 18 to 55 years of age group and numerically higher than that observed at the 25 µg and 50 µg mRNA-1273 dose levels in the older age groups. The nAb response at the 100 µg dose level was similar across all age groups.

mRNA-1273-P201 (Study 201; NCT04405076) is an ongoing Phase 2a, safety, reactogenicity, and immunogenicity study in healthy adults that provided confirmation of the immunogenicity of both the 100 µg and 50 µg doses. The study was designed as a randomized, observer-blind, placebo-controlled dose confirmation study (Part A). Two mRNA-1273 dose levels, 50 µg and 100 µg, and placebo were evaluated in 2 age cohorts: Cohort 1 enrolled participants ≥ 18 to < 55 years old (300 participants), and Cohort 2 enrolled participants ≥ 55 years old (300 participants). A total of 600 participants received either mRNA-1273 or placebo according to a 1:1:1 randomization ratio; ie, within each age cohort, 100 participants each received mRNA-1273 50 µg, mRNA-1273 100 µg, or placebo. mRNA-1273 demonstrated an acceptable safety profile in the participant population enrolled in this study at both dose levels and both age cohorts, as observed through 6 months after the second injection.

An amendment to the Study 201 protocol adapted the study design to include open-label interventional phases (Part B and Part C). Part B allowed unblinding of participants and offered 2 injections of mRNA-1273 in an open-label manner, 28 days apart, to all participants who received placebo in Part A. Part B also offered a single booster dose (BD) of mRNA-1273 (50 µg)

to participants who received 1 or 2 doses of mRNA-1273 (50 µg or 100 µg) in Part A at least 6 months prior. Part C was prompted by the need to proactively prepare for vaccination strategies that induce broader protection, including against emerging variants of SARS-CoV-2 such as B.1.351. Part C enrolled participants from Study 301 who received 2 doses of mRNA-1273 100 µg at least 6 months prior. Part C participants received a single injection of mRNA-1273.351 (20 µg or 50 µg) or mRNA-1273.351 mixture (50 µg total – 25 µg of mRNA-1273 and 25 µg of mRNA-1273.351).

Vaccination with mRNA-1273 in Study 201 resulted in robust immune responses to SARS-CoV-2 in participants 18 years and older at both dose levels, and persistence of immune response was observed up to 6 months after the second injection in Part A of the study. In Part A of Study 201, the time course and magnitude of antibody (Ab; both bAb and nAb) responses to mRNA-1273 was similar between 100 µg and 50 µg dose levels at each postbaseline timepoint (Days 29, 43, 57, and 209), although the 100 µg dose group had numerically greater responses. In Part B of Study 201, administration of a 50 µg BD of mRNA-1273 6 months or more after the primary series improved the immune responses to 1.7-fold the peak achieved after the primary vaccination series in the current mRNA-1273-P301 study, where efficacy of mRNA-1273 against COVID-19 was demonstrated.

This present ongoing Phase 3 study (mRNA-1273-P301) is a pivotal Phase 3, efficacy, safety, and immunogenicity study that has provided the primary clinical evidence of VE and safety (see Section 4 for details of the study design). The study was designed as a randomized, observer and participant blind, placebo-controlled study of the efficacy, safety, and immunogenicity of mRNA-1273 compared to placebo. More than 30,000 participants 18 years of age and older were randomized 1:1 to mRNA-1273 100 µg or placebo based on 3 strata: ≥ 65 years of age, 18 to < 65 years of age and at increased risk for complications of COVID-19, and 18 to < 65 years of age and not at risk. The initial mRNA-1273 100 µg dose was followed by a second 100 µg dose 28 days later. Participants are being followed for efficacy and safety until 24 months after the second dose. Vaccine efficacy was demonstrated based on the prespecified efficacy success criterion at the interim analysis (IA) (11 Nov 2020 dataset), based on a total of 95 adjudicated COVID-19 cases. The subsequent primary analysis of efficacy was performed with a total of 196 adjudicated COVID-19 cases (25 Nov 2020 dataset) and was consistent with the IA. mRNA-1273 was subsequently granted emergency use authorization (EUA) in the US and conditional approvals worldwide.

After EUA in the US was granted for mRNA-1273 and another mRNA COVID-19 vaccine, Part B, the Open-Label Observational Phase of the study was initiated. All participants in Part A were invited to proceed to Part B, starting with a participant decision visit (PDV) at the study site, at which participants were given the option to be unblinded to their original group assignment or

remain blinded. Unblinded participants who had received placebo in Part A had the choice to be vaccinated with mRNA-1273 in Part B.

On 25 Aug 2021, Moderna announced that it had completed the rolling submission process for its Biologics License Application (BLA) to the US Food and Drug Administration (FDA) for the full licensure of the Moderna COVID-19 vaccine (mRNA-1273) for active immunization to prevent COVID-19 in individuals 18 years of age and older. mRNA-1273 showed durable efficacy of 93% through 6 months after the second dose. mRNA-1273 was highly immunogenic as measured by both bAb and nAb in both SARS-CoV-2 baseline-negative and baseline-positive individuals, as indicated by increased bAb and nAb levels 1 month after first injection (Day 29) and 1 month after second injection (Day 57). mRNA-1273 demonstrated an acceptable safety profile in the participant population enrolled in this study, having a reactogenicity profile consistent with parenteral vaccination and was generally well tolerated. No unexpected findings were identified in the final assessment of the randomized, blinded phase of the study (Part A).

Protocol mRNA-1273-P301 Amendment 9 initiated Part C of the study (Section 4.1.3), providing for administration of a BD of mRNA-1273.

2.3. Benefit/Risk Assessment

2.3.1. Potential Benefits of Study Participation

The target study population for this study is adults with no known history of SARS-CoV-2 infection but whose locations or circumstances put them at high risk of COVID-19. The following benefits may accrue to participants:

- The mRNA-1273 vaccine may be an effective vaccine against COVID-19.
- A baseline (Day 1) evaluation for SARS-CoV-2 infection and ongoing surveillance for COVID-19 throughout the study.
- Contributing to the development of a vaccine against COVID-19, a current pandemic disease.

2.3.2. Risks From Study Participation

Immediate systemic allergic reactions (eg, anaphylaxis) can occur following any vaccination. These reactions are very rare and are estimated to occur once per 450,000 vaccinations for vaccines that do not contain allergens such as gelatin or egg protein (Zent et al 2002). As a precaution, all participants will remain under observation at the study site for at least 30 minutes after vaccination.

Vasovagal syncope (fainting) can occur before or after any vaccination, is usually triggered by the pain or anxiety caused by the injection, and is not related to the substance injected. Therefore, it is important that standard precautions and procedures be followed to avoid injury from fainting.

Intramuscular injection with other mRNA vaccines manufactured by the Sponsor containing the SM-102 lipid formulation commonly results in a transient and self-limiting local inflammatory reaction. This typically includes pain, erythema (redness), or swelling (hardness) at the injection site, which are mostly mild to moderate in severity and usually occur within 24 hours of vaccination. More severe but self-limited local reactions, erythema and induration, have been observed at doses of mRNA-1273 exceeding the dose proposed in this study.

Most systemic ARs observed after vaccination do not exceed Grade 1 to Grade 2 in severity. The most commonly reported systemic ARs are anticipated to be fever, fatigue, chills, headache, myalgias, and arthralgias. More severe reactions, including erythema, induration, fever, headache, and nausea were reported after receiving doses of mRNA-1273 that were greater than the dose proposed for use in this study. In all cases, the reactions resolved spontaneously.

Laboratory abnormalities (including increases in liver functional tests and serum lipase levels) following vaccination were observed in clinical studies with similar mRNA-based vaccines. These abnormalities were without clinical symptoms or signs and returned toward baseline (Day 1) values over time. The clinical significance of these observations is unknown. Further details are provided in the current IB.

There is a theoretical risk that active vaccination to prevent the novel viral infection caused by SARS-CoV-2 may cause a paradoxical increase in the risk of disease. This possibility is based on the rare phenomenon of vaccine-associated disease enhancement which was first seen in the 1960s with 2 vaccines made in the same way (formalin-inactivated whole virus) and designed to protect children against infection with respiratory syncytial virus (Chin et al 1969) or measles (Fulginiti et al 1967). Disease enhancement has also been proposed as a possible explanation for cases of more serious disease associated with dengue vaccination (Thomas and Yoon 2019; WHO 2019).

To monitor the risk of enhanced disease in this study, an independent Data and Safety Monitoring Board (DSMB) will review unblinded cases of COVID-19 to assess for inefficacy and also for numerical imbalance in cases of both COVID-19 and severe COVID-19 with the purpose of providing a non-binding recommendation to the Sponsor (Section 8.4.2). To date, clinical immunogenicity data from the DMID Phase 1 study of mRNA-1273 demonstrated high levels of nAbs and Th-1-polarized CD4+ T-cell responses (Jackson et al 2020). In addition, in Study P301, after a median follow-up of 2 months after the second dose of vaccine, the overwhelming majority of COVID-19 cases occurred in participants who received placebo rather than mRNA-1273 (Baden et al 2020), consistent with a low risk of enhanced respiratory disease following vaccination with mRNA-1273. These data suggest that a paradoxical increase in the risk of disease, while not eliminated, is likely to be low. Further details are provided in the current IB.

In the context of the EUA for individuals 18 years and older for mRNA-1273, there have been very rare reports of myocarditis and pericarditis occurring after vaccination with Moderna

COVID-19 vaccine. Further details about this risk are described in the Fact Sheets (available on www.fda.gov). Although causality has not been established, the majority of the cases have been reported in young males shortly after the second dose of the vaccine. These are typically mild cases and individuals tend to recover within a short time following standard treatment and rest (Gargano et al 2021).

The adverse events (AEs) after getting a third dose of mRNA-1273 are currently being studied. It is unknown if the AEs after getting a third dose of mRNA-1273 are different from getting 2 doses of mRNA-1273. Based on cumulative evidence, the benefit-risk profile of a BD of mRNA-1273 is favorable, particularly in light of increasing breakthrough disease with the emergence of variants.

2.3.3. Overall Benefit/Risk Conclusion

All participants will be included based on their increased risk of SARS-CoV-2 infection. Accordingly, all will benefit from baseline and ongoing evaluations for SARS-CoV-2 infection.

Since this is a placebo-controlled study (Section 4), half the participants will have the potential to receive mRNA-1273 vaccine, the efficacy of which is unknown at present. Vaccination with mRNA-1273 may not prevent COVID-19 in all vaccinees.

Participants who receive placebo as part of this study may have an opportunity cost of not being treated with another investigational vaccine against COVID-19.

The placebo for this study is a saline solution, without any LNP. Thus, participants receiving saline may be at lower risk of AEs related to injection than participants receiving mRNA-1273.

Safety findings will be monitored and periodically reviewed by the DSMB to evaluate the safety and treatment status of all participants. The DSMB will review and assess the safety data as described in Section 8.4.2.

Considering the lack of approved vaccines for COVID-19 prior to initiation of the study, the participants’ risk of COVID-19 outside the study, and the nonclinical and clinical data to date, the Sponsor considers the potential benefits of participation to exceed the risks.

Based on the interim results from the pivotal Phase 3 study, mRNA-1273 prevents COVID-19 and severe COVID-19. The demonstrated clinical benefit of mRNA-1273 is supported by evidence of a robust immune response both in terms of bAbs and nAbs as well as the induction of CD4+ T-cells with a Th-1 dominant phenotype. Based on administration of mRNA-1273 to approximately 15,693 adults across all 3 clinical studies to date, there have been no emergent safety concerns and the AE profile is manifested largely by mild to moderate reactogenicity lasting 2 to 3 days. Additionally, interim results of the ongoing Moderna Phase 2a study (mRNA-1273-P201) demonstrate a favorable benefit-risk profile of a BD of mRNA-1273.

3. OBJECTIVES AND ENDPOINTS

Table 1: Objectives and Endpoints

Objectives and Endpoints	
**Primary Objective**	**Primary Endpoints**
**Efficacy Objective (Primary):**<br>To demonstrate the efficacy of mRNA-1273 to prevent COVID-19.	**Efficacy Endpoints (Primary):** Vaccine efficacy of mRNA-1273 to prevent the first occurrence of COVID-19 starting 14 days after the second dose of IP, where COVID-19 is defined as symptomatic disease based on the following criteria:<br>• The participant must have experienced at least TWO of the following systemic symptoms: fever (≥ 38ºC), chills, myalgia, headache, sore throat, new olfactory and taste disorder(s), OR<br>• The participant must have experienced at least ONE of the following respiratory signs/symptoms: cough, shortness of breath or difficulty breathing, OR clinical or radiographical evidence of pneumonia; AND<br>• The participant must have at least 1 NP swab or saliva sample (or respiratory sample, if hospitalized) positive for SARS-CoV-2 by RT-PCR
**Safety Objective (Primary):**<br>To evaluate the safety and reactogenicity of 2 injections of the mRNA-1273 vaccine given 28 days apart.	**Safety Endpoint (Primary):**<br>• Solicited local and systemic ARs through 7 days after each dose of IP (Part A).<br>• Unsolicited AEs through 28 days after each dose of IP (Part A [all participants] and Part C [only those who receive the BD]).<br>• MAAEs or AEs leading to withdrawal through the entire study period.<br>• SAEs throughout the entire study period.

**Objectives and Endpoints**	
**Efficacy Objectives (Secondary)**	**Efficacy Endpoints (Secondary)**
To evaluate the efficacy of mRNA-1273 to prevent severe COVID-19	Vaccine efficacy of mRNA-1273 to prevent severe COVID-19, defined as first occurrence of COVID-19 starting 14 days after the second dose of IP, (as per the primary endpoint) AND any of the following:<br>• Clinical signs indicative of severe systemic illness, respiratory rate ≥ 30 per minute, heart rate ≥ 125 beats per minute, $SpO_2 \leq 93\%$ on room air at sea level or $PaO_2/FIO_2 < 300$ mmHg, OR<br>• Respiratory failure or acute respiratory distress syndrome (defined as needing high-flow oxygen, non-invasive or mechanical ventilation, or extracorporeal membrane oxygenation), evidence of shock (systolic BP < 90 mmHg, diastolic BP < 60 mmHg or requiring vasopressors), OR<br>• Significant acute renal, hepatic, or neurologic dysfunction, OR<br>• Admission to an intensive care unit or death
To evaluate the efficacy of mRNA-1273 to prevent serologically confirmed SARS-CoV-2 infection or COVID-19 regardless of symptomatology or severity.	Vaccine efficacy of mRNA-1273 to prevent the first occurrence of either COVID-19 or SARS-CoV-2 infection starting 14 days after the second IP dose.<br>This endpoint is a combination of COVID-19, defined as for the primary endpoint, and asymptomatic SARS-CoV-2 infection, determined by seroconversion assessed by bAb levels against SARS-CoV-2 as measured by a ligand-binding assay specific to the SARS-CoV-2 nucleocapsid protein and with a negative NP swab sample for SARS-CoV-2 at Day 1 (Section 8.1.1).

Objectives and Endpoints	
**Efficacy Objectives (Secondary)**	**Efficacy Endpoints (Secondary)**
To evaluate VE against a secondary definition of COVID-19.	Vaccine efficacy of mRNA-1273 to prevent the secondary case definition of COVID-19 starting 14 days after the second IP dose.<br>The secondary case definition of COVID-19 is defined as the following systemic symptoms: fever (temperature ≥ 38ºC), or chills, cough, shortness of breath or difficulty breathing, fatigue, muscle aches or body aches, headache, new loss of taste or smell, sore throat, nasal congestion or rhinorrhea, nausea or vomiting, or diarrhea, AND a positive NP swab or saliva sample (or respiratory sample, if hospitalized) for SARS-CoV-2 by RT-PCR
To evaluate VE to prevent death caused by COVID-19.	Vaccine efficacy of mRNA-1273 to prevent death due to a cause directly attributed to a complication of COVID-19, starting 14 days after the second IP dose.
To evaluate the efficacy of mRNA-1273 to prevent COVID-19 after the first dose of IP.	Vaccine efficacy of mRNA-1273 to prevent the first occurrence of COVID-19 starting 14 days after the first dose of IP.
To evaluate the efficacy of mRNA-1273 to prevent COVID-19 in all study participants, regardless of evidence of prior SARS-CoV-2 infection.	Vaccine efficacy of mRNA-1273 to prevent the first occurrence of COVID-19 starting 14 days after the second dose of IP regardless of evidence of prior SARS-CoV-2 infection determined by serologic titer against SARS-CoV-2 nucleocapsid (FAS analysis population, see Section 9.4).
To evaluate the efficacy of mRNA-1273 to prevent asymptomatic SARS-CoV-2 infection.	Vaccine efficacy to prevent the first occurrence of SARS-CoV-2 infection in the absence of symptoms defining COVID-19 starting 14 days after the second IP dose. SARS-CoV-2 infection determined by seroconversion assessed by bAb levels against SARS-CoV-2 as measured by a ligand-binding assay specific to the SARS-CoV-2 nucleocapsid protein and with a negative NP swab sample for SARS-CoV-2 at Day 1 (Section 8.1.1).

Objectives and Endpoints	
**Immunogenicity Objective (Secondary):**	**Immunogenicity Endpoints (Secondary):**
To evaluate the immunogenicity of 2 doses of mRNA-1273 given 28 days apart.	• GMT of SARS-CoV-2-specific nAb on Day 1, Day 29, Day 57, Day 209, Day 394, and Day 759.<br>• GMFR of SARS-CoV-2-specific nAb relative to Day 1 on Day 29, Day 57, Day 209, Day 394, and Day 759.<br>• Quantified levels or GMT of S protein-specific bAb on Day 1, Day 29, Day 57, Day 209, Day 394, and Day 759.<br>• GMFR of S protein-specific bAb relative to Day 1 on Day 29, Day 57, Day 209, Day 394, and Day 759.
**Immunogenicity Objective (Secondary) for Part C:**	**Immunogenicity Endpoints (Secondary) for Part C:**
To infer effectiveness of the 50 µg of mRNA-1273 booster by establishing noninferiority of Ab response after the BD compared to the primary series of mRNA-1273 using GMT values of serum Ab and seroresponse rate of post booster in Part C compared with primary series recipients of mRNA-1273.	Key secondary endpoints:<br>• GMT of post-booster (post-BD-Day 1; BD-Day 29) Ab as compared to post-primary series mRNA-1273 (post-Dose 2; Day 57)<br>• Seroresponse rate of post-booster/BD-Day 1 from baseline (pre-Dose 1) as compared to post-Dose 2 from baseline (pre-Dose 1) in primary series recipients of mRNA-1273.<br>Seroresponse is defined as a titer change from baseline (pre-Dose 1 in primary series) below the LLOQ to ≥ 4 × LLOQ, or at least a 4-fold rise if baseline is ≥ LLOQ.
**Exploratory Objectives for Part A**	
To evaluate the effect of mRNA-1273 on the viral infection kinetics as measured by viral load at SARS-CoV-2 infection diagnosis by RT-PCR and number of days from the estimated date of SARS-CoV-2 infection until undetectable SARS-CoV-2 infection by RT-PCR.	
To assess VE to reduce the duration of symptoms of COVID-19.	
To evaluate VE against all-cause mortality.	
To assess VE against burden of disease due to COVID-19.	
To evaluate the genetic and/or phenotypic relationships of isolated SARS-CoV-2 strains to the vaccine sequence.	
To evaluate immune response markers after dosing with IP as correlates of risk of COVID-19 and as correlates of risk of SARS-CoV-2 infection.	

**Objectives and Endpoints**
To conduct additional analyses related to furthering the understanding of SARS-CoV-2 infection and COVID-19, including analyses related to the immunology of this or other vaccines, detection of viral infection, and clinical conduct.
**Exploratory Objectives for Part C**
To evaluate the safety of a BD of mRNA-1273.
To evaluate the immunogenicity of a BD of mRNA-1273.

Abbreviations: Ab = antibody; AE = adverse event; AR = adverse reaction; bAb = binding antibody; BD = booster dose; BP = blood pressure; COVID-19 = coronavirus disease 2019; FAS = full analysis set; GMFR = geometric mean fold rise; GMT = geometric mean titer; IP = investigational product; LLOQ = lower limit of quantification; MAAE = medically attended adverse event; nAb = neutralizing antibody; NP = nasopharyngeal; RT-PCR = reverse transcriptase polymerase chain reaction; S = spike; SAE = serious adverse event; SARS-CoV-2 = severe acute respiratory syndrome coronavirus 2; VE = vaccine efficacy.

4. STUDY DESIGN

4.1. General Design

This is a 3-part Phase 3 study, comprising Part A, Part B, and Part C. Participants in Part A, the Blinded Phase of this study, were blinded to their treatment assignment. Given that the primary efficacy endpoint for mRNA-1273 against COVID-19 was met per the protocol-defined IA, Part B, the Open-Label Observational Phase of this study, was designed to offer eligible participants who received placebo in Part A of this study an option to request open-label mRNA-1273, subject to availability of investigational vaccine (Figure 1). Part C, the Booster Dose Phase, providing for an mRNA-1273 BD, is designed to offer eligible participants the option to request a BD of mRNA-1273.

Upon entry into Part A, all participants had 8 scheduled clinic visits, including Screening, Day 1, Day 29, Day 57, Day 209, Day 394, Day 759, as specified in the Schedules of Events (SoEs) (Table 16, Table 17, Table 18, Table 19). With the initiation of Part B, a PDV was scheduled for all participants currently enrolled in Part A at that time (Table 20) and participants eligible for Part B dosing may have had up to 2 additional scheduled clinic visits as specified in SoEs (Table 21 and Table 22). Part C will begin with a BD-1 visit (see SoE; Table 23) scheduled for all participants currently enrolled in Part B, and participants deciding to receive a BD may have up to 3 additional clinic visits scheduled (Section 4.1.3; Table 23).

This study will be conducted in compliance with the protocol, Good Clinical Practice (GCP), and all applicable regulatory requirements.

4.1.1. Part A, the Blinded Phase

The Blinded Phase of this study is a randomized, stratified, observer-blind, placebo-controlled evaluation of the efficacy, safety, and immunogenicity of mRNA-1273 SARS-CoV-2 vaccine compared to placebo in adults 18 years of age and older who have no known history of SARS-CoV-2 infection but whose locations or circumstances put them at appreciable risk of acquiring COVID-19 and/or SARS-CoV-2 infection. Figure 1 shows the study flow and Appendix 1 (Table 16, Table 17, Table 18, and Table 19) presents the planned SoEs.

Approximately 30,000 participants will be randomly assigned to receive doses of either 100 µg of mRNA-1273 vaccine or a placebo control in a 1:1 randomization ratio. Assignment will be stratified by age and health risk (Section 6.2.1.1). This is a case-driven study and thus final sample size of the study will depend on the actual attack rate of COVID-19.

All participants will be assessed for efficacy and safety endpoints and provide a nasopharyngeal (NP) swab sample and blood sample before the first and second dose of investigational product (IP), in addition to a series of post-dose blood samples for immunogenicity through 24 months

after the second dose of IP. Efficacy assessments will include surveillance for COVID-19 with reverse transcriptase polymerase chain reaction (RT-PCR) confirmation of SARS-CoV-2 infection after the first and second dose of IP. As noted above, this is a case-driven study: if the prespecified criteria for early efficacy are met at the time of either IA or overall efficacy at the primary analysis, a final study report describing the efficacy and safety of mRNA-1273 will be prepared based on the data available at that time. In the event that success criteria are met either at the time of the IA or when the total number of cases toward the primary endpoint have accrued, participants will continue to be followed in a blinded fashion until Month 25 to enable assessment of long-term safety and durability of VE. If the study concludes early, all participants will be requested to provide a final blood sample at the time of study conclusion.

Each participant will receive 2 doses of IP by 0.5 mL IM injection, the first on Day 1 and the second on Day 29. An NP swab sample will be collected prior to the first and second dose of IP for evaluation by RT-PCR. To preserve observer blinding, only delegated unblinded study personnel responsible for study vaccine preparation, administration and/or accountability will have knowledge of study treatment assignment (Section 6.2.8.1).

Participants will be given an electronic diary (eDiary) to report solicited ARs for 7 days after each dose of IP and to prompt an unscheduled clinic visit for clinical evaluation and NP swab sample if a participant experiences any symptoms of COVID-19 (throughout study). Participants will use the eDiary to report solicited ARs for 7 days after each dose of IP and weekly eDiary prompts (every 7 days) to elicit an unscheduled Illness Visit if the participant is experiencing COVID-19 symptoms. All participants will receive safety calls on Day 8, Day 15, Day 22, Day 36, and Day 43 that will serve both to monitor for unsolicited AEs and to monitor for symptoms of COVID-19.

Safety telephone calls and eDiary safety prompts will be performed in conjunction with surveillance for COVID-19 according to the SoEs (Table 16, Table 17, Table 18, and Table 19) and are intended to capture SAEs, medically attended AEs (MAAEs), AEs leading to withdrawal, concomitant medications associated with these events, receipt of non-study vaccinations, and pregnancy (Section 8.2.1). If an eDiary prompt results in identification of a relevant safety event, a follow-up safety call will be triggered.

Surveillance for COVID-19 will be performed through weekly contacts with the participant via a combination of telephone calls and completion of an eDiary starting at Day 1 through the end of the study (Section 8.1.2). Participants with symptoms of COVID-19 lasting at least 48 hours (except for fever and/or respiratory symptoms) will return to the clinic or will be visited at home by medically qualified site staff within 72 hours to collect an NP swab sample for RT-PCR testing for SARS-CoV-2 and other respiratory pathogens, or alternatively, if a clinic or home visit is not possible, will submit a saliva sample for SARS-CoV-2 RT-PCR testing (Section 8.1.1).

All study participants who experience COVID-19 symptoms and subsequently present for an Illness Visit (in-clinic or at home) will be given an instruction card listing symptoms and severity grading system along with a thermometer, an oxygen saturation monitor, and saliva collection tubes. The list of symptoms is presented in Section 8.1.2 and the severity scoring system is presented in Section 8.1.3. Study participants will be contacted by the investigator (or appropriately delegated study staff) daily with telemedicine visits through Day 14 (from the Illness Visit or initial COVID-19 contact) or until symptoms have resolved, whichever is later, and which may include symptoms persisting longer than the 28-day Convalescent Period. If symptoms persist beyond Day 60, then telemedicine calls can be reduced to weekly; however, the participant should still report the daily symptoms at that contact. During the telemedicine visit (preferably done in the evening), the participant will be asked to verbally report the severity of each symptom and their highest body temperature and lowest oxygen saturation for that day, and the investigator will determine if medical attention is required due to worsening of COVID-19 symptoms (Table 19). Study participants will collect their own saliva sample on 3, 5, 7, 9, 14, and 21 days after the initial Illness Visit meeting criteria for COVID-19 (defined as the date of onset of symptoms and positive virologic test). Finally, a Convalescent Visit will be scheduled approximately 28 days after the initial Illness Visit. At this visit, a saliva sample will be collected and a blood sample will be drawn for immunologic assessment of SARS-CoV-2 infection.

At each dosing visit, participants will be instructed (Day 1) or reminded (Day 29) on how to document and report solicited ARs in the eDiary provided. Solicited ARs will be assessed for 7 days after each IP dose and unsolicited AEs will be assessed for 28 days after each IP dose; SAEs, MAAEs, AEs leading to withdrawal, will be assessed throughout the study.

Participants may experience AEs that necessitate an unscheduled visit. There may also be situations in which the investigator asks a participant to report for an unscheduled visit following the report of an AE. Additional examinations may be conducted at these visits as necessary to ensure the safety and well-being of participants during the study. Electronic case report forms (eCRFs) should be completed for each unscheduled visit.

4.1.2. Part B, the Open-Label Observational Phase

The Part B, the Open-Label Observational Phase of the study is prompted by the authorization of a COVID-19 vaccine under EUA. Transitioning the study to Part B permits all ongoing study participants to be informed of the availability and eligibility criteria of any COVID-19 vaccine made available under an EUA and the option to offer all ongoing study participants who request unblinding an opportunity to schedule a PDV to learn their original treatment assignment (placebo vs. mRNA-1273 vaccine).

Part B, the Open-Label Observational Phase of the study, also provides the opportunity for eligible study participants who previously received placebo, to actively request to receive 2 doses of mRNA-1273 vaccine (as long as investigational vaccine is available). All study participants will receive a notification letter and will be asked to schedule a PDV. Principal Investigators should consider current local public health guidance for administration of COVID-19 vaccines under EUA when determining the scheduling priority of participants. With regard to eligibility in Part B, the CDC-EUA guidance specifications will supersede eligibility criteria in the protocol.

All participants will proceed to Part B, the Open-Label Observational Phase of the study, starting with a PDV (Figure 2).

At the PDV, all participants will:

- Be encouraged to remain in the ongoing study,
- Be given the option to be unblinded as to their original group assignment (placebo vs. mRNA-1273 vaccine),
- Be counselled about the importance of continuing other public health measures to limit the spread of disease including social distancing, wearing a mask, and hand-washing,
- Sign a revised informed consent form (ICF),
- Provide an NP swab sample for RT-PCR for SARS-CoV-2 and a blood sample for serology prior to unblinding.

Figure 2 shows the Part B Study Flow schematic.

The following participants will continue with the original study SoEs presented in Table 16, Table 17, Table 18, and Table 19 (Figure 2):

- Participants who request to not be unblinded,
- Participants who request to be unblinded and received mRNA-1273, and
- Participants who request to be unblinded and received placebo and choose to remain on placebo.

Participants who are unblinded, received placebo, are eligible, and request to receive mRNA-1273 (as long as investigational vaccine is available) (Figure 2), will have the following clinic visits as shown in the Supplemental SoE (Table 21):

- Open-label Dose 1 (OL-D1): To occur at the PDV or at a scheduled subsequent visit.
- Open-label Dose 2 (OL-D29): To occur 28 days after Dose 1 on OL-D1.
- Open-label Clinic Visit (OL-D57): To occur 1 month after Dose 2 on OL-D29.

- And in addition, continue to comply with the Original Study SoEs (Table 16, Table 17, Table 18, and Table 19) as applicable.
- Participants who are unblinded, and received ONLY 1 dose of mRNA-1273 may be eligible to receive 1 more dose of mRNA-1273 if: They had a dosing error in Part A of the study that resulted in 1 dose of mRNA-1273 and 1 dose of placebo being administered.
- They did not have an AE that contraindicated the second dose of mRNA-1273 in Part A of the study.
- They did not withdraw consent in Part A of the study.
- They did not complete their second dose in Part A of the study for reasons other than the above.

If participants are eligible, and request to receive mRNA-1273 (Figure 2) will proceed to have the following clinic visits as shown in the Modified Supplemental SoEs (Table 22):

- Their second dose on OL-D1: To occur at the PDV or at a scheduled subsequent visit.
- Open-label Clinic Visit (OL-D29): To occur 1 month after Dose 2 on OL-D1.
- And in addition, continue to comply with the Original Study SoEs (Table 16, Table 17, Table 18, and Table 19) as applicable.

All participants remain on their original SoE to complete 24 months of follow-up after Dose 2; study participants who enter the Supplemental/Modified Supplemental SoE as described above do so in addition to their original SoE. Accordingly, all study participants will complete the full study follow-up to 24 months after the second dose of their original inoculation (mRNA-1273 or placebo) or 25 months after the first dose if the second dose is missed.

At the point when the mRNA-1273 vaccine is no longer available for study use, any participant who schedules a PDV after this timepoint will be unblinded (with no option to stay blinded) but will not be offered to receive mRNA-1273 study vaccine. These participants will have all the same procedures performed as outlined in Table 20 with the exception of being offered to receive mRNA-1273 vaccine. There will be no scientific metric or ethical advantage for participants to remain blinded when most of the study has already been unblinded. If the participant wants to receive EUA vaccine outside of the study after being unblinded, they will need to be withdrawn from the study.

After the BLA database lock (on 04 May 2021), any remaining participants who had not been unblinded were unblinded by the Moderna study team and received their unblinding treatment assignment (placebo vs. mRNA vaccine) via certified letter from the study site. These participants were not required to return to the site for an NP swab sample or blood draw.

4.1.3. Part C, the Booster Dose Phase

Part C, the Booster Dose Phase providing a BD for all eligible participants who chose to receive one, is prompted by interim results of an ongoing Moderna Phase 2a study (mRNA-1273-P201), as described in Section 4.2.

Each study participant will receive a notification letter and will be asked to schedule a BD-1 visit at their study site (Table 23). Principal Investigators should consider current local public health guidance for administration of COVID-19 vaccines under EUA and marketing authorization (if any) when determining the scheduling priority of participants. At the BD-1 visit, each participant will:

- Be encouraged to remain in the ongoing study,
- Sign a revised ICF that includes both updated safety information relevant to the ongoing study and a BD, and the option to receive a BD,
- Be given the option to receive a BD consisting of a 50 µg dose of mRNA-1273,
- Be counselled about the importance of continuing other public health measures to limit the spread of disease including social distancing, wearing a mask, and hand-washing.

After the BD-1 visit, participants who do not receive a BD will continue with the Part C Supplemental SoE for the BD-2 and BD-3 visits but will not have the BD-1a visit or the safety calls (Table 23).

Participants who request a BD and are eligible and have no contraindications to further dosing (see Section 7.1) will have the following study site visits and complete scheduled activities (subject to investigational vaccine availability) according to the Part C Supplemental SoE (Table 23):

- BD-1 visit: Participants will receive a single 50 µg dose of mRNA-1273.
- BD-1a visit: Optional, and subject to blood sample kit availability, Day 4, 3 days after BD on Day 1.
- BD-2 visit: Day 29, 28 days after the BD on Day 1.
- BD-3 visit: Day 181, 180 days after the BD on Day 1.

The investigator is responsible for conducting all assessments as specified in the Part C Supplemental SoE, according to the schedule. As this Supplemental SoE is intended to occur in addition to the original SoEs (Table 16, Table 17, Table 18, and Table 19) being followed by all participants in Part B, there is a possibility for study visits to overlap. If visits overlap according to respective visit windows, a single visit may be done with the combined study procedures completed once (refer to Table 4 footnotes for more detailed instructions and exceptions).

4.2. Scientific Rationale for Study Design

The mRNA-1273 vaccine is being developed to prevent COVID-19, the disease resulting from SARS-CoV-2 infection. The present study has been designed to primarily evaluate the clinical efficacy and safety of mRNA-1273 to prevent COVID-19 for up to 2 years after the planned second dose of mRNA-1273. The immunogenicity endpoints and detection of SARS-CoV-2 infection are secondary objectives.

The design and focus of the study as designed for Part A were dependent on the then current COVID-19 pandemic, requiring identification of participant candidates at high risk of SARS-CoV-2 infection. This Phase 3 study was designed to be a randomized, stratified, observer-blind, placebo-controlled study to evaluate the efficacy, safety, and immunogenicity of mRNA-1273 SARS-CoV-2 vaccine compared to placebo in adults 18 years of age and older who had no known history of SARS-CoV-2 infection but whose locations or circumstances put them at appreciable risk of acquiring COVID-19 and/or SARS-CoV-2 infection.

Following authorization of a COVID-19 vaccine under EUA, the study design was amended to include a transition to Part B, the Open-Label Observational Phase (Section 2.2.2). The demonstration of efficacy against COVID-19 with satisfactory safety data, at a time when the COVID-19 pandemic remained critical, warranted allowing those study participants who actively requested it to know their original assignment (placebo or mRNA-1273 vaccine) on study. Transitioning the study to the Open-Label Observational Phase permitted all ongoing study participants (a) to be informed of the availability and eligibility criteria of any COVID-19 vaccine made available under an EUA and (b) to schedule a study visit to know their original group assignment (placebo vs. mRNA-1273 vaccine). Part B also provided the opportunity for eligible study participants who previously received placebo to receive 2 doses of mRNA-1273 vaccine under open-label conditions. Prompted by the anticipated recommendations from the FDA and CDC regarding boosters, this study design was further amended to include a transition to Part C, the Open-Label Observational BD Phase. The interim results of an ongoing Moderna Phase 2a study (mRNA-1273-P201; NCT04405076), in which participants who, 6 to 8 months prior, received 2 doses of 50 µg or 100 µg of mRNA-1273 were administered a 50 µg booster of mRNA-1273. Results demonstrated enhanced immune responses compared to pre-boost levels and met the noninferiority criteria stipulated in the FDA Guidance on EUA for Vaccines to Prevent COVID-19 (DHHS 2021). In addition, no new safety signals emerged upon administration of the BD in Study mRNA-1273-P201. Based on cumulative evidence, the benefit-risk profile of a BD of mRNA-1273 is favorable, particularly in light of increasing breakthrough disease with the emergence of variants. With the wider availability of boosters under EUA, providing the option of a BD to all eligible participants currently enrolled in Part B is expected to promote retention of participants in the ongoing study and thereby defend the scientific integrity of the study for the

planned 2-year duration of follow-up after the completion of the primary vaccination series regimen.

4.3. Choice of Dose and Control Product

The primary vaccination series originally selected for Part A of this study, 2 doses of 100 µg mRNA-1273 administered 28 days apart, was based on assessment of available safety and immunogenicity data from Phase 1 studies of mRNA-1647 (NCT03382405), the DMID study 20-0003 entitled “Phase I, Open-Label, Dose-Ranging Study of the Safety and Immunogenicity of 2019-nCoV Vaccine (mRNA-1273) in Healthy Adults” (NCT04283461) and Study 201 (Section 2.2.2). The primary vaccination series (at the 100-µg dose level) used in this study is being used in these studies, with evidence of safety and immunogenicity. At the time Part A was initiated, there was no licensed SARS-CoV-2 vaccine currently available to serve as a reference control; accordingly, 0.9% sodium chloride (normal saline) injection (United States Pharmacopeia [USP]) was used as a placebo control during Part A.

For Part C, the provision of the option of a single BD for eligible participants during the Open-Label Observational Phase, the BD selected is 50 µg, based primarily on data from Study mRNA-1273-P201. In Part A of Study mRNA-1273-P201, the time course and magnitude of Ab (both bAb and nAb) responses to mRNA-1273 was similar between 100 µg and 50 µg dose levels at each postbaseline timepoint (Days 29, 43, 57, and 209), although the 100-µg dose group had numerically greater responses. In Part B of Study mRNA-1273-P201, administration of a 50 µg BD of mRNA-1273 6 months or more after the primary series improved the immune responses to 1.7-fold the peak achieved after the primary vaccination series in the current mRNA-1273-P301 study, where efficacy of mRNA-1273 against COVID-19 was demonstrated.

4.4. End of Study Definition

Participants are considered to have completed the study if they complete the final visit at Day 759 (Month 25), 24 months following their receipt of the original second dose of IP (where original refers to the IP received following randomization); if a participant did not receive a second dose of IP, their follow-up will be 25 months after first dose of IP. For participants who enter Part C, if visit BD-3 falls after Day 759 (Month 25), then BD-3 will be the final visit.

The study duration will be approximately 26 months for each participant. This includes a screening period of up to 1 month and a study period of 25 months that includes the first dose of IP on Day 1 and the second dose on Day 29 (Part A). The participant's final scheduled visit will be on Day 759 (Month 25), 24 months after the second dose of IP on Day 29 (Month 1) or 25 months after the first dose if the second dose is missed. For participants who enter Part C, if visit BD-3 falls after Day 759 (Month 25), then BD-3 will be the final visit.

The end of study will be the final participant’s final scheduled visit at Day 759 (Month 25) or BD-3, whichever is later.

5. STUDY POPULATION

The study population, adults at risk of SARS-CoV-2 infection who have no known history of SARS-CoV-2 infection, is a subset of the planned target population. Additionally, potential study participants at increased risk of complications from COVID-19 will be included since it is hypothesized that these participants might derive the greatest benefit from a vaccine. Participants ≥ 65 years of age will be eligible for enrollment with or without underlying medical conditions further increasing their risk of severe COVID-19.

Given the disproportionate disease burden of COVID-19 in racial and ethnic minorities, the study will also aim to enroll a representative sample of participants from these minority population and adjust site selection and enrollment accordingly, per FDA Draft Guidance “Enhancing the Diversity of Clinical Trial Populations - Eligibility Criteria, Enrollment Practices, and Trial Designs” (DHHS 2020b). Study sites may be selected on the basis of SARS-COV-2 infection risk of the local population as well. Approximately 30,000 participants will be enrolled.

There will be no prospective approval of protocol deviations to recruitment and enrollment criteria (also known as protocol waivers or exemptions).

5.1. Inclusion and Exclusion Criteria (Part A, Part B, and Part C)

5.1.1. Inclusion Criteria

Participants are eligible to be included in the study, or further dosing (Part B and Part C), only if all the following criteria apply:

1. (Part A only) Adults, ≥ 18 years of age at time of consent, who are at high risk of SARS-CoV-2 infection, defined as adults whose locations or circumstances put them at appreciable risk of exposure to SARS-CoV-2 and COVID-19
2. Understands and agrees to comply with the study procedures and provides written informed consent.
3. Able to comply with study procedures based on the assessment of the investigator.
4. Female participants of nonchildbearing potential may be enrolled in the study. Nonchildbearing potential is defined as surgically sterile (history of bilateral tubal ligation, bilateral oophorectomy, hysterectomy) or postmenopausal (defined as amenorrhea for ≥ 12 consecutive months prior to Screening without an alternative medical cause). A follicle-stimulating hormone (FSH) level may be measured at the discretion of the investigator to confirm postmenopausal status (see additional information in Appendix 11.3).

5. Female participants of childbearing potential may be enrolled in the study if the participant fulfills all the following criteria:

   - Has a negative pregnancy test at Screening and on the day of the first dose (Day 1, OL-D1, and BD-1).
   - Has practiced adequate contraception or has abstained from all activities that could result in pregnancy for at least 28 days prior to the first dose (Day 1, OL-D1, and BD-1).
   - Has agreed to continue adequate contraception through 3 months following the last dose (Day 29, OL-D29, and BD-1).
   - Is not currently breastfeeding.

   Adequate female contraception is defined as consistent and correct use of an FDA approved contraceptive method in accordance with the product label. For example:

   - Barrier method (such as condoms, diaphragm, or cervical cap) used in conjunction with spermicide
   - Intrauterine device
   - Prescription hormonal contraceptive taken or administered via oral (pill), transdermal (patch), subdermal, or IM route
   - Sterilization of a female participant's monogamous male partner prior to entry into the study

   Note: periodic abstinence (eg, calendar, ovulation, symptothermal, post-ovulation methods) and withdrawal are not acceptable methods of contraception.

6. Healthy adults or adults with pre-existing medical conditions who are in stable condition. A stable medical condition is defined as disease not requiring significant change in therapy or hospitalization for worsening disease during the 3 months before enrollment.
7. (Part C Only) Is currently enrolled in Part B of the current study (mRNA-1273-P301).
8. (Part C Only) Has received at least 1 dose of mRNA-1273 in the current study (mRNA-1273-P301).

5.1.2. Exclusion Criteria

Participants are excluded from the study or further dosing (Part B and Part C) if any of the following criteria apply:

1. Is acutely ill or febrile 72 hours prior to or at Screening or dosing (Part B and Part C). Fever is defined as a body temperature ≥ 38.0°C/100.4°F. Participants meeting this criterion may be rescheduled within the relevant window periods. Afebrile participants with minor illnesses can be enrolled/dosed at the discretion of the investigator.
2. Is pregnant or breastfeeding.
3. (Part A Only) Known history of SARS-CoV-2 infection.
4. Prior (Part A) or concurrent (Part B and Part C) administration of a non-study coronavirus (SARS-CoV, MERS-CoV) vaccine or current/planned simultaneous participation in another interventional study to prevent or treat COVID-19
5. (Part A Only) Demonstrated inability to comply with the study procedures.
6. An immediate family member or household member of this study's personnel.
7. Known or suspected allergy or history of anaphylaxis, urticaria, or other significant AR to the vaccine or its excipients.
8. Bleeding disorder considered a contraindication to IM injection or phlebotomy.
9. Has received or plans to receive a non-study vaccine within 28 days prior to or after any dose of IP (except for seasonal influenza vaccine which is not permitted within 14 days before or after any dose of IP, see Section 6.4.3).
10. (Part A only) Has participated in an interventional clinical study within 28 days prior to the day of enrollment.
11. Immunosuppressive or immunodeficient state, asplenia, recurrent severe infections (human immunodeficiency virus [HIV]-positive participants with CD4 count ≥ 350 cells/mm^3 and an undetectable HIV viral load within the past year [low level variations from 50-500 viral copies which do not lead to changes in antiretroviral therapy are permitted]).
12. Has received systemic immunosuppressants or immune-modifying drugs for > 14 days in total within 6 months prior to IP dose administration (for corticosteroids ≥ 20 mg/day of prednisone equivalent).
13. Has received systemic immunoglobulins or blood products within 3 months prior to the day of IP dose administration.
14. Has donated ≥ 450 mL of blood products within 28 days prior to IP dose administration.

5.2. Participant Restrictions

Participants must not eat or drink anything hot or cold within 10 minutes before oral temperature is taken.

5.3. Screen Failures (Part A, Blinded Phase Only)

Screen failures are defined as participants who consent to participate in the clinical study but are not subsequently randomly assigned to treatment. A minimum set of screen failure information is required to ensure transparent reporting of screen failures to meet the Consolidated Standards of Reporting Trials (CONSORT) publishing requirements and to respond to queries from regulatory authorities. Minimum information includes date of informed consent, demography, screen failure details, eligibility criteria, and information on any SAE that may have occurred from the time informed consent was obtained to the time of withdrawal.

Participants meeting the exclusion criterion #1, acutely ill or febrile prior to or at the Screening Visit (exclusion criterion #1, Section 5.1.2), may be rescheduled within the relevant window periods and will retain their initially assigned participant number.

6. STUDY TREATMENT

6.1. Investigational Product

The term "investigational product" refers to both mRNA-1273 vaccine (blinded and open-label) and placebo administered in this study.

The mRNA-1273 IP is an LNP dispersion of an mRNA encoding the prefusion stabilized S protein of SARS-CoV-2 formulated in LNPs composed of 4 lipids (1 proprietary and 3 commercially available): the proprietary ionizable lipid SM-102; cholesterol; 1,2-distearoyl-sn-glycero-3-phosphocholine (DSPC); and 1-monomethoxypolyethyleneglycol-2,3-dimyristylglycerol with polyethylene glycol of average molecular weight 2000 (PEG2000-DMG). The mRNA-1273 vaccine is provided as a sterile liquid for injection and is a white to off-white dispersion in appearance, at a concentration of 0.2 mg/mL in 20 mM Tris buffer containing 87 mg/mL sucrose and 10.7 mM sodium acetate at pH 7.5.

The placebo is 0.9% sodium chloride (normal saline) injection, which meets the criteria of the USP.

6.2. Dosing and Management of Investigational Product

6.2.1. Method of Randomly Assigning Participants to Treatment Groups (Part A, Blinded Phase Only)

Approximately 30,000 participants will be randomly assigned in 1:1 ratio to receive either mRNA-1273 100 µg or placebo. The randomization will be in a blinded manner using a centralized interactive response technology (IRT), in accordance with pre-generated randomization schedules. Only the unblinded personnel (Section 6.2.8.1) will have controlled access to which arm the participant is randomly assigned.

Dose group assignment in is summarized in Table 2.

Table 2: Summary of Treatment Groups

Treatment Groups	Investigational Product	Age (years)	Estimated Total Participants
mRNA-1273	mRNA-1273 100 µg	≥ 18	15,000
Placebo	Placebo	≥ 18	15,000
**Total**			**30,000**

6.2.1.1. Stratification

Randomization in Part A, Blinded Phase of the study will be stratified based on age and, if they are < 65 years of age, based on the presence or absence of risk factors for severe illness from COVID-19 based on CDC recommendation as of March 2020 (CDC 2020b). There will be 3 strata

for randomization: ≥ 65 years, < 65 years and categorized to be at increased risk ("at risk") for the complications of COVID-19, and < 65 years "not at risk." Risk will be defined based on the study participants' relevant past and current medical history. At least 25% of enrolled participants, up to 50%, will be either ≥ 65 years of age or < 65 years of age and "at risk" at Screening.

Participants who are < 65 years old will be categorized as at risk for severe COVID-19 illness if they have at least 1 of the following risk factors at Screening:

- Chronic lung disease (eg, emphysema and chronic bronchitis), idiopathic pulmonary fibrosis and cystic fibrosis) or moderate to severe asthma
- Significant cardiac disease (eg, heart failure, coronary artery disease, congenital heart disease, cardiomyopathies, and pulmonary hypertension)
- Severe obesity (body mass index ≥ 40 kg/m^2)
- Diabetes (Type 1, Type 2, or gestational)
- Liver disease
- HIV infection

6.2.2. Administration of Investigational Product

In Part A and Part B, as applicable, IP will be administered as an IM injection into the deltoid muscle on a 2-dose injection schedule on Day 1 and Day 29. For injections administered for Part A and Part B, each injection will have a volume of 0.5 mL and contain mRNA-1273 100 µg or saline placebo. For Part C, each injection will have a volume of 0.25 mL and contain mRNA-1273 50 µg. Preferably, vaccine should be administered into the nondominant arm. The second dose of IP should be administered in the same arm as the first dose (Part A and Part B, as applicable).

The IP will be prepared for injection as a single 0.5 mL (Part A and Part B) or 0.25 mL (Part C) dose for each participant per protocol, as detailed in the Pharmacy Manual. Unblinded personnel who will not participate in any other aspect of the study during Part A, the Blinded Phase, will perform IP accountability, dose preparation, and IP administration. The investigator will designate unblinded medically qualified personnel (not involved in assessments of study endpoints) to administer the IP according to the procedures stipulated in this study protocol (Part A, the Blinded Phase), and Pharmacy Manual. Study-specific training will be provided. Study site personnel who were blinded during the Blinded Phase will be unblinded at the participant level at the PDV.

At each visit when IP is administered, participants will be monitored for a minimum of 30 minutes after administration. Assessments will include vital sign measurements and monitoring for local or systemic reactions (Table 16, Table 21, Table 22, and Table 23).

Eligibility for a subsequent dose of IP is determined by following the criteria outlined in Section 7.

The study site will be appropriately staffed with individuals with basic cardiopulmonary resuscitation training/certification. Either on-site resuscitation equipment and personnel or appropriate protocols for the rapid transport of participant to a resuscitation area/facility are required.

6.2.3. Delivery and Receipt

The Sponsor or designee is responsible for the following:

- Supplying the IP
- Confirming the appropriate labeling of mRNA-1273 IP, so that it complies with the legal requirements of the US

The investigator is responsible for acknowledging the receipt of the IP by a designated staff member at the site, including the following:

- Confirming that the IP was received in good condition
- Confirmation that the temperature during shipment from the Sponsor to the investigator's designated storage location was appropriate
- Confirming whether the Sponsor has authorized the IP for use
- Ensuring the appropriate dose level of mRNA-1273 is properly prepared using aseptic technique

Further description of the IP and instructions for the receipt, storage, preparation, administration, accountability, and destruction of the IP are described in the mRNA-1273-P201 Pharmacy Manual.

6.2.4. Packaging and Labeling

The Sponsor will provide the investigator and study site with adequate quantities of mRNA-1273. The sterile vaccine product is packaged in a 10R glass vial with a 5.0-mL or 6.3-mL fill volume (Part A and Part B) or a 10R glass vial with an 8.0 mL fill volume (Part C). mRNA-1273 vaccine will have all required labeling per regulations and will be supplied to the pharmacy in an unblinded manner. Each vial will be individually labeled for future participant identification purposes.

mRNA-1273 will be packaged and labeled in accordance with the standard operating procedures of the Sponsor or of its designee, Code of Federal Regulations Title 21 (CFR), Good Manufacturing Practice guidelines, International Council for Harmonisation (ICH) GCP guidelines, guidelines for Quality System Regulations, and applicable regulations.

The Sponsor or Sponsor's designee will supply the 0.9% sodium chloride injection for use as both a placebo and a diluent to mRNA-1273. The 0.9% sodium chloride bears a commercial label and does not contain study-specific identification (Part A only).

6.2.5. Storage

The vials of mRNA-1273 with 5.0 ml fill volume must be stored at 2°C to 8°C in a secure area with limited access (unblinded personnel only) and protected from moisture and light until it is prepared for administration (Section 6.2.2). The vials of mRNA-1273 with 6.3 mL or 8.0 mL fill volume must be stored at -20°C in a secure area with limited access and protected from moisture and light until it is prepared for administration. The vials of mRNA-1273 with 6.3 mL fill volume can be stored at 2°C to 8°C for up to 30 days, but site personnel must account for this manually as it will not be managed in the IRT system. The freezer or refrigerator should have automated temperature recording and a 24-hour alert system in place that allows for rapid response in case of freezer or refrigerator malfunction. There must be an available backup freezer or refrigerator. The freezers or refrigerators must be connected to a backup generator. In addition, vaccine accountability study staff (eg, the unblinded personnel) are required to keep a temperature log to establish a record of compliance with these storage conditions. The site is responsible for reporting any mRNA-1273 that was not temperature controlled during shipment or during storage to the unblinded site monitor. Such mRNA-1273 will be retained for inspection by the unblinded monitor and disposed of according to approved methods.

The 0.9% sodium chloride injection (USP) should be stored at 20ºC to 25ºC (68ºF to 77ºF) in a restricted access area (applicable to Part A only).

6.2.6. Investigational Product Accountability

It is the investigator's responsibility that the unblinded personnel maintain accurate records in an IP accountability log of receipt of all IP, inventory at the site, dispensing of mRNA-1273 and placebo, IP injections, and return to the Sponsor or alternative disposition of used/unused products.

An unblinded site monitor will review the inventory and accountability log during site visits and at the completion of the study. Additional details are found in the mRNA-1273-P301 Pharmacy Manual.

6.2.7. Handling and Disposal

An unblinded site monitor will reconcile the IP during the conduct and at the end of the study for compliance. Once fully reconciled at the site at the end of the study, the IP can be destroyed at the investigational site or at a Sponsor-selected third party, as appropriate.

Investigational product may be destroyed at the study site only if permitted by local regulations and authorized by the Sponsor. A Certificate of Destruction must be completed and sent to the Sponsor or designee.

6.2.8. Unblinding

6.2.8.1. Planned Unblinding

See Section 4.1.2 regarding the PDV.

Part A, the Blinded Phase of this study is observer-blind. The investigator, study staff, study participants, site monitors, and Sponsor personnel (or its designees) will be blinded to the IP administered, with the following exceptions:

- Unblinded personnel (of limited number) will be assigned to vaccine accountability procedures and will prepare IP for all participants. These personnel will have no study functions other than study vaccine management, documentation, accountability, preparation, and administration. They will not be involved in participant evaluations and will not reveal the identity of IP to either the participant or the blinded study site personnel involved in the conduct of the study unless this information is necessary in the case of an emergency.
- Unblinded medically qualified study site personnel will administer the IP. They will not be involved in assessments of any study endpoints.
- The dosing assignment will be concealed by having the unblinded personnel prepare the IP in a secure location that is not accessible or visible to other study staff. An opaque sleeve over the syringe used for injection will maintain the blind at the time of injection, as the doses containing mRNA-1273 will look different than placebo. Only delegated unblinded site staff will conduct the injection procedure. Once the injection is completed, only the blinded study staff will perform further assessments and interact with the participants. Access to the randomization code will be strictly controlled at the pharmacy.
- Unblinded site monitors, not involved in other aspects of monitoring, will be assigned as the IP accountability monitors. They will have responsibilities to ensure that sites are following all proper IP accountability, preparation, and administration procedures.
- An unblinded statistical and programming team will perform the pre-planned IAs (Section 9.6).
- An independent DSMB will review the interim data to safeguard the interests of clinical study participants and to help ensure the integrity of the study. The DSMB will review unblinded statistical outputs and IA results, provided by the independent unblinded statistician, and make recommendations to the Sponsor (Section 8.4.2).

- At the initiation of Part B, the Open-Label Observational Phase of this study (Section 4.1.3), study site personnel who were blinded during the Blinded Phase will be unblinded at the participant level at the PDV.
- If prespecified criteria for early efficacy are met by an IA or if the primary efficacy analysis is completed based on accrual of prespecified COVID-19 cases, pre-identified Sponsor and contract research organization (CRO) team members responsible for the analysis and reporting will be unblinded to treatment assignments in order to prepare a final study report. In order to maintain an observer-blind design, investigators, site staff, participants, and Sponsor and CRO staff with oversight of study conduct will remain blinded to treatment allocation for the study duration. All study participants will be followed for efficacy and safety endpoints through the remainder of planned study period and results will be summarized in an end of study report (Sections 4.1 and 9.1).

6.2.8.2. Unplanned Unblinding

A participant or participants may be unblinded in the event of an SAE or other severe event, or if there is a medical emergency requiring the identity of the product to be known to properly treat a participant. If a participant becomes seriously ill or pregnant during the study, the study investigator may request that the blind will be broken if knowledge of the administered vaccine will affect that participant’s dosing options. In this situation or in the event of a medical emergency requiring identification of the IP administered to an individual participant, the investigator will make every attempt to contact the Sponsor medical lead to explain the need for opening the code within 24 hours of opening the code. The investigator will be responsible for documenting the time, date, reason for the code break, and the names of the personnel involved.

In addition to the situations described above where the blind may be broken, the data will also be unblinded to a statistical team at specified timepoints for IAs as outlined in Section 9.6.

In December 2020, COVID-19 vaccines started to become available under EUA as an alternative option to some participants based on evolving recommended populations by the CDC and local supply chain distribution. While all participants should be encouraged to stay blinded in the study for as long as possible, their participation should not otherwise deny them the opportunity to receive a COVID-19 vaccine under EUA. Clinical investigators can exercise discretion as to whether individual participants should be unblinded upon request to allow them to make an informed decision regarding receipt of a COVID-19 vaccine outside of this study. Investigator judgment should consider a participant’s risk status under CDC recommendations, any current local public health guidance, and their access to imminently receive a COVID-19 vaccine under an EUA.

If a decision is made to unblind a participant to support an informed decision to receive a COVID-19 vaccine under an EUA outside of this study, investigators are asked to take the following steps:

- Obtain a final assessment of safety from the participant to collect and resolve any outstanding safety experience. Verbal interview is sufficient.
- Unblind the participant in the IRT and inform the participant of their treatment assignment.
- Inform the Sponsor Medical Lead within 24 hours to confirm rationale for unblinding.

Participants who are confirmed to have received placebo upon unblinding and intend to receive a COVID-19 vaccine under an EUA outside of this study will be withdrawn from the study at the point of unblinding (Section 7.3.1).

Note: Participants who requested unblinding because of a stated imminent access to a COVID-19 vaccine under an EUA outside of this study, were withdrawn from study, and then were unable to in fact receive a COVID-19 vaccine, will be permitted to re-enter this study upon re-consent to receive the mRNA-1273 vaccine once they are eligible (as long as investigational vaccine is available).

6.3. Study Treatment Compliance

All doses of IP will be administered at the study site under direct observation of unblinded medically qualified study personnel and appropriately recorded (date and time) in the eCRF. Unblinded personnel will confirm that the participant has received the entire dose of vaccine. If a participant does not receive vaccine or does not receive all of the planned doses, the reason for the missed dose will be recorded. Data will be reconciled with site accountability records to assess compliance.

Participants who miss the second dose of IP due to noncompliance with the visit schedule and not due to a safety pause will still be required to follow the original visit and testing schedule as described in the protocol. Except for participants who withdraw consent or who are withdrawn from the study due to receipt of non-study COVID-19 primary series or first booster vaccine (Section 7.3.1), a participant who withdraws or is withheld from receiving the second dose of study vaccine in Part A or Part B will remain in the study and complete all efficacy, safety, and immunogenicity assessments required through the participant's scheduled end of study.

The study site is responsible for ensuring that participants comply with the study windows allowed. If a participant misses a visit, every effort should be made to contact the participant and complete a visit within the defined visit window (SoE Tables, Section 11.1). If a participant does not complete a visit within the time window, that visit will be classified as a missed visit (with the

exception of Dose 2 visits in Part A and Part B, as applicable) and the participant will continue with subsequent scheduled study visits. All safety requirements of the missed visit will be captured and included in the subsequent visit (eg, clinical laboratory testing, eDiary review for reactogenicity, immunologic testing, as applicable).

6.4. Prior and Concomitant Therapy

6.4.1. Prior Medications and Therapies

Information about prior medications (including any prescription or over-the-counter medications, vaccines, or blood products) taken by the participant within the 28 days before providing informed consent (or as designated in the inclusion/exclusion requirements) will be recorded in the participant's eCRF.

6.4.2. Concomitant Medications and Therapies

Study site staff must question the participant regarding any medications taken and vaccinations received by the participant and record the following information in the eCRF:

- All non-study vaccinations administered within the period starting 28 days before the first dose of IP.
- Seasonal influenza vaccine administered for the current influenza season (typically October through April in the Northern Hemisphere).
- All concomitant medications and non-study vaccinations taken through 28 days after each dose of IP. Antipyretics and analgesics taken prophylactically (ie, taken in the absence of any symptoms in anticipation of an injection reaction) will be recorded as such.
- Any concomitant medications used to prevent or treat COVID-19.
- Any concomitant medications relevant to or for the treatment of an SAE or an MAAE.
- Part A: Participant will be asked in the eDiary if they have taken any antipyretic or analgesic to treat or prevent fever or pain within 7 days after each IP injection, including the day of dosing. Reported antipyretic or analgesic medications should be recorded in the source document by the site staff during the post-injection study visits or via other participant interactions (eg, phone calls).

6.4.3. Concomitant Medications and Vaccines That May Lead to the Elimination of a Participant From Per-protocol Analyses

The use of the following concomitant medications and/or vaccines will not require withdrawal of the participant from the study but may determine a participant's eligibility to receive a second dose or evaluability in the per-protocol (PP) analysis (Analysis Sets are described in Section 9.4):

- Any investigational or nonregistered product (drug or vaccine) other than the study vaccine used during the study period.
- A non-study vaccine administered during the period from 28 days before through 28 days after each dose of IP or any seasonal influenza vaccine that was administered within 14 days before or after any dose of IP.
- Immunoglobulins and/or any blood products administered during the study period (except for treatment of COVID-19).
- Medications that suppress the immune system (except for treatment of COVID-19).

In addition, any participant confirmed to have received or plans to receive a non-study COVID-19 vaccine, either licensed or under EUA, may also not be included in the PP analysis (and may also be withdrawn from the study per Section 7.3.1 if non-study COVID-19 vaccine is primary series or first booster).

If a participant takes a prohibited drug therapy, the investigator and the CRO's medical monitor will make a joint decision about continuing or withholding further dosing from the participant based on the time the medication was administered, the drug's pharmacology and pharmacokinetics, and whether use of the medication will compromise the participant's safety or the interpretation of data. It is the investigator's responsibility to ensure that details regarding the concomitant medications are adequately recorded in the eCRF.

All medication and interventions necessary for the appropriate care for the study participant, particularly to treat COVID-19, should be administered and appropriately documented along with the AE.

7. DELAYING OR DISCONTINUING STUDY TREATMENT AND PARTICIPANT WITHDRAWAL FROM THE STUDY

7.1. Criteria for Delay of Study Treatment

Body temperature must be measured at the Day 1, Day 29, OL-D1, OL-D29, and BD-1 visits prior to any study treatment administration.

The following events constitute criteria for delay of study treatment, and if any of these events occur at the time scheduled for dosing, the participant may be injected at a later date within the time window specified in Table 16, Table 21, Table 22, and Table 23, or the participant may be discontinued from dosing at the discretion of the investigator (Section 7.2):

- Acute moderate or severe infection with or without fever at the time of dosing.
- Fever, defined as body temperature ≥ 38.0°C (100.4°F) at the time of dosing.
- Part B only: Diagnosed COVID-19 by detection of SARS-CoV-2 by RT-PCR and accompanying symptoms. Participant must be asymptomatic at the time of dosing, although they may still be RT-PCR positive, with the exception of fatigue and loss of sense of smell or taste, if by the investigator's judgement these are deemed to be mild chronic sequelae.
- Part C only: Seasonal influenza vaccine that was administered within 14 days of the BD-1 visit.
- Part C only: Diagnosed COVID-19 by detection of SARS-CoV-2 by RT-PCR and accompanying symptoms after completion of the primary vaccination series in Part A or Part B. For all study participants who were confirmed to have COVID-19 infection after Dose 1 and Dose 2 of mRNA-1273 but before the booster, the BD-1 visit should be delayed for 90 days from the receipt of monoclonal Ab (mAb) or convalescent plasma (CP) if given as a part of treatment per CDC recommendations. For participants who did not receive mAb or CP, BD can be administered after a minimum of 30 days from the onset of COVID-19 symptoms and complete resolution of the acute illness (except fatigue and loss of sense of smell or taste). A delay of 90 days may be considered, if feasible. If these criteria delay the BD-1 visit date beyond the allowable window per the study timeline, the subject may not receive the dose as a part of the study.

Participants with a minor illness without fever, as assessed by the investigator, can be administered IP. Participants with a fever of 38.0°C (100.4°F) or higher will be contacted within the time window acceptable for participation and reevaluated for eligibility. If the investigator determines that the participant's health on the day of administration temporarily precludes dosing with IP, the visit should be rescheduled within the allowed interval for that visit.

7.2. Discontinuation of Study Treatment

Every reasonable attempt will be made to follow up with participants for safety throughout the entire study period, even if further dosing is discontinued or the participant misses one or more visits. Except for participants who withdraw consent or who are withdrawn from the study due to receipt of non-study COVID-19 primary series or first booster vaccine (Section 7.3.1), a participant who withdraws or is withheld from receiving the second dose of study vaccine in Part A or Part B will be considered to have discontinued treatment but will remain in the study and complete all scheduled visits and assessments (Section 11.1). Except for participants who withdraw consent or who are withdrawn from the study due to receipt of non-study COVID-19 primary series or first booster vaccine (Section 7.3.1), a participant who does not receive the BD in Part C will NOT be considered to have discontinued treatment and will remain in the study and complete all scheduled visits and assessments.

The investigator, in consultation with the Sponsor's medical monitor, may withhold a participant from further dosing if the participant experiences any of the following:

- Becomes pregnant (Section 8.3.6)
- Develops, during the course of the study, symptoms or conditions listed in the exclusion criteria
- Experiences an AE (other than reactogenicity) after dosing that is considered by the investigator to be related to IP (Section 8.3.10) and is of Grade 3 (severe) or greater intensity (Section 8.3.9)
- Experiences an AE or SAE that, in the judgment of the investigator, requires study IP withdrawal due to its nature, severity, or required treatment, regardless of the causal relationship to vaccine
- Experiences a clinically abnormal vital sign measurement or finding on physical examination, or general condition that, in the judgment of the investigator, requires IP withdrawal

The reason(s) for discontinuation from further dosing will be recorded in the eCRF.

Prior to receiving a second dose of study vaccine in Part A or Part B and prior to receiving a BD in Part C (as applicable), participants will be reassessed to ensure that they continue to meet eligibility requirements as outlined below.

The following events in a participant constitute absolute contraindications to any further dosing of the IP to that participant. If any of these events occur during the study, the participant must not receive additional doses of vaccine but will be encouraged to continue study participation for safety through end-of-study period as defined in Section 4.4.

- Part A only: Diagnosed COVID-19 by detection of SARS-CoV-2 in a Day 1 NP swab sample or COVID-19 diagnosed prior to Day 29. If COVID-19 is suspected on or prior to Day 29, further administration of IP must be withheld until COVID-19 test results are available.
- Anaphylaxis or systemic hypersensitivity reaction following the administration of vaccine.
- Any SAE judged by investigator or Sponsor to be related to study vaccine.
- Any clinically significant medical condition that, in the opinion of the investigator, poses an additional risk to the participant if he/she continues to participate in the study.

7.3. Participant Withdrawal From the Study

7.3.1. Participant Withdrawal

Participants who withdraw from the study will not be replaced. A "withdrawal" from the study refers to a situation wherein a participant does not return for the final visit planned in the protocol. The statistical management of participant withdrawals is discussed in Section 9.

Participants can withdraw consent and withdraw from the study at any time, for any reason, without prejudice to further treatment the participant may need to receive. The investigator will request that the study participant complete all study procedures pending at the time of withdrawal.

A participant who withdraws consent or who is withdrawn from the study may request destruction of any samples taken and not tested, and the investigator must document this in the site study records. If the participant withdraws consent for disclosure of future information, the Sponsor may retain and continue to use any data collected before such a withdrawal of consent (see Section 11.2.10).

The Sponsor will continue to retain and use all research results that have already been collected for the study evaluation, unless the participant has requested destruction of these samples. All biological samples that have already been collected may be retained and analyzed at a later date (or as permitted by local regulations).

If participant desires to withdraw from the study because of an AE, the investigator will try to obtain agreement to follow up with the participant until the event is considered resolved or stable and will then complete the end of study eCRF.

Information related to the withdrawal will be documented in the eCRF. The investigator will document whether the decision to withdraw a participant from the study was made by the participant or by the investigator, as well as which of the following possible reasons was responsible for withdrawal:

- AE (specify)
- SAE (specify)
- Death
- Lost to follow-up (LTFU)
- Physician decision (specify)
- Pregnancy
- Protocol deviation
- Study terminated by Sponsor
- Withdrawal of consent by participant (specify); this includes participants who, at the PDV, withdraw consent from continuing in the study.
- Other (specify)

Participants who are withdrawn from the study because of AEs (including SAEs) must be clearly distinguished from participants who are withdrawn for other reasons. Investigators will follow up with participants who are withdrawn from the study as result of an SAE or AE until resolution of the event.

Participants who are confirmed to have received placebo upon unblinding and intend to receive a COVID-19 vaccine either licensed or under an EUA outside of this study will be withdrawn from the study. In addition, any participant confirmed to have received or plans to receive a non-study COVID-19 vaccine for their primary series or first booster only, either licensed or under EUA, will be withdrawn from the study. If a participant has an ongoing AE or pregnancy at the time of receipt of a non-study COVID-19 primary series or first booster vaccine, the participant will remain in the study to allow follow-up until the resolution of the event. Participants receiving a second or subsequent non-study COVID-19 boosters will remain in the study and follow all study procedures.

7.4. Lost to Follow-up

Participants will be considered LTFU if they repeatedly fail to return for scheduled visits without stating an intention to withdraw consent and they cannot be contacted by the study site. The following actions must be taken if a participant fails to return to the clinic for a required study visit:

- The site must attempt to contact the participant and reschedule the missed visit as soon as possible, counsel the participant on the importance of maintaining the assigned visit schedule, and ascertain whether the participant wishes to and/or should continue in the study.

- Before a participant is deemed LTFU, the investigator or designee must make every effort to regain contact with the participant (where possible, 3 telephone calls and, if necessary, a certified letter to the participant’s last known mailing address or local equivalent methods). These contact attempts (eg, dates of telephone calls and registered letters) should be documented in the participant’s medical record. A participant should not be considered LTFU until these efforts have been made.
- Should the participant continue to be unreachable, he/she will be considered to have withdrawn from the study.

8. STUDY ASSESSMENTS AND PROCEDURES

Before performing any study procedures, all potential participants will sign an ICF (as detailed in Section 11.2.6). Participants will undergo study procedures at the timepoints specified in the SoEs (Section 11.1).

A participant also can be seen for an unscheduled visit at any time during the study. An unscheduled visit may be prompted by reactogenicity issues, Illness Visit criteria for COVID-19, or new or ongoing AEs. The site also has the discretion to make reminder phone calls or send text messages to inform the participant about visits, review eDiary requirements, or follow-up on ongoing or outstanding issues.

In accordance with "FDA Guidance on Conduct of Clinical Trials of Medical Products during the COVID-19 Pandemic" (DHHS 2020a), investigators may convert study site visits to telemedicine visits with the approval of the Sponsor. Such action should be taken to protect the safety and well-being of study participants and study site staff or to comply with state or municipal mandates.

General considerations for study assessments and procedures include the following:

- Protocol waivers or exemptions are not allowed. The study procedures and their timing must be followed as presented in Section 11.1. Adherence to the study design requirements is essential and required for study conduct.
- Immediate safety concerns should be discussed with the Sponsor immediately upon occurrence or awareness to determine if the participant should continue study treatment or participation in the study.
- All screening evaluations must be completed and reviewed to confirm that potential participants meet all eligibility criteria. The investigator will maintain a screening log to record details of all participants screened and to confirm eligibility or record reasons for screening failure, as applicable.
- Procedures conducted as part of the participant's routine clinical management (eg, blood count) and obtained before signing of the ICF may be utilized for screening or baseline purposes provided the procedures met the protocol-specified criteria and were performed within the time frame defined in the SoE.

8.1. Efficacy and Immunogenicity Assessments and Procedures

8.1.1. Efficacy Assessments Related to COVID-19 and SARS-CoV-2 Infection

Each study participant will have an NP swab sample collected for SARS-CoV-2 testing by RT-PCR on Day 1 and Day 29, prior to receiving a dose of the IP as specified in the SoE (Section 11.1).

COVID-19:

To be considered as a case of COVID-19 for the evaluation of the Primary Efficacy Endpoint, the following criteria must be met:

- The participant must have experienced at least TWO of the following systemic symptoms: fever (≥ 38ºC), chills, myalgia, headache, sore throat, new olfactory and taste disorder(s), OR
- The participant must have experienced at least ONE of the following respiratory signs/symptoms: cough, shortness of breath or difficulty breathing, OR clinical or radiographical evidence of pneumonia; AND
- The participant must have at least 1 NP swab or saliva sample (or respiratory sample, if hospitalized) positive for SARS-CoV-2 by RT-PCR.

Severe COVID-19:

To be considered severe COVID-19, the following criteria must be met:

- Confirmed case of COVID-19 as per the Primary Efficacy Endpoint case definition, plus any of the following:
    - Clinical signs indicative of severe systemic illness, respiratory rate ≥ 30 per minute, heart rate ≥ 125 beats per minute, SpO_2 ≤ 93% on room air at sea level or PaO_2/FIO_2 < 300 mmHg, OR
    - Respiratory failure or acute respiratory distress syndrome (defined as needing high-flow oxygen, non-invasive or mechanical ventilation, or extracorporeal membrane oxygenation), evidence of shock (systolic blood pressure [BP] < 90 mmHg, diastolic BP < 60 mmHg or requiring vasopressors), OR
    - Significant acute renal, hepatic, or neurologic dysfunction, OR
    - Admission to an intensive care unit or death.

The secondary case definition of COVID-19 is defined as having any of the following systemic symptoms: fever (temperature ≥ 38ºC), or chills, cough, shortness of breath or difficulty breathing, fatigue, muscle aches, or body aches, headache, new loss of taste or smell, sore throat, nasal

congestion or rhinorrhea, nausea, or vomiting or diarrhea, AND a positive NP swab or saliva sample (or respiratory sample, if hospitalized) for SARS-CoV-2 by RT-PCR.

Death attributed to COVID-19 is defined as any participant who dies during the study with a cause directly attributed to a complication of COVID-19.

SARS-CoV-2 Infection:

- SARS-CoV-2 infection is defined by seroconversion due to infection measured by bAb against SARS-CoV-2 nucleocapsid. Seroconversion is defined as follows for participants who are seronegative at Baseline:
    - Binding antibody levels against SARS-CoV-2 nucleocapsid either below the limit of detection (LOD) or lower limit of quantification (LLOQ) at Study Day 1 that increase to above or equal to LOD or LLOQ starting at Study Day 57 or later.

8.1.2. Surveillance for COVID-19 Symptoms

Surveillance for COVID-19 symptoms will be conducted by a combination of safety phone calls and eDiary completion as presented in the SoEs in Section 11.1, in addition to study site visits (Figure 3). If there is no response to an eDiary prompt for 2 days, the site staff will contact the study participant by phone.

According to the CDC as of 10 Jun 2020 (CDC 2020c), patients with COVID-19 have reported a wide range of symptoms ranging from mild symptoms to severe illness. Throughout the study, to surveil for COVID-19, the following prespecified symptoms that meet the criteria for suspicion of COVID-19 will be elicited weekly from the participant and the presence of any one of these symptoms lasting at least 48 hours (except for fever and/or respiratory symptoms) will result in the site arranging an Illness Visit to collect an NP swab sample within 72 hours:

- Fever (temperature ≥ 38ºC) or chills (of any duration, including ≤ 48 hours)
- Shortness of breath or difficulty breathing (of any duration, including ≤ 48 hours)
- Cough (of any duration, including ≤ 48 hours)
- Fatigue
- Muscle or body aches
- Headache
- New loss of taste or smell
- Sore throat
- Congestion or runny nose

- Nausea or vomiting
- Diarrhea

It is important to note that some of the symptoms of COVID-19 overlap with solicited systemic ARs that are expected after vaccination with mRNA-1273 (eg, myalgia, headache, fever, and chills). During the first 7 days after vaccination, when these solicited ARs are common, investigators should use their clinical judgement to decide if an NP swab sample should be collected (Part A). The collection of an NP swab sample prior to the Day 1 and Day 29 vaccination can help ensure that cases of COVID-19 are not overlooked. Any study participant reporting respiratory symptoms during the 7-day period after vaccination should be evaluated for COVID-19.

Figure 3: Surveillance for COVID-19 Symptoms and the Corresponding Clinical Data Pathways

Symptoms that meet the criteria for suspicion of COVID-19 infection

- Fever (temperature ≥ 38°C) or chills
- Cough
- Shortness of breath or difficulty breathing
- Fatigue
- Muscle or body aches
- Headache
- New loss of taste or smell
- Sore throat
- Congestion or runny nose
- Nausea or vomiting
- Diarrhea

Elicited through interaction with the (in person or phone) and/or clinical evaluation by investigator

- Clinical signs indicative of severe systemic illness, Respiratory Rates ≥ 30 per minute, Heart Rate ≥ 125 beats per minute, SpO2 ≤ 93% on room air at sea level or PaO2/FIO2 < 300 mm Hg, OR
- Respiratory failure or Acute Respiratory Distress Syndrome (ARDS), (defined as needing high-flow oxygen, non-invasive or mechanical ventilation, or ECMO), evidence of shock (systolic blood pressure < 90 mmHg, diastolic BP < 60 mmHg or requiring vasopressors), OR
- Significant acute renal, hepatic or neurologic dysfunction, OR
- Admission to an intensive care unit or death.

From Medically-Attended HCP Visit (site or external)

- Positive virologic result by RT-PCR for SARS-CoV-2 infection

PCR results on NP Swab, Nasal Swab, or Saliva Sample

During the course of the study, participants with symptoms of COVID-19 will be asked to return within 72 hours or as soon as possible to the study site or medically qualified staff from the study site will conduct a home visit as soon as possible to collect an NP swab sample (for RT-PCR), collect a blood sample for immunologic analysis of SARS-CoV-2 infection, and to evaluate for COVID-19. Both study site visits and home visits are referred to as Illness Visits. An additional NP swab sample will be collected and tested for the presence of other respiratory pathogens.

In addition, the study site may collect an additional (non-study/local) respiratory sample for SARS-CoV-2 testing to be able to render appropriate medical care for the study participant as determined by local standards of care. If neither a study site visit or home visit is possible,

participants will be sent a saliva kit via courier or other Sponsor-approved method. The study site will arrange to retrieve a saliva sample by local courier or other Sponsor-approved method, and the sample will be tested by RT-PCR for SARS-CoV-2. If participants are confirmed to have SARS-CoV-2 infection, the investigator will notify the participant, and the participant's primary care physician, of the diagnosis. If the study participant does not have a primary care physician, the investigator will assist them to obtain one. The participant will also be instructed on infection prevention measures consistent with local public health guidance.

If scheduled, a study site Illness Visit includes assessments such as medical history, physical examination, blood sampling for clinical laboratory testing, and 2 NP swabs, 1 for viral polymerase chain reaction (PCR) SARS-CoV-2 testing and 1 for multiplex PCR testing for respiratory viruses, to evaluate the severity of the clinical case. Radiologic imaging studies may be conducted. Blood samples will be collected for potential future immunologic assessment of SARS-CoV-2 infection. Every effort to complete an Illness Visit that includes both the NP swabs and blood draw should be made by the site, even in the presence of a local positive or negative Clinical Laboratory Improvement Amendments (CLIA) certified or CLIA-certified waiver laboratory result for the participant.

Cases are defined as participants meeting clinical criteria based both on symptoms for COVID-19 and on RT-PCR detection of SARS-CoV-2 from samples collected within 72 hours of the study participant reporting symptoms meeting the definition of COVID-19. Participants who are hospitalized for COVID-19 without the opportunity for a clinic or home visit will also be considered cases, assuming that the symptomatology criteria for COVID-19 are met and a respiratory sample is positive for SARS-CoV-2 by PCR at a CLIA-certified or CLIA-certified waiver laboratory. Investigators are encouraged to try to obtain a respiratory sample during the course of hospitalization for submission to the study central laboratory, if feasible. The investigator should determine if the criteria for severe COVID-19 has been met.

Evidence of severe COVID-19 is defined as in Section 8.1.1.

All clinical findings will be recorded in the eCRF. All confirmed cases of COVID-19 will be captured as MAAEs, along with relevant concomitant medications and details about severity, seriousness, and outcome.

8.1.3. Convalescent Period Starting With the Illness Visit

All study participants who experience COVID-19 symptoms and subsequently present for an Illness Visit (in-clinic or at home) will be given an instruction card listing symptoms and severity grading system, a thermometer, an oxygen saturation monitor, and saliva collection tubes. Participants will be trained on the use of the oxygen saturation monitor and how to take saliva

specimens. The list of symptoms is presented in Section 8.1.2 and the severity scoring system is presented in Table 3.

Table 3: Grading of COVID-19 Symptoms

Grading	All Symptoms	For Nausea/Vomiting ONLY	For Sense of Smell/Taste ONLY
None	No symptom		
Mild	I had the symptom, but I could still do my normal activities.	I was able to eat and drink normally.	I had the symptom, but I retained some taste/smell.
Moderate	The symptom really bothered me. It was hard to do my normal activities.	It bothered me enough that I did not eat or drink normally.	My taste/smell was significantly affected.
Severe	The symptom was very bad. I was not able to do activities that I usually do.	I could not eat or drink.	I have no taste or smell.

Abbreviation: COVID-19 = coronavirus disease 2019.

The initial Illness Visit is considered Day 1 for the Convalescent Period. Starting on Day 2 of the Convalescent Period, the investigator (or medically qualified staff appropriately delegated by the investigator) will contact participants daily with telemedicine visits through Day 14 (from the Illness Visit or initial COVID-19 contact) or until symptoms have resolved, whichever is later, and which may include symptoms persisting longer than the 28-day Convalescent Period (with the exception of mild loss of sense of taste/smell).

If a participant is completely asymptomatic for 72 hours prior to Day 14, including normal oxygen saturation and temperature, then telemedicine calls can be reduced to weekly; however, the participant should still report the daily symptoms at that contact. The participant should also report their oxygen saturation and temperature measured on the day of the contact, but these need not be recorded daily during this reduced frequency monitoring period. If a participant reports a change or recurrence of symptoms, daily telemedicine visits should be resumed to the daily schedule. For these participants, a final telemedicine visit will occur on Day 14 to determine before symptom monitoring can be discontinued.

If the symptoms persist after Day 60, then telemedicine calls can be reduced to weekly; however, the participant should still report the daily sypmptoms at that contact.

Telemedicine visits may be conducted by videoconference or by audio only (telephone). During the telemedicine visit (preferably done in the evening), the participant will be asked to verbally report the severity of each symptom and their highest body temperature and lowest oxygen saturation for that day, and the investigator will determine if medical attention is required due to

worsening of COVID-19 symptoms. The presence and severity of each symptom reported by the participant will be noted in the appropriate source document (Table 19).

During the telemedicine visits, participants will be reminded both to collect their own saliva sample on 3, 5, 7, 9, 14, and 21 days after the initial Illness Visit and to return the sample to the study site. Immediately upon receipt of a saliva sample, the study site will send it for testing to the study central virology laboratory.

During the telemedicine visits, if the participant has a positive result for SARS-CoV-2 from the Day 1 Illness Visit, the participant will continue the Convalescent Period. If the participant has a negative result for SARS-CoV-2 from the Day 1 Illness Visit, the participant will exit the Convalescent Period, including discontinuation of daily telemedicine visits and collection of saliva samples, and will return to their respective study schedule (Table 16, Table 17, Table 18, or Table 23). Participants who are pending a central laboratory PCR may exit from the Convalescent Period based on a negative PCR result from a CLIA-certified or CLIA-certified waiver local laboratory, at the investigator’s discretion. However, if the central laboratory PCR results are positive after exit from the Convalescent Period, the Convalescent Period must resume.

All participants confirmed to be COVID-19 cases will be scheduled for a Convalescent Visit (study site or home visit) 28 days after the initial Illness Visit. At this visit, a saliva sample will be collected and a blood sample will be drawn for immunologic assessment of SARS-CoV-2 infection (Table 19).

If the participant is hospitalized, medically qualified site personnel will try to obtain medical records and SARS-CoV-2 diagnostic results and document if the criteria for COVID-19 or severe COVID-19 have been met. If the participant is later discharged from the hospital during the 28-day period following diagnosis of COVID-19, the study site personnel will arrange for a resumption of a schedule for telemedicine visits and sampling for the remainder of the Convalescent Period, followed by a return to their respective study schedule (Table 16, Table 17, Table 18, or Table 23).

8.1.4. Ancillary Supplies for Participant Use

Study sites will distribute Sponsor-provided oral thermometers and rulers for use by participants in assessing body temperature and injection site reactions for recording solicited ARs in eDiaries (Section 8.2.2). Based on availability, smartphone devices may be provided to those participants who do not have their own device to use for eDiary activities.

Participants will also receive the following Sponsor-provided supplies at Illness Visits where COVID-19 is suspected:

- An instruction card listing symptoms and severity grading system
- A pulse oximeter for measuring oxygen saturation

- Saliva collection tubes and instructions/means for returning saliva samples collected at home to the study site
- Additional oral thermometer, if required.

8.1.5. Immunogenicity Assessments

Blood samples for immunogenicity assessments will be collected at the timepoints indicated in the SoEs (Section 11.1) and in Table 4. Blood samples for immunogenicity assessment will be collected before administration of IP. The following analytes will be measured:

- Serum bAb levels against SARS-CoV-2 as measured by ligand-binding assay specific to the SARS-CoV-2 S protein
- Serum bAb levels against SARS-CoV-2 as measured by ligand-binding assay specific to the SARS-CoV-2 nucleocapsid protein
- Serum nAb titer against SARS-CoV-2 as measured by pseudovirus and/or live virus neutralization assays

Serum will be tested using the ligand-binding assay specific to the SARS-CoV-2 nucleocapsid to determine the immunologic status of study participants at baseline and assess for seroconversion due to infection during the course of the study. Serum from a subset of participants will be tested in the other assays. The selection of the subset and timepoints to be tested will be described in the statistical analysis plan (SAP). Sample aliquots will be designed to ensure that backup samples are available and that adequate vial volumes may allow for further testing. The actual time and date of each sample collected will be recorded in the eCRF, and unique sample identification will be used to maintain the blind at the laboratory at all times and to allow for automated sample tracking and storage. Handling and preparation of the samples for analysis, as well as shipping and storage requirements, will be provided in a separate study manual.

The ligand-binding assay and measurement of nAb titers will be performed in laboratories designated by the Sponsor.

8.2. Safety Assessments

Safety assessments will include monitoring and recording of the following for each participant:

- Solicited local and systemic ARs (Section 8.3.5) that occur during the 7 days following each injection (ie, the day of dosing and 6 subsequent days). Solicited ARs will be recorded daily using eDiaries (Section 8.2.2) (Part A).
- Unsolicited AEs observed or reported during the 28 days following each injection (ie, the day of injection and 27 subsequent days) (Part A [all participants] and Part C [only those

who receive the BD]). Unsolicited AEs are AEs that are not included in the protocol-defined solicited ARs (Section 8.3.5).

- AEs leading to discontinuation from dosing and/or study participation from Day 1 through Day 759 or withdrawal from the study.
- MAAEs from Day 1 through Day 759 or withdrawal from the study.
- SAEs from Day 1 through Day 759 or withdrawal from the study.
- AEs of special interest (AESIs; for participants who receive the BD in Part C) from BD-Day 1 through Day 759 or withdrawal from the study.
- Abnormal vital sign measurements.
- Physical examination findings.
- Pregnancy and accompanying outcomes.
- Concomitant medications and non-study vaccinations.

8.2.1. Safety Phone Calls

A safety phone call is a telephone call made to the participant by medically qualified study staff. Medically qualified staff are those appropriately delegated individuals who are permitted to elicit verbal medical history from participants based on local regulations and local licensing requirements.

This call will follow a script, which will facilitate the collection of relevant safety information. The participant will be interviewed according to the script about occurrence of unsolicited AEs, MAAEs, SAEs, or AEs leading to study withdrawal and concomitant medications associated with those events, receipt of any non-study vaccinations, and pregnancy. Occurrence of AEs will only be collected by safety phone call during the Vaccination Phase (Table 16, Table 21, and Table 23).

The timing of the safety phone calls and the relevant safety information collected is provided in the SoEs (Section 11.1).

All safety information described by the participant must be documented in source documents and not documented on the script used for the safety telephone contact. All AEs, MAAEs, SAEs, and AEs leading to study withdrawal must be recorded in the eCRF as specified in Section 8.3.7.

8.2.2. Use of Electronic Diaries

In Part A, the Blinded Phase of the study, at the time of consent, the participants must confirm they will be willing to complete an eDiary (for 7-day reactogenicity and to surveil weekly for COVID-19 symptoms) using either an application downloaded to their smartphone or using a

device that is provided at the time of enrollment. This study will utilize the Medidata Patient Cloud Application as the eDiary for both collection of 7-day reactogenicity and weekly eDiary prompts to elicit an unscheduled Illness Visit if the participant is experiencing COVID-19 symptoms. This application allows for real-time data collection on a 21 CFR Part 11 compliant system directly from participants.

In Part A, the Blinded Phase of the study, before enrollment on Day 1, the participant will be instructed to download the eDiary application on their personal smartphone or will be provided a Sponsor-provisioned device to record solicited ARs (Section 8.3.5) and also to be utilized for eDiary prompts through the COVID-19 surveillance period.

In Part A, the Blinded Phase of the study, at each dosing visit, participants will record data into the eDiary starting approximately 30 minutes after dosing under supervision of the study site staff to ensure successful entry of assessments. The 30-minute assessment is an opportunity for site staff to train the participant. The site staff will perform any retraining as necessary. Study participants will continue to record data in an eDiary after they leave the study site, preferably in the evening and at the same time each day, on the day of dosing and for 6 days following dosing.

In Part A, the Blinded Phase of the study, the following local ARs will be solicited by the eDiary: pain at injection site, erythema (redness) at injection site, swelling/induration (hardness) at injection site, and localized axillary swelling or tenderness ipsilateral to the injection arm. The following systemic ARs will be solicited by the eDiary: headache, fatigue, myalgia (muscle aches all over the body), arthralgia (aching in several joints), nausea/vomiting, body temperature (potentially fever), and chills.

In Part A, the Blinded Phase of the study, solicited local and systemic reactogenicity ARs, as defined in Section 8.3.5, will be collected on the day of each IP injection and during the 7 days after IP injection (ie, the day of dosing and 6 subsequent days). Any solicited AR that is ongoing beyond Day 7 will be reported in an eDiary until resolution. Adverse reactions recorded in diaries beyond Day 7 should be reviewed by study site staff either during the next scheduled phone call or at the next study site visit (Table 16).

If eDiary prompts result in identification of relevant safety events according to the study period or symptoms of COVID-19, a follow-up safety call will be triggered.

In Part A, the Blinded Phase of the study, at each dosing visit, participants will be instructed or reminded on thermometer usage to measure body temperature, ruler usage to measure injection site erythema and swelling/induration (hardness), and self-assessment for localized axillary swelling or tenderness on the same side as the injection arm.

In Part A, the Blinded Phase of the study, daily oral body temperature measurement should be performed at approximately the same time each day using the thermometer provided by the study site.

Surveillance for signs and symptoms of COVID-19 will start at Part A Day 1 and continue weekly through the entire Surveillance Phase of the study (Table 17 and Table 18). After participants complete Part A Vaccination Phase of the Study (Table 16 and Table 21), the weekly eDiary Safety Follow-up prompts will be triggered on Day 61 (Table 17). The weekly eDiary prompts will utilize the same Medidata Patient Cloud Application. Each week (ie, every 7 days) the participant will receive a prompt on their smartphone device to regularly surveil for signs and symptoms of COVID-19 and any other changes in health status. The participant will be trained on how to complete the weekly eDiary prompts at the Part A Day 57 clinic visit and also reminded to call the site immediately if they experience any COVID-19 symptoms. The weekly eDiary prompt will inquire about the following:

- Changes in health since the last time completing the weekly eDiary prompt or the last contact with the study staff
- Any known exposure to someone with SARS-CoV-2 infection or COVID-19 since the last time completing the weekly eDiary prompt or the last contact with the study staff
- Capture of any COVID-19 symptoms currently being experienced or experienced since the last time completing the weekly check-in as defined in Section 8.1.2
- Any contact with a healthcare provider that may indicate an MAAE.

A positive response by the participant to these prompts will result in a notification to both the participant and study staff to arrange a call. A follow-up safety call will be performed to the participant to determine if an unscheduled Illness Visit for the participant should be arranged as defined in Section 8.1.2. The results of the safety call should be recorded in the appropriate source documentation.

If a participant does not respond to the weekly eDiary within a 2-day window around the scheduled timepoint, study staff will follow up directly with the participant via phone call or text to confirm their health status and to remind the participant of the importance of maintaining weekly contact via the eDiary prompt.

In addition, there will be an eDiary prompt to solicit the collection of information regarding participant's history of facial injections or dermal fillers, for cosmetic or medical indications such as migraine headaches.

8.2.3. Demographics/Medical History

Demographic information relating to the participant's sex, age, and race will be recorded at Screening on the appropriate eCRF page.

Additionally, information regarding participant occupational circumstances (eg, essential worker status) will be collected at Screening.

Medical history of each participant, including risk factors for severe COVID-19 as defined in Section 6.2.1.1, will be collected and recorded on the Medical History eCRF page. Significant findings that were present prior to the signature of the informed consent will also be included in the Medical History eCRF page.

Study participants will also be asked to report history of receipt of seasonal influenza vaccine during the current influenza season (typically October through April in the Northern Hemisphere) as a concomitant medication.

8.2.4. Physical Examination

A full physical examination, including vital signs, height, and weight, will be performed at Screening and on Day 1, and symptom-directed physical examinations at other scheduled timepoints as indicated in the SoEs (Section 11.1). The full examination will include assessment of skin, head, ears, eyes, nose, throat, neck, thyroid, lungs, heart, cardiovascular, abdomen, lymph nodes, and musculoskeletal system/extremities. Symptom-directed physical examinations may be performed at other timepoints at the discretion of the investigator.

On each dosing day before injection, the arm receiving the injection should be examined and the associated lymph nodes should be evaluated.

Body mass index will be calculated at the Screening Visit (Day 0).

Any clinically significant finding identified during a study visit after the first dose should be reported as an MAAE.

Significant new findings that begin or worsen after informed consent must be recorded on the AE eCRF page.

8.2.5. Vital Sign Measurements

Vital signs will be measured at the timepoints indicated in the SoEs (Section 11.1). The participant will be seated for at least 5 minutes before all measurements are taken. On Day 1 and Day 29, vital sign measurements will be collected once before IP injection and at least 30 minutes after IP injection (before participants are discharged from the study site). When applicable, vital sign measurements should be performed before blood collection.

Febrile participants at Day 1 and Day 29 visits (fever is defined as a body temperature ≥ 38.0°C/100.4°F) may be rescheduled within the relevant window periods. Criteria for delay of study treatment are provided in Section 7.1. Afebrile participants with minor illnesses may be injected at the discretion of the investigator.

If any of the vital sign measurements meet the toxicity grading criteria for clinical abnormalities of Grade 3 or greater, the abnormal value and grade will be documented on the AE page of the eCRF (unless there is another known cause of the abnormality that would result in an AE classification). The investigator will continue to monitor the participant with additional assessments until the vital sign value has reached the reference range, returns to the vital sign value at baseline, is considered stable, or until the investigator determines that follow-up is no longer medically necessary.

8.2.6. Blood Sampling

The maximum planned volumes of blood sampled for immunogenicity analyses per participant are approximately 50 mL for 1 day, 100 mL for 28 days (Table 16), and 300 to 420 mL (dependent on participation in Part B and Part C) for the total study volume (Table 4).

Table 4: Maximum Planned Immunogenicity Blood Sampling Volumes per Participant by Visit

**Study Visit Day**	**Table 16 D1**	**Table 16 D29**	**Table 16 D57**	**Table 17 D209**	**Table 17 D394**	**Table 18 D759**	**Total**
Immunogenicity blood samples [a]	50 mL	50 mL	50 mL	50 mL	50 mL	50 mL	300 mL
Study Visit Day	**Part B Day 1[b]**	**Part B Day 29[b]**	**Part B Day 57[b]**	NA	NA	NA	NA
Immunogenicity blood samples [a]	20 mL	20 mL	20 mL	NA	NA	NA	60 mL[c]
Study Visit Day	**Part C Day 1[d]**	**Part C Day 4[d]**	**Part C Day 29[d]**	**Part C Day 181[d]**	NA	NA	NA
Immunogenicity blood samples [a]	24 mL	8 mL	24 mL	24 mL	NA	NA	80 mL

Abbreviations: COVID-19 = coronavirus disease 2019; D = Day; NA = not applicable; RT-PCR = reverse transcriptase polymerase chain reaction; OL = open-label; SoE = schedule of events.

[a] Additional blood samples (16 mL) will be taken at Illness Day 1 and Convalescent Visit Day 28 for those participants who report symptoms of COVID-19 and who have RT-PCR confirmed symptomatic COVID-19, respectively.

[b] If a visit from SoE Table 16 and Table 17 and a Part B visit overlap, only the Part B sample will be taken (using the Part B kit). The exception would be if an Illness Day 1 or Convalescent Visit Day 28 overlapped with a Part B visit. In this case the Illness and Convalescent Visit Day 28 samples would be taken and the corresponding kits used.

[c] For participants who received placebo during the Part A Blinded Phase and receive mRNA-1273 during Part B. Note that immunogenicity blood samples are collected only on OL-D1 and OL-D57 (Table 21) or OL-D1 and OL-D29 (Table 22) in Part B.

[d] If a visit from SoE Table 17 and Table 18 and a Part C visit overlap, only the Part C sample will be taken (using the Part C kit). Exceptions would be if an Illness Day 1 or Convalescent Visit Day 28 overlapped with a Part C visit, in this case the Illness and Convalescent Visit Day 28 samples would be taken and corresponding kits used, or in the case of a Part C Day 4 visit, both sets of samples would need to be taken using both kits.

8.3. Safety Definitions and Procedures

8.3.1. Adverse Event

An AE is defined as any untoward medical occurrence associated with the use of a drug in humans, whether or not considered drug related.

A treatment-emergent AE (TEAE) is defined as any event not present before exposure to IP or any event already present that worsens in intensity or frequency after exposure.

Events Meeting the Adverse Event Definition

- Exacerbation of a chronic or intermittent pre-existing condition including either an increase in frequency and/or intensity of the condition.
- New conditions detected or diagnosed after the first dose of IP even though they may have been present before the start of the study.

Events NOT Meeting the Adverse Event Definition

- Medical or surgical procedure (eg, endoscopy, appendectomy): the condition that leads to the procedure should be the AE.
- Situations in which an untoward medical occurrence did not occur (social and/or convenience admission to a hospital).

An AR is any AE for which there is a reasonable possibility that the IP caused the AE (Section 8.3.5). For the purposes of investigational new drug safety reporting, "reasonable possibility" means that there is evidence to suggest a causal relationship between the IP and the AE.

An unsolicited AE is any AE reported by the participant that is not specified as a solicited AR in the protocol; or is specified as a solicited AR in the protocol, but starts outside the protocol-defined period for reporting solicited ARs (ie, for the 7 days after each dose of IP, Part A, Blinded Phase only).

8.3.2. Medically Attended Adverse Events

An MAAE is an AE that leads to an unscheduled visit (including a telemedicine visit) to a healthcare practitioner (HCP). This would include visits to a study site for unscheduled

assessments (eg, rash assessment, abnormal laboratory follow-up, COVID-19 [Section 8.1.1]) and visits to HCPs external to the study site (eg, urgent care, primary care physician). Investigators will review unsolicited AEs for the occurrence of any MAAEs. All MAAEs must be fully reported on the MAAE page of the eCRF.

8.3.2.1. Anaphylaxis

All suspected cases of anaphylaxis should be recorded as MAAEs and reported as an SAE, based on criteria for a medically important event, unless the event meets other serious criteria. As an SAE, the event should be reported to the Sponsor or designee immediately and in all circumstances within 24 hours as per Section 8.3.12 (Reporting SAEs). The investigator will submit any updated anaphylaxis case data to the Sponsor within 24 hours of it being available. For reporting purposes, a participant who displays signs/symptoms consistent with anaphylaxis as shown below should be reported as a potential case of anaphylaxis. This is provided as general guidance for investigators and is based on the Brighton Collaboration case definition (Rüggeberg et al 2007).

Anaphylaxis is an acute hypersensitivity reaction with multi-organ system involvement that can present as, or rapidly progress to, a severe life-threatening reaction. It may occur following exposure to allergens from a variety of sources. Anaphylaxis is a clinical syndrome characterized by:

- Sudden onset AND
- Rapid progression of signs and symptoms AND
- Involving 2 or more organ systems, as follows:
    - **Skin/mucosal**: urticaria (hives), generalized erythema, angioedema, generalized pruritus with skin rash, generalized prickle sensation, red and itchy eyes
    - **Cardiovascular**: measured hypotension, clinical diagnosis of uncompensated shock, loss of consciousness or decreased level of consciousness, evidence of reduced peripheral circulation
    - **Respiratory**: bilateral wheeze (bronchospasm), difficulty breathing, stridor, upper airway swelling (lip, tongue, throat, uvula, or larynx), respiratory distress, persistent dry cough, hoarse voice, sensation of throat closure, sneezing, rhinorrhea
    - **Gastrointestinal**: diarrhea, abdominal pain, nausea, vomiting

8.3.3. Adverse Events of Special Interest

An AESI is a serious or nonserious AE of scientific and medical concern specific to the Sponsor's product or program, for which ongoing monitoring and immediate notification by the investigator to the Sponsor is required and documentation is in the form of a case narrative. Such events may

require further investigation to characterize and understand them. Refer to Section 11.5 for a list of AESIs pertinent to this study. All AESIs will be collected for participants receiving the BD in Part C through the remaining study period and must be reported to the Sponsor or designee immediately and in all circumstances within 24 hours of becoming aware of the event via the electronic data capture system. If a site receives a report of a new AESI from a study participant or receives updated data on a previously reported AESI, and the eCRF has been taken offline, then the site can report this information on a paper AESI form using the SAE Mailbox, the SAE Hotline, or the SAE Fax line (Section 8.3.11).

Acute Myocarditis and/or Pericarditis

These definitions are intended to serve as a guide to help in the reporting of suspected cases of myocarditis and/or pericarditis; however, the diagnosis of suspected cases is left to the investigator’s clinical judgement.

All suspected cases of probable and confirmed myocarditis, pericarditis, or myopericarditis should be recorded as an AESI, and reported as an SAE, if the event meets seriousness criteria. As an SAE, the event should be reported to the Sponsor or designee immediately and in all circumstances within 24 hours as per Section 8.3.11. The investigator will submit any updated myocarditis, pericarditis, or myopericarditis case data to the Sponsor within 24 hours of it being available. For reporting purposes, a participant who displays signs/symptoms consistent with the CDC case definitions as described below (Gargano et al 2021), should be reported as a potential case of confirmed or probable myocarditis, pericarditis, or myopericarditis.

Acute Myocarditis Case Definition

Presence of ≥ 1 new or worsening of the following clinical symptoms (persons who lack the listed symptoms but who meet other criteria may be classified as subclinical myocarditis [probable or confirmed]):

- Chest pain/pressure/discomfort
- Dyspnea/shortness of breath/pain with breathing
- Palpitations
- Syncope

AND

For PROBABLE CASE:

Presence of ≥ 1 new finding of the following:

- Troponin level above upper limit of normal (any type of troponin)

- Abnormal electrocardiogram (ECG or EKG) or rhythm monitoring findings consistent with myocarditis
  - To meet the ECG or rhythm monitoring criterion, a probable case must include at least 1 of the following:
    - ST segment or T-wave abnormalities
    - Paroxysmal or sustained atrial, supraventricular, or ventricular arrhythmias
    - AV nodal conduction delays or intraventricular conduction defects
- Abnormal cardiac function or wall motion abnormalities on echocardiogram
- Cardiac magnetic resonance imaging (cMRI) finding consistent with myocarditis (Ferreira et al 2018)

AND

- No other identifiable cause of the symptoms and findings

For CONFIRMED CASE:

- Histopathologic confirmation of myocarditis (using Dallas criteria [Aretz et al 1987])

OR

- cMRI findings consistent with myocarditis in the presence of troponin level above upper limit of normal (any type of troponin)

AND

- No other identifiable cause of the symptoms and findings

Acute Pericarditis Case Definition

Presence of ≥ 2 new or worsening of the following clinical features (Adler et al 2015):

- Acute chest pain (Typically described as pain made worse by lying down, deep inspiration, or cough; and relieved by sitting up or leaning forward, although other types of chest pain may occur)
- Pericardial rub on examination
- New ST-elevation or PR-depression on EKG
- New or worsening pericardial effusion on echocardiogram or magnetic resonance imaging

Myopericarditis Case Definition

Participants who meet criteria for both myocarditis and pericarditis may be described under myopericarditis.

8.3.4. Serious Adverse Events

An AE (including an AR) is considered an SAE if, in the view of either the investigator or Sponsor, it results in any of the following outcomes:

- **Death**
  A death that occurs during the study or that comes to the attention of the investigator during the protocol-defined follow-up period must be reported to the Sponsor, whether or not it is considered related to IP.
- **Is life-threatening**
  An AE is considered life-threatening if, in the view of either the investigator or the Sponsor, its occurrence places the participant at immediate risk of death. It does not include an AE that, had it occurred in a more severe form, might have caused death.
- **Inpatient hospitalization or prolongation of existing hospitalization**
  In general, inpatient hospitalization indicates the participant was admitted to the hospital or emergency ward for at least one overnight stay for observation and/or treatment that would not have been appropriate in the physician's office or outpatient setting. The hospital or emergency ward admission should be considered an SAE regardless of whether opinions differ as to the necessity of the admission. Complications that occur during inpatient hospitalization will be recorded as an AE; however, if a complication/AE prolongs hospitalization or otherwise fulfills SAE criteria, the complication/AE will be recorded as a separate SAE.
- **Persistent or significant incapacity or substantial disruption of the ability to conduct normal life functions**
  This definition is not intended to include experiences of relatively minor medical significance such as uncomplicated headache, nausea/vomiting, diarrhea, influenza, and accidental trauma (eg, sprained ankle) which may interfere with or prevent everyday life functions but do not constitute a substantial disruption.
- **Congenital anomaly or birth defect**
- **Medically important event**
  Medical judgment should be exercised in deciding whether SAE reporting is appropriate in other situations, such as important medical events that may not be immediately life-threatening or result in death or hospitalization but may jeopardize the participant or require medical or surgical intervention to prevent one of the other outcomes listed in the

above definition. These events should usually be considered serious. Examples of such medical events include allergic bronchospasm requiring intensive treatment in an emergency room or at home, blood dyscrasias or convulsions that do not result in inpatient hospitalization, or the development of drug dependency or drug abuse.

8.3.5. Solicited Adverse Reactions (Part A)

The term "reactogenicity" refers to the occurrence and intensity of selected signs and symptoms (ARs) occurring after IP injection. The eDiary will solicit daily participant reporting of ARs using a structured checklist (Section 8.2.2). Participants will record such occurrences in an eDiary on the day of IP injection and for the 6 days after the day of dosing.

Severity grading of reactogenicity will occur automatically based on participant entry into the eDiary according to the grading scales presented in Table 5 modified from the Toxicity Grading Scale for Healthy Adult and Adolescent Volunteers Enrolled in Preventative Vaccine Clinical Trials (DHHS 2007).

If a solicited local or systemic AR continues beyond 7 days after dosing, the participant will be prompted daily to capture solicited local or systemic AR in the eDiary until resolution. Adverse reactions recorded in eDiaries beyond Day 7 should be reviewed by the investigator either via phone call or at the following study visit. All solicited ARs (local and systemic) will be considered causally related to dosing.

Table 5: Solicited Adverse Reactions and Grades

Reaction	Grade 0	Grade 1	Grade 2	Grade 3	Grade 4
Injection site pain	None	Does not interfere with activity	Repeated use of over-the-counter pain reliever > 24 hours or interferes with activity	Any use of prescription pain reliever or prevents daily activity	Requires emergency room visit or hospitalization
Injection site erythema (redness)	< 25 mm/ < 2.5 cm	25 – 50 mm/ 2.5 – 5 cm	51 – 100 mm/ 5.1 – 10 cm	> 100 mm/ > 10 cm	Necrosis or exfoliative dermatitis
Injection site swelling/induration (hardness)	< 25 mm/ < 2.5 cm	25 – 50 mm/ 2.5 – 5 cm	51 – 100 mm/ 5.1 – 10 cm	> 100 mm/ > 10 cm	Necrosis
Axillary (underarm) swelling or tenderness ipsilateral to the side of injection	None	No interference with activity	Repeated use of over-the-counter (non-narcotic) pain reliever > 24 hours or some interference with activity	Any use of prescription (narcotic) pain reliever or prevents daily activity	Emergency room visit or hospitalization
Headache	None	No interference with activity	Repeated use of over-the-counter pain reliever > 24 hours or some interference with activity	Significant; any use of prescription pain reliever or prevents daily activity	Requires emergency room visit or hospitalization
Fatigue	None	No interference with activity	Some interference with activity	Significant; prevents daily activity	Requires emergency room visit or hospitalization
Myalgia (muscle aches all over body)	None	No interference with activity	Some interference with activity	Significant; prevents daily activity	Requires emergency room visit or hospitalization
Arthralgia (joint aches in several joints)	None	No interference with activity	Some interference with activity	Significant; prevents daily activity	Requires emergency room visit or hospitalization

**Reaction**	**Grade 0**	**Grade 1**	**Grade 2**	**Grade 3**	**Grade 4**
Nausea/vomiting	None	No interference with activity or 1-2 episodes/ 24 hours	Some interference with activity or > 2 episodes/24 hours	Prevents daily activity, requires outpatient intravenous hydration	Requires emergency room visit or hospitalization for hypotensive shock
Chills	None	No interference with activity	Some interference with activity not requiring medical intervention	Prevents daily activity and requires medical intervention	Requires emergency room visit or hospitalization
Fever (oral)	< 38.0°C<br>< 100.4°F	38.0 – 38.4°C<br>100.4 – 101.1°F	38.5 – 38.9°C<br>101.2 – 102.0°F	39.0 – 40.0°C<br>102.1 – 104.0°F	> 40.0°C<br>> 104.0°F

Any solicited AR that meets any of the following criteria must be entered into the participant's source document and must also be recorded as an AE in the participant's Adverse Event eCRF:

- Solicited local or systemic AR that results in a visit to an HCP (MAAE)
- Solicited local or systemic AR leading to the participant withdrawing from the study or the participant being withdrawn from the study by the investigator (AE leading to withdrawal)
- Solicited local or systemic AR lasting beyond 7 days post injection
- Solicited local or systemic AR that leads to participant withdrawal from IP
- Solicited local or systemic AR that otherwise meets the definition of an SAE

8.3.6. Recording and Follow-up of Pregnancy

Female participants who have a positive pregnancy test at Screening should not be enrolled; participants who have a positive pregnancy test any time during the study should receive no further dosing with IP but should be asked to remain in the study and be followed-up for safety. Pregnancy testing is scheduled to occur at Screening, Blinded and OL Day 1, Blinded and OL Day 29, and Part C BD-Day 1 (Table 16, Table 21, Table 22, and Table 23).

Details of all pregnancies in female participants will be collected after the start of study treatment and until Day 759.

- If a pregnancy is reported, the investigator should inform the Sponsor within 24 hours of learning of the pregnancy and should follow the procedures outlined in this section.
- Abnormal pregnancy outcomes (eg, spontaneous abortion, fetal death, stillbirth, congenital anomalies, ectopic pregnancy) are considered SAEs.

Pregnancies occurring in participants after enrollment must be reported to Sponsor or designee within 24 hours of the site learning of its occurrence. If the participant agrees to submit this information, the pregnancy must be followed to determine the outcome, including spontaneous or voluntary termination, details of the birth, and the presence or absence of any birth defects, congenital abnormalities, or maternal and/or newborn complications. This follow-up should occur even if intended duration of the safety follow-up for the study has ended. Pregnancy report forms will be distributed to the study site to be used for this purpose. The investigator must immediately (within 24 hours of awareness) report to the Sponsor any pregnancy resulting in an abnormal outcome according to the procedures described for SAEs.

8.3.7. Recording and Follow-up of an AE and/or SAE

The investigator is responsible for ensuring that all AEs and SAEs are recorded in the eCRF and reported to the Sponsor.

Solicited ARs will be collected from Day 1 through 7 days after each dose (Part A). Other (unsolicited) AEs will be collected from Day 1 through 28 days after each dose in Part A (all participants) and Part C (only those who receive the BD).

Both MAAEs and SAEs will be collected from Day 1 throughout the entire study duration (Day 759 for all participants), as specified in the SoEs (Table 16, Table 17, Table 18, Table 19, Table 21, Table 22, and Table 23). Any AEs occurring before receipt of IP will be analyzed separately from TEAEs.

At every study site visit or telephone contact, participants will be asked a standard question to elicit any medically related changes, including surveillance for COVID-19 symptoms, in their well-being according to the scripts provided. Participants will also be asked if they have been hospitalized, had any accidents, used any new medications, changed concomitant medication regimens (both prescription and over-the-counter medications), or had any non-study vaccinations.

In addition to participant observations, physical examination findings or other documents relevant to participant safety classified as an AE will be documented on the AE page of the eCRF.

After the initial AE/SAE report, the investigator is required to proactively follow each participant at subsequent visits/contacts. All AEs and SAEs will be treated as medically appropriate and followed until resolution, stabilization, the event is otherwise explained, or the participant is LTFU (as defined in Section 7.4).

8.3.8. Time Period and Frequency for Collecting AE and SAE Information

All confirmed serious COVID-19 cases and SAEs will be recorded and reported to the Sponsor or designee immediately and under no circumstance should this exceed 24 hours, as indicated in Section 8.3.11. The investigator will submit any updated serious COVID-19 cases and SAE data to the Sponsor within 24 hours of it being available. COVID-19 cases are defined in Section 8.1.1.

Investigators are not obligated to actively seek AEs or SAEs after conclusion of the study participation (Day 759). However, if an investigator learns of any SAE, including a death, at any time after a participant has been discharged from the study and considers the event to be reasonably related to the study IP or study participation, the investigator must promptly notify the Sponsor.

8.3.9. Assessment of Intensity

An event is defined as “serious” when it meets at least one of the predefined outcomes as described in the definition of an SAE (Section 8.3.4), NOT when it is rated as severe.

The severity (or intensity) of an AR or AE refers to the extent to which it affects the participant's daily activities. The Toxicity Grading Scale for Healthy Adult and Adolescent Volunteers Enrolled in Preventative Vaccine Clinical Trials (DHHS 2007) will be used to categorize local and systemic reactogenicity events (solicited ARs), clinical laboratory test results, and vital sign measurements observed during this study. Specific criteria for local and systemic reactogenicity events are presented in Section 8.3.55.

The determination of severity for all unsolicited AEs should be made by the investigator based upon medical judgment and the definitions of severity as follows:

- Mild: These events do not interfere with the participant's daily activities.
- Moderate: These events cause some interference with the participant's daily activities and require limited or no medical intervention.
- Severe: These events prevent the participant's daily activity and require intensive therapeutic intervention.

Study staff should elicit from the participant the impact of AEs on the participant's activities of daily living to assess severity and document appropriately in the participant's source documentation. Changes in the severity of an AE should be documented in the participant's source documentation to allow an assessment of the duration of the event at each level of intensity to be performed. An AE characterized as intermittent requires documentation of onset and duration of each episode. An AE that fluctuates in severity during the course of the event is reported once in the eCRF at the highest severity observed.

8.3.10. Assessment of Causality

The investigator's assessment of an AE's relationship to IP is part of the documentation process but is not a factor in determining what is or is not reported in the study.

The investigator will assess causality (ie, whether there is a reasonable possibility that the IP caused the event) for all AEs and SAEs. The relationship will be characterized using the following classification:

Not related: There is not a reasonable possibility of a relationship to the IP. Participant did not receive the IP OR temporal sequence of the AE onset relative to administration of the IP is not reasonable OR the AE is more likely explained by another cause than the IP.

Related: There is a reasonable possibility of a relationship to the IP. There is evidence of exposure to the IP. The temporal sequence of the AE onset relative to the administration of the IP is reasonable. The AE is more likely explained by the IP than by another cause.

8.3.11. Reporting Adverse Events

The investigator is responsible for reporting all AEs that are observed or reported during the study, regardless of their relationship to IP or their clinical significance. If there is any doubt as to whether a clinical observation is an AE, the event should be reported.

All unsolicited AEs reported or observed during the study will be recorded on the AE page of the eCRF. Information to be collected includes, type of event, time of onset, investigator-specified assessment of severity (impact on activities of daily living) and relationship to IP, time of resolution of the event, seriousness, as well as any required treatment or evaluations, and outcome. The unsolicited AEs resulting from concurrent illnesses, reactions to concurrent illnesses, reactions to concurrent medications, or progression of disease states must also be reported. All AEs will be followed until they are resolved or stable or judged by the investigator to be not clinically significant. The Medical Dictionary for Regulatory Activities (MedDRA) will be used to code all unsolicited AEs.

Any medical condition that is present at the time that the participant is screened but does not deteriorate should not be reported as an unsolicited AE. However, if it deteriorates at any time during the study, it should be recorded as an unsolicited AE.

Any AE or COVID-19 case considered serious by the investigator or that meets SAE criteria (Section 8.3.4) must be reported to the Sponsor immediately (within 24 hours of becoming aware of the SAE or COVID-19 case). The investigator will assess whether there is a reasonable possibility that the IP caused the SAE. The Sponsor will be responsible for notifying the relevant regulatory authorities of any SAE as outlined in the 21 US CFR Parts 312 and 320. The investigator is responsible for notifying the institutional review board (IRB) directly.

If the eCRF is unavailable at the time of the SAE, the following contact information is to be used for SAE reporting:

- SAE Mailbox: Safety_Moderna@iqvia.com
- SAE Hotline (USA and Canada): +1-866-599-1341
- SAE Fax line (USA and Canada): +1-866-599-1342

8.3.12. Regulatory Reporting Requirements for SAEs

Prompt notification by the investigator to the Sponsor of a SAE is essential so that legal obligations and ethical responsibilities towards the safety of participants and the safety of a study intervention under clinical investigation are met.

The Sponsor has a legal responsibility to notify both the local regulatory authority and other regulatory agencies about the safety of a study intervention under clinical investigation. The

Sponsor will comply with country-specific regulatory requirements relating to safety reporting to the regulatory authority, IRBs, and investigators.

Investigator safety reports must be prepared for suspected unexpected serious ARs according to local regulatory requirements and Sponsor policy and forwarded to investigators as necessary.

An investigator who receives an investigator safety report describing a SAE or other specific safety information (eg, summary or listing of SAEs) from the Sponsor will review and then file it along with the IB and will notify the IRB/IEC, if appropriate according to local requirements.

8.4. Monitoring Committees

8.4.1. Protocol Safety Review Team

A Protocol Safety Review Team (PSRT) will be formed to review interim and cumulative blinded (unblinded in Part C) safety data on a regular basis with a remit to escalate concerns to the DSMB (DSMB limited to Part A only). The PSRT composition, its remit, and frequency of data review will be further defined in a charter.

8.4.2. Data and Safety Monitoring Board

An independent DSMB will periodically review blinded and unblinded data, including both safety and cases of COVID-19 at scheduled data review meetings and at 2 planned IAs.

- In addition to blinded and unblinded review of safety data, at each data review meeting the DSMB will review the numbers and rate of COVID-19 cases, including rate of severe COVID disease with prespecified thresholds for imbalance in the treatment groups which would trigger halting rules.
- At the IA, the DSMB will review the IA results and make recommendations to an Oversight Group in terms of study results reporting and unblinding based on the boundaries of early efficacy as described in Section 9.6 of the protocol. The Oversight Group will be comprised of a voting member each from the Sponsor, Biomedical Advance Research and Development Authority, and NIAID.
- The DSMB will monitor the study for non-efficacy at the IA. The boundary for non-efficacy is non-binding and will be provided in the DSMB analysis plan.
- The DSMB will also monitor the study for vaccine harm based on severe COVID-19. Continuous harm monitoring will be provided for COVID-19 and severe COVID-19 separately. For harm monitoring, cases will be counted starting after the first dose of study vaccination. Boundaries will be provided based on the exact 1-sided binomial tests conditional on the total number of cases under the assumption of VE=0%. If the prespecified stopping boundary is reached for either COVID-19 or severe COVID-19, then

the unblinded statisticians will immediately inform the DSMB that the harm rules have been met. Details will be provided in the DSMB analysis plan.

- The boundaries are considered guidelines, ie, a recommendation to modify the study would not be based solely on statistical rules, as many other factors (ie, totality of the data from the study including additional efficacy, safety, and immunogenicity endpoints as well as data external to the study) may be part of the decision process. In the case of a recommendation to continue the trial regardless of crossing the boundaries for efficacy or inefficacy, the reason for disregarding the boundary must be documented in the meeting minutes and communicated to the Sponsor.

After each data review meeting or IA, the DSMB will make a recommendation to the Sponsor through an Oversight Group to take one of the following courses of action:

- Stop further enrollment due to meeting criteria for early efficacy or due to a safety concern.
- Pause enrollment and consider a change in study design.
- Continue enrollment and/or study conduct as planned.

The Sponsor may also request that the DSMB conduct ad hoc reviews of safety events from this study or other data, including new nonclinical or clinical information related to mRNA-1273 external to this study. The DSMB will review all available study data to adjudicate such events in accordance with the DSMB charter.

The DSMB composition, its remit, and frequency of data review will be further defined in the DSMB charter and analysis plan.

8.4.3. Adjudication Committee

An Adjudication Committee (AC) will be assembled for the purpose of reviewing potential cases to determine if the criteria for the primary and secondary endpoints have been met. The AC will remain blinded to treatment assignment when adjudicating cases for participants whose treatment group is still blinded. The AC composition, its remit, and frequency of data review will be further defined in a charter.

8.4.4. Independent Cardiac Event Adjudication Committee

An independent Cardiac Event Adjudication Committee (CEAC) that includes pediatric and adult cardiologists will review suspected cases of myocarditis and pericarditis to determine if they meet CDC criteria of “probable” or “confirmed” events, and to assess severity (Gargano et al 2021). Any cases that the CEAC assesses as representing probable or confirmed cases of myocarditis or pericarditis will be referred to the Sponsor, who will then make a final decision on whether to

suspend further enrollment and/or study dosing based on an assessment of the overall potential risk to study participants.

The CEAC will operate under the rules of an approved charter that will be written and reviewed at the organizational meeting of the CEAC. Details regarding the CEAC composition, responsibilities, procedures, and frequency of data review will be defined in its charter.

8.5. Management of Overdose

As the study treatment is to be administered by a healthcare professional, it is unlikely that an overdose will occur. Dose deviations will be tracked as protocol deviations (Section 11.2.8).

8.6. Pharmacokinetics

Pharmacokinetic parameters are not evaluated in this study**.**

8.7. Pharmacodynamics

Pharmacodynamic parameters are not evaluated in this study.

8.8. Exploratory Assessments and Biomarkers

Exploratory assessments may include assessment of biomarkers for safety, reactogenicity, inflammatory and cardiac function. Serologic markers of disease severity, immune response to SARS-CoV-2, RT-PCR of NP swab or saliva samples, and genetic sequences of SARS-CoV-2 strains isolated from participants' samples may also be measured.

8.9. Medical Resource Utilization and Health Economics

Medical resource utilization and health economics parameters are not evaluated in this study.

9. STATISTICAL CONSIDERATIONS

This section summarizes the planned statistical analysis strategy and procedures for the study, focusing on the Blinded Phase of the study (Part A). The details of statistical analysis will be provided in the SAP, which will be finalized before the clinical database lock for the study and treatment unblinding. If, after the study has begun, but prior to any unblinding, changes are made to primary and/or key secondary objectives/hypotheses, or the statistical methods related to those hypotheses, then the protocol will be amended (consistent with ICH Guideline E9). Changes to other secondary or exploratory analyses made after the protocol has been finalized, along with an explanation as to when and why they occurred, will be listed in the SAP or clinical study report (CSR) for the study. Ad hoc exploratory analyses, if any, will be clearly identified in the CSR. Statistical analysis strategy incorporating the Open-Label Observational Phase (Part B), and to assess the BD of mRNA-1273 (Part C) will be provided in the SAP.

9.1. Blinding and Responsibility for Analyses

Blinding during the Part A, the Blinded Phase of the study will be conducted as described in Section 6.2.8. The Sponsor Biostatistics department or designee will generate the randomized allocation schedule(s) for study treatment assignment. Randomization will be implemented via an IRT.

Planned interim and primary analyses are described in Section 9.6. Participant-level unblinding will be restricted to an independent unblinded statistician and, as needed, a statistical programmer performing the IAs, who will have no other responsibilities associated with the study.

In addition to the routine study monitoring outlined in this protocol, an external DSMB will review interim data to safeguard the interests of clinical study participants and to enhancing the integrity of the study. The DSMB will review treatment-level results of the IAs, provided by the independent unblinded statistician. Limited additional Sponsor personnel may be unblinded to the treatment-level results of the IAs, if required, in order to act on the recommendations of the DSMB. The extent to which individuals are unblinded with respect to results of IAs will be documented. Depending on the recommendation of the DSMB, the Sponsor may prepare a regulatory submission after an IA. In this case, pre-identified Sponsor members including the analysis and reporting team will be unblinded to treatment assignments and remain unblinded for the remainder of the study. Participants and investigators will remain blinded.

9.2. Statistical Hypotheses

For the primary efficacy objective, the null hypothesis of this study is that the VE of mRNA-1273 to prevent first occurrence of COVID-19 is ≤ 30% (ie, $H_0^{efficacy}$: VE ≤ 0.3). The study will be considered to meet the primary efficacy objective if the corresponding confidence interval (CI) of VE rules out 30% at either one of the IAs or at the primary analysis.

Vaccine efficacy is defined as the percent reduction in the hazard of the primary endpoint (mRNA-1273 vs. placebo). Equivalently, the null hypothesis is:

$H_0^{efficacy}$: hazard ratio (HR) $\geq$ 0.7 (equivalently, proportional hazards VE $\leq$ 0.3).

A stratified Cox proportional hazard model will be used to assess the magnitude of the treatment group difference (ie, HR) between mRNA-1273 and placebo at a 1-sided 0.025 significance level.

The primary analysis population for efficacy will be the PP Set, defined in Section 9.4. In the primary analysis of efficacy, cases will be counted starting 14 days after the second dose of IP.

Hypothesis testing for immunogenicity is described in Section 9.5.3.2.

9.3. Sample Size Determination

The sample size is driven by the total number of cases to demonstrate VE (mRNA-1273 vs. placebo) to prevent COVID-19 in Part A. Under the assumption of proportional hazards over time and with 1:1 randomization of mRNA-1273 and placebo, a total of 151 COVID-19 cases will provide 90% power to detect a 60% reduction in hazard rate (60% VE), rejecting the null hypothesis H_0: VE $\leq$ 30%, with 2 IAs at 35% and 70% of the target total number of cases using a 1-sided O’Brien-Fleming boundary for efficacy and a log-rank test statistic with a 1-sided false positive error rate of 0.025. The total number of cases pertains to the PP Set accruing at least 14 days after the second dose. There are 2 planned IAs in this study, which will be performed when approximately 35% and 70% of the target total number of cases have been observed. Approximately 30,000 participants will be randomized with the following assumptions:

- The target VE against COVID-19 is 60% (with 95% CI lower bound ruling out 30%, rejecting the null hypothesis H_0: VE $\leq$ 30%)
- A 6-month COVID-19 incidence rate of 0.75% in the placebo arm
- An annual dropout rate of 2% (loss of evaluable participants)
- Two IAs at 35% and 70% of total target cases across the 2 treatment groups with O’Brien-Fleming boundaries for efficacy monitoring
- 3-month uniform accrual
- Approximately 15% of participants will be excluded from the PP population, and participants are at risk for COVID-19 starting 14 days after the second dose

Table 6 provides sample size with 90% power to demonstrate VE on COVID-19.

Table 6: Conditions and Sample Size to Demonstrate Vaccine Efficacy

Target VE	Lower Bound	Randomization Ratio	Total # of Cases	6-Month Incidence Rate		Total Sample Size[a]
--	--	--	--	Placebo	mRNA-1273	--
60%	30%	1:1	151	0.75%	0.30%	30,000

Abbreviations: IP = investigational product; PP = Per Protocol; VE = vaccine efficacy.

[a] Sample size to account for 15% participants to be excluded from the PP Set (eg, seropositive at baseline, have not received planned IP).

The sample size is calculated using R package gsDesign (Anderson 2020).

Under these above assumptions including 6-month incidence rate of 0.75% on placebo, with 30,000 participants, it will take approximately 5, 8, and 10 months from study start (first subject first dose), respectively, to accrue 35% (approximately 53), 70% (approximately 106) and 100% (151) of the target number of cases in the PP Set.

Figure 4 shows the power of the primary efficacy endpoint under true VE at the 2 planned IAs and the primary efficacy analysis assuming a total of 151 events.

Figure 4: Boundary Crossing Probabilities by Effect Size

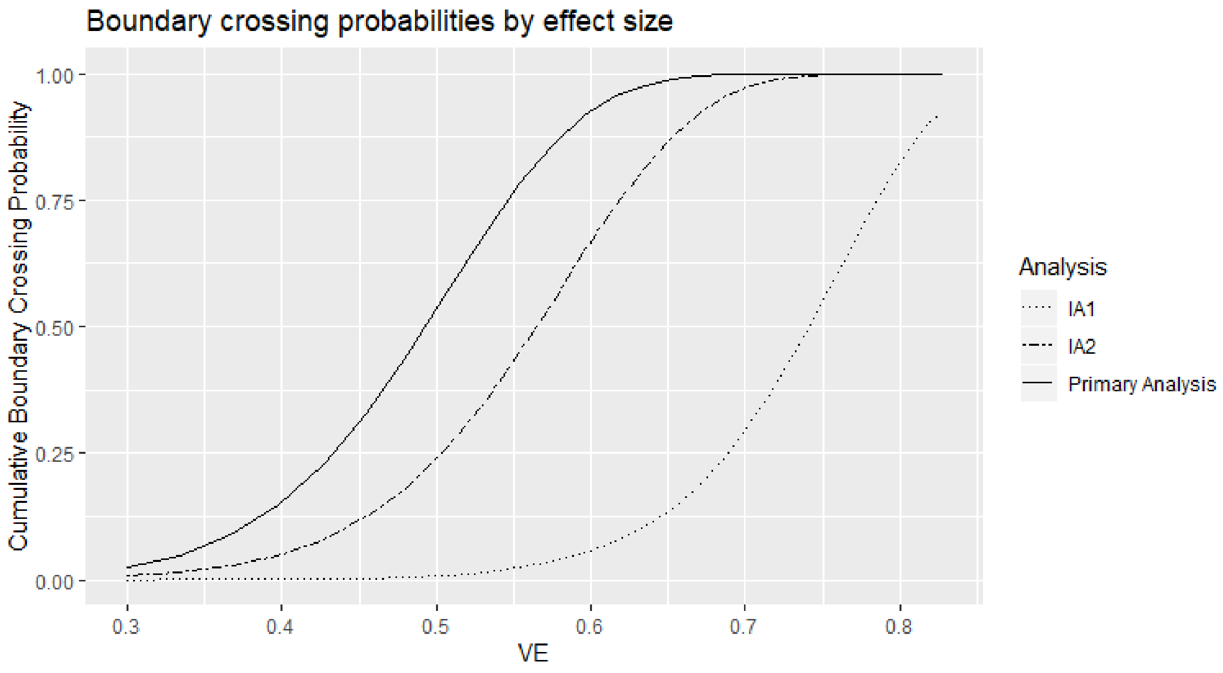

The Sponsor may adjust the size of the study or duration of follow-up based on the blinded review of the total number of cases of COVID-19 accrued during the study, in addition to estimated percentages of study participants with serologic evidence of SARS-CoV-2 infection at baseline.

9.3.1. Power for Selected Secondary Efficacy Endpoints

For the secondary objective on VE against virologically confirmed SARS-CoV-2 infection or COVID-19 regardless of symptomatology or severity (COV-INF), the study will have ≥ 90% power to demonstrate the VE is above 30% (to reject null hypothesis VE ≤ 30%) at 1-sided alpha of 2.5% if the true VE to prevent COV-INF is 60% because every COVID-19 endpoint is necessarily a COV-INF endpoint.

For the secondary objective on VE against severe COVID-19, Table 7 provides power of demonstrating VE based on a total of 30 and 60 events under different scenarios of true VE and VE criteria.

Table 7: Power of Demonstrating Vaccine Efficacy Against Severe COVID-19

True VE	Total No. of Severe COVID-19 Cases	Severe COVID-19 (Secondary Objective)	
		To Reject Null Hypothesis	Power
60%	30	VE ≤ 0%	70.90%
		VE ≤ 10%	60.30%
		VE ≤ 20%	47.50%
70%		VE ≤ 0%	> 90%
		VE ≤ 10%	85.30%
		VE ≤ 20%	76.60%
80%		VE ≤ 0%	> 90%
		VE ≤ 10%	> 90%
		VE ≤ 20%	> 90%
60%	60	VE ≤ 0%	> 90%
		VE ≤ 10%	88.10%
		VE ≤ 20%	76.60%
70%		VE ≤ 0%	> 90%
		VE≤ 10%	> 90%
		VE≤ 20%	> 90%
80%		VE≤ 0%	> 90%
		VE≤ 10%	> 90%
		VE≤ 20%	> 90%

Abbreviations: COVID-19 = coronavirus disease 2019; VE = vaccine efficacy.

9.4. Analysis Populations

Analysis populations for statistical analyses are Randomization Set, Full Analysis Set (FAS), modified Intent-to-Treat (mITT) Set, PP Set, Immunogenicity Subset, Solicited Safety Set, and Safety Set, as shown in Table 8.

Table 8: Populations for Analyses

Population	Description
Randomization Set	All participants who are randomized, regardless of the participants' treatment status in the study.
Full Analysis Set (FAS)	All randomized participants who received at least one dose of IP.<br>Participants will be analyzed according to the group to which they were randomized.
Modified Intent-to-Treat (mITT) Set	All participants in the FAS who had no immunologic or virologic evidence of prior COVID-19 (ie, negative NP swab test at Day 1, and/or bAb against SARS-CoV-2 nucleocapsid below LOD or LLOQ) at Day 1 before the first dose of IP.<br>Participants will be analyzed according to the group to which they were randomized.
Per-protocol (PP) Set	All participants in the mITT Set who received planned doses of IP per schedule and have no major protocol deviations, as determined and documented by Sponsor prior to database lock and unblinding, that impact critical or key study data.<br>Participants will be analyzed according to the group to which they were randomized.
Immunogenicity Subset	All participants in the FAS who were sampled into a subset for characterizing mRNA-1273 immunogenicity and had a valid immunogenicity test result prior to the first dose of IP and at least 1 valid result after the first dose of IP. Participants in the subset who had major protocol deviations that impact critical or key immunogenicity or study data may be excluded from the Immunogenicity Subset. The details of the Immunogenicity Subset will be documented prior to analysis of immunogenicity data.
Solicited Safety Set	The Solicited Safety Set consists of all randomized participants who received at least one dose of IP and contributed any solicited AR data.<br>The Solicited Safety Set will be used for the analyses of solicited ARs and participants will be included in the treatment group corresponding to the IP that they actually received.
Safety Set	All randomized participants who received at least one dose of IP. The Safety Set will be used for all analyses of safety except for the solicited ARs.<br>Participants will be included in the treatment group corresponding to the IP that they actually received.

Abbreviations: AR = adverse reaction; bAb = binding antibody; COVID-19 = coronavirus disease 2019; IP = investigational product; LLOQ = lower limit of quantification; LOD = limit of detection; NP = nasopharyngeal; SARS-CoV-2 = severe acute respiratory syndrome coronavirus 2.

9.5. Statistical Analyses

This section provides a summary of the planned statistical analyses of the primary and secondary endpoints.

The overall Type I error rate for the primary endpoint at the IAs and the primary analysis is strictly controlled at 2.5% (1-sided) based on the Lan-DeMets O'Brien-Fleming approximation spending function (see Section 9.6 for details). The primary efficacy results that will be considered statistically significant after consideration of the strategy for controlling the Type I error as described in Section 9.6. Statistical significance of the primary efficacy endpoint can be achieved at either one of the IAs or at the primary analysis. A sequential/hierarchical testing procedure will be used to control Type 1 error rate over the primary efficacy endpoint and the secondary efficacy endpoints. Secondary efficacy endpoints will only be tested when the primary efficacy endpoint achieves statistical significance. Multiplicity adjustments among the secondary efficacy endpoints may be performed for secondary efficacy endpoints, in that case, will be specified in the SAP.

No formal multiple comparison adjustments will be employed for multiple safety endpoints or multiple efficacy endpoints. Nominal p-values and CIs may be computed for other efficacy analyses without controlling for multiplicity as a measure of VE.

9.5.1. Efficacy Analyses

Efficacy analyses will be performed using the FAS, mITT, and PP populations, and participants will be included in the treatment group to which they were randomized. The primary analysis population will be the PP Set.

Table 9 summarizes the analysis approach for primary and secondary efficacy endpoints for Part A, the randomized, observer-blind, and placebo-controlled phase of the study. Sensitivity analysis methods are described for each endpoint as applicable.

Table 9: Statistical Analysis Methods of Efficacy Endpoints

Endpoint	Statistical Analysis Methods
**Primary endpoint:**<br>Vaccine Efficacy (VE) of mRNA-1273 to prevent COVID-19	• Primary analysis: VE will be estimated with 1 – HR (mRNA-1273 vs. placebo) using a Cox proportional hazard regression model with treatment group as a fixed effect and adjusting for stratification factor based on the PP Set, with cases counted starting 14 days after the second dose of IP.<br>• Analysis using the same model based on the mITT Set.<br>• Sensitivity analysis using the same model based on the PP Set, with cases counted starting either immediately after the second dose of IP or immediately after the first dose of IP.

Endpoint	Statistical Analysis Methods
	• Subgroup analysis of the primary efficacy endpoint will be performed to assess consistency of VE, such as in the age groups ≥ 18 and < 65 years and ≥ 65 years.<br>• Supportive analysis of VE to be estimated with 1 – ratio of incidence rates with 95% CI using the exact method conditional upon the total number of cases.<br>• Supportive analysis of cumulative incidence VE.
**Secondary endpoints:**<br>• Vaccine efficacy of mRNA-1273 to prevent severe COVID-19<br>• Vaccine efficacy of mRNA-1273 to prevent serologically confirmed SARS-CoV-2 infection or COVID-19 regardless of symptomatology or severity<br>• Vaccine efficacy of mRNA-1273 to prevent COVID-19 using a secondary definition of symptoms<br>• Vaccine efficacy of mRNA-1273 to prevent death due to COVID-19<br>• Vaccine efficacy of mRNA-1273 to prevent COVID-19 after the first dose of IP<br>• Vaccine efficacy of mRNA-1273 to prevent asymptomatic SARS-CoV-2 infection	Similar analysis method as for the primary endpoint analysis.<br>For each of the secondary endpoints:<br>• Primary analysis: VE will be estimated with 1 – HR (mRNA-1273 vs. placebo) using a Cox proportional hazard regression model with treatment group as a fixed effect and adjusting for stratification factor based on the PP Set, with cases counted starting 14 days after the second dose of IP.<br>• Analysis using the same model based on the mITT Set.<br>• Sensitivity analyses with cases counted starting immediately after the second dose of IP, 14 days after the first dose of IP, immediately after the first dose of IP, and immediately after randomization.<br>• Vaccine efficacy and 95% CI based on the case incidence will be estimated with 1 – ratio of incidence rates using the exact method conditional upon the total number of cases.
• Vaccine efficacy of mRNA-1273 to prevent COVID-19 in all study participants, regardless of evidence of prior SARS-CoV-2 infection	The FAS population will be used for this secondary objective, using similar analysis methods as for the primary endpoint analysis.<br>• Primary analysis: VE will be estimated with 1 – HR (mRNA-1273 vs. placebo) using a Cox proportional hazard regression model with treatment group as a fixed effect and adjusting for stratification factor based on the FAS, with cases counted starting 14 days after the second dose of IP.<br>• Sensitivity analyses with cases counted starting immediately after the second dose of IP, 14 days after the first dose of IP, immediately after the first dose of IP, and immediately after randomization.

Abbreviations: CI = confidence internval; COVID-19 = coronavirus disease 2019; FAS = Full Analysis Set; HR= hazard ratio; IP = investigational product; mITT = modified Intent-to-Treat; PP = Per Protocol; SARS-CoV-2 = severe acute respiratory syndrome coronavirus 2.

9.5.1.1. Efficacy Analysis on Primary Endpoint

To assess the primary efficacy endpoint of VE of mRNA-1273 in preventing the first occurrence of COVID-19 from 14 days after second dose of IP, Cox proportional hazards regression will be used to estimate proportional hazards VE (PH VE), measured by one minus the HR (mRNA-1273 vs. placebo), with a 2-sided score-based 95% CI and 2-sided p-value for testing H_0: VE ≤ 30%.

Vaccine efficacy is defined as the percent reduction in the hazard of the primary endpoint (mRNA-1273 vs. placebo). The VE will be estimated using one minus the HR (mRNA-1273 vs. placebo) estimand. A stratified Cox proportional hazard model will be used to assess the magnitude of the treatment group difference (ie, HR) between mRNA-1273 and placebo. The HR and its 95% CI from the stratified Cox model with Efron's method of tie handling and with treatment group as covariate will be reported. The same stratification factors used for randomization will be applied to the stratified Cox model.

For the primary efficacy endpoints, participants without documented COVID-19 will be censored at the last study assessment date. Potential intercurrent events may include: 1) death unrelated to COVID-19 and 2) early COVID-19 up to 14 days after second study dose.

In the estimand of the primary analysis on the primary endpoint, a hypothetical strategy will be used for death unrelated to COVID-19; a hypothetical strategy will also be used for early COVID-19, where the time to COVID-19 will be censored at the onset day of early case. The details of intercurrent event description and estimand strategies are presented in Section 11.4.1.

For the primary efficacy analysis, cases will be counted starting 14 days after the second vaccination. Sensitivity analyses with cases counted immediately after the second vaccination, and after randomization will also be carried out.

Analyses of the primary endpoint will be also performed based on the mITT Set using the same methods described above.

For the primary efficacy analysis, cases will be counted starting 14 days after the second dose of IP. Sensitivity analyses with cases counted starting immediately after the second dose of IP and starting immediately after randomization will also be carried out.

Subgroup analysis of the primary efficacy endpoint will be performed in selected subgroups, such as age groups ≥ 18 and < 65 year and ≥ 65 years to assess consistency of VE as described in Section 9.5.5.

As a supportive analysis, VE will also be estimated by one minus the infection rate ratio, where the number of cases (ie, participants with first occurrence of COVID-19) will be used and the CI will be computed using the exact method conditional upon the total number of cases. Cumulative incidence VE, one minus the ratio of cumulative incidences (mRNA-1273 vs. placebo) of

COVID-19, may also be assessed, the cumulative incidence for each arm will be estimated using a covariate adjustment method based on Zeng (2004) that makes use of baseline characteristics.

Additional analysis to evaluate VE against COVID-19 incorporating duration and presence/severity of symptoms will also be performed; the details will be provided in the SAP.

9.5.1.2. Efficacy Analysis on Secondary Endpoint

For each of the below secondary objectives:

- Vaccine efficacy to prevent severe COVID-19
- Vaccine efficacy to prevent serologically confirmed SARS-CoV-2 infection or COVID-19 regardless of symptomatology or severity
- Vaccine efficacy to prevent COVID-19 using a broad definition of symptoms
- Vaccine efficacy to prevent death caused by COVID-19
- Vaccine efficacy to prevent asymptomatic SARS-CoV-2 infection

For each of the above secondary objectives, the same Cox proportional hazard model described above for the primary objective will be applied using the PP Set, with cases counted starting 14 days after the second dose of IP. Sensitivity analyses with cases counted starting after the second dose of IP, 14 days after the first dose of IP, after the first dose of IP, and after randomization will also be performed.

The same model will be applied using the mITT population with cases counted starting 14 days after the second dose of IP.

Vaccine efficacy will be estimated with 1- ratio of incidence rates with the 95% CI using the exact method conditional upon the total number of cases.

<u>Vaccine efficacy to prevent COVID-19 after the first dose of IP</u>

The same Cox proportional hazard model described above for the primary objective will be applied using the PP Set, with cases counted starting 14 days after the first dose of IP.

Vaccine efficacy to prevent COVID-19 regardless of prior SARS-CoV-2 infection

The FAS will be used for analysis to evaluate VE to prevent COVID-19 regardless of prior SARS-CoV-2 infection. The same methods described above for the primary objective will be applied with cases counted starting 14 days after the second dose of IP. Sensitivity analyses with cases counted starting after the second dose of IP, 14 days after the first dose of IP, the first dose of IP, and randomization will also be performed.

Vaccine efficacy will be estimated with 1 – ratio of incidence rates with the 95% CI using the exact method conditional upon the total number of cases.

9.5.1.3. Long-term Efficacy Analysis

Long-term efficacy of the primary vaccination series with mRNA-1273 will be evaluated after the primary analysis by including data collected in the Open-Label Observational Phase (Part B). Long-term efficacy data will be summarized descriptively by treatment cohort described in Table 10 without cohort comparison. The long-term efficacy endpoints are listed in

Table 11with the same case definitions specified inTable 1. In the primary approach, cases will be counted starting 14 days after the second dose of IP for participants in treatment cohorts of mRNA-1273 and placebo or starting 14 days after the second dose of mRNA-1273 for participants in the Placebo-mRNA-1273 Cohort. Sensitivity analyses with cases starting from the second dose, 14 days after the first dose, or first dose of IP for participants in the cohorts of mRNA-1273 and placebo, or mRNA-1273 for participants in the Placebo-mRNA-1273 Cohort may be provided. Incidences of cases assessed by numbers, rates, and 2-sided 95% CI based on the exact method adjusting for person-time will be summarized by treatment cohort. The Kaplan-Meier analysis will be used to estimate cumulative incidences of time to first cases by treatment cohort. Long-term efficacy analysis will be performed using the PP Set and mITT Set.

Efficacy analyses of unblinded/open-label phase data collected in Part B will be provided, as measured by the incidence rate of COVID-19 after the BD of mRNA-1273. The details of analysis of long-term efficacy and open-label phase data (Part B and Part C) will be provided in the SAP.

Table 10: Treatment Cohorts for the Long-term Efficacy Analyses

Treatment Cohort	Description
**mRNA-1273**	Participants randomized to mRNA-1273 in the Blinded Phase.
**Placebo**	Participants randomized to Placebo in the Blinded Phase and did not crossover to mRNA-1273 in the Open-Label Observational Phase.
**Placebo-mRNA-1273**	Participants randomized to Placebo in the Blinded Phase and crossed over to mRNA-1273 in the Open-Label Observational Phase.

Table 11: Long-term Efficacy Endpoints

Long-term Efficacy Endpoint
Cases of COVID-19 starting 14 days after the second injection of:<br>• IP for participants in the mRNA-1273 Cohort and the Placebo Cohort,<br>or<br>• mRNA-1273 for participants in the Placebo-mRNA-1273 Cohort.
Cases of severe COVID-19 starting 14 days after the second injection of:<br>• IP for participants in the mRNA-1273 Cohort and the Placebo Cohort,<br>or<br>• mRNA-1273 for participants in the Placebo-mRNA-1273 Cohort.
Cases of either COVID-19 or SARS-CoV-2 infection starting 14 days after the second injection of:<br>• IP for participants in the mRNA-1273 Cohort and the Placebo Cohort,<br>or<br>• mRNA-1273 for participants in the Placebo-mRNA-1273 Cohort.
Cases with a secondary (less restrictive) definition of COVID-19 starting 14 days after the second injection of:<br>• IP for participants in the mRNA-1273 Cohort and the Placebo Cohort,<br>or<br>• mRNA-1273 for participants in the Placebo-mRNA-1273 Cohort.
Cases of death due to a cause directly attributed to a complication of COVID-19, starting 14 days after the second injection of:<br>• IP for participants in the mRNA-1273 Cohort and Placebo Cohort,<br>or<br>• mRNA-1273 for participants in the Placebo-mRNA-1273 Cohort.
Cases of COVID-19 starting 14 days after the first injection of:<br>• IP for participants in the mRNA-1273 Cohort and the Placebo Cohort,<br>or<br>• mRNA-1273 for participants in the Placebo-mRNA-1273 Cohort.
Regardless of evidence of prior SARS-CoV-2 infection determined by serologic titer against SARS-CoV-2 nucleocapsid, cases of COVID-19 starting 14 days after the second injection of:<br>• IP for participants in the mRNA-1273 Cohort and the Placebo Cohort,<br>or<br>• mRNA-1273 for participants in the Placebo-mRNA-1273 Cohort.
Cases of SARS-CoV-2 infection in the absence of symptoms defining COVID-19 starting 14 days after the second injection of:<br>• IP for participants in the mRNA-1273 Cohort and the Placebo Cohort,<br>or<br>• mRNA-1273 for participants in the Placebo-mRNA-1273 Cohort.

Abbreviations: COVID-19 = coronavirus disease 2019; IP = investigational product; SARS-CoV-2 = severe acute respiratory syndrome coronavirus 2.

9.5.2. Safety Analyses

All safety analyses will be based on the Safety Set, except summaries of solicited ARs, which will be based on the Solicited Safety Set. All safety analyses will be provided by treatment group, and by treatment cohort as applicable, unless otherwise specified. Pregnancies and their known outcomes will be summarized (Section 8.3.6).

9.5.2.1. Adverse Events

Safety and reactogenicity will be assessed by clinical review of all relevant parameters. Safety analyses will be provided for Parts A, B, and C separately unless specified otherwise.

The number and percentage of participants with any solicited local AR, with any solicited systemic AR, and with any solicited AR during the 7-day follow-up period after each dose will be provided only for Part A. A 2-sided 95% exact CI using the Clopper-Pearson method will be also provided for the percentage of participants with any solicited AR for each treatment group. Analysis of solicited AR will be provided using the Solicited Safety Set for Part A.

The number and percentage of participants with unsolicited AEs (Part A [all participants] and Part C [only those who receive the BD]), SAEs, MAAEs, AESIs (Part C [only those who receive the BD]), and AEs leading to discontinuation from IP or withdrawal from the study will be summarized. Unsolicited AEs will be presented by MedDRA preferred term and system organ class. Analyses of AEs will be provided for Part A and Part B using the Safety Set. For Part C, analysis of AEs will be provided in the Part C Safety Set (ie, participants who received booster in Part C).

For all other safety parameters, descriptive summary statistics will be provided, and Table 12 summarizes the analysis strategy for safety parameters. Further details will be described in the SAP.

Table 12: Analysis Strategy for Safety Parameters in Part A

Safety Endpoint	Number and Percentage of Participants, Number of Events	95% CI
Any Solicited AR (Part A; overall and by local, systemic)	X	X
Any Unsolicited AE	X	
Any SAE	X	
Any Unsolicited MAAE	X	
Any Unsolicited Treatment-Related AE	X	
Any Treatment-Related SAE	X	
Discontinuation due to AE	X	
Any Grade 3 and above AE	X	
Any Treatment-Related Grade 3 and above AE	X	

Abbreviations: AE = adverse event; AR = adverse reaction; CI =confidence internval; MAAE = medically attended adverse event; MedDRA = Medical Dictionary for Regulatory Activities; SAE = serious adverse event.

Notes: 95% CI using the Clopper-Pearson method, X = results will be provided. Unsolicited AEs will be summarized by System Organ Class and Preferred Term coded by MedDRA. Safety parameters will be analyzed in applicable study parts and participants as further specified in the statistical analysis plan.

9.5.2.2. Baseline Descriptive Statistics

Demographic variables and baseline characteristics will be summarized by treatment group, and by treatment cohort as applicable, by descriptive statistics (mean, standard deviation for continuous variable, and number and percentage for categorical variables).

9.5.3. Immunogenicity Analyses

The secondary immunogenicity endpoints will be analyzed using the Immunogenicity Subset by treatment group, by treatment cohort as applicable, and by baseline SARS-CoV-2 serostatus, unless otherwise specified. Details for immunogenicity analyses will be provided in the SAP.

The SAP will describe the complete set of immunogenicity analyses, including the approach to sample individuals into an Immunogenicity Subset for characterizing mRNA-1273 immunogenicity and assessing immunological correlates of risk and protection.

9.5.3.1. Part A

Data from quantitative immunogenicity assays will be summarized for each treatment group using positive response rates and geometric means with 95% Cis, for each timepoint for which an assessment is performed. Data from qualitative (ie, yielding a positive or negative result) assays will be summarized by tabulating the frequency of positive responses for each assay by group at each timepoint that an assessment is performed. Analyses will focus on the 2 key immunogenicity

timepoints and the change in marker response between them: Day 1 before the first dose of IP and Day 57 (28 days after the second dose of IP). The SAP will describe the complete set of immunogenicity analyses.

Quantitative levels or geometric mean titer (GMT) of specific bAb with corresponding 95% CI at each timepoint and geometric mean fold rise (GMFR) of specific bAb with corresponding 95% CI at each postbaseline timepoint over pre-dose baseline at Day 1 will be provided by study arm. Descriptive summary statistics including median, minimum, and maximum will also be provided.

The GMT of specific nAb with corresponding 95% CI at each timepoint and GMFR of specific nAb with corresponding 95% CI at each postbaseline timepoint over pre-dose baseline at Day 1 will be provided by study arm. Descriptive summary statistics including median, minimum, and maximum will also be provided. For summarizations of group values, Ab values reported as below the LLOQ will be replaced by 0.5 × LLOQ. Values that are reported as greater than the upper limit of quantification (ULOQ) without the actual values will be converted to the ULOQ.

The number and percentage of participants with a fold rise ≥ 2, ≥ 3, and ≥ 4 of serum SARS-CoV-2-specific nAb titers and participants with seroresponse from baseline will be provided with 2-sided 95% CI using the Clopper-Pearson method at each post-baseline timepoint.

Seroresponse at a participant level may be defined as a change from below the LLOQ to at least 4 times the LLOQ, or at least a 4-fold rise in nAb or vaccine antigen-specific bAb in participants with pre-existing nAb or bAb of at least the LLOQ at baseline/pre-vaccination. Seroresponse may also be defined for each specific assay assessing nAb or bAb. The definition of seroresponse will be finalized and documented in the SAP.

The GMT of specific nAb for each group and the geometric mean ratio (GMR) of mRNA-1273 versus placebo in Part A with corresponding 2-sided 95% CI will be estimated at each study timepoint using an analysis of covariance model with the treatment group and baseline values, if applicable, as explanatory variables, the analysis may adjust for the stratification factor (Table 13).

Table 13: Immunogenicity Endpoints and Statistical Methods

Endpoint	Statistical Analysis Methods
Specific bAb and nAb titers/values	• GMT of each group, GMR (mRNA-1273 vs. placebo)<br>• GMT estimated by the ANCOVA model
Fold rise	• GMFR – descriptive statistics<br>• Binomial endpoints of fold rise ≥ 2, 3, and 4, and seroconversion due to vaccination – the Clopper-Pearson method

Abbreviations: ANCOVA = analysis of covariance; bAb = binding antibody; GMFR = geometric mean fold rise, GMR = geometric mean ratio; GMT = geometric mean titer; nAb = neutralizing antibody.

9.5.3.2. Part C

In Part C, the immunogenicity analysis of BD vaccine response will be performed using the noninferiority tests of the 2 null hypotheses based on the 2 key secondary endpoints, respectively. Further considerations for immunogenicity analyses are provided in the SAP.

Part C Key Secondary Endpoint 1: Ab GMT at BD-Day 29

Hypothesis: immunogenicity response to mRNA-1273 BD as measured by Ab GMT at BD-Day 29 in Part C is noninferior compared with Ab GMT at Day 57 (28 days after second dose) in the primary series of mRNA-1273.

The noninferiority of post-booster GMT at BD-Day 29 in Part C as compared to the primary series at Day 57 is considered to be demonstrated if the lower bound of the 95% CI of the GMR (ratio of GMT at BD-Day 29 vs. GMT at Day 57 [28 days after Dose 2 of the primary series]) is ≥ 0.67 using a noninferiority margin of 1.5 (1.5-fold immuno-bridging margin for the lower bound of the 95% CI for GMT ratio/GMR).

The GMT with 95% CI will be summarized using t-distribution of the log transferred values and then back transformed to the original scale. The GMR with 95% CI to compare post-booster GMT at BD-Day 29 in Part C with the primary series GMT at Day 57 (28 days after the second dose) will be computed based on the t-distribution of mean difference in the log transferred values and then back transformed to the original scale.

Part C Key Secondary Endpoint 2: Ab Seroresponse Rate at BD-Day 29

Hypothesis: immunogenicity response to mRNA-1273 BD as measured by seroresponse rate (SRR) at BD-Day 29 in Part C is noninferior compared with SRR of the primary series at Day 57 (28 days after second dose of the primary series of mRNA-1273).

The noninferiority in SRR post-booster at BD-Day 29 in Part C compared with SRR of the primary series at Day 57 (28 days after the second dose of the primary series of mRNA-1273) is considered to be demonstrated if the lower bound of the 95% CI of the SRR difference (SRR of the booster at BD-Day 29 – SRR of the primary series at Day 57) is > –10% (using the noninferiority margin of 10%). The SRR difference is defined as the seroresponse rate at BD-Day 29 in Part C minus the seroresponse rate at Day 57 (28 days after the second dose) following the primary series of mRNA-1273. The seroresponse of a booster or the primary series is defined as a titer change from baseline (pre-Dose 1 in primary series) below the LLOQ to ≥ 4 × LLOQ, or at least a 4-fold rise if baseline is ≥ LLOQ.

The SRR with 95% CI (using Clopper-Pearson method) will be provided. The SRR difference with 95% CI to compare post-booster SRR at BD-Day 29 in Part C with the primary series SRR at

Day 57 (28 days after the second dose) will be computed. The method for computing 95% CI of seroresponse rate difference will be provided in the SAP.

The primary immunogenicity objective in Part C is met if noninferiority is demonstrated based on both key secondary endpoints (both GMT and SRR) .

9.5.4. Exploratory Analyses in Part A

The endpoint of viral infection kinetics will be assessed by determining the number of days until testing of saliva samples becomes negative after COVID-19 is established.

The endpoint of duration of symptoms will be assessed by determining the total number of days that a study participant with COVID-19 remains symptomatic through daily assessments after diagnosis.

Vaccine efficacy to prevent all-cause mortality will be assessed by similar analysis methods as used for the primary endpoint analysis, using PP Set, mITT Set, and FAS. All deaths, regardless of cause from the time of randomization, will be included. If the number of deaths becomes large enough to warrant analysis, the same Cox proportional hazard model described above for the primary objective will be applied using the PP Set, the mITT Set, and the FAS. Death, regardless of cause, from randomization will be included.

This endpoint of burden of disease (BOD) is defined based on the post SARS-CoV-2 infection follow-up. A BOD score will be used to reflect the severity of symptoms with maximum score at COVID-19 death (Table 14).

Table 14: Burden of Disease Score

Patient State	BOD Score
Uninfected/Asymptomatic infection	0
Symptomatic without hospitalization	1
Hospitalization	2
Death	3

Abbreviation: BOD = burden of disease.

9.5.5. Subgroup Analyses

To determine whether the VE is consistent across various subgroups, the VE and its 95% CI may be estimated using the similar model within each category of the following classification variables.

- Age groups: ≥ 18, < 65, and ≥ 65
- Age and health risk for severe disease: ≥ 18 and < 65 and not at risk; ≥ 18 and < 65 and at risk, and ≥ 65

- Sex (female, male)
- Race
- At risk for severe COVID-19 illness (Section 6.2.1.1)

Subgroup analysis for the long-term efficacy may be performed descriptively by treatment cohort within above categories.

9.6. Interim Analyses

In Part A, there are 2 planned IAs at 35% and 70% of total target cases across the 2 treatment groups. The primary objective of the IAs is for early detection of reliable evidence that VE is above 30%. The Lan-DeMets O'Brien-Fleming approximation spending function is used for calculating efficacy bounds and to preserve the (1-sided) 0.025 false positive error rate over the IAs and the primary analysis (when the target number of cases have been observed), relative to the hypothesis:

$H_0^{efficacy}$: HR $\geq$ 0.7 (equivalently, proportional hazards VE $\leq$ 0.3).

There is no intention to stop the study early if the efficacy has been demonstrated at any of the IAs. If efficacy is demonstrated at an IA, the subsequent IA or primary analysis will be considered supportive in nature. The DSMB will review the IA results and make recommendations to the Sponsor in terms of study results reporting and unblinding based on the boundaries of early efficacy as described in this section, safety data, and data external to this study. In addition to possible early efficacy at IAs, the DSMB will monitor for non-efficacy and vaccine harm; the guiding principles (non-binding) is provided in Section 8.4.2, and the details will be provided in the SAP.

Table 15 summarizes the timing, number of cases and decision guidance at each IA and primary analysis.

The first IA will occur when approximately 35% of the total cases have been observed (across both treatment groups). The study will be considered positive at the first IA if the p-value for rejecting HR $\geq$ 0.7 is less than 0.0002 based on the Lan-DeMets O'Brien-Fleming approximation spending function. This corresponds to an observed HR of approximately 0.259, or an observed VE approximately 0.741.

The second IA will occur when approximately 70% of the total cases have been observed. The study will be considered positive (VE has been demonstrate) if the p-value for rejecting HR $\geq$ 0.7 is less than 0.0073 based on the Lan-DeMets O'Brien-Fleming approximation spending function. This corresponds to an observed HR of approximately 0.435, or an observed VE of approximately 0.565.

The primary analysis will be performed when approximately 151 cases have been observed in the study. The study will be considered positive at the primary analysis if the 1-sided p-value for rejecting HR ≥ 0.7 is less than 0.0227. This corresponds to an observed HR of approximately 0.505 or observed VE of approximately 0.495.

Table 15: Interim Boundaries Using O'Brien-Fleming Spending Function, Calculation Based on the PP Set for the Primary Efficacy Endpoint

Information Fraction (% of total #cases)	Number of Cases	Nominal Alpha	Efficacy Boundary Rejecting H0: VE ≤ 30%	Cum Prob (crossing efficacy boundary if the true VE = 60%)
IA1 35%	53	0.0002	VE ≥ 0.741<br>(HR ≤ 0.259)	4.6%
IA2 70%	106	0.0073	VE ≥ 0.565<br>(HR ≤ 0.435)	61.5%
Primary analysis 100%	151	0.0227	VE ≥ 0.495<br>(HR ≤ 0.505)	90.0%

Abbreviations: HR = hazard ratio; IA: = interim analysis; PP = per-protocol; VE = vaccine efficacy.

10. REFERENCES

Adler Y, Charron P, Imazio M, Badano L, Barón-Esquivias G, Bogaert J, et al. 2015 ESC guidelines for the diagnosis and management of pericardial diseases: the task force for the diagnosis and management of pericardial diseases of the European Society of Cardiology (ESC) endorsed by: The European Association for Cardio-Thoracic Surgery (EACTS). Eur Heart J. 2015;36(42):2921-64.

Anderson K. gsDesign, version 3.1.1. 2020. Available from The Comprehensive R Archive Network at https://cran.r-project.org/web/packages/gsDesign/.

Aretz HT. Myocarditis: the Dallas criteria. Hum Pathol. 1987;18(6):619-24.

Baden LR, El Sahly HM, Essink B, Kotloff K, Frey S, Novak R, et al. Efficacy and safety of the mRNA-1273 SARS-CoV-2 vaccine. N Engl J Med. 2020. Doi: 10.1056/NEJMoa2035389. Online ahead of print.

Centers for Disease Control and Prevention (CDC). 2020a. Coronavirus Disease 2019 (COVID-19). Retrieved from: https://www.cdc.gov/coronavirus/2019-ncov/cases-updates/cases-in-us.html. (accessed 2020 May 29).

Centers for Disease Control and Prevention (CDC). 2020b. Coronavirus Disease 2019 (COVID-19). Retrieved from: https://www.cdc.gov/coronavirus/2019-ncov/need-extra-precautions/people-at-higher-risk.html (accessed 2020 May 29).

Centers for Disease Control and Prevention (CDC). 2020c. Coronavirus Disease 2019 (COVID-19). Retrieved from: https://www.cdc.gov/coronavirus/2019-ncov/symptoms-testing/symptoms.html (accessed 2020 Jun 11).

Chen Y, Lu S, Jia H, Deng Y, Zhou J, Huang B, et al. A novel neutralizing monoclonal antibody targeting the N-terminal domain of the MERS-CoV spike protein. Emerg Microbes Infect. 2017;6(5):e37.

Chin J, Magoffin RL, Shearer LA, Schieble JH, Lennette EH. Field evaluation of a respiratory syncytial virus vaccine and a trivalent parainfluenza virus vaccine in a pediatric population. Am J Epidemiol 1969;89:449–63.

ClinicalTrials.gov [Internet]. Bethesda (MD): National Library of Medicine (US). – Identifier NCT03076385, Safety, tolerability, and immunogenicity of VAL-506440 in healthy adult subjects. Retrieved from: https://clinicaltrials.gov/ct2/keydates/NCT03076385. (accessed 2020 Jun 01).

ClinicalTrials.gov [Internet]. Bethesda (MD): National Library of Medicine (US). – Identifier NCT03325075, Safety, tolerability, and immunogenicity of VAL-181388 in healthy subjects. Retrieved from: https://clinicaltrials.gov/ct2/keydates/NCT03325075. (accessed 2020 Jun 01).

ClinicalTrials.gov [Internet]. Bethesda (MD): National Library of Medicine (US). – Identifier NCT03345043, Safety, tolerability, and immunogenicity of VAL-339851 in healthy adult subjects. Retrieved from: https://clinicaltrials.gov/ct2/keydates/NCT03345043. (accessed 2020 Jun 01).

ClinicalTrials.gov [Internet]. Bethesda (MD): National Library of Medicine (US). – Identifier NCT03382405, Safety, reactogenicity, and immunogenicity of cytomegalovirus vaccines mRNA-1647 and mRNA-1443 in healthy adults. Retrieved from: https://clinicaltrials.gov/ct2/keydates/NCT03382405. (accessed 2020 Jun 01).

ClinicalTrials.gov [Internet]. Bethesda (MD): National Library of Medicine (US). – Identifier NCT03392389, Safety, reactogenicity, and immunogenicity of mRNA-1653 in healthy adults. Retrieved from: https://clinicaltrials.gov/ct2/keydates/NCT03392389. (accessed 2020 Jun 01).

ClinicalTrials.gov [Internet]. Bethesda (MD): National Library of Medicine (US). – Identifier NCT04283461, Safety and immunogenicity study of SARS-CoV-2 vaccine (mRNA-1273) to treat novel coronavirus; 2020 Feb 22. Retrieved from: https://clinicaltrials.gov/ct2/keydates/NCT04283461. (accessed 2020 Jun 01).

ClinicalTrials.gov [Internet]. Bethesda (MD): National Library of Medicine (US). – Identifier NCT04405076, Dose-Confirmation Study to Evaluate the Safety, Reactogenicity, and Immunogenicity of mRNA-1273 COVID-19 Vaccine in Adults Aged 18 Years and Older. Retrieved from: https://clinicaltrials.gov/ct2/show/NCT04405076. (accessed 2022 Feb 22).

Corti D, Zhao J, Pedotti M, Simonelli L, Agnihothram S, Fett C, et al. Prophylactic and postexposure efficacy of a potent human monoclonal antibody against MERS coronavirus. Proc Natl Acad Sci U S A. 2015;112(33):10473-8.

Department of Health and Human Services (DHHS), Food and Drug Administration, Center for Biologics Evaluation and Research (US). Guidance for industry: Toxicity grading scale for healthy adult and adolescent volunteers enrolled in preventative vaccine clinical trials. September 2007 [cited 2019 Apr 10] [10 screens]. Available from: https://www.fda.gov/downloads/BiologicsBloodVaccines/GuidanceComplianceRegulatoryInformation/Guidances/Vaccines/ucm091977.pdf.

Department of Health and Human Services (DHHS), Food and Drug Administration, Center for Biologics Evaluation and Research (US). Guidance Document. FDA guidance on conduct of clinical trials of medical products during COVID-19 public health emergency. March 2020a. Updated on April 16, 2020. [cited 2020 Apr 17]. Available from: https://www.fda.gov/media/136238/download.

Department of Health and Human Services (DHHS), Food and Drug Administration, Center for Drug Evaluation and Research (US). Guidance for industry: Enhancing the Diversity of Clinical

Trial Populations – Eligibility Criteria, Enrollment Practices, and Trial Designs. November 2020b. Available from: https://www.fda.gov/media/127712/download.

Department of Health and Human Services (DHHS), Food and Drug Administration, Center for Biologics Evaluation and Research (US). Guidance for industry: Emergency Use Authorization for Vaccines to Prevent COVID-19. May 2021. Available from: https://www.fda.gov/media/142749/download.

Ferreira VM, Schulz-Menger J, Holmvang G, Kramer CM, Carbone I, Sechtem U, et al. Cardiovascular magnetic resonance in nonischemic myocardial inflammation: expert recommendations. J Am Coll Cardiol. 2018;72(24):3158-76.

Fulginiti VA, Eller JJ, Downie AW, Kempe CH. Altered reactivity to measles virus. Atypical measles in children previously immunized with inactivated measles virus vaccines. JAMA. 1967;202:1075-80.

Gargano JW, Wallace M, Hadler SC, Langley G, Su JR, Oster ME, et al. Use of mRNA COVID-19 vaccine after reports of myocarditis among vaccine recipients: update from the advisory committee on immunization practices – United States, June 2021. MMWR Morb Mortal Wkly Rep. 2021;70(27):977-82.

Jackson LA, Anderson EJ, Rouphael NG, Roberts PC, Makhene M, Coler RN, et al. An mRNA vaccine against SARS-CoV-2 – preliminary report. N Engl J Med. 2020;383(20):1920-31.

Johnson RF, Bagci U, Keith L, Tang X, Mollura DJ, Zeitlin L, et al. 3B11-N, a monoclonal antibody against MERS-CoV, reduces lung pathology in rhesus monkeys following intratracheal inoculation of MERS-CoV Jordan-n3/2012. Virology. 2016;490:49-58.

Kim Y, Lee H, Park K, Park S, Lim JH, So MK, et al. Selection and characterization of monoclonal antibodies targeting Middle East respiratory syndrome coronavirus through a human synthetic fab phage display library panning. Antibodies (Basel). 2019;8(3).

Rüggeberg JU, Gold MS, Bayas JM, Blum MD, Bonhoeffer J, Friedlander S, et al. Anaphylaxis: case definition and guidelines for data collection, analysis, and presentation of immunization safety data. Vaccine. 2007;25(31):5675-84.

Thomas SJ and Yoon IK. A review of Dengvaxia®: development to deployment. Hum Vaccin Immunother. 2019;15(10), 2295-2314.

Wang L, Shi W, Joyce MG, Modjarrad K, Zhang Y, Leung K, et al. Evaluation of candidate vaccine approaches for MERS-CoV. Nat Commun. 2015;6:7712.

Wang L, Shi W, Chappell JD, Joyce MG, Zhang Y, Kanekiyo M, et al. Importance of neutralizing monoclonal antibodies targeting multiple antigenic sites on the Middle East respiratory syndrome coronavirus spike glycoprotein to avoid neutralization escape. J Virol. 2018;92(10).

Widjaja I, Wang C, van Haperen R, Gutiérrez-Álvarez J, van Dieren B, Okba NMA, et al. Towards a solution to MERS: protective human monoclonal antibodies targeting different domains and functions of the MERS-coronavirus spike glycoprotein. Emerg Microbes Infect. 2019;8(1):516-30.

World Health Organization (WHO). Dengue vaccine: WHO position paper, September 2018 – recommendations. Vaccine. 2019;37(35):4848-9.

World Health Organization (WHO). 2020 (Data reported as of 2020, May 28). Coronavirus disease 2019 (COVID-19) Situation Report – 129. Retrieved from: https://www.who.int/docs/default-source/coronaviruse/situation-reports/20200528-covid-19-sitrep-129.pdf?sfvrsn=5b154880_2. (accessed 2020 May 29).

Wrapp D, Wang N, Corbett KS, Goldsmith JA, Hseih CL, Abiona O, et al. Cryo-EM structure of the 2019-nCoV spike in the prefusion conformation. Science. 2020;367:1260-3.

Yu X, Zhang S, Jiang L, Cui Y, Li D, Wang D, et al. Structural basis for the neutralization of MERS-CoV by a human monoclonal antibody MERS-27. Sci Rep. 2015;5:13133.

Zeng D. Estimating marginal survival function by adjusting for dependent censoring using many covariates. Ann Statist. 2004;32(4):1533-55.

Zent O, Arras-Reiter C, Broeker M, Hennig R. Immediate allergic reactions after vaccinations – a post-marketing surveillance review. Eur J Pediatr. 2002;161(1):21-25.

11. SUPPORTING DOCUMENTATION AND OPERATIONAL CONSIDERATIONS

11.1. APPENDIX 1: Schedules of Events

If a participant cannot attend a study site visit (scheduled or unscheduled, with the exception of Screening, Day 1, Day 29, PDV, OL-D1, OL-D29, and BD-1), a home visit is acceptable if performed by appropriately delegated study site staff or a home healthcare service provided by the Sponsor. If neither a participant visit to the study site nor a home visit to the participant is possible (with the exception of Screening, Day 1, Day 29, PDV, OL-D1, OL-D29, and BD-1), a safety phone call should be performed that includes the assessments scheduled for the biweekly safety phone calls (Table 16).

The Supplemental SoEs (Table 21 and Table 23) and the Modified Supplemental SoE (Table 22) are intended to occur in addition to the original SoEs, as applicable (Table 16, Table 17Table 18Table 19), and therefore there is a possibility for study visits to overlap. If visits overlap according to respective visit windows, a single visit may be completed with any duplicated study procedures each completed once (refer to Table 4 footnotes for more detailed instructions and exceptions).

Table 16: Schedule of Events (Vaccination Phase, Day 1 – Day 57)

Visit Number	0	1				2				3
**Type of Visit**	C	C	SC	SC	SC	C	SC	SC	SC	C
**Month/Weekly Timepoint**		M0				M1				M2
**Study Visit Day**	D0[1] (Screening)	D1 (Baseline)	7 days after D1 D8	14 days after D1 D15	21 days after D1 D22	D29	7 days after D29 D36	14 days after D29 D43	21 days after D29 D50	D57
**Window Allowance (Days)**	-28		+3	+3	+3	-3/+7	+3	+3	+3	-3/+7
**Days Since Most Recent Vaccination**	-	0	7	14	21	28/0	7	14	21	28
ICF, demographics, concomitant medications, medical history	X									
Confirm participant meets inclusion and exclusion criteria	X	X								
Physical examination[2]	X	X				X				X
Pregnancy testing[3]	X	X				X				
Randomization		X								
**Dosing**										
Study injection (including 30-minute post-dosing observation period)		X				X				
**Efficacy Assessment**										
Surveillance for COVID-19/Unscheduled Visit[4]		X	X	X	X	X	X	X	X	X
Nasopharyngeal swab[5]		X				X				
**Immunogenicity Assessment**										
Blood for immunologic analysis[5]		X				X				X

Visit Number	0	1				2				3
Type of Visit	C	C	SC	SC	SC	C	SC	SC	SC	C
Month/Weekly Timepoint		M0				M1				M2
**Study Visit Day**	D0[1] (Screening)	D1 (Baseline)	7 days after D1 D8	14 days after D1 D15	21 days after D1 D22	D29	7 days after D29 D36	14 days after D29 D43	21 days after D29 D50	D57
**Window Allowance (Days)**	-28		+3	+3	+3	-3/+7	+3	+3	+3	-3/+7
**Days Since Most Recent Vaccination**	-	0	7	14	21	28/0	7	14	21	28
**Safety Assessments**										
eDiary activation for recording solicited adverse reactions (7 days)[6]		X				X				
Review of eDiary			X				X			
Follow-up safety[7]			X	X	X		X	X	X	
Recording of unsolicited AEs		X	X	X	X	X	X	X	X	X
Recording of MAAEs, AE leading to withdrawal, and concomitant medications relevant to or for the treatment of the MAAE[8]		X	X	X	X	X	X	X	X	X
Recording of SAEs and concomitant medications relevant to or for the treatment of the SAE[8]	X	X	X	X	X	X	X	X	X	X
Recording of concomitant medications and non-study vaccinations[8]		X	X	X	X	X	X	X	X	X

Abbreviations: AE = adverse event; AR = adverse reaction; C = clinic visit; COVID-19 = coronavirus disease 2019; D = day; eDiary = electronic diary; FDA = Food and Drug Administration; ICF = informed consent form; M = month; MAAE = medically attended AE; NP = nasopharyngeal; RT-PCR = reverse transcriptase polymerase chain reaction; SAE = serious adverse event; SARS-CoV-2 = severe acute respiratory syndrome coronavirus 2; SC = safety (phone) call.

Note: In accordance with FDA Guidance on Conduct of Clinical Trials of Medical Products during the COVID-19 Pandemic (DHHS 2020a), investigators may convert study site visits to telemedicine visits with the approval of the Sponsor. All scheduled study visits should be completed within the respective visit windows. If the participant is not able to come on-site for a study site visit as a result of the COVID-19 pandemic (self-quarantine or disruption of study site activities following business continuity plans and/or local government mandates for “stay at home” or “shelter in place”), a safety call to the participant should be made in place of the study site visit. The safety call should encompass all scheduled visit assessments that can be completed remotely, such as assessment for AEs and concomitant medications (eg, as defined in scheduled biweekly safety phone calls). Home visits will be permitted for all non-dosing visits except for Screening if a participant cannot come to the study site as a result of the COVID-19 pandemic.

1. Day 0 and Day 1 may be combined the same day. Additionally, the Day 0 visit may be performed over multiple visits if within the 28-day screening window.
2. Physical examination: a full physical examination, including vital signs, height, and weight, will be performed at Screening and Day 1. Body mass index will be calculated at the Screening Visit (Day 0) only. Symptom-directed physical examinations will be performed on Day 29 and Day 57. On each dosing day before injection, the arm receiving the injection should be examined and the associated lymph nodes should be evaluated. Any clinically significant finding identified during a study visit should be reported as an MAAE. Vital signs are to be collected pre- and post-dosing on days of injection (Day 1 and Day 29). When applicable, vital sign measurements should be performed before blood collection. Participants who are febrile (body temperature ≥ 38.0°C/100.4°F) before dosing on Day 1 or Day 29 must be rescheduled within the relevant window period to receive the injection. Afebrile participants with minor illnesses can be enrolled at the discretion of the investigator.
3. Pregnancy test at Screening and Day 1 and before the second vaccination will be a point-of-care urine test. At the discretion of the investigator a pregnancy test either via blood or point-of-care urine test can be performed. Follicle-stimulating hormone level may be measured to confirm menopausal status at the discretion of the investigator.
4. Participants with symptoms of COVID-19 will be asked to return within 72 hours or as soon as possible to the study site for an unscheduled visit, to include an NP swab sample (for RT-PCR testing) and other clinical evaluations. If a study site visit is not possible, a home visit may be arranged to collect the NP swab sample and conduct clinical evaluations. If a home visit is not possible, the participant will be asked to submit a saliva sample to the study site by a Sponsor-approved method. At this visit, an NP swab and blood sample will be collected to evaluate for the presence of SARS-CoV-2 infection. An additional NP swab sample will be collected to test for the presence of other respiratory pathogens. Additionally, clinical information will be carefully collected to evaluate the severity of the clinical case. It is important to note that some of the symptoms of COVID-19 overlap with solicited systemic ARs that are expected after vaccination with mRNA-1273 (eg, myalgia, headache, fever, and chills). During the first 7 days after vaccination, when these solicited ARs are common, investigators should use their clinical judgement to decide if an NP swab should be collected. The collection of an NP swab prior to the Day 1 and Day 29 vaccination can help ensure that cases of COVID-19 are not overlooked. Any study participant reporting respiratory symptoms during the 7-day period after vaccination should be evaluated for COVID-19.
5. Sample must be collected prior to dosing on days of injection (Day 1 and Day 29).
6. The participant will record entries in the eDiary approximately 30 minutes after dosing while at the study site, with instruction provided by study staff. Study participants will continue to record in the eDiary each day after they leave the study site, preferably in the evening, on the day of dosing and for 6 days following. Any solicited AR that is ongoing beyond Day 7 will be reported until resolution. Adverse reactions recorded in eDiaries beyond Day 7 should be reviewed either via phone call or at the following study visit. Participants will be given thermometers to record their temperatures and rulers to measure any injection site reactions.
7. Trained study personnel will call all participants to collect information relating to any unsolicited AEs through Day 57 (including any signs and symptoms of COVID-19), MAAEs, AEs leading to withdrawal, SAEs, information on concomitant medications associated with those events, and any non-study vaccinations.
8. All concomitant medications and non-study vaccinations will be recorded through 28 days after each injection; all concomitant medications relevant to or for the treatment of an SAE or MAAE will be recorded from Screening through the final visit (Day 759).

Table 17: Schedule of Events (Surveillance Phase, Day 64 – Day 394)

**Visit Number**						4						5
**Type of Visit**	eDiary	SC	SC	SC	SC	C	SC	SC	SC	SC	SC	C
**Month (M)/Weekly (W) Timepoint**	W	M3	M4	M5	M6	M7	M8	M9	M10	M11	M12	M13
**Study Visit Day**	D64[1]	D85[1]	D119 [1] 90 days after D29	D149[1]	D179[1]	D209[1] 180 days after D29	D239[1]	D269[1]	D299[1]	D329[1]	D359[1]	D394[1] 365 days after D29
**Window Allowance (Days)**	±2	±3	±3	±3	±3	±14	±3	±3	±3	±3	±3	±14
**Days Since Most Recent Vaccination**	-	56	90	120	150	180	210	240	270	300	330	365
Physical examination[2]						X						X
**Efficacy Assessments**												
eDiary activation for surveillance for COVID-19/Unscheduled Visit	X											
eDiary Weekly prompts for surveillance for COVID-19	-----------------Weekly eDiary prompts (Day 64 through Day 759)------------------											
Surveillance for COVID-19/Unscheduled Visit[3]		X	X	X	X	X	X	X	X	X	X	X
**Immunogenicity Assessment**												
Blood for immunologic analysis						X						X
**Safety Assessments**												
eDiary activation for safety follow-up	X[4]											
eDiary Weekly prompts for safety follow-up	--------------Weekly eDiary prompts (Day 64 through Day 759)---------------------											

Visit Number						4						5
**Type of Visit**	eDiary	SC	SC	SC	SC	C	SC	SC	SC	SC	SC	C
**Month (M)/Weekly (W) Timepoint**	W	M3	M4	M5	M6	M7	M8	M9	M10	M11	M12	M13
**Study Visit Day**	D64[1]	D85[1]	D119 [1] 90 days after D29	D149[1]	D179[1]	D209[1] 180 days after D29	D239[1]	D269[1]	D299[1]	D329[1]	D359[1]	D394[1] 365 days after D29
**Window Allowance (Days)**	±2	±3	±3	±3	±3	±14	±3	±3	±3	±3	±3	±14
**Days Since Most Recent Vaccination**	-	56	90	120	150	180	210	240	270	300	330	365
Follow-up safety[5]		X	X	X	X	X	X	X	X	X	X	X
Recording of MAAEs, AESIs (Part C), AE leading to withdrawal and concomitant medications relevant to or for the treatment of the MAAE[6]		X	X	X	X	X	X	X	X	X	X	X
Recording of SAEs and concomitant medications relevant to or for the treatment of the SAE[6]		X	X	X	X	X	X	X	X	X	X	X
Recording of concomitant medications and non-study vaccinations[6,7]		X	X	X	X	X	X	X	X	X	X	X

Abbreviations: AE = adverse event; AESI = adverse event of special interest; C = clinic visit; COVID-19 = coronavirus disease 2019; D = day; eDiary = electronic diary; ePRO = electronic patient-reported outcome; FDA = Food and Drug Administration; IRB = institutional review board; M = month; MAAE = medically attended AE; NP= nasopharyngeal swab; RT-PCR = reverse transcriptase polymerase chain reaction; SAE = serious adverse event; SARS-CoV-2 = severe acute respiratory syndrome coronavirus 2; SC = safety (phone) call; W = week.

Note: In accordance with FDA Guidance on Conduct of Clinical Trials of Medical Products during the COVID-19 Pandemic (DHHS 2020a), investigators may convert study site visits to telemedicine visits with the approval of the Sponsor.

1. All scheduled study visits should be completed within the respective visit windows. If the participant is not able to come on-site for a study site visit as a result of the COVID-19 pandemic (self-quarantine or disruption of study site activities following business continuity plans and/or local government mandates for “stay at home” or “shelter in place”), a safety phone call to the participant should be made in place of the study site visit. The safety call should encompass all scheduled visit assessments that can be completed remotely, such as assessment for AEs and concomitant medications (eg, as defined in scheduled safety phone calls). Home visits will be permitted for all non-dosing visits except for Screening if a participant cannot come to the study site as a result of the

COVID-19 pandemic. Home visits must be permitted by the site IRB and the participant via informed consent and have prior approval from the Sponsor (or its designee).

2. Symptom-directed physical examinations may be performed at the discretion of the investigator.
3. Participants with symptoms of COVID-19 will be asked to return within 72 hours or as soon as possible to the study site for an unscheduled visit, to include an NP swab sample (for RT-PCR testing) and other clinical evaluations. If a study site visit is not possible, a home visit may be arranged to collect the NP swab sample and conduct clinical evaluations. If a home visit is not possible, the participant will be asked to submit a saliva sample to the study site by a Sponsor-approved method. At this visit, an NP swab and blood sample will be collected to evaluate for the presence of SARS-CoV-2 infection. An additional NP swab sample will be collected to test for the presence of other respiratory pathogens. In addition, the study site may collect an additional (local/non-central) respiratory sample for SARS-CoV-2 testing to be able to render appropriate medical care for the study participant as determined by local standards of care. Additionally, clinical information will be carefully collected to evaluate the severity of the clinical case.
4. The eDiary Safety Follow-up prompts will be triggering off of Day 61 to take into consideration the (-3 day) window allowance at Day 29. This first Safety Follow-Up prompt at Day 61 will follow the same 7-day surveillance per protocol.
5. Trained study personnel will call all participants to collect information relating to any sign/symptoms of COVID-19, MAAEs, AESIs, AEs leading to withdrawal, SAEs, information on concomitant medications associated with those events, and any non-study vaccinations.
6. All concomitant medications relevant to or for the treatment of an SAE or MAAE will be recorded from Screening through the final visit (Day 759).
7. One additional ePRO prompt will be sent to participants specifically to solicit the collection of information regarding participant’s history of facial injections or dermal fillers, for cosmetic or medical indications such as migraine headaches.

Table 18: Schedule of Events (Surveillance Phase, Day 401 – Day 759)

**Visit Number**						6
**Type of Visit**	SC	SC	SC	SC	SC	C
**Month (M)/Weekly (W) Timepoint**	M15	M17	M19	M21	M23	M25
**Study Visit Day**	D454[1]	D514[1]	D574[1]	D634[1]	D694[1]	D759[1] 365 days after Year 1
**Window Allowance (Days)**	±7	±7	±7	±7	±7	-14/+28
**Days Since Most Recent Vaccination**						730
Physical examination[2]						X
eDiary weekly prompts for surveillance for COVID-19	----Weekly eDiary prompts (Day 401 through Day 759)------------					
Surveillance for COVID-19/Unscheduled Visit[3]	X	X	X	X	X	X
Blood for immunologic analysis						X
eDiary weekly prompts for safety follow-up	-----Weekly eDiary prompts (Day 401 through Day 759)-----------					
Follow-up safety[4]	X	X	X	X	X	X
Recording of MAAEs, AESIs (Part C), AE leading to withdrawal and concomitant medications relevant to or for the treatment of the MAAE[5]	X	X	X	X	X	X
Recording of SAEs and concomitant medications relevant to or for the treatment of the SAE[5]	X	X	X	X	X	X
Recording of concomitant medications and non-study vaccinations[5]	X	X	X	X	X	X

Abbreviations: AE = adverse event; AESI = adverse event of special interest; C = clinic visit; COVID-19 = coronavirus disease 2019; D = day; eDiary = electronic diary; FDA = Food and Drug Administration; IRB = institutional review board; M = month; MAAE = medically attended AE; NP= nasopharyngeal swab; RT-PCR = reverse transcriptase polymerase chain reaction; SAE = serious adverse event; SARS-CoV-2 = severe acute respiratory syndrome coronavirus 2; SC = safety (phone) call W = week.

Note: In accordance with FDA Guidance on Conduct of Clinical Trials of Medical Products during the COVID-19 Pandemic (DHHS 2020a), investigators may convert study site visits to telemedicine visits with the approval of the Sponsor.

1. All scheduled study visits should be completed within the respective visit windows. If the participant is not able to come on-site for a study site visit as a result of the COVID-19 pandemic (self-quarantine or disruption of study site activities following business continuity plans and/or local government mandates for "stay at home" or "shelter in place"), a safety phone call to the participant should be made in place of the study site visit. The safety phone call should encompass all scheduled visit assessments that can be completed remotely, such as assessment for AEs and concomitant medications (eg, as defined in scheduled safety phone calls). Home visits will be permitted for all non-dosing visits except for Screening if a participant cannot come to the study site as a result of the COVID-19 pandemic. Home visits must be permitted by the site IRB and the participant via informed consent and have prior approval from the Sponsor (or its designee).
2. Symptom-directed physical examinations may be performed at the discretion of the investigator.
3. Participants with symptoms of COVID-19 will be asked to return within 72 hours or as soon as possible to the study site for an unscheduled visit, to include an NP swab sample (for RT-PCR testing) and other clinical evaluations. If a study site visit is not possible, a home visit may be arranged to collect the NP swab sample and conduct clinical evaluations. If a home visit is not possible, the participant will be asked to submit a saliva sample to the study site by a Sponsor-approved method. At this visit, an NP swab and blood sample will be collected to evaluate for the presence of SARS-CoV-2 infection. An additional NP swab sample will be collected to test for the presence of other respiratory pathogens. In addition, the study site may collect an additional (local/non-central) respiratory sample for SARS-CoV-2 testing to be able to render appropriate medical care for the study participant as determined by local standards of care. Additionally, clinical information will be carefully collected to evaluate the severity of the clinical case.
4. Trained study personnel will call all participants to collect information relating to any MAAEs, AESIs, AEs leading to withdrawal, SAEs, information on concomitant medications associated with those events, and any non-study vaccinations.
5. All concomitant medications relevant to or for the treatment of an SAE or MAAE will be recorded from Screening through the final visit (Day 759).

Table 19: Schedule of Events (Convalescent Period, Starting With the Illness Visit)

Unscheduled Visit	1								2
Type of Visit	C/H								C/H
Daily Timepoint	D1	D2- D6	D7	D8-D13	D14	D15-D20	D21	D22-D27	D28
**Window Allowance (Days)**	-	±1	±1	±1	±1	±1	±1	±1	+7
**Safety Assessments**									
Symptom-directed physical examination[1]	X								X
Follow-up safety[2]	X								X
Recording of MAAEs, AESIs (Part C), AE leading to withdrawal and concomitant medications relevant to or for the treatment of the MAAE[3]	X								X
Recording of SAEs and concomitant medications relevant to or for the treatment of the SAE[3]	X								X
Recording of concomitant medications and non-study vaccinations[3]	X								X
**Efficacy Assessments**									
Daily telemedicine visit[4]		-------------------------------------Daily--------------------------							
Respiratory illness sample[5]	X								
Blood sample for immunologic assessment of SARS-CoV-2 infection[6]	X								X
Saliva sample		D3[7], D5[7]	D7[7]	D9[7]	D14[7]		D21[7]		X[8]

Abbreviations: AE = adverse event; AESI = adverse event of special interest; C = clinic visit; CLIA = Clinical Laboratory Improvement Amendments; COVID-19 = coronavirus disease 2019; D = day; FDA = Food and Drug Administration; H = home visit; MAAE = medically attended AE; NP= nasopharyngeal swab; RT-PCR = reverse transcriptase polymerase chain reaction; SAE = serious adverse event; SARS-CoV-2 = severe acute respiratory syndrome coronavirus 2.
Note: In accordance with FDA Guidance on Conduct of Clinical Trials of Medical Products during the COVID-19 Pandemic (DHHS 2020a), investigators may convert study site visits to telemedicine visits with the approval of the Sponsor.

1. Physical examination: a symptom-directed physical examination, including vital signs will be performed at the initial visit to confirm the diagnosis (denoted as D1 in this table) and at the Convalescent Visit (28 days after diagnosis [D28 in this table]).
2. Trained study personnel will call all participants to collect information relating to any AEs, MAAEs, AEs leading to withdrawal, SAEs, information on concomitant medications associated with those events, and any non-study vaccinations. All safety events will be followed until resolution.
3. All concomitant medications relevant to or for the treatment of an SAE or MAAE will be recorded from Screening through the final visit (Day 759).
4. Participants will have daily telemedicine visits (via video or phone) for 14 days (from the point of the Illness Visit or initial COVID-19 contact) or until symptoms resolve, whichever is later, and which may include symptoms persisting longer than the 28-day Convalescent Period, with the exception of mild loss of sense of taste/smell). If a participant is completely asymptomatic for 72 hours prior to Day 14, including normal oxygen saturation and temperature, then telemedicine calls can be reduced to weekly; however, the participant should still report the daily symptoms at that contact. The participant should also report their oxygen saturation and temperature measured on the day of the contact, but these need not be recorded daily during this reduced frequency monitoring period. If a participant reports a change or recurrence of symptoms, daily telemedicine visits should be resumed to the daily schedule. For these participants, a final telemedicine visit will occur on Day 14 to determine before symptom monitoring can be discontinued. If symptoms persist after Day 60, then telemedicine calls can be reduced to weekly; however, the participant should still report the daily symptoms at that contact. Telemedicine visits may be performed by medically qualified staff appropriately delegated by the investigator. During the telemedicine visit (preferably done in the evening) the participant will be asked to verbally report the severity of each symptom and their highest body temperature and lowest oxygen saturation for that day, and the investigator will determine if medical attention is required due to worsening of COVID-19. The participant will also be reminded to collect a saliva sample and return it to the study site, on the appropriate days. Please note, the negative (-1 day) window allowance does not apply to these daily telemedicine visits and if the +1 day window is used, the site should still collect symptoms from the participant for the previous day.
5. Participants with symptoms of COVID-19 will be asked to return within 72 hours or as soon as possible to the study site or medically qualified staff from the site will conduct a home visit as soon as possible to collect an NP swab sample (for RT-PCR) and collect a blood sample for immunologic assessment of SARS-CoV-2 infection and evaluate for COVID-19. An additional NP swab sample will also be tested for the presence of other respiratory pathogens. In addition, the study site may collect an additional (local/non-central) respiratory sample for SARS-CoV-2 testing to be able to render appropriate medical care for the study participant as determined by local standards of care. Additionally, clinical information will be carefully collected to evaluate the severity of the clinical case. If the RT-PCR test from the NP swab sample from the Illness Visit is negative for SARS-CoV-2, the participant will exit the Convalescent Period and resume the study schedule (Table 16, Table 17, and Table 18).
6. This can be a home visit if necessary.
7. Participants will collect their own saliva sample using the saliva collection tubes provided on 3, 5, 7, 9, 14, and 21 days after the initial Illness Visit, and return them to the study site according to Sponsor instructions. Participants who are pending a central laboratory RT-PCR may exit from the Convalescent Period based on a negative RT-PCR result from a CLIA-certified or CLIA-certified waiver local laboratory at the investigator’s discretion.
8. At this visit, a saliva sample will be collected.

Table 20: Part B: Participant Decision Visit

	All Participants
Sign revised informed consent form	X
Confirm participant's request to be unblinded or not to be unblinded[1]	X
Nasopharyngeal swab	X
Blood for immunologic analysis	X
Counselling the importance of public health measures[2]	X

		Open-label Cohort			
**Participant Status after PDV**	**Blinded Cohort**	**Previously Receiving mRNA-1273**	**Remaining on Placebo**	**Placebo Requesting mRNA-1273**	**mRNA-1273 who Received 1 Dose ONLY**
Continue with original schedules of events, as applicable (Table 16, Table 17, Table 18, and Table 19)	X	X	X	X	X
Supplemental Schedule of Events: Open-Label Days 1-57 (Table 21)[c]	--	--	--	X	--
Modified Supplemental Schedule of Events: Open-Label Days 1-57 (Table 22)[3]	--	--	--	--	X

Abbreviation: PDV = participant decision visit.

1. At the point when the mRNA-1273 vaccine is no longer available for study use, any participant who schedules a PDV after this timepoint will be unblinded (with no option to stay blinded), but will not be offered mRNA-1273 study vaccine.
2. All participants are counselled about the importance of continuing other public health measures to limit the spread of disease including physical-social distancing, wearing a mask, and hand-washing.
3. The Supplemental and the Modified Supplemental Schedule of Events are intended to occur in addition to the original schedules of events, as applicable (Table 16, Table 17, Table 18, and Table 19) and therefore there is a possibility for study visits to overlap. If visits overlap according to respective visit windows, a single visit may be done with the combined study procedures completed once.

Table 21: Supplemental Schedule of Events: Open-Label Observational Phase - Placebo Participants who Request to Receive mRNA-1273

NOTE: Supplemental Schedule of Events: Open-Label Observational Phase is to be used ONLY for participants who received placebo during the Blinded Phase of this study (Part A), and request to receive mRNA-1273. These participants will comply with this Supplemental SoE (Table 21) in addition to the original SoEs as applicable (Table 16, Table 17, Table 18, and Table 19). As this Supplemental SoE is intended to occur in addition to the original SoE, there is a possibility for study visits to overlap. If visits overlap according to respective visit windows, a single visit may be done with the combined study procedures completed once.

**Visit Number**	0	OL-1		OL-2		OL-3
**Type of Visit**	C	C	SC	C	SC	C
**Study Visit Day**	PDV[1] and OL-D1[1]		7 days after OL-D1 (OL-D8)	OL-D29	7 days after OL-D29 (OL-D36)	OL-D57
**Window Allowance (Days)**	-		+3	-3/+14	+3	-3/+14
**Days Since Most Recent Vaccination**	0		7	28/0	7	28
Confirm signing of ICF, concomitant medications, medical history	X					
Confirm participant meets inclusion and exclusion criteria	X					
Physical examination[2]	X			X		X
Pregnancy testing[3]	X			X		
**Immunogenicity Assessment**						
Blood for immunologic analysis[5]	X					X
**Dosing**						
Study injection (including 30-minute post-dosing observation period[6])	X			X		
**Efficacy Assessment**						
Surveillance for COVID-19/Unscheduled Visit[4]	X		X	X	X	X
Nasopharyngeal swab[5]	X					

NOTE: Supplemental Schedule of Events: Open-Label Observational Phase is to be used ONLY for participants who received placebo during the Blinded Phase of this study (Part A), and request to receive mRNA-1273. These participants will comply with this Supplemental SoE (Table 21) in addition to the original SoEs as applicable (Table 16, Table 17, Table 18, and Table 19). As this Supplemental SoE is intended to occur in addition to the original SoE, there is a possibility for study visits to overlap. If visits overlap according to respective visit windows, a single visit may be done with the combined study procedures completed once.

**Visit Number**	0	OL-1		OL-2		OL-3
**Type of Visit**	C	C	SC	C	SC	C
**Study Visit Day**	PDV[1] and OL-D1[1]		7 days after OL-D1 (OL-D8)	OL-D29	7 days after OL-D29 (OL-D36)	OL-D57
**Window Allowance (Days)**	-		+3	-3/+14	+3	-3/+14
**Days Since Most Recent Vaccination**	0		7	28/0	7	28
**Safety Assessments**						
Follow-up safety[7]			X		X	
Recording of MAAEs, AE leading to withdrawal, and concomitant medications relevant to or for the treatment of the MAAE[8]	X		X	X	X	X
Recording of SAEs and concomitant medications relevant to or for the treatment of the SAE[8]	X		X	X	X	X
Recording of concomitant medications and non-study vaccinations[8]	X		X	X	X	X

Abbreviations: AE = adverse event; AR = adverse reaction; C = clinic visit; COVID-19 = coronavirus disease 2019; D = day; FDA = Food and Drug Administration; ICF = informed consent form; MAAE = medically attended AE; NP = nasopharyngeal; OL = open-label; PDV = participant decision visit; RT-PCR = reverse transcriptase polymerase chain reaction; SAE = serious adverse event; SARS-CoV-2 = severe acute respiratory syndrome coronavirus 2; SC = safety (phone) call; SoE = Schedule of Events.

Note: In accordance with FDA Guidance on Conduct of Clinical Trials of Medical Products during the COVID-19 Pandemic (DHHS 2020a), investigators may convert study site visits to telemedicine visits with the approval of the Sponsor. All scheduled study visits should be completed within the respective visit windows. If the participant is not able to come on-site for a study site visit as a result of the COVID-19 pandemic (self-quarantine or disruption of study site activities following business continuity plans and/or local government mandates for “stay at home” or “shelter in place”), a safety call to the participant should be made in place of the study site visit. The safety call should encompass all scheduled visit assessments that can be completed remotely, such as assessment for AEs and

concomitant medications (eg, as defined in scheduled biweekly safety phone calls). Home visits will be permitted for all non-dosing visits except for Screening if a participant cannot come to the study site as a result of the COVID-19 pandemic.

4. The Day 0 and OL-D1 visit may be performed on 2 separate visits.
5. Symptom-directed physical examination will be performed at the PDV and on OL-D29. On each dosing day before injection, the arm receiving the injection should be examined and the associated lymph nodes should be evaluated. Any clinically significant finding identified during a study visit should be reported as an MAAE. Vital signs are to be collected pre- and post-dosing on days of injection (OL-D1 and OL-D29). Participants who are febrile (body temperature ≥ 38.0°C/100.4°F) before dosing (OL-D1 and OL-D29) must be rescheduled within the relevant window period to receive the injection. Afebrile participants with minor illnesses can be enrolled at the discretion of the investigator.
6. Pregnancy test at the PDV and OL-D29 will be a point-of-care urine test. At the discretion of the investigator, a pregnancy test either via blood or point-of-care urine test can be performed. Follicle-stimulating hormone level may be measured to confirm menopausal status at the discretion of the investigator.
7. Participants with symptoms of COVID-19 will be asked to return within 72 hours or as soon as possible to the study site for an unscheduled visit, to include an NP swab sample (for RT-PCR testing) and other clinical evaluations. If a study site visit is not possible, a home visit may be arranged to collect the NP sample and conduct clinical evaluations. If a home visit is not possible, the participant will be asked to submit a saliva sample to the study site by a Sponsor-approved method. At this visit, an NP swab and blood sample will be collected to evaluate for the presence of SARS-CoV-2 infection. An additional NP swab sample will be collected to test for the presence of other respiratory pathogens. In addition, the study site may collect an additional (local/non-central) respiratory sample for SARS-CoV-2 testing to be able to render appropriate medical care for the study participant as determined by local standards of care. Additionally, clinical information will be carefully collected to evaluate the severity of the clinical case. It is important to note that some of the symptoms of COVID-19 overlap with solicited systemic ARs that are expected after vaccination with mRNA-1273 (eg, myalgia, headache, fever, and chills). During the first 7 days after vaccination, when these solicited ARs are common, investigators should use their clinical judgement to decide if an NP swab should be collected. The collection of an NP swab prior to the PDV can help ensure that cases of COVID-19 are not overlooked. Any study participant reporting respiratory symptoms during the 7-day period after vaccination should be evaluated for COVID-19.
8. Sample must be collected prior to unblinding and injection at the PDV.
9. Post-dosing, participants will have a 30-minute observation period.
10. Trained study personnel will call all participants to collect information relating to any MAAEs (including any signs and symptoms of COVID-19), AESIs, AEs leading to withdrawal, SAEs, information on concomitant medications associated with those events, and any non-study vaccinations.
11. All concomitant medications relevant to or for the treatment of an SAE or MAAE will be recorded from Screening through the final visit (Day 759).

Table 22: Modified Supplemental Schedule of Events: Open-Label Observational Phase – mRNA-1273 Participants Who ONLY Received 1 Dose of mRNA-1273

NOTE: Modified Supplemental Schedule of Events: Open-Label Observational Phase is to be used ONLY for participants who ONLY received 1 dose of mRNA-1273 during the Blinded Phase of this study (Part A) and consented to receive mRNA-1273 (see Section 4.1.2 for details). These participants will comply with this SoE (Table 22) in addition to the original SoEs as applicable (Table 16, Table 17, Table 18, and Table 19). As this Modified Supplemental SoE is intended to occur in addition to the original SoE, there is a possibility for study visits to overlap. If visits overlap according to respective visit windows, a single visit may be done with the combined study procedures completed once.

**Visit Number**	0	OL-1		OL-2
**Type of Visit**	C	C	SC	C
**Study Visit Day**	PDV[1] and OL-D1[1]		7 days after OL-D1 OL-D8	OL-D29
**Window Allowance (Days)**	-		+3	-3/+14
**Days Since Most Recent Vaccination**	0		7	28
Confirm signing of ICF, concomitant medications, medical history	X			
Confirm participant meets inclusion and exclusion criteria	X			
Physical examination[2]	X			X
Pregnancy testing[3]	X			
**Immunogenicity Assessment**				
Blood for immunologic analysis[4]	X			X
**Dosing**				
Study injection (including 30-minute post-dosing observation period[5])	X			

NOTE: Modified Supplemental Schedule of Events: Open-Label Observational Phase is to be used ONLY for participants who ONLY received 1 dose of mRNA-1273 during the Blinded Phase of this study (Part A) and consented to receive mRNA-1273 (see Section 4.1.2 for details). These participants will comply with this SoE (Table 22) in addition to the original SoEs as applicable (Table 16, Table 17, Table 18, and Table 19). As this Modified Supplemental SoE is intended to occur in addition to the original SoE, there is a possibility for study visits to overlap. If visits overlap according to respective visit windows, a single visit may be done with the combined study procedures completed once.

**Visit Number**	0	OL-1		OL-2
**Type of Visit**	C	C	SC	C
**Study Visit Day**	PDV[1] and OL-D1[1]		7 days after OL-D1 OL-D8	OL-D29
**Window Allowance (Days)**	-		+3	-3/+14
**Days Since Most Recent Vaccination**	0		7	28
**Efficacy Assessment**				
Surveillance for COVID-19/Unscheduled Visit[6]	X		X	X
Nasopharyngeal swab[5]	X			
**Safety Assessments**				
Follow-up safety[7]			X	
Recording of MAAEs, AE leading to withdrawal, and concomitant medications relevant to or for the treatment of the MAAE[8]	X		X	X
Recording of SAEs and concomitant medications relevant to or for the treatment of the SAE[8]	X		X	X
Recording of concomitant medications and non-study vaccinations[8]	X		X	X

Abbreviations: AE = adverse event; AR = adverse reaction; C = clinic visit; COVID-19 = coronavirus disease 2019; D = day; FDA = Food and Drug Administration; ICF = informed consent form; MAAE = medically attended AE; NP = nasopharyngeal; OL = open-label; PDV = participant decision visit; RT-PCR = reverse transcriptase polymerase chain reaction; SAE = serious adverse event; SARS-CoV-2 = severe acute respiratory syndrome coronavirus 2; SC = safety (phone) call; SoE = Schedule of Events.

Note: In accordance with FDA Guidance on Conduct of Clinical Trials of Medical Products during the COVID-19 Pandemic (DHHS 2020a), investigators may convert study site visits to telemedicine visits with the approval of the Sponsor. All scheduled study visits should be completed within the respective visit windows. If the participant is not able to come on-site for a study site visit as a result of the COVID-19 pandemic (self-quarantine or disruption of study site activities following business continuity plans and/or local government mandates for “stay at home” or “shelter in place”), a safety call to the participant should be made in place of the study site visit. The safety call should encompass all scheduled visit assessments that can be completed remotely, such as assessment for AEs and concomitant medications (eg, as defined in scheduled biweekly safety phone calls). Home visits will be permitted for all non-dosing visits except for Screening if a participant cannot come to the study site as a result of the COVID-19 pandemic.

1. Day 0 and OL-D1 visit may be performed on 2 separate visits.
2. Symptom-directed physical examination will be performed at the PDV. On dosing day before injection, the arm receiving the injection should be examined and the associated lymph nodes should be evaluated. Any clinically significant finding identified during a study visit should be reported as an MAAE. Vital signs are to be collected pre- and post-dosing on the days of injection (OL-D1). Participants who are febrile (body temperature ≥ 38.0°C/100.4°F) before dosing (OL-D1) must be rescheduled within the relevant window period to receive the injection. Afebrile participants with minor illnesses can be enrolled at the discretion of the investigator.
3. Pregnancy test at the PDV will be a point-of-care urine test. At the discretion of the investigator, a pregnancy test either via blood or point-of-care urine test can be performed. Follicle-stimulating hormone level may be measured to confirm menopausal status at the discretion of the investigator.
4. Sample must be collected prior to unblinding and injection at the PDV.
5. Post-dosing, participants will have a 30-minute observation period.
6. Participants with symptoms of COVID-19 will be asked to return within 72 hours or as soon as possible to the study site for an unscheduled visit, to include an NP swab sample (for RT-PCR testing) and other clinical evaluations. If a study site visit is not possible, a home visit may be arranged to collect the NP sample and conduct clinical evaluations. If a home visit is not possible, the participant will be asked to submit a saliva sample to the study site by a Sponsor-approved method. At this visit, an NP swab and blood sample will be collected to evaluate for the presence of SARS-CoV-2 infection. An additional NP swab sample will be collected to test for the presence of other respiratory pathogens. In addition, the study site may collect an additional (local/non-central) respiratory sample for SARS-CoV-2 testing to be able to render appropriate medical care for the study participant as determined by local standards of care. Additionally, clinical information will be carefully collected to evaluate the severity of the clinical case. It is important to note that some of the symptoms of COVID-19 overlap with solicited systemic ARs that are expected after vaccination with mRNA-1273 (eg, myalgia, headache, fever, and chills). During the first 7 days after vaccination, when these solicited ARs are common, investigators should use their clinical judgement to decide if an NP swab should be collected. The collection of an NP swab prior to the PDV can help ensure that cases of COVID-19 are not overlooked. Any study participant reporting respiratory symptoms during the 7-day period after vaccination should be evaluated for COVID-19.
7. Trained study personnel will call all participants to collect information relating to any MAAEs (including any signs and symptoms of COVID-19), AEs leading to withdrawal, SAEs, information on concomitant medications associated with those events, and any non-study vaccinations.
8. All concomitant medications relevant to or for the treatment of an SAE or MAAE will be recorded from Screening through the final visit (Day 759).

Table 23: Part C Supplemental Schedule of Events: Booster Dose Phase

**NOTE: The Part C Supplemental Schedule of Events is to be used for all participants currently enrolled in Part B and who are eligible for Part C. As this Supplemental SoE is intended to occur in addition to and in parallel with the original SoEs (Table 16, Table 17, Table 18, and Table 19), there is a possibility for study visits to overlap. If visits overlap according to respective visit windows, a single visit may be done with the combined study procedures completed once.**					
**Visit Number**	BD-1	BD-1a		BD-2	BD-3
**Type of Visit**	C	C	SC[10]	C	C
**Study Visit Day**	BD-D1[1]	BD-D4	7, 14, and 21 days after BD-D1: (BD-D8, BD-D15, BD-D22)	BD-D29	BD-D181
**Window Allowance (Days)**	-	-2	+3	-3/+14	±14
**Days Since Most Recent Vaccination (in Part C, if applicable)**	0	3	7, 14, 21	28	180
Confirm signing of ICF, concomitant medications, medical history	X				
Confirm participant meets inclusion and exclusion criteria	X				
Physical examination[2]	X			X	X
Pregnancy testing[3]	X				
**Immunogenicity Assessment**					
Blood for immunologic analysis	X[4]			X	X
**Biomarker Assessment**					
Blood sample for potential biomarker analysis[5]		X			
**Dosing**					
Study injection (including 30-minute post-dosing observation period[6])	X				
**Efficacy Assessment**					
Surveillance for COVID-19/Unscheduled Visit[7]	X		X	X	X

**NOTE: The Part C Supplemental Schedule of Events is to be used for all participants currently enrolled in Part B and who are eligible for Part C. As this Supplemental SoE is intended to occur in addition to and in parallel with the original SoEs (Table 16, Table 17, Table 18, and Table 19), there is a possibility for study visits to overlap. If visits overlap according to respective visit windows, a single visit may be done with the combined study procedures completed once.**					
**Visit Number**	BD-1	BD-1a		BD-2	BD-3
**Type of Visit**	C	C	SC[10]	C	C
**Study Visit Day**	BD-D1[1]	BD-D4	7, 14, and 21 days after BD-D1: (BD-D8, BD-D15, BD-D22)	BD-D29	BD-D181
**Window Allowance (Days)**	-	-2	+3	-3/+14	±14
**Days Since Most Recent Vaccination (in Part C, if applicable)**	0	3	7, 14, 21	28	180
Nasopharyngeal swab[8]	X				
**Safety Assessments**					
Follow-up safety[9,10]			X		
Recording of unsolicited AEs[10]	X	X	X	X	
Recording of MAAEs, AESIs, AE leading to withdrawal and concomitant medications relevant to or for the treatment of the MAAE[11]	X	X	X	X	X
Recording of SAEs and concomitant medications relevant to or for the treatment of the SAE[11]	X	X	X	X	X
Recording of concomitant medications and non-study vaccinations[11]	X	X	X	X	X

Abbreviations: AE = adverse event; AESI = adverse event of special interest; BD = booster dose; C = clinic visit; COVID-19 = coronavirus disease 2019; D = day; FDA = Food and Drug Administration; ICF = informed consent form; MAAE = medically attended AE; NP= nasopharyngeal swab; RT-PCR = reverse transcriptase polymerase chain reaction; SAE = serious adverse event; SARS-CoV-2 = severe acute respiratory syndrome coronavirus 2; SC = safety (phone) call; SoE = Schedule of Events.

Note: In accordance with FDA Guidance on Conduct of Clinical Trials of Medical Products during the COVID-19 Pandemic (DHHS 2020a), investigators may convert study site visits to telemedicine visits with the approval of the Sponsor. All scheduled study visits should be completed within the respective visit windows. If the participant is not able to come on-site for a study site visit as a result of the COVID-19 pandemic (self-quarantine or disruption of study site activities following business continuity plans and/or local government mandates for “stay at home” or “shelter in place”), a safety call to the participant should be made in place of the study site visit. The safety call should encompass all scheduled visit assessments that can be completed remotely, such as assessment for AEs and concomitant medications (eg, as defined in scheduled biweekly safety phone calls). Home visits will be permitted for all non-dosing visits except for Screening if a participant cannot come to the study site as a result of the COVID-19 pandemic.

1. A BD may be administered to all participants who are currently enrolled in Part B and have received at least 1 dose of mRNA-1273, provided there are no current contraindications for further dosing (Section 7).
2. For participants who receive a BD, symptom-directed physical examination will be performed at the BD-1 visit. On dosing day before injection, the arm receiving the injection should be examined and the associated lymph nodes should be evaluated. At visits BD-2 (BD-Day 29) and BD-3 (BD-Day 181), a symptom-directed physical examination may be performed at the discretion of the investigator. Any clinically significant finding identified during a study visit should be reported as an MAAE. For participants who receive a BD, vital signs are to be collected pre- and post-dosing (participant will be seated for at least 5 minutes before all measurements are taken per Section 8.2.5) on the day of injection (BD-Day 1). Participants who are febrile (body temperature ≥ 38.0°C/100.4°F) before dosing (BD-Day 1) must be rescheduled to receive the injection. Afebrile participants with minor illnesses can be vaccinated at the discretion of the investigator. Physical examination and vital sign collection are optional for participants who chose not to receive a BD.
3. For participants who receive a BD, the pregnancy test at the BD-1 visit will be a point-of-care urine test. At the discretion of the investigator, a pregnancy test either via blood or point-of-care urine test can be performed. Follicle-stimulating hormone level may be measured to confirm menopausal status at the discretion of the investigator
4. Sample must be collected prior to injection (if receiving a booster) at the BD-1 visit.
5. Only for participants who chose to receive a BD. Serum sample from two ~4 mL optional blood draws, subject to availability of blood draw kits, and will be confirmed with participant at time of re-consenting for Part C. Biomarker plasma and biomarker serum samples will be stored for potential future biomarker assessment.
6. Post-dosing, participants will have a 30-minute observation period.
7. Participants with symptoms of COVID-19 will be asked to return within 72 hours or as soon as possible to the study site for an unscheduled visit, to collect an NP swab sample (for RT-PCR testing) and other clinical evaluations. If a study site visit is not possible, a home visit may be arranged to collect the NP sample and conduct clinical evaluations. If a home visit is not possible, the participant will be asked to submit a saliva sample to the study site by a Sponsor-approved method. At this visit, an NP swab and blood sample will be collected to evaluate for the presence of SARS-CoV-2 infection. An additional NP swab sample will be collected to test for the presence of other respiratory pathogens. In addition, the study site may collect an additional respiratory sample for SARS-CoV-2 testing to be able to render appropriate medical care for the study participant as determined by local

standards of care. Additionally, clinical information will be carefully collected to evaluate the severity of the clinical case. Any study participant reporting respiratory symptoms during the 7-day period after vaccination should be evaluated for COVID-19.

8. The NP swab sample is strongly encouraged but ultimately optional for the participants. If collected, it must be collected prior to injection at the BD-1 visit.

9. Trained study personnel will call all participants to collect information relating to any unsolicited AEs, MAAEs (including any signs and symptoms of COVID-19), AESIs, AEs leading to withdrawal, SAEs, information on concomitant medications associated with those events, and any non-study vaccinations.

10. Only for participants who chose to receive a BD.

11. All concomitant medications relevant to or for the treatment of an SAE or MAAE will be recorded from Screening through the final visit (Day 759).

11.2. APPENDIX 2: Study Governance Considerations

11.2.1. Regulatory and Ethical Considerations

This study will be conducted in accordance with the protocol and with the following:

- Consensus ethical principles derived from international guidelines including the Declaration of Helsinki and Council for International Organizations of Medical Sciences International Ethical Guidelines.
- Applicable ICH GCP Guidelines.
- Applicable laws and regulatory requirements.
- The protocol, protocol amendments, ICF, Investigator Brochure, and other relevant documents (eg, advertisements) must be submitted to an IRB/IEC by the investigator and reviewed and approved by the IRB/IEC before the study is initiated.
- Any amendments to the protocol will require IRB/IEC approval before implementation of changes made to the study design, except for changes necessary to eliminate an immediate hazard to study participants.
- The investigator will be responsible for the following:

– Providing written summaries of the status of the study to the IRB/IEC annually or more frequently in accordance with the requirements, policies, and procedures established by the IRB/IEC

– Notifying the IRB/IEC of SAEs or other significant safety findings as required by IRB/IEC procedures

– Providing oversight of the conduct of the study at the site and adherence to requirements of 21 CFR, ICH guidelines, the IRB/IEC, European regulation 536/2014 for clinical studies (if applicable), and all other applicable local regulations.

11.2.2. Study Monitoring

Before an investigational site can enter a participant into the study, a representative of Moderna or its representatives will visit the investigational study site to:

- Determine the adequacy of the facilities.
- Discuss with the investigator(s) and other personnel their responsibilities with regard to protocol adherence, and the responsibilities of Moderna or its representatives. This will be documented in a Clinical Study Agreement between Moderna, designated CRO, and the investigator.

According to ICH GCP guideline, the Sponsor of the study is responsible for ensuring the proper conduct of the study with regard to protocol adherence and validity of data recorded on the eCRFs. The study monitor's duties are to aid the investigator and Moderna in the maintenance of complete, accurate, legible, well-organized, and easily retrievable data. The study monitor will advise the investigator of the regulatory necessity for study-related monitoring, audits, IRB/IEC review, and inspection by providing direct access to the source data/documents. In addition, the study monitor will explain to and interpret for the investigator all regulations applicable to the clinical evaluation of an IP as documented in ICH guidelines.

It is the study monitor's responsibility to inspect the eCRFs and source documentation throughout the study to protect the rights of the participants; to verify adherence to the protocol; to verify completeness, accuracy, and consistency of the data; and to confirm adherence of study conduct to any local regulations. Details will be outlined in the Clinical Monitoring Plan. During the study, a monitor from Moderna or a representative will have regular contacts with the investigational site, for the following:

- Provide information and support to the investigator(s).
- Confirm that facilities remain acceptable.
- Confirm that the investigational team is adhering to the protocol, that the data are being accurately recorded in the eCRFs, and that IP accountability checks are being performed.
- Perform source data verification. This includes a comparison of the data in the eCRFs with the participant's medical records at the hospital or practice, and other records relevant to the study. This will require direct access to all original records for each participant (eg, clinical charts or electronic medical record system).
- Record and report any protocol deviations not previously sent.
- Confirm AEs and SAEs have been properly documented on eCRFs and confirm any SAEs have been forwarded to the SAE Hotline, and those SAEs that met criteria for reporting have been forwarded to the IRB/IEC.

The monitor will be available between visits if the investigator(s) or other staff needs information or advice.

In accordance with FDA Guidance on Conduct of Clinical Trials of Medical Products during the COVID-19 Pandemic (DHHS 2020a), investigators may convert study site visits to telemedicine visits with the approval of the Sponsor (Section 8 for Procedures).

The DSMB will also have responsibility for safety monitoring (Section 8.4.2).

11.2.3. Audits and Inspections

Moderna, their designee(s), the IRB/IEC, or regulatory authorities will be allowed to conduct site visits to the investigational facilities for the purpose of monitoring or inspecting any aspect of the study. The investigator agrees to allow Moderna, their designee(s), the IRB/IEC, or regulatory authorities to inspect the IP storage area, IP stocks, IP records, participant charts and study source documents, and other records relative to study conduct.

Authorized representatives of Moderna, a regulatory authority, and any IRB/IEC may visit the site to perform audits or inspections, including source data verification. The purpose of a Sponsor audit or inspection is to systematically and independently examine all study-related activities and documents to determine whether these activities were conducted, and data were recorded, analyzed, and accurately reported according to the protocol, ICH GCP E6R2, and any applicable regulatory requirements. The investigator should contact Moderna immediately if contacted by a regulatory agency about an inspection.

The Principal Investigator must obtain IRB approval for the investigation. Initial IRB approval, and all materials approved by the IRB for this study including the participant consent form and recruitment materials must be maintained by the investigator and made available for inspection.

11.2.4. Financial Disclosure

The investigator is required to provide financial disclosure information to allow the Sponsor to submit the complete and accurate certification or disclosure statements required under 21 CFR 54. In addition, the investigator must provide the Sponsor with a commitment to promptly update this information if any relevant changes occur during the course of the investigation and for 1 year following the completion of the study.

The Sponsor, the CRO, and the study site are not financially responsible for further testing or treatment of any medical condition that may be detected during the screening process. In addition, in the absence of specific arrangements, the Sponsor, the CRO, and the study site are not financially responsible for further treatment of the disease under study.

11.2.5. Recruitment Procedures

Advertisements to be used for the recruitment of study participants, and any other written information regarding this study to be provided to the participant should be submitted to the Sponsor for approval. All documents must be approved by the IRB.

11.2.6. Informed Consent Process

The informed consent document(s) must meet the requirements of 21 CFR 50, local regulations, ICH guidelines, Health Insurance Portability and Accountability Act requirements, where

applicable, and the IRB/IEC or study center. All consent documents will be approved by the appropriate IRB/IEC. The actual ICF used at each center may differ, depending on local regulations and IEC/IRB requirements. However, all versions must contain the standard information found in the sample ICF provided by the Sponsor. Any change to the content of the ICF must be approved by the Sponsor and the IEC / IRB prior to the form being used.

If new information becomes available that may be relevant to the participant's willingness to continue participation in the study, this will be communicated to him/her in a timely manner. Such information will be provided via a revised ICF or an addendum to the original ICF.

The investigator or his/her representative will explain the nature of the study to the participant and answer all questions regarding the study.

The investigator is responsible for ensuring that the participant fully understands the nature and purpose of the study. Information should be given in both oral and written form whenever possible.

No participant should be obliged to participate in the study. The participant must be informed that participation is voluntary. Participants, their relatives, guardians, or (if applicable) legal representatives must be given ample opportunity to inquire about details of the study. The information must make clear that refusal to participate in the study or withdrawal from the study at any stage is without any prejudice to the participant's subsequent care.

The participant must be allowed sufficient time to decide whether they wish to participate. The participant must be made aware of and give consent to direct access to his/her source medical records by study monitors, auditors, the IRB/IEC, and regulatory authorities. The participant should be informed that such access will not violate participant confidentiality or any applicable regulations. The participant should also be informed that he/she is authorizing such access by signing the ICF.

A copy of the ICF(s) must be provided to the participant.

A participant who is rescreened/rescheduled (including at the BD-1 visit) is not required to re-sign the same version of the ICF if this occurs within 28 days from the previous ICF signature date. The ICF will also explain that excess serum from immunogenicity testing may be used for future research, which may be performed at the discretion of the Sponsor to further characterize the immune response to SARS-CoV-2, additional assay development, and the immune response across CoV.

11.2.7. Protocol Amendments

No change or amendment to this protocol may be made by the investigator or the Sponsor after the protocol has been agreed to and signed by all parties unless such change(s) or amendment(s)

has (have) been agreed upon by the investigator or the Sponsor. Any change agreed upon will be recorded in writing, and the written amendment will be signed by the investigator and the Sponsor. Institutional review board approval is required prior to the implementation of an amendment, unless overriding safety reasons warrant immediate action, in which case the IRB(s)/IEC(s) will be promptly notified.

Any modifications to the protocol or the ICF, which may impact the conduct of the study, potential benefit of the study, or may affect participant safety, including changes of study objectives, study design, participant population, sample sizes, study procedures, or significant administrative aspects will require a formal amendment to the protocol. Such amendment will be released by the Sponsor, agreed by the investigator(s), and approved by the relevant IRB(s)/IEC(s) prior to implementation. A signed and dated statement that the protocol, any subsequent relevant amended documents and the ICF have been approved by relevant IRB(s)/IEC(s) must be provided to the Sponsor before the study is initiated.

Administrative changes of the protocol are minor corrections and/or clarifications that have no effect on the way the study is to be conducted. These administrative changes will be released by the Sponsor, agreed by the investigator(s), and notified to the IRB(s)/IEC(s).

11.2.8. Protocol Deviations

The noncompliance may be either on the part of the participant, the investigator, or the study site staff. As a result of deviations, corrective actions are to be developed by the site and implemented promptly.

It is the responsibility of the site investigator to use continuous vigilance to identify and report deviations to the Sponsor or its designee. All deviations must be addressed in study source documents, reported to study monitor. Protocol deviations must be sent to the reviewing IRB/IEC per their policies. The site investigator is responsible for knowing and adhering to the reviewing IRB/IEC requirements.

11.2.9. Data Protection

Participants will be assigned a unique identifier by the Sponsor. Any participant records or datasets that are transferred to the Sponsor will contain the identifier only; participant names or any information which would make the participant identifiable will not be transferred.

The participant must be informed that his/her personal study-related data will be used by the Sponsor in accordance with local data protection law. The level of disclosure must also be explained to the participant.

The participant must be informed that his/her medical records may be examined by Clinical Quality Assurance auditors or other authorized personnel appointed by the Sponsor, by appropriate IRB/IEC members, and by inspectors from regulatory authorities.

Individual participant medical information obtained as a result of this study is considered confidential, and disclosure to third parties is prohibited. Information will be accessible to authorized parties or personnel only. Medical information may be given to the participant's physician or to other appropriate medical personnel responsible for the participant's well-being. Each participant will be asked to complete a form allowing the investigator to notify the participant's primary health care provider of his/her participation in this study.

All laboratory specimens, evaluation forms, reports, and other records will be identified in a manner designed to maintain participant confidentiality. All records will be kept in a secure storage area with limited access. Clinical information will not be released without the written permission of the participant, except as necessary for monitoring and auditing by the Sponsor, its designee, relevant regulatory authority, or the IRB.

The investigator and all employees and coworkers involved with this study may not disclose or use for any purpose other than performance of the study, any data, record, or other unpublished, confidential information disclosed to those individuals for the purpose of the study. Prior written agreement from the Sponsor or its designee must be obtained for the disclosure of any confidential information to other parties.

11.2.10. Sample Retention and Future Biomedical Research

The retention period of laboratory samples will be 15 years, or as permitted by local regulations, to address further scientific questions related to mRNA-1273 or anti-respiratory virus immune response. In addition, identifiable samples can be destroyed at any time at the request of the participant. During the study, or during the retention period, in addition to the analysis outlined in the study endpoints, exploratory analysis may be conducted, using other Ab-based methodologies, on any remaining blood or serum samples, including participants who provide samples for screening, but are not subsequently enrolled. These analyses would extend the search for other potentially relevant biomarkers to investigate the effect of mRNA-1273, as well as to determine how changes in the markers may relate to exposure and clinical outcomes. A decision to perform such exploratory research may arise from new scientific findings related to the drug class or disease, as well as reagent and assay availability.

11.2.11. Data Quality Assurance and Quality Control

Data collection is the responsibility of the clinical study staff at the site under the supervision of the site investigator. The investigator is responsible for ensuring the accuracy, completeness, legibility, and timeliness of the data reported.

- All participant data relating to the study will be recorded eCRF unless transmitted to the Sponsor or designee electronically (eg, laboratory data). The investigator is responsible for verifying that data entries are accurate and correct by physically or electronically signing the eCRF.
- The investigator must maintain accurate documentation (source data) that supports the information entered in the eCRF.
- The investigator must permit study-related monitoring, audits, IRB/IEC review, and regulatory agency inspections and provide direct access to source data documents.
- Monitoring details describing strategy (eg, risk-based initiatives in operations and quality such as Risk Management and Mitigation Strategies and Analytical Risk-Based Monitoring), methods, responsibilities and requirements, including handling of noncompliance issues and monitoring techniques (central, remote, or on-site monitoring) are provided in the study Monitoring Plan, Centralized Monitoring Plan, and Risk Management Plan.
- The Sponsor or designee is responsible for the data management of this study including quality checking of the data.
- The Sponsor assumes accountability for actions delegated to other individuals (eg, CROs).
- Study monitors will perform ongoing source data verification to confirm that data entered into the eCRF by authorized site personnel are accurate, complete, and verifiable from source documents; that the safety and rights of participants are being protected; and that the study is being conducted in accordance with the currently approved protocol and any other study agreements, ICH GCP, and all applicable regulatory requirements.
- Records and documents, including signed ICFs, pertaining to the conduct of this study must be retained by the investigator for 15 years after study completion unless local regulations or institutional policies require a longer retention period. No records may be destroyed during the retention period without the written approval of the Sponsor. No records may be transferred to another location or party without written notification to the Sponsor.

Quality assurance (QA) includes all the planned and systematic actions that are established to ensure that the clinical study is performed, and the data are generated, documented (recorded), and reported according to ICH GCP and local/regional regulatory standards.

A QA representative from the Sponsor or qualified designee, who is independent of and separated from routine monitoring, may periodically arrange inspections/audits of the clinical study by reviewing the data obtained and procedural aspects. These inspections may include on-site inspections/audits and source data checks. Direct access to source documents is required for the purpose of these periodic inspections/audits.

11.2.12. Data Collection and Management

This study will be conducted in compliance with ICH CGP guidelines. This study will also be conducted in accordance with the most recent version of the Declaration of Helsinki.

This study will use electronic data collection (to collect data directly from the investigational site using eCRFs). The investigator is responsible for ensuring that all sections of each eCRF are completed promptly and correctly and that entries can be verified against any source data.

Study monitors will perform source document verification to identify inconsistencies between the eCRFs and source documents. Discrepancies will be resolved in accordance with the principles of GCP. Detailed study monitoring procedures are provided in the Clinical Monitoring Plan.

AEs will be coded with the most current available version of MedDRA. Concomitant medications will be coded using WHO – Drug Reference List.

11.2.13. Source Documents

Source documents are original documents or certified copies, and include, but are not limited to, diaries, medical and hospital records, screening logs, informed consent/assent forms, telephone contact logs, and worksheets. Source documents provide evidence for the existence of the participant and substantiate the integrity of the data collected. Source documents are filed at the investigator’s site.

Data reported on the CRF or entered in the eCRF that are transcribed from source documents must be consistent with the source documents or the discrepancies must be explained. The investigator may need to request previous medical records or transfer records, depending on the study. Also, current medical records must be available.

Moderna or its designee requires that the investigator prepare and maintain adequate and accurate records for each participant treated with the IP. Source documents such as any hospital, clinic, or office charts and the signed ICFs are to be included in the investigator’s files with the participant’s study records.

11.2.14. Retention of Records

The Principal Investigator must maintain all documentation relating to the study for a period of at least 2 years after the last marketing application approval or, if not approved, 2 years following the discontinuance of the test article for investigation. If this requirement differs from any local regulations, the local regulations will take precedence unless the local retention policy is less than 2 years.

If it becomes necessary for Moderna or the regulatory authority to review any documentation relating to the study, the investigator must permit access to such records. No records will be destroyed without the written consent of the Sponsor, if applicable. It is the responsibility of the Sponsor to inform the investigator when these documents no longer need to be retained.

11.2.15. Study and Site Closure

If the study is prematurely terminated or suspended, the Sponsor shall promptly inform the investigators, the IECs/IRBs, the regulatory authorities, and any CRO(s) used in the study of the reason for termination or suspension, as specified by the applicable regulatory requirements. The investigator shall promptly inform the participant and should assure appropriate participant therapy and/or follow-up.

The Sponsor or designee reserves the right to close the study site or terminate the study at any time for any reason at the sole discretion of the Sponsor.

The investigator may initiate study-site closure at any time, provided there is reasonable cause and sufficient notice is given in advance of the intended termination.

Reasons for the early closure of a study site by the Sponsor or investigator may include but are not limited to:

- Continuation of the study represents a significant medical risk to participants.
- Failure of the investigator to comply with the protocol, the requirements of the IRB/IEC or local health authorities, the Sponsor's procedures, or GCP guidelines.
- Inadequate recruitment of participants by the investigator.
- Discontinuation of further mRNA-1273 development.

Study sites will be closed upon study completion. A study site is considered closed when all required documents and study supplies have been collected and a study-site closure visit has been performed.

11.2.16. Publication Policy

The results of this study may be published or presented at scientific meetings. If this is foreseen, the investigator agrees to submit all manuscripts or abstracts to the Sponsor before submission. This allows the Sponsor to protect proprietary information and to provide comments.

The Sponsor will comply with the requirements for publication of study results. In accordance with standard editorial and ethical practice, the Sponsor will generally support publication of multicenter studies only in their entirety and not as individual site data. In this case, a coordinating investigator will be designated by mutual agreement.

Authorship will be determined by mutual agreement and in line with International Committee of Medical Journal Editors authorship requirements.

The clinical study plan and the results of the study will be published on www.ClinicalTrials.gov in accordance with 21 CFR 50.25(c). The results of and data from this study belong to Moderna.

11.3. APPENDIX 3: Contraceptive Guidance

Woman of Childbearing Potential (WOCBP)

Females of childbearing potential are those who are considered fertile following menarche and until becoming postmenopausal unless permanently sterile (see below). If fertility is unclear (eg, amenorrhea in adolescents or athletes) and a menstrual cycle cannot be confirmed before first dose of study treatment, additional evaluation should be considered.

Women in the following categories are not considered WOCBP:

1. Premenarchal
2. Premenopausal, surgically sterile female with 1 of the following:
   a. Documented complete hysterectomy
   b. Documented bilateral salpingectomy
   c. Documented bilateral oophorectomy

For individuals with permanent infertility due to an alternate medical cause other than the above, (eg, mullerian agenesis, androgen insensitivity), investigator discretion should be applied in determining study entry.

Note: Documentation can come from the site personnel's: review of the participant's medical records, medical examination, or medical history interview.

3. Postmenopausal female

- A postmenopausal state is defined as no menses for 12 months without an alternative medical cause. The following age-specific requirements apply:
  - Women < 50 years of age would be considered postmenopausal if they have been amenorrheic for 12 months or more following cessation of exogenous hormonal treatments and if they have luteinizing hormone and FSH levels in the postmenopausal range for the institution.
  - Women ≥ 50 years of age would be considered postmenopausal if they have been amenorrheic for 12 months or more, had radiation-induced menopause with last menses > 1 year ago or had chemotherapy-induced menopause with last menses > 1 year ago.
- A high FSH level in the postmenopausal range may be used to confirm a postmenopausal state in women not using hormonal replacement therapy (HRT).
- Females on HRT and whose menopausal status is in doubt will be required to use one of the non-estrogen hormonal highly effective contraception methods if they wish to

continue their HRT during the study. Otherwise, they must discontinue HRT to allow confirmation of postmenopausal status before study enrollment.

11.4. APPENDIX 4: Statistical Appendices

11.4.1. Estimands and Estimand Specifications

Table 24: Intercurrent Event Types

Label	Intercurrent Event Type	Comment
IcEv1 (death without confirmation of cases, ie, unrelated death)	Unrelated death without documented confirmed COVID-19	Participants in PP Set who die due to reasons unrelated to COVID-19 without confirmation of being a case will all be included in statistical analysis.
IcEv2 (early COVID-19)	COVID-19 starting up to 14 days after the second dose of IP	Participants in PP Set who experience early COVID-19 up to 14 days after the second dose of IP will all be included in statistical analysis.

Abbreviations: COVID-19 = coronavirus disease 2019; IcEv: intercurrent event; IP = investigational product; PP: per-protocol.

Table 25: Primary Objective and Estimands With Rationale for Strategies to Address Intercurrent Events for Per-protocol Analysis

**Objective: To demonstrate the efficacy of mRNA-1273 to prevent COVID-19**	
**Estimand Description**	Vaccine efficacy will be measured using 1 – HR (mRNA-1273/placebo) of COVID-19 from 14 days after second dose of IP in adults. A hypothetical strategy will be used for deaths unrelated to COVID-19, and a hypothetical strategy will also be used for early COVID-19 in participants in the PP Set.
**Target Population**	Adults aged 18 years and older in circumstances at a high risk of SARS-CoV-2 infection but without medical conditions that pose additional risk of developing severe disease.<br>The population excludes those previously infected or vaccinated for SARS-CoV-2 or with a medical condition, on treatment that poses additional risks (including those requiring immunosuppressants or immune-modifying drugs), or SARS-CoV-2 pre-positive.
**Variable/Endpoint**	Time to COVID-19 disease, censoring at early discontinuation, early COVID-19, or last assessment for an event not being observed, whichever comes earlier.
**Treatment Condition(s)**	**Test:** mRNA-1273<br>**Reference:** Placebo
**Estimand Label**	**Estimand 1**
**Population-Level Summary**	Vaccine efficacy defined as 1 - HR of mRNA-1273/placebo
**Intercurrent Event Strategy**	
**IcEv1 (Unrelated death):**	Hypothetical
**IcEv2 (early infection):**	Hypothetical
**Rationale for Strategy(s)**	Hypothetical: unrelated death without confirmation of COVID-19 will be censored at the time of death as if there is no event, handled with independent censoring.<br>Hypothetical: early case in PP Set will be censored at the time of case onset if it is not a case, handled with independent censoring.

Abbreviations: COVID-19 = coronavirus disease 2019; HR = hazard ratio; IcEv = intercurrent event; IP = investigational product; PP = per protocol; SARS-CoV-2 = severe acute respiratory syndrome coronavirus 2.

11.4.2. Statistical Methods and Sensitivity Analyses

Table 26: Summary of Statistical Methods and Sensitivity Analyses

Estimand Label	Estimand Description	Main Estimation			Sensitivity Analysis
		Analysis Set	Imputation/Data/Censoring Rules	Analysis Model/Method	
Estimand 1	See Table 25	PP Set	Participants who did not develop COVID-19, ongoing or complete the study will be censored at the last assessment.<br>Participants who discontinue early or die without COVID-19 will be censored at the date of discontinuation or death, respectively.<br>Participant who is a confirmed case prior to 14 days after the second dose of IP will be censored on the date of confirmation.	The (1 - HR) with 95% CI will be estimated using Cox proportional hazard regression analysis with treatment arm as a fixed effect stratified by randomization stratification factors. And the null hypothesis will be tested using Wald Chi-square method.	As described in Section 9.5.1 with cases counted starting after the second dose of IP.

Abbreviations: CI = confidence interval; COVID-19 = coronavirus disease 2019; HR = hazard ratio; IP = investigational product; PP = per protocol.

11.5. APPENDIX 5: Adverse Events of Special Interest Terms

The Investigator's medical judgement must be applied to assess an event as an AESI, as most AESIs are based on medical concepts. Table 27 below does not provide a comprehensive list of terms.

Table 27 describes events/medical concepts that are of interest in COVID-19 vaccine safety surveillance. Some are specific to vaccines; however, some are of interest due to their occurrence in the context of concurrent or recent COVID-19. Events that occur in Part C (after participants receive a booster dose) and fall into the descriptions below should be reported as AESIs, per the reporting processes specified in Section 8.3.3, even when they occur during/following COVID-19 infection.

Please note: COVID-19 itself is not an AESI.

Table 27: Adverse Events of Special Interest

Medical Concept	Medical Concept Descriptions/Guidance
**Anosmia, Ageusia**	• New onset of anosmia or ageusia associated with COVID-19 or idiopathic etiology<br>• DOES NOT INCLUDE anosmia or ageusia associated with sinus/nasal congestion, congenital, or traumatic etiologies
**Subacute thyroiditis**	• Acute inflammatory disease of the thyroid (immune-mediated or idiopathic)<br>• DOES NOT INCLUDE new onset of chronic thyroiditis
**Acute pancreatitis**	• New onset of pancreatitis in the absence of a clear, alternate etiology, such as alcohol, gallstones, trauma, recent invasive procedure, etc.
**Appendicitis**	• Any event of appendicitis
**Rhabdomyolysis**	• New onset of rhabdomyolysis in the absence of a clear, alternate etiology, such as drug/alcohol abuse, excessive exercise, trauma, etc.
**Acute respiratory distress syndrome (ARDS)**	• New onset of ARDS/respiratory failure due to acute inflammatory lung injury<br>• DOES NOT INCLUDE non-specific symptoms of shortness of breath or dyspnea, nor events with underlying etiologies of heart failure or fluid overload
**Coagulation disorders**	• New onset of thrombosis, thromboembolic event, or non-traumatic hemorrhage/bleeding disorder (ex. stroke, DVT, pulmonary embolism, disseminated intravascular coagulation (DIC), etc.)

Medical Concept	Medical Concept Descriptions/Guidance
**Acute cardiovascular injury**	• New onset of clinically confirmed, acute cardiovascular injury, such as myocarditis, pericarditis, arrhythmia confirmed by ECG (ex. atrial fibrillation, atrial flutter, supraventricular tachycardia), stress cardiomyopathy, heart failure, acute coronary syndrome, myocardial infarction, etc.<br>• DOES NOT INCLUDE transient sinus tachycardia/bradycardia, non-specific symptoms such as palpitations, racing heart, heart fluttering or pounding, irregular heartbeats, shortness of breath, chest pain/discomfort, etc.
**Acute kidney injury**	• New onset of acute kidney injury or acute renal failure in the absence of a clear, alternate etiology, such as urinary tract infection/urosepsis, trauma, tumor, nephrotoxic medications/substances, etc.;<br>• Increase in serum creatinine by ≥ 0.3 mg/dl (or ≥26.5 µmol/l) within 48 hours; **OR**<br>• Increase in serum creatinine to ≥ 1.5 times baseline, known or presumed to have occurred within prior 7 days
**Acute liver injury**	• New onset in the absence of a clear, alternate etiology, such as trauma, tumor, hepatotoxic medications/substances, etc.:<br>• >3-fold elevation above the upper normal limit for ALT or AST; **OR**<br>• >2-fold elevation above the upper normal limit for total serum bilirubin or GGT or ALP
**Dermatologic findings**	• Chilblain-like lesions<br>• Single organ cutaneous vasculitis<br>• Erythema multiforme<br>• Bullous rash<br>• Severe cutaneous adverse reactions, such as Stevens-Johnson syndrome, Toxic epidermal necrolysis, Drug reaction with eosinophilia and systemic symptoms (DRESS), fixed drug eruptions, and necrotic or exfoliative reactions
**Systemic inflammatory syndromes**	• Multisystem inflammatory syndrome in adults (MIS-A) or children (MIS-C)<br>• Kawasaki's disease<br>• Hemophagocytic lymphohistiocytosis (HLH)
**Thrombocytopenia**	• Platelet count < 150 x10^9/L (thrombocytopenia)<br>• New clinical diagnosis, or worsening, of thrombocytopenic condition, such as immune thrombocytopenia, thrombocytopenic purpura, or HELLP syndrome

Medical Concept	Medical Concept Descriptions/Guidance
**Acute aseptic arthritis**	Clinical syndrome characterized by acute onset of signs and symptoms of joint inflammation without recent trauma for a period of no longer than 6 weeks, synovial increased leukocyte count and the absence of microorganisms on Gram stain, routine culture and/or PCR.<br>• DOES NOT INCLUDE new onset of chronic arthritic conditions
**New onset, or worsening, of neurological disease**	• Immune-mediated neurological disorders<br>• Guillain-Barre Syndrome<br>• Acute disseminated encephalomyelitis (ADEM)<br>• Peripheral facial nerve palsy (Bell's palsy)<br>• Transverse myelitis<br>• Encephalitis/Encephalomyelitis<br>• Aseptic meningitis<br>• Seizures/convulsions/epilepsy<br>• Narcolepsy/hypersomnia
**Anaphylaxis**	• Anaphylaxis associated with study drug administration
**Other syndromes**	• Fibromyalgia<br>• Postural Orthostatic Tachycardia Syndrome<br>• Chronic Fatigue Syndrome<br>• Myalgic encephalomyelitis<br>• Post viral fatigue syndrome<br>• Myasthenia gravis

Abbreviations: ALP = alkaline phosphatase; ALT = alanine transaminase; AST = aspartate transaminase; COVID-19 = coronavirus disease 2019.

11.6. APPENDIX 6: Protocol Amendment History

The document history table for this protocol and the Protocol Amendment Summary of Changes Table for the current Amendment 10 is located directly before the Table of Contents.

A description of Amendment 9, Amendment 8, Amendment 7, Amendment 6, Amendment 5, Amendment 4, Amendment 3, Amendment 2, and Amendment 1 is presented in this appendix.

Amendment 9, 10 Sep 2021

Main Rationale for the Amendment:

1. To provide a 50 µg BD of mRNA-1273 to participants. This change is prompted by the announcement by the President of the US that, as of 20 Sep 2021, certain individuals will be eligible to receive a BD in addition to the prior receipt of a primary vaccine regimen with mRNA-1273. The interim results of an ongoing Moderna Phase 2 study (mRNA-1273-P201), in which participants who 6 to 8 months prior received 2 doses of

50 µg or 100 µg of mRNA-1273 were administered a 50 µg booster of mRNA-1273, demonstrated enhanced immune responses compared to pre-boost levels and met the noninferiority criteria stipulated in the FDA Guidance on EUA for Vaccines to Prevent COVID-19. Additionally, no new safety signals emerged upon administration of the BD in Study mRNA-1273-P201. Based on cumulative evidence, the benefit-risk profile of a BD of mRNA-1273 is favorable, particularly in light of increasing breakthrough disease with the emergence of the Delta variant. The details of eligibility for BDs provided by the US federal government have not been specified as of the time of writing this protocol amendment. Providing the option for a BD to all federally eligible participants currently enrolled in Part B is expected to promote retention of participants in the ongoing study and thereby defend the scientific integrity of the study for the planned 2-year duration of follow-up after the completion of the primary vaccination series.

2. To provide guidance to the investigators regarding assessing and reporting AESIs, including myocarditis and pericarditis, for this study population.

Summary of Major Changes From Protocol Amendment 8 to Protocol Amendment 9:

Section # and Name	Description of Change	Brief Rationale
Section 1.2 (Schema); Section 4.1 (General Design); Section 4.1.3 (Part C, the Booster Dose Phase); Section 4.3 (Choice of Dose and Control Product); Section 5.1.1 (Inclusion Criteria); Section 6.2.2 (Administration of Investigational Product); Section 6.2.4 (Packaging and Labeling); Section 6.2.5 (Storage); Section 8.1.2 (Surveillance for COVID-19 Symptoms); Section 8.1.5 (Immunogenicity Assessments); Section 8.2 (Safety Assessments); Section 8.2.1 (Safety Phone Calls); Section 8.2.6 (Blood Sampling); Section 8.3.6 (Recording and Follow-up of Pregnancy); Section 8.3.7 (Recording and Follow-up of an AE and/or SAE); Section 9 (Statistical Considerations); Section 9.5.1.3 (Long-term Efficacy Analysis); Section 9.5.2.1 (Adverse Events); Section 11.1. (APPENDIX 1: Schedules of Events; Table 23)	Text updated to reflect initiation of Part C, providing for administration of an mRNA-1273 booster dose.	To promote retention of participants in the ongoing study and thereby defend the scientific integrity of the study for the planned 2-year duration of follow-up after completion of the primary vaccination series.

Section # and Name	Description of Change	Brief Rationale
Section 2.1 (Study Rationale);<br>Section 2.2.2 (Clinical Studies);<br>Section 2.3.2 (Risks from Study Participation)	Updated the status of ongoing clinical research studies.	To provide context for Protocol Amendment 9.
Section 2.3.2 (Risks from Study Participation)	Updated to include no clinical evidence for enhanced vaccine disease from the Phase 3 clinical trial. Also updated to include very rare reports of myocarditis and pericarditis occurring after vaccination with Moderna COVID-19 vaccine.	To provide updated information to the investigators in line with updates made to the IB.
Section 3 (Objectives and Endpoints)	Updated to include evaluation of the safety and immunogenicity of a booster dose of mRNA-1273 as exploratory objectives.	To identify objectives related to the initiation of Part C.
Section 8.1.1 (Efficacy Assessments Related to COVID-19 and SARS-CoV-2 Infection)	Definition of seroconversion for participants seropositive at Baseline has been deleted.	Seroconversion in participants seropositive at Baseline was not assessed for the Part A Blinded Phase and will not be assessed for Part B or Part C.
Section 8.1.2 (Surveillance for COVID-19 Symptoms); Section 11.1 (Appendix 1: Schedules of Events)	Clarified that 2 NP swab samples are collected at Illness Visits.	To enhance consistency of data collection at study sites.
Section 8.3.3 (Adverse Events of Special Interest); Section 10 (References);<br>Section 11.5 (Appendix 5: Adverse Events of Special Interest Terms)	Added CDC case definitions for myocarditis and pericarditis, as well as other terms for other potentially relevant adverse events of special interest.	To provide guidance to the investigators regarding assessing and reporting adverse events of special interest, including myocarditis and pericarditis, for this study population.
Section 8.4.4 (Independent Cardiac Event Adjudication Committee)	Added Section 8.4.4, Independent Cardiac Event Adjudication Committee.	To describe the proposed mechanism to assess risk of myocarditis and pericarditis in the study population (to address Center for Biologics Evaluation and Research [CBER] request to describe how risk of myocarditis and pericarditis will be assessed in the study population receiving mRNA-1273).

Section # and Name	Description of Change	Brief Rationale
Section 8.8 (Exploratory Assessments and Biomarkers)	Text updated to reflect initiation of Part C of the study.	To reflect more comprehensively the current thinking on potential exploratory assessments.
Section 9.5.3 (Immunogenicity Analyses)	A definition of seroresponse was added.	To more clearly define the relevant antibody response.
Section 11.1 (Appendix 1: Schedules of Events; Table 17 and Table 18)	Adjusted the collection of eDiary data to be weekly from Day 64 through Day 759.	To ensure no gap in eDiary data collection, as they run weekly, regardless of scheduled study site visits or safety calls.
Section 11.1 (Appendix 1: Schedules of Events; Table 18)	Reduced the frequency of safety calls from every month after Visit 5 (Month 13) to every 2 months after Visit 5.	To reduce participant burden. COVID-19 and safety surveillance will be maintained through the continued weekly surveillance by eDiary during the second year of the Surveillance Phase.

Amendment 8, 23 Mar 2021

Main Rationale for the Amendment:

The purpose of this amendment is to update language around unblinding and open-label dosing in the context of the majority of participants already having completed their blinded follow-up. In addition, the amendment updates the reporting of serious COVID-19 cases.

The summary of changes table provided here describes the major changes made in Amendment 8 relative to Amendment 7, including the sections modified and the corresponding rationales. As applicable, the synopsis of Amendment 8 has been modified to correspond to changes in the body of the protocol.

Summary of Major Changes From Protocol Amendment 7 to Protocol Amendment 8:

Section # and Name	Description of Change	Brief Rationale
Section 4.1.2 (Part B, the Open-Label Observational Phase)	States that, due to ethical and scientific concerns, all participants will be unblinded once investigational product is no longer available. In addition, the text indicates that if participants want to receive Emergency Use Authorization (EUA) vaccine outside of the study, participants will need to be withdrawn from the study.<br><br>The amendment also provides directions for unblinding after Biologics License Application (BLA) database lock.	At the time of this amendment, more than 95% of the enrolled participants have completed blinded follow-up, either through being unblinded or study discontinuation. Therefore, there is no scientific value for participants to remain blinded.<br>In addition, non-study EUA vaccines are widely available, so ethically, participants should be unblinded to ensure they can make an informed choice if they wish to receive EUA COVID-19 vaccine outside of the study.
Section 8.3.7 (Time Period and Frequency for collecting Adverse Events and Serious Adverse Event Information) and Section 8.3.10 (Reporting Adverse Events)	Reporting of only serious COVID-19 cases within 24 hours to Sponsor or it's designee.	Nonserious COVID-19 cases are an efficacy endpoint, so reporting to Pharmacovigilance is no longer warranted.

Amendment 7, 10 Feb 2021

Main Rationale for the Amendment:

The purpose of this amendment is to collect safety information on suspected cases of anaphylaxis and to include participant history of facial injections or dermal fillers in the eDiary.

The summary of changes table provided here describes the major changes made in Amendment 7 relative to Amendment 6, including the sections modified and the corresponding rationales. The synopsis of Amendment 7 has been modified to correspond to changes in the body of the protocol.

Summary of Major Changes From Protocol Amendment 6 to Protocol Amendment 7:

Section # and Name	Description of Change	Brief Rationale
Title Page, Protocol Approval Page, Headers, Protocol Amendment Summary of Changes	Updated the protocol version and date.	To reflect the new version and date of the protocol.

Section # and Name	Description of Change	Brief Rationale
Sections 1.1 (Synopsis),<br>6.2.2 (Administration of Investigational Product),<br>6.2.8.1 (Planned Unblinding)	Clarified that study site personnel who were blinded during the Blinded Phase will be unblinded **at the participant level** at the Participant Decision clinic visit.	To clarify that the site personnel is being unblinded only at the Participant Decision clinic visit.
Sections 1.1 (Synopsis), 4.1.2 (Part B, the Open-Label Observational Phase)	Clarified that with regard to EUA eligibility in Part B, the CDC-EUA guidance specifications will supersede eligibility criteria in the protocol.	To clarify that the CDC-EUA guidance specifications supersede protocol-specified criteria for EUA eligibility in Part B.
Section 4.1 (General Design)	Corrected the number of scheduled clinic visits and added mention of the participant decision visit.	To clarify the number of scheduled visits.
Section 4.1.2 (Part B, the Open-Label Observational Phase)	Updated text to clarify requirements for Part B subjects who are unblinded and received only 1 dose of mRNA-1273 during Part A.	Clarification of requirements for Part B subjects who received only 1 dose of mRNA-1273 during Part A.
Section 6.2.4 (Packaging and Labeling)	Updated to indicate that vaccine product is packaged in a 10R glass vial with a 6-dose, 5.0-mL or 10-dose, 6.3-mL fill volume.	To include IP presentation for additional supplies that may be used in Part B of the study.
Section 6.2.5 (Storage)	Updated to specify storage requirements for the 6-dose and 10-dose mRNA vaccine.	To include IP storage conditions for additional supplies that may be used in Part B of the study.
Sections 6.4.2 (Concomitant Medications and Therapies),<br>7.2 (Discontinuation of Study Treatment), 8.2.2 (Use of Electronic Diaries), 8.2.6 (Blood Sampling Volumes, Table 4)	Clarified requirements for Part A and Part B.	Clarification of concomitant medication, eDiary, and blood sampling procedures during Part A and Part B.
Section 8.2.2 (Use of Electronic Diaries), Appendix 1 (Schedule of Events, Table 17 footnote 7)	Clarified that eDiary prompts to surveil for weekly COVID-19 symptoms will be performed for all participants in Part A and Part B.<br><br>Added eDiary prompt to solicit collection of participant history of facial injections or dermal fillers.	Clarification.<br><br>To assure all concomitant medications are recorded.

Section # and Name	Description of Change	Brief Rationale
Section 8.2.4 (Physical Examination)	Removed body mass index from the participant decision visit.	Clarification.
Section 8.2.6 (Blood Sampling Volumes)	Updated maximum planned volume of blood collection for the complete study to include a range.	Clarification.
Section 8.3.2 (Medically Attended Adverse Events)	Added suspected cases of anaphylaxis as an MAAE and requirement to report as an SAE.	Sponsor collecting anaphylaxis information.
Section 9.4 (Analysis Populations, Table 8); Synopsis	Clarified Immunogenicity Subset.	Clarification.
Section 9.5.1.1 (Efficacy Analysis on Primary Endpoint), Appendix 4 (Statistical Appendices, Table 23 and Table 24)	Updated intercurrent events and estimands.	Clarification of estimand.
Section 9.5.1.3 (Long-term Efficacy Analysis); Synopsis	Updated case count parameters.	Provide long-term efficacy analysis strategy relevant to change in study design.
Appendix 1 (Schedule of Events, Table 21 footnote 2, and Table 22 footnote 2)	Updated physical examination to symptom-directed physical examination and removed height and weight requirements from the Open-Label Clinic Visits.	Clarification.

Amendment 6, 23 Dec 2020

Main Rationale for the Amendment:

The purpose of this amendment is to inform all ongoing study participants of the availability of and eligibility criteria of any COVID-19 vaccine made available under an EUA and to offer participants who originally received placebo in this study the potential benefit of vaccination against COVID-19, given that the primary efficacy endpoint for mRNA-1273 against COVID-19 was met per the protocol-defined IA.

The summary of changes table provided here describes the major changes made in Amendment 6 relative to Amendment 5, including the sections modified and the corresponding rationales. The synopsis of Amendment 6 has been modified to correspond to changes in the body of the protocol.

Summary of Major Changes From Protocol Amendment 5 to Protocol Amendment 6:

Section # and Name	Description of Change	Brief Rationale
Title Page, Protocol Approval Page, Headers, Protocol Amendment Summary of Changes	Updated the protocol version and date.	To reflect the new version and date of the protocol.
Sections 1.1 (Synopsis), 1.2 (Schema), 4.1.2 (Part B, the Open-Label Observational Phase), 6.2.8.1 (Planned Unblinding), 7.3.1 (Participant Withdrawal), and 11.1 (Schedules of Events)	Added a “Participant Decision clinic visit”.	This visit provides the opportunity for study site personnel to discuss with and offer to participants, the choice to be unblinded, as well as offering to participants who originally received placebo, the choice to receive active vaccination with mRNA-1273 and possible vaccination against COVID-19. Participants will also sign a revised informed consent form at this visit.
Sections 1.1 (Synopsis), 1.2 (Schema), 4.1 (General Design), 4.2 (Scientific Rationale for Study), 6.2 (Method of Randomly Assigning Participants to Treatment Groups [Part A], Blinded Phase Only), and 11.1 (Schedules of Events)	Changes to study design that split the study into a Blinded Phase and an Open-Label Observational Phase.	These changes accommodate the breaking of the blind and offering of open-label vaccination to all participants. This change also distinguishes the Open-Label Supplemental Schedule of Events (Vaccination Phase) for participants who received placebo, and who meet EUA eligibility, and request to receive active vaccine.
Sections 1.1 (Synopsis), 1.2 (Schema), 4.1.2 (Part B, the Open-Label Observational Phase), and 11.1 (Schedules of Events)	Added a Supplemental SoE and a Modified Supplemental SoE.	These provide paths for participants who choose to be unblinded and receive mRNA-1273, and for participants who previously received only 1 dose of blinded mRNA-1273 during the study (Blinded Phase), to transition into the Open-label Phase of the study.

Section # and Name	Description of Change	Brief Rationale
Section 2.2.2 (Clinical Studies)	Updated status of ongoing clinical studies, including this study (mRNA-1273-P301).	The status of the 3 clinical studies (one Phase 1, one Phase 2a, and this Phase 3 study) have changed since Amendment 5.<br>In addition, the results of the interim analyses in this study of the primary efficacy endpoint (prevention of COVID-19 infection), a major secondary endpoint (prevention of severe COVID-19), and safety and reactogenicity endpoints are now available and are provided here. These results provide the justification for offering participants the opportunity to receive active IP (mRNA-1273) and the potential benefit of vaccination against COVID-19.
Section 1.1 (Synopsis) and 9.5.1.3 (Long-term Efficacy Analysis)	Addition of long-term efficacy analyses.	Allows for the analysis of the long-term efficacy of mRNA-1273 in the following treatment cohorts:<br>• mRNA-1273 Cohort: Participants randomized to mRNA-1273 in the Blinded Phase.<br>• Placebo Cohort: Participants randomized to Placebo in the Blinded Phase and did not crossover to mRNA-1273 in the Open-label Phase.<br>• Placebo-mRNA-1273 Cohort: Participants randomized to Placebo in the Blinded Phase and crossed over to mRNA-1273 in the Open-label Phase.

Section # and Name	Description of Change	Brief Rationale
Section 2.3.3 (Overall Benefit/Risk Conclusion)	Updated the Benefit/Risk Assessment.	Based on the interim results from this pivotal Phase 3 study, mRNA-1273 prevents COVID-19 and severe COVID-19. The demonstrated clinical benefit of mRNA-1273 is supported by evidence of a robust immune response both in terms of binding antibodies (bAbs) and neutralizing antibodies (nAbs) as well as the induction of CD4+ T-cells with a Th-1 dominant phenotype.
Section 11.1	Added footnote to Table 16.	To clarify that the eDiary Safety prompts will be triggering off on Day 61 to take into consideration the (-3 day) window allowance on Day 29.

Amendment 5, 11 Nov 2020

Main Rationale for the Amendment:

The main purpose of this amendment is to clarify that the eDiary prompts for safety surveillance will be weekly and to add Month 19 safety call.

The summary of changes table provided here describes the major changes made in Amendment 5 relative to Amendment 4, including the sections modified and the corresponding rationales. The synopsis of Amendment 5 has been modified to correspond to changes in the body of the protocol.

Summary of Major Changes From Protocol Amendment 4 to Protocol Amendment 5:

Section # and Name	Description of Change	Brief Rationale
Title Page, Protocol Approval Page, Headers, Protocol Amendment Summary of Changes	Updated the protocol version and date.	To reflect the new version and date of the protocol.
Section 11.1	Updated Appendix 1 (Tables 16 and 17) .	To clarify the weekly schedule for eDiary prompts through Year 1 and Year 2 of follow-up.
Section 11.1	Added Month 19 Safety Call.	To clarify that participants will have continuous weekly (eDiary prompts) and monthly (safety calls) through Year 1 and Year 2 of follow-up.

Amendment 4, 30 Sep 2020

Main Rationale for the Amendment:

The main purpose of this amendment is to increase the upper limit for stratification of enrolled participants considered "at risk" at Screening to 50%.

The summary of changes table provided here describes the major changes made in Amendment 4 relative to Amendment 3, including the sections modified and the corresponding rationales. The synopsis of Amendment 4 has been modified to correspond to changes in the body of the protocol.

Summary of Major Changes From Protocol Amendment 3 to Protocol Amendment 4:

Section # and Name	Description of Change	Brief Rationale
Title Page, Protocol Approval Page, Headers, Protocol Amendment Summary of Changes	Updated the protocol version and date.	To reflect the new version and date of the protocol.
Section 6.2.1.1 (Stratification)	Increased the upper limit for stratification of enrolled participants considered "at risk" at Screening to up to 50%, from 40%.	To enhance the diversity of the study population by increasing the number of racial and ethnic minority participants in the study. as participants from these communities often have higher rates of comorbidities.
Section 9.5.1.1 (Efficacy Analysis on Primary Endpoint) and Section 11.4.1 (Estimands and Estimand Specifications)	Updated the description of an intercurrent event (unrelated death) and its strategy	To align with the description in the Statistical Analysis Plan.

Amendment 3, 20 Aug 2020

Main Rationale for the Amendment:

The main purpose of this amendment is to make changes to the protocol in response to feedback from Center for Biologics Evaluation and Research.

The summary of changes table provided here describes the major changes made in Amendment 3 relative to Amendment 2, including the sections modified and the corresponding rationales. Minor editorial or formatting changes are not included in this summary table. The synopsis of Amendment 3 has been modified to correspond to changes in the body of the protocol.

Summary of Major Changes From Protocol Amendment 2 to Protocol Amendment 3:

Section # and Name	Description of Change	Brief Rationale
Title Page, Protocol Approval Page, Headers, Protocol Amendment Summary of Changes	Updated the protocol version and date.	To reflect the new version and date of the protocol.

Section # and Name	Description of Change	Brief Rationale
Section 5 (Study Population)	Added a sentence to describe the intent to enroll a representative sample of racial and ethnic minority participants in the study.	To enhance the diversity of the study population.
Section 5.2 (Exclusion Criteria)	Added clarification to exclusion criterion #11 to define the parameters based on screening CD4 count and viral load for exclusion of study participants.	To clarify the definition of controlled HIV disease in the exclusion criterion such that only participants with well-controlled HIV disease are enrolled in the study.
Section 5.2 (Exclusion Criteria)	Removed “topical tacrolimus” from exclusion criterion #12.	No evidence to support any systemic effect of topical tacrolimus to warrant excluding them.
Section 6.2.1.1 (Stratification)	Added HIV infection to the risk factors at Screening.	To stratify participants based on certain risk factors.
Section 8.2.3 (Demographics/Medical History)	Added collection of risk factors for complications of COVID-19.	To document the diagnosis of any risk factor for complications of COVID-19 used for stratification.
Section 9.3 (Sample Size Determination)	Removed redundant bullet.	To remove redundancy in the assumptions listed.
Section 9.5.2 (Safety Analyses)	Removed safety analysis by serostatus.	No added value for this analysis. Other subgroup analyses may be specified in the SAP, as needed.
Section 9.5.5 (Subgroup Analyses)	Removed the categories (white, non-white) from the Race Variable.	To allow for more refined Race categorization being collected in the eCRF.
Appendix 11.3	Removed “cessation of exogenous hormonal therapy”.	To allow postmenopausal women to take these medications if needed for the treatment of the symptoms of menopause.
Appendix 11.3	Removed “using hormonal contraception” from postmenopausal female with high FSH levels.	Not a standard of care for women for treatment of menopausal symptoms.

Amendment 2, 31 Jul 2020:

Main Rationale for the Amendment:

The main purpose of this amendment is to provide more intensive surveillance of symptoms and severity of cases of COVID-19 after the first dose of IP.

The summary of changes table provided here describes the major changes made in Amendment 2 relative to Amendment 1, including the sections modified and the corresponding rationales. Minor editorial or formatting changes are not included in this summary table. The synopsis of Amendment 2 has been modified to correspond to changes in the body of the protocol.

Summary of Major Changes From Protocol Amendment 1 to Protocol Amendment 2:

Section # and Name	Description of Change	Brief Rationale
Title Page, Protocol Approval Page, Headers, Protocol Amendment Summary of Changes	Updated the protocol version and date.	Reflect the new version and date of the protocol.
Section 4.1 (General Design), Section 7.2 (Discontinuation of Study Treatment), Section 8.1.1 (Efficacy Assessments Related to COVID-19 and SARS-CoV-2 Infection), Section 8.1.2 (Surveillance for COVID-19 Symptoms),Table 14 (Schedule of Events [Vaccination Phase, Day 1 – Day 57])	Added a Day 29 NP swab prior to Dose 2.	Improve surveillance for asymptomatic infection prior to Dose 2 to assist in discriminating COVID-19 symptoms from solicited systemic reactions after vaccination.
Section 4.1 (General Design), Table 15 (Schedule of Events [Surveillance Phase, Day 64 – Day 394])	Changed the clinic visit at Month 4 to a safety call.	Improve participant safety and adherence to protocol.
Section 4.1 (General Design), Section 8.1.2 (Surveillance for COVID-19 Symptoms), Table 14 Footnote 5 (Schedule of Events [Vaccination Phase, Day 1 – Day 57])	Clarified symptom duration (> 48 hours) to trigger NP swab collection and investigator judgement whether to obtain an NP swab in the 7 days following vaccination due to overlap of solicited systemic symptoms and COVID-19.	Improve surveillance for cases of COVID-19.
Section 4.1 (General Design), Section 8.1.3 (Convalescent Period Starting with the Illness Visit), Section 8.1.4 (Ancillary Supplies for Participant Use)	Removed mention of continuous biometric monitoring.	Simplify data management and clinical operations.
Section 5.1 (Inclusion Criteria), Section	Removed the inclusion criterion regarding male contraception.	Requirement not generally applicable for a Phase 3 vaccine study.
Section 5.2 (Exclusion Criteria), Section 6.4.3 (Concomitant Medications and Vaccines that May Lead to the Elimination of a Participant from Per-Protocol Analyses)	Clarified language around influenza vaccination.	Make restrictions on influenza vaccination less restrictive than for other licensed vaccines relative to administration of investigational product.
Section 5.2 (Exclusion Criteria)	Removed restriction on enrollment of participants with human immunodeficiency virus (HIV) infection.	Participants on stable antiretroviral therapy are not excluded, which diversifies the participant group.
Section 8.3.7 (Time Period and Frequency for Collecting AE and SAE Information), Section 8.3.10 (Reporting Adverse Events)	Added expedited reporting of confirmed COVID-19 cases.	Improve surveillance for cases of COVID-19
Section 8.4.2 (Data and Safety Monitoring Board)	Added an Oversight Group.	Clarify responsibility for declaring early efficacy or for taking action to stop, pause, or continue the study.

Section # and Name	Description of Change	Brief Rationale
Section 8.4.2 (Data and Safety Monitoring Board)	Updated description of monitoring for potential harm, including details on case counting for the purpose of harm monitoring to align with a DSMB analysis plan.	Clarify description for harm monitoring, based on FDA feedback.

Amendment 1, 26 Jun 2020:

Main Rationale for the Amendment:

The main purpose of this amendment is to provide more intensive surveillance of symptoms and severity of cases of COVID-19 after the first dose of IP.

The summary of changes table provided here describes the major changes made in Amendment 1 relative to the original protocol, including the sections modified and the corresponding rationales. Minor editorial or formatting changes are not included in this summary table.

Summary of Major Changes in Protocol Amendment 1:

Section # and Name	Description of Change	Brief Rationale
Title page, Signature page, and header	Updated the protocol version and date.	Reflect the new version and date of the protocol.
Synopsis; Section 4.1, General Design; Section 8.1.3, Convalescent Period; Section 11.1, schedules of events, Table 17	Increased the frequency of telemedicine contacts during the Convalescent Period following the Initial Illness Visit.	Improve the amount and quality of COVID-19 symptom data collected during the Convalescent Period.
Synopsis; Section 4.1, General Design; Section 8.1.3, Convalescent Period; Section 8.1.4, Ancillary Supplies for Participant Use; Section 11.1, schedules of events, Table 17	Added monitoring of oxygen saturation to the Convalescent Period.	Improve surveillance for incidence of COVID-19 during the study.
Synopsis; Section 8.1.3, Convalescent Period; Section 8.1.4, Ancillary Supplies for Participant Use; Section 11.1, schedules of events, Table 17	Increased the frequency of monitoring for SARS-CoV-2, using saliva as the preferred sample matrix after the Illness Visit during the Convalescent Period.	Improve the sensitivity of monitoring the time course of viral shedding during COVID-19.
Synopsis; Section 3, Objectives and Endpoints	Added a respiratory sample for hospitalized participants as a matrix for confirming the presence of SARS-CoV-2.	Increase the potential number of evaluable COVID-19 cases.
Synopsis; Section 3, Objectives and Endpoints	Broadened the definition for seroconversion at a participant level.	Included neutralizing antibody (nAb) in addition to binding antibody (bAb).

Section # and Name	Description of Change	Brief Rationale
Section 8, Study Assessments and Procedures; Section 8.1.3, Convalescent Period; Section 8.1.4, Ancillary Supplies for Participant Use; Section 8.2.2, Use of Electronic Diaries; Section 11.1, schedules of events, Table 17	Eliminated paper diaries, substituting an instruction card listing symptoms and a severity grading system to enhance the quality of data obtained by telemedicine contacts.	Reduce fomite transmission of SARS-CoV-2 and increase frequency of investigative staff interactions with participants during the Convalescent Period.
Section 8.1.2, Surveillance for COVID-19 Symptoms	Decreased the number of symptoms (to one of the 11 CDC symptoms) that would result in an Illness Visit.	Increase the likelihood of capturing all COVID-19 cases in the earliest stage of disease.
Section 8.2.2, Use of Electronic Diaries	Expanded the scope of eDiary prompts and data collected during the Surveillance Phase.	Increase the likelihood of capturing all COVID-19 cases in the earliest stage of disease.

Signature Page for VV-CLIN-003185 v4.0

Approval	Rituparna Das<br>Clinical<br>12-Apr-2022 01:48:09 GMT+0000

Signature Page for VV-CLIN-003185 v4.0

ModernaTX, Inc.

Protocol mRNA-1273-P301

A Phase 3, Randomized, Stratified, Observer-Blind, Placebo-Controlled Study to Evaluate the Efficacy, Safety, and Immunogenicity of mRNA-1273 SARS-CoV-2 Vaccine in Adults Aged 18 Years and Older

Statistical Analysis Plan

Part B and Part C

SAP Version 2.0

Version Date of SAP: 16 May 2022

Prepared by:

PPD
3575 Quakerbridge Road
Suite 201
Hamilton, NJ 08619

DOCUMENT HISTORY

Version	Date	Description of main modifications
1.0	15 January 2022	Original version
2.0	16 May 2022	• Added secondary (immunogenicity) objective and endpoint specific for Part C to align with Protocol Amendment 10.<br>• Added analysis sets for effectiveness and safety analyses for the primary series.<br>• Clarification made throughout the document.

TABLE OF CONTENTS

LIST OF ABBREVIATIONS VI

1. INTRODUCTION 9

2. STUDY OBJECTIVES 11

2.1. PRIMARY OBJECTIVES 11
2.2. SECONDARY OBJECTIVES 11
2.2.1. Secondary Efficacy Objectives 11
2.3. EXPLORATORY OBJECTIVES 11

3. STUDY ENDPOINTS 12

3.1. PRIMARY ENDPOINTS 12
3.1.1. Primary Efficacy Endpoint (Part A: Blinded Phase Only) 12
3.1.2. Safety Endpoints for Primary Safety Objective 12
3.2. SECONDARY ENDPOINTS 13
3.2.1. Secondary Efficacy Endpoints (Part A: Blinded Phase Only) 13
3.2.2. Secondary Immunogenicity Endpoints 14
3.3. ENDPOINTS TO ASSESS EFFECTIVENESS OF MRNA-1273 16
3.4. EXPLORATORY ENDPOINTS 16
3.5. OVERALL STUDY DESIGN 17
3.6. STATISTICAL HYPOTHESIS (PART A) 21
3.7. SAMPLE SIZE AND POWER 21
3.8. RANDOMIZATION 21
3.9. BLINDING AND UNBLINDING 21

4. ANALYSIS POPULATIONS 22

4.1. PART C SAFETY SET 23
4.2. PART C PER-PROTOCOL RANDOM SUBCOHORT FOR IMMUNOGENICITY (PART C PPRSI) 23
4.3. PART C PER-PROTOCOL IMMUNOGENICITY SUBSET (PART C PPIS) 23
4.4. PART C PER-PROTOCOL IMMUNOGENICITY SUBSET – PRE-BOOSTER SARS-COV-2 NEGATIVE (PART C PPIS-NEG) 23
4.5. PART C PER-PROTOCOL (PP) SET 24
4.6. PER-PROTOCOL (PP) PRIMARY SERIES SET 24
4.7. PRIMARY SERIES SAFETY SET 25

5. STATISTICAL ANALYSIS 25

5.1. GENERAL CONSIDERATIONS 25
5.1.1. General Considerations, Derivations and Strategy for incomplete/missing data 25
5.1.2. Baseline (Pre-Dose 1) in Part A, Unblinding/PDV in Part B, Pre-booster in Part C and pre-Vaccination/pre-Dose 1 of mRNA-1273 26

5.1.3. SARS-CoV-2 status ... *27*
5.1.4. Analysis Strategy and Treatment Groups ... *28*
5.1.5. Analysis Periods ... *30*
5.2. BACKGROUND CHARACTERISTICS ... 33
5.2.1. Subject Disposition ... *33*
5.2.2. Demographics and Baseline Characteristics ... *33*
5.2.3. Medical History ... *34*
5.2.4. Prior and Concomitant Medications ... *34*
5.2.5. Study Exposure ... *35*
5.2.6. Major Protocol Deviations ... *35*
5.3. EFFECTIVENESS ANALYSIS ... 35
5.3.1. Analysis of the Primary Efficacy Endpoint for Part B, Part C, and Long-term Analyses ... *36*
5.3.1.1. Primary Efficacy Endpoint Definition/Derivation ... 36
5.3.1.2. Analysis approach for effectiveness of vaccine ... 37
5.3.1.2.1. Effectiveness of the primary series ... 38
5.3.1.2.1.1. Relative Effectiveness of recent vaccinated vs early vaccinated (the Primary Series of mRNA-1273) ... 39
5.3.1.2.2. Effectiveness of the booster ... 39
5.3.1.2.3. Effectiveness of booster (50 µg mRNA-1273) ... 40
5.3.1.3. Subgroup Analysis ... 41
5.3.1.4. Variant-specific analysis of effectiveness ... 41
5.3.2. Analysis of Other Efficacy Endpoints ... *42*
5.3.2.1. Derivation of Other Efficacy Endpoint ... 42
5.3.2.1.1. Derivation of Secondary Efficacy Endpoint: Severe COVID-19 ... 42
5.3.2.1.2. Derivation of a secondary definition of COVID-19 ... 42
5.3.2.1.3. Derivation of serologically confirmed SARS-CoV-2 infection or COVID-19 regardless of symptomatology or severity ... 43
5.3.2.1.4. Derivation of asymptomatic SARS-CoV-2 infection ... 43
5.3.2.2. Analysis of Other Efficacy Endpoints ... 44
5.4. SAFETY ANALYSIS ... 44
5.4.1. Unsolicited Treatment-emergent Adverse Events ... *44*
5.4.1.1. Overview of Unsolicited TEAEs ... 45
5.4.1.2. TEAEs by System Organ Class and Preferred Term ... 46
5.4.1.3. Subgroup Analysis of TEAEs ... 47
5.4.2. Other Safety Endpoints Pregnancies ... *47*
5.5. IMMUNOGENICITY ANALYSIS ... 47
5.5.1. Immunogenicity Data ... *48*
5.5.2. Summary of Antibody-Mediated Immunogenicity Endpoints ... *49*
5.5.2.1.1. Seroresponse Rate (SRR) of the Primary Series and Booster ... 49
5.5.3. Analysis of Antibody-Mediated Immunogenicity Endpoints ... *51*
5.5.3.1. Durability of immune response to the Primary Series: Mixed Model for Repeated Measures (MMRM) ... 51

5.5.3.2. Immunobridging of response of a Booster as compared to Primary Series 52
5.6. EXPLORATORY ANALYSIS OF EFFECTIVENESS 54
5.6.1. Durability of Vaccine Efficacy of Primary Series (including data from both blinded and OL- Phase before boost Data).. 54
5.6.1.1. Methods Proposed by Tsiatis and Davidian (2021a/b)............................ 55
5.6.1.2. Methods Proposed by Lin et al (2021, 2022) 56
5.6.2 The effectiveness of booster.. 57
5.6.2.1 Methods Proposed by Lin et al (2021, 2022) 57
5.6.2.2 Methods Proposed by Fintzi and Follmann (2021) 58

6 CHANGES FROM PLANNED ANALYSES IN PROTOCOL........................60

7 REFERENCES..60

8 LIST OF APPENDICES...62

APPENDIX A STANDARDS FOR VARIABLE DISPLAY IN TFLS...62
APPENDIX B ANALYSIS VISIT WINDOWS 63
APPENDIX C IMPUTATION RULES FOR MISSING DATES OF PRIOR/CONCOMITANT MEDICATIONS AND NON-STUDY VACCINATIONS...67
APPENDIX D IMPUTATION RULES FOR MISSING DATES OF AES.....................................70
APPENDIX E SCHEDULE OF EVENTS 71
APPENDIX F TREATMENT GROUPS/COHORTS BASED ON THE PROTOCOL AND TREATMENT GROUPS/COHORTS BASED ON ACTUAL TREATMENT...................................72
APPENDIX G CALCULATION OF OBSERVED GMT AND GMFR...74
APPENDIX H DESCRIPTION OF PSEUDOVIRUS NEUTRALIZATION ASSAY USED IN P301 75
APPENDIX I ASSAY-SPECIFIC CRITICAL FOLD-RISE (CFR) CRITERIA ESTABLISHED FOR SELECT ASSAYS 76

List of Abbreviations

Abbreviation	Definition
AE	adverse event
ANCOVA	analysis of covariance
AR	adverse reaction
ARDS	acute respiratory distress syndrome
bAb	binding antibody
BARDA	Biomedical Advanced Research and Development Authority
BD	booster dose
BMI	body mass index
BOD	burden of disease
BOI	burden of infection
CDC	Centers for Disease Control and Prevention
CFR	critical fold-rise
CI	confidence interval
COVID-19	coronavirus disease 2019
COV-INF	SARS-CoV-2 infection or COVID-19 regardless of symptomatology or severity
CRO	contract research organization
CSP	clinical study protocol
DHHS	Department of Health and Human Services
ECMO	extracorporeal membrane oxygenation
eCRF	electronic case report form
eDiary	electronic diary
ELISA	enzyme-linked immunosorbent assay
EUA	Emergency Use Authorization
FAS	full analysis set

Abbreviation	Definition
GM	geometric mean
GMFR	geometric mean fold rise
GMR	geometric mean ratio
GMT	geometric mean titer
HIV	human immunodeficiency virus
HR	hazard ratio
ICH	International Council for Harmonisation
IP	investigational product
IRT	interactive response technology
LOD	limit of detection
LLOQ	lower limit of quantification
MAAEs	medically-attended adverse events
MedDRA	Medical Dictionary for Regulatory Activities
MMRM	Mixed Model for Repeated Measures
mRNA	messenger ribonucleic acid
MSD	Meso scale discovery
nAb	neutralizing antibody
NIAID	National Institute of Allergy and Infectious Diseases
NP	nasopharyngeal
OL	open label
PDV	Participant decision visit
PH	proportional hazard
PP	per-protocol
PS	primary series
PT	preferred term
RCDF	reverse cumulative distribution function
REML	restricted (or residual, or reduced) maximum likelihood

Abbreviation	Definition
RT-PCR	reverse transcription polymerase chain reaction
SAE	serious adverse event
SAP	statistical analysis plan
SARS-CoV-2	severe acute respiratory syndrome coronavirus 2
SAS	Statistical Analysis System
SD	standard deviation
SOC	system organ class
SRR	seroresponse rate
TEAE	treatment-emergent adverse event
ULOQ	upper limit of quantification
VE	vaccine efficacy
WHO	World Health Organization
WHODD	World Health Organization drug dictionary

1. Introduction

This statistical analysis plan (SAP) for Part B and Part C, version 2.0, is the amendment of version 1.0, which is based on clinical study protocol (CSP) Amendment 10, dated 07-APR-2022 and the approved electronic case report form (eCRF) Version 16.007. Part B, the Open-Label (OL) Observational Phase of the study, is prompted by the authorization of a coronavirus disease 2019 (COVID-19) vaccine under Emergency Use Authorization (EUA). The CSP Amendment 6 informed all ongoing study participants of the availability and eligibility criteria of any COVID-19 vaccine made available under an EUA and offers participants who originally received placebo in this study the potential benefit of vaccination against COVID-19, given that the primary efficacy endpoint for messenger ribonucleic acid (mRNA)-1273 against COVID-19 was met per the protocol-defined interim analysis. Part C, the Booster Dose (BD) Phase of the study, is prompted by interim results of an ongoing Moderna Phase 2a study (mRNA-1273-P201) that demonstrated enhanced immune responses compared to pre-boost levels and met the noninferiority criteria outlined for booster approval. The CSP Amendment 9 provides the option for a BD to all eligible participants enrolled in Part B.

SAP, Version 1.0 (dated 08 Sep 2020) and Version 2.0 (dated 08 Mar 2021) were developed. For the randomized blinded phase (Part A), three analyses have been performed with Vaccine efficacy assessed:

- an IA of efficacy met the primary objective of efficacy based on the formal pre-specified study success criterion based on a data snapshot on 11 November 2020,
- the primary analysis based on a data snapshot on 25 Nov 2020, and the final analysis of blinded phase/Part A based on database lock on 04 May 2021 with a data cutoff date of 26 March 2021.
- the final analysis of blinded phase/Part A based on database lock of 04 May 2021

The primary objective of VE of mRNA-1273 to prevent COVID-19 has been met at the interim analysis based on a total of 95 adjudicated COVID-19 starting 14 days after the

Dose 2 using the Per-Protocol Set with an VE of 94.5% (95% CI: 86.5, 97.8); $p < 0.0001$ (data snapshot on 25 Nov 2020).

A clinical study report (CSR) has been developed based on the final analysis of the randomized blinded phase (Part A) dated 03 August 2021. Efficacy, safety and immunogenicity of Part A have been analyzed and reported in the CSR for Part A. A CSR addendum presenting safety and efficacy results of the Part B Open-label phase was developed based on the database lock on 04 May 2021.

Therefore, analyses of blinded phase (Part A) data are considered to be completed.

This SAP Part B and Part C focuses on the OL phase and BD phase of the study and provides statistical analysis details/data derivations focusing on Part B and Part C. The SAP Part B and Part C will also provide more details for the unblinded phase (Part B) analyses that were briefly described in SAP Version 2.0, dated 08-MAR-2021. In addition, methods for analysis of the primary series based on Part B and Part A, and for mRNA-1273 using data across parts is included.

Modifications or additions to the analysis plan which are not "principal" in nature is also documented, if the methods in this SAP Part B and Part C differ from the methods described in the protocol, the SAP will prevail.

PPD Biostatistics and programming team, designee of Moderna Biostatistics and Programming department, will perform the statistical analysis; Statistical Analysis System (SAS) Version 9.4 or higher will be used, and R or RStudio may be used.

In this document, subject and participant are used interchangeably; injection of IP, injection, and dose are used interchangeably; vaccination group and treatment group are used interchangeably; Part A and the blinded phase are used interchangeably; Part B and the Open-label (OL) phase are used interchangeably; Part C and the booster dose (BD) phase are used interchangeably; treatment groups and treatment cohorts are used interchangeably; titers and levels are used interchangeably.

2. Study Objectives

2.1. Primary Objectives

Refer to Protocol Section 3 and SAP Part A v2.0. The primary objective of VE of mRNA-1273 to prevent COVID-19 has been met at the interim analysis based on a total of 95 adjudicated COVID-19 starting 14 days after the Dose 2 using the Per-Protocol Set with an VE of 94.5% (95% CI: 86.5, 97.8); $p < 0.0001$ (data snapshot on 25 Nov 2020).

For the blinded phase (Part A), three analyses have been performed with Vaccine efficacy assessed: an IA of efficacy met the primary objective of efficacy based on the formal pre-specified study success criterion, the primary analysis based on a data snapshot on 25 Nov 2020, and the final analysis of blinded phase/Part A based on database lock of 04 May 2021. A clinical study report (CSR) has been developed based on the final analysis of the randomized blinded phase (Part A) dated 03 August 2021.

2.2. Secondary Objectives

2.2.1. Secondary Efficacy Objectives

Refer to protocol Section 3 and SAP version 2.0 for Part A and Part B.

The secondary objective for Part C is to infer effectiveness of the 50 µg of mRNA-1273 booster by establishing noninferiority of Ab response after the booster dose compared to the primary series of mRNA-1273 using GMT values of serum Ab and seroresponse rate (SRR) of post booster in Part C compared with primary series recipients of mRNA-1273.

2.3. Exploratory Objectives

Refer to SAP version 2.0 for the exploratory objectives for Part A and Part B.

The exploratory objectives for Part C are:

- To evaluate the safety of a booster dose of mRNA-1273.
- To evaluate the immunogenicity of a booster dose of mRNA-1273.

3. Study Endpoints

3.1. Primary Endpoints

3.1.1. Primary Efficacy Endpoint (Part A: Blinded Phase Only)

The primary efficacy objective will be evaluated by the VE of mRNA-1273 to prevent the first occurrence of COVID-19 starting 14 days after the second dose of IP, where COVID-19 is defined as symptomatic disease based on the following criteria:

- The participant must have experienced at least TWO of the following systemic symptoms: Fever (≥ 38ºC), chills, myalgia, headache, sore throat, new olfactory and taste disorder(s), OR
- The participant must have experienced at least ONE of the following respiratory signs/symptoms: cough, shortness of breath or difficulty breathing, OR clinical or radiographical evidence of pneumonia; AND
- The participant must have at least one nasopharyngeal (NP) swab or saliva sample (or respiratory sample, if hospitalized) positive for SARS-CoV-2 by RT-PCR.

3.1.2. Safety Endpoints for Primary Safety Objective

The primary safety objective will be evaluated by the following safety endpoints:

- Solicited local and systemic adverse reactions (ARs) through 7 days after each dose of IP (Part A: Blinded Phase only).
- Unsolicited adverse events (AEs) through 28 days after each dose of IP (Part A: Blinded Phase only).
- Medically-attended AEs (MAAEs) or AEs leading to withdrawal through the entire study period.
- Serious AEs (SAEs) throughout the entire study period.

3.2. Secondary Endpoints

3.2.1. Secondary Efficacy Endpoints (Part A: Blinded Phase Only)

The secondary efficacy endpoints in Part A have been analyzed and demonstrated consistent vaccine efficacy of mRNA-1273, please refer to SAP Version 2.0 and Blinded phase (Part A) CSR. The secondary efficacy objectives/endpoints in Part A included:

- The VE of mRNA-1273 to prevent severe COVID-19, defined as first occurrence of COVID-19 starting 14 days after the second dose of IP, (as per the primary endpoint) AND any of the following:
  - Clinical signs indicative of severe systemic illness, Respiratory Rates ≥ 30 per minute, Heart Rate ≥ 125 beats per minute, $SpO_2 \leq 93\%$ on room air at sea level or $PaO_2/FIO_2 < 300$ mm Hg, OR
  - Respiratory failure or Acute Respiratory Distress Syndrome (ARDS), (defined as needing high-flow oxygen, non-invasive or mechanical ventilation, or ECMO), evidence of shock (systolic blood pressure < 90 mmHg, diastolic BP < 60 mmHg or requiring vasopressors), OR
  - Significant acute renal, hepatic or neurologic dysfunction, OR
  - Admission to an intensive care unit or death.
- The VE of mRNA-1273 to prevent the first occurrence of either COVID-19 or SARS-CoV-2 infection starting 14 days after the second injection of IP. This endpoint is a combination of COVID-19, defined as for the primary endpoint, and asymptomatic SARS-CoV-2 infection, determined by seroconversion assessed by bAb levels against SARS-CoV-2 as measured by a ligand-binding assay specific to the SARS-CoV-2 nucleocapsid protein and with a negative NP swab sample for SARS-CoV-2 at Day 1 .
- The VE of mRNA-1273 to prevent the secondary case definition of COVID-19 starting 14 days after the second injection of IP. The secondary case definition of

COVID-19 is defined as at least one of the following systemic symptoms: fever (temperature ≥ 38ºC), or chills, cough, shortness of breath or difficulty breathing, fatigue, muscle aches or body aches, headache, new loss of taste or smell, sore throat, nasal congestion or rhinorrhea, nausea or vomiting, or diarrhea AND a positive NP swab, nasal swab, or saliva sample (or respiratory sample, if hospitalized) for SARS-CoV-2 by RT-PCR.

- The VE of mRNA-1273 to prevent death due to a cause directly attributed to a complication of COVID-19, starting 14 days after the second IP dose.
- The VE of mRNA-1273 to prevent the first occurrence of COVID-19 starting 14 days after the first dose of IP.
- The VE of mRNA-1273 to prevent the first occurrence of COVID-19 starting 14 days after the second dose of IP regardless of evidence of prior SARS-CoV-2 infection determined by serologic titer against SARS-CoV-2 nucleocapsid or RT-PCR at baseline.
- The VE to prevent the first occurrence of SARS-CoV-2 infection in the absence of symptoms defining COVID-19 starting 14 days after the second IP dose. SARS-CoV-2 infection will be determined by seroconversion assessed by bAb levels against SARS-CoV-2 as measured by a ligand-binding assay specific to the SARS-CoV-2 nucleocapsid protein and with a negative NP swab sample for SARS-CoV-2 at Day 1.

3.2.2. Secondary Immunogenicity Endpoints

The secondary immunogenicity objective (Part A), to evaluate the immunogenicity of 2 doses of mRNA-1273 given 28 days apart, have been evaluated and summarized in the Part A CSR (dated 03 Aug 2021). Immune response to mRNA-1273 have been evaluated using binding and neutralizing antibodies specific to the SARS-CoV-2 proteins at baseline (pre-vaccination), 1 month after Dose 1 (Day 29) and 1 month after Dose 2 (Day 57) in a pre-select stratified random subcohort of participants including the following antigens:

- Serum bAb levels against SARS-CoV-2 as measured by ligand-binding assay specific to the SARS-CoV-2 S protein (MSD).
- Serum nAb titer against SARS-CoV-2 as measured by pseudovirus neutralization assays (PsVNA).

Blood samples for immunogenicity assessments continue as planned at the time points indicated in the SoEs of the protocol.

The immunogenicity endpoints (Part A) includes:

- Geometric mean titer (GMT) of SARS-CoV-2-specific binding and neutralizing antibodies(bAb and nAb)
- Geometric mean fold rise (GMFR) of SARS-CoV-2-specific bAb and nAb relative to Day 1.
- Seroresponse rate as compared to baseline (pre-vaccination/pre-Dose 1)

The following secondary immunogenicity endpoints at the specified timepoint are specifically for Part C immunogenicity analyses:

- GMT of post-booster antibodies at BD-Day 29 as compared to post-primary series mRNA-1273 at Day 57
- Seroresponse rate of booster from pre-Dose 1 of mRNA-1273 primary series at post-booster BD-Day 29 from Pre-vaccination/Pre-Dose 1 of the primary series as compared to post-Dose 2 (at Day 57) from Pre-vaccination/Pre-Dose 1 of the primary series in recipients of mRNA-1273.
  - Seroresponse is defined as at least 4-foldrise from pre-Dose 1 of mRNA-1273 primary series, for those below the LLOQ to ≥ 4 × LLOQ
  - Additional definitions of Seroresponse of a booster may be used and the details are provided in Section 5.5.2.1.1

Given the emergence of new SARS-CoV-2 variants in multiple waves, immune response of mRNA-1273 as measured by nAb and/or bAb against select variant strains (of interest)

would be of interest. If these data (nAb and/or bAb against variant strains) are available, respective endpoints similar to those listed above against prototype strains will be assessed.

3.3. Endpoints to assess effectiveness of mRNA-1273

Effectiveness of the primary series and a booster (Part C) will be evaluated by the following endpoints with details specified in section 5.3:

- COVID-19
- severe COVID-19
- COVID-19 or SARS-CoV-2 infection, serologically confirmed SARS-CoV-2 infection or COVID-19 regardless of symptomatology or severity
- COVID-19 based on a secondary (less restrictive) definition of COVID-19
- death caused by COVID-19
- Asymptomatic SARS-CoV-2 infection

3.4. Exploratory Endpoints

The following exploratory objective/endpoints for Part A have been evaluated and results have been presented in the final blinded phase CSR (dated 03 Aug 2021) or have been included in accepted/published articles:

- VE of mRNA-1273 to reduce duration and presence/severity of COVID-19 symptoms
- VE of mRNA-1273 against Burden of Disease
- VE of mRNA-1273 against Burden of Infection
- VE of mRNA-1273 against all-cause mortality
- Effect of mRNA-1273 against viral load (Pajon, 2021)
- Preliminary analysis of immune response markers as correlates of risk has been performed and the results are published (Gilbert, 2021)

3.5. Overall Study Design

This is a 3-part Phase 3 study: Part A, Part B and Part C. Participants in Part A, the Blinded Phase of this study are blinded to their treatment assignment. Given that the primary efficacy endpoint for mRNA-1273 against COVID-19 was met per the protocol-defined interim analysis, Part B, the Open-Label (OL) Observational Phase of this study is designed to offer participants who received placebo in Part A of this study and who meet EUA eligibility, an option to request open-label mRNA-1273 (Figure 1). Part C, the booster dose (BD) Phase, is designed to offer eligible participants the option to receive a booster dose of mRNA-1273.

Study design of Part A is described in protocol section 4.1.1 and SAP Version 2.0 section 4.1.1 .

3.5.1. Part B, the Open-Label Observational Phase

All participants will proceed to Part B, the Open-Label Observational Phase of the study, starting with a Participant Decision clinic visit (PDV) (Figure 2). Participants are given the option to be unblinded to their original treatment. A NP swab for RT-PCR for SARS-CoV-2 and a blood sample for serology are collected prior to unblinding.

Participants who are unblinded, only received placebo in the blinded phase, are EUA eligible, and request to receive mRNA-1273 will proceed to receive two doses of mRNA-1273 in Part B (Table 21 supplemental scheduled of event in the protocol).

Participants who are unblinded and received only one dose of mRNA-1273 in the blinded phase, are EUA eligible, and request to receive mRNA-1273 will proceed to receive one dose of mRNA-1273 in Part B (Table 22 modified supplemental scheduled of event in the protocol).

All participants remain on their original schedule of event to complete 24 months of follow-up after the second injection of IP; study participants who enter the Supplemental or Modified Supplemental schedule of event as described above do so in addition to their

original schedule of event. Accordingly, all study participants will complete the full study follow-up to 24 months after the second dose of their original inoculation (mRNA-1273 or placebo).

Solicited adverse reactions (ARs) after each injection of mRNA-1273 are not collected in the open-label phase. Participants will use an electronic diary (eDiary) to prompt an unscheduled clinic visit if experiencing any symptoms of COVID-19. Surveillance for COVID-19 will be performed through weekly contacts with the participants via a combination of telephone calls and completion of an eDiary starting at Day 1 through the end of the study. An illness visit will be scheduled if the participant is experiencing COVID-19 symptoms.

3.5.2. Part C, the Booster Dose Phase

All participants will be asked to schedule BD-1 (BD Day 1) visit as the start of Part C, the Booster Dose Phase. Eligible participants will be provided an option to receive BD consisting of a single 50 µg dose of mRNA-1273.

After the BD 1 visit, participants who do not receive a BD will continue with the Part C schedule of events with the exception of the BD-1a (BD Day 4) visit and blood draw and the safety calls (Table 23 in the protocol).

At the BD 1 visit, participants who request a BD, are eligible and have no contraindications to further dosing (Section 7.1 in the protocol) will have subsequent study site visits and complete scheduled activities (subject to investigational vaccine availability) according to the Part C schedule of events (Table 23 in the protocol).

Figure 1 Study Flow Diagram: Part A, the Blinded Phase followed by Part B, the Open-Label Phase Including Part C, the Booster Dose Phase

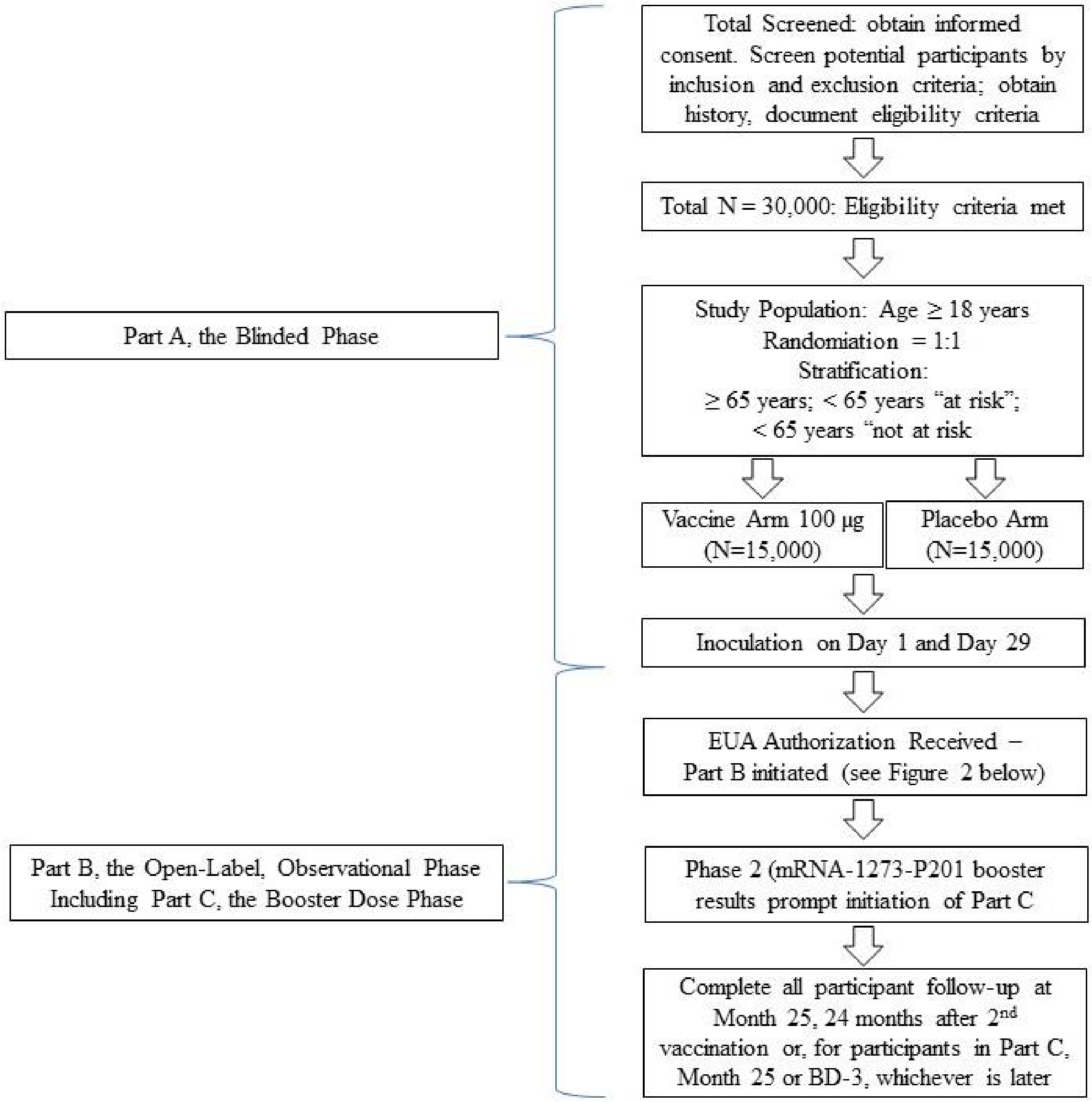

Abbreviations: BD = booster dose visit; EUA = emergency use authorization

Figure 2 Part B, the Open-Label Observational Phase, and Part C, the Booster Dose Phase

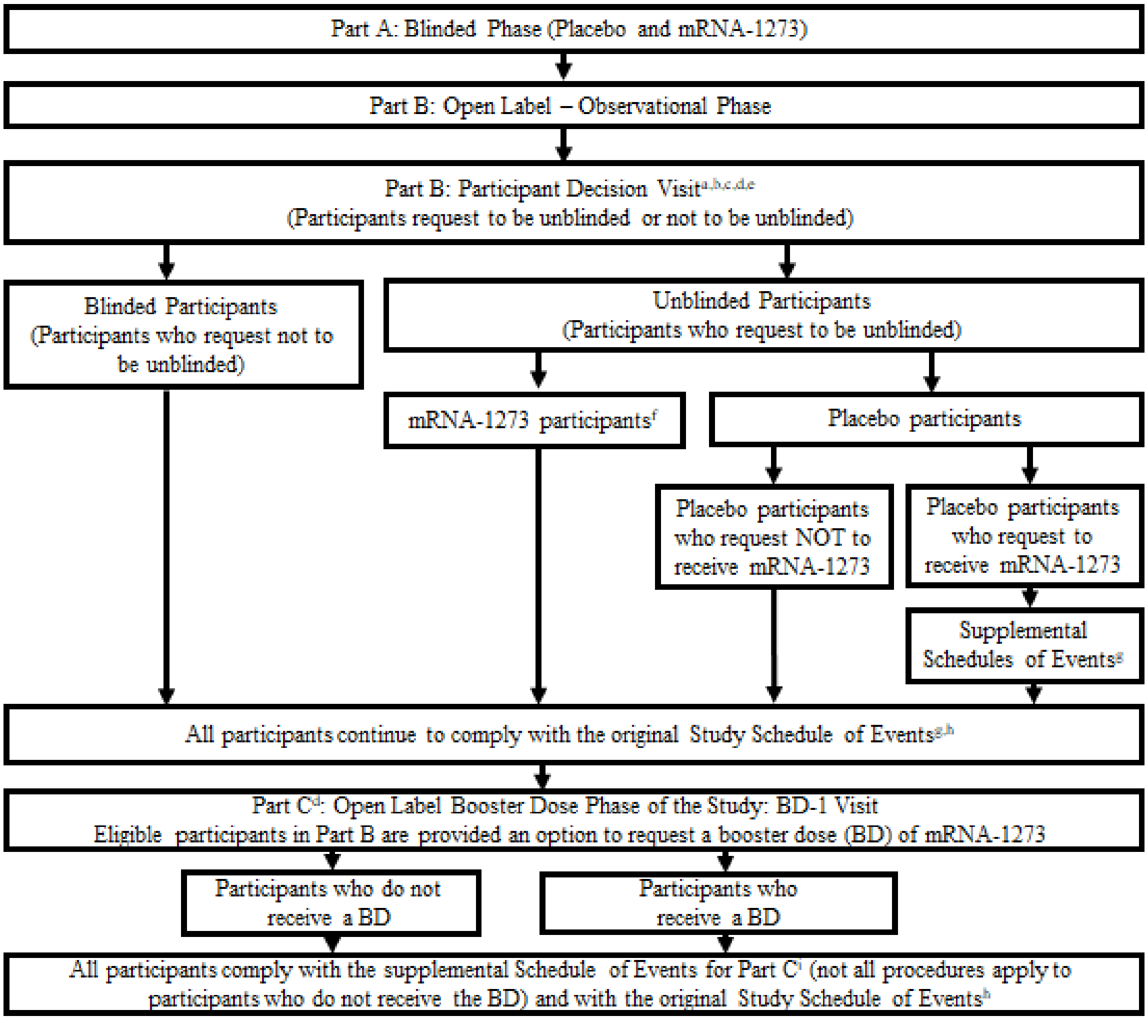

a All participants are encouraged to remain in the study.
b All participants are given the option to be unblinded to treatment received in Part A: Blinded Phase.
c All participants are counselled about the importance of continuing other public health measures to limit the spread
of disease including physical-social distancing, wearing a mask, and hand-washing.
d All participants sign a revised informed consent form.
e All participants consent to provide a nasopharyngeal swab and a blood sample for immunologic analysis.
f mRNA-1273 recipients who only received 1 dose of the mRNA-1273 vaccine will receive the second dose of mRNA-1273 vaccine.
g Placebo recipients who request to receive mRNA-1273 and meet eligibility will comply with a Supplemental Schedule of Events in addition to the Original Study Schedule of Events included in the Clinical Study Protocol.
h Original Study Schedule of Events are included in the Clinical Study Protocol

i Procedures required or not required based on receipt of the booster dose are clarified in the Part C Supplemental Schedule of Events included in the Clinical Study Protocol

3.6. Statistical Hypothesis (Part A)

For the primary efficacy objective of the blinded phase (Part A), the null hypothesis of this study is the VE of mRNA-1273 to prevent first occurrence of COVID-19 is ≤ 30%, i.e. $H_0^{efficacy}$: VE ≤ 0.3.

The primary efficacy objective has been demonstrated based on pre-specified success criterion at the interim analysis based on 95 adjudicated cases (data snapshot 11 Nov 2020).

The primary analysis (data snapshot 25 Nov 2020) and the final analysis of the blinded phase (DBL 04 May 2021) have consistent results on vaccine efficacy, confirming persistent, high efficacy over the blinded phase.

3.7. Sample Size and Power

Please refer to the protocol and SAP Version 2.0 for details.

3.8. Randomization

Please refer to the protocol and SAP Version 2.0 for details.

3.9. Blinding and Unblinding

Part A of this study is an observer-blind part. The investigator, study staff, study participants, site monitors, and Sponsor personnel (or its designees) were blinded to the IP administered until unblinding in Part B, with the certain exceptions specified in Section 6.2.8 of the protocol. Refer to SAP Version 2.0 for more details.

Part B and Part C of the study is open-label.

4. Analysis Populations

The following analysis sets have been defined in SAP Version 2.0. Refer to SAP Version 2.0 for details.

- Randomization Set
- Full Analysis Set (FAS)
- Modified Intent-to-Treat (mITT) Set
- Per-protocol (PP) Set
- Per-Protocol Random Subcohort for Immunogenicity (PPRSI)
- Safety Set
- Solicited Safety Set

The following analysis sets are defined in this SAP for Part C:

- Part C Safety Set
- Part C Per-Protocol Random Subcohort for Immunogenicity (Part C PPRSI)
- Part C Per-Protocol Immunogenicity Subset (Part C PPIS)
- Part C Per-Protocol Immunogenicity Subset – Pre-booster SARS-CoV-2 negative (Part C PPIS-Neg)
- Part C Per-Protocol Set (Part C PPS)

The following analysis sets are defined in this SAP for Primary Series (for those randomized to mRNA-1273 in Part A and those crossover to mRNA-1273 in Part B):

- Per Protocol Primary Series Set (PP Primary Series)
- Primary Series Safety Set

4.1. Part C Safety Set

The Part C Safety Set consists of participants who were randomized in Part A and received mRNA-1273 50 ug booster in Part C. The Part C Safety Set will be used for all analysis of safety in Part C.

4.2. Part C Per-Protocol Random Subcohort for Immunogenicity (Part C PPRSI)

The Part C PPRSI consists of participants who were in PPRSI in Part A defined in SAP Version 2.0, received booster in Part C, and did not have major PDs after Part A that impact major or critical data. Participants who did not receive 2 doses of the primary series of mRNA-1273, or did not receive Dose 2 of mRNA-1273 within [21, 42] days after Dose 1 would be excluded from Part C PPRSI.

Part C PPRSI may be used to summarize immune response after booster in Part C.

4.3. Part C Per-Protocol Immunogenicity Subset (Part C PPIS)

The Part C PPIS consists of participants who were in PPRSI in Part A (defined in SAP Version 2.0), randomized to mRNA-1273 in Part A, were baseline/pre-Day 1 SARS-CoV-2 negative and received booster in Part C.

Part C PPIS is a subset of PPRSI in Part A, and a subset of Part C PPRSI. Part C PPIS will be used to summarize immune response after booster in Part C

4.4. Part C Per-Protocol Immunogenicity Subset – Pre-booster SARS-CoV-2 Negative (Part C PPIS-Neg)

The Part C PPIS-Neg consists of participants who are in Part C PPIS and are pre-booster SARS-CoV-2 negative, defined as no virologic or serologic evidence of SARS-CoV-2 infection on or before BD-Day 1 (pre-booster), i.e. RT-PCR result is not positive if available at BD-Day 1 and a negative bAb specific to SARS-CoV-2 nucleocapsid (as measured by Roche Elecsys Anti-SARS-CoV-2 assay) on or before BD-Day 1.

Part C PPIS-Neg will be used for analysis of immune response to booster, especially, to be compared with immune response after primary series of mRNA-1273 to infer efficacy of a booster.

4.5. Part C Per-Protocol (PP) Set

The Part C Per-Protocol Set (Part C PPS) consists of all participants who were randomized in Part A, received mRNA-1273 50 ug booster in Part C after primary series per schedule, pre-booster SARS-CoV-2 negative, and have no major protocol deviations that impact critical or key data.

Participants who did not receive the treatment as randomized in Part A, did not receive 2 doses of the primary series of mRNA-1273, or did not receive Dose 2 of mRNA-1273 within [21, 42] days after Dose 1 will be excluded from the Part C Per-Protocol Set.

Part C PPS will be used for analysis of effectiveness of booster.

4.6. Per-Protocol (PP) Primary Series Set

The PP Primary Series Set consists of all participants in Per-Protocol Set who received two doses of mRNA-1273 primary series per schedule in Part A or Part B, with negative pre-Dose 1 SARS-CoV-2 status, and had no major protocol deviations that impact critical or key study data.
Participants who did not receive the treatment as randomized in Part A, did not receive 2 doses of the primary series of mRNA-1273, or did not receive Dose 2 of mRNA-1273 within [21, 42] days after Dose 1 of mRNA-1273 will be excluded from the PP Primary Series Set.

PP Primary Series Set will be used for the effectiveness analysis of the mRNA-1273 primary series. PP Primary Series Set will also be used to assess effectiveness of participants received booster dose and those who did not receive booster dose by pre-booster SARS-CoV-2 status.

4.7. Primary Series Safety Set

The Primary Series Safety Set consists of all participants who were randomized in Part A and received at least one dose of mRNA-1273 primary series. The Primary Series Safety Set will be used for the safety analysis of the mRNA-1273 primary series.

5. Statistical Analysis

5.1. General Considerations

5.1.1. General Considerations, Derivations and Strategy for incomplete/missing data

The Schedule of Events is provided in the protocol Table 16-23.

Continuous variables will be summarized using the following descriptive summary statistics: the number of subjects (n), mean, standard deviation (SD), median, minimum (min), and maximum (max).

Categorical variables will be summarized using counts and percentages.

Age: unless otherwise specified, age is calculated as the age at screening and will be used for derivation of age group.

Study day relative to an injection will be calculated as date of assessment/event – date of the most recent injection prior to the assessment/event + 1, unless otherwise specified.

Unscheduled visits: measurements at Unscheduled visits will be included in analysis as follows:

- In scheduled visit windows per specified visit windowing rules.
- In the derivation of baseline measurements.
- In the derivation of maximum/minimum values after 1st dose and maximum/minimum change from baseline values for safety analyses.
- In individual subject data listings as appropriate.

Visit windowing rules: The analysis visit windows for protocol-defined visits are provided in Appendix B.

Incomplete/missing data:

- Imputation rules for missing dates of prior/concomitant medications, non-study vaccinations and procedures are provided in Appendix C.
- Imputation rules for missing AE dates are provided in Appendix D.
- If the laboratory results are reported as below the LLOQ (e.g. < 0.1), the numeric values will be substituted by 0.5 × LLOQ in the summary when treating the results as continuous variables. If the laboratory results are reported as greater than the ULOQ (e.g. > 3000) without actual value, the numeric values will be substituted by ULOQ in the summary statistics for continuous variable.
- Other incomplete/missing data will not be imputed, unless specified otherwise.

5.1.2. Baseline (Pre-Dose 1) in Part A, Unblinding/PDV in Part B, Pre-booster in Part C and pre-Vaccination/pre-Dose 1 of mRNA-1273

Baseline (Pre-Dose 1), unless specified otherwise, is defined as the most recent non-missing measurement (scheduled or unscheduled) collected before the first dose in Part A.

For analyses of Part B (OL phase), please also refer to Section 6.1 of SAP Version 2.0.

For analyses of primary series of mRNA-1273 (Part A and Part B), first dose of mRNA-1273, either in Part A for those randomized to mRNA-1273 in Part A, or in Part B for those who were randomized to Placebo in Part A and unblinded and received mRNA-1273 in Part B, will be used as reference of "baseline", and will be referred to as **Pre-vaccination**, or, **Pre-Dose 1 of mRNA-1273**.

For analyses of booster (Part C), the booster dose of mRNA-1273 in the BD phase will be used as reference for relevant analyses, and will be referred to as **Pre-booster**.

For the summary statistics of all numerical variables unless otherwise specified, the display precision will follow programming standards. Please see Appendix A for variable display standards.

When count data are presented, the percentage will be suppressed when the count is zero in order to draw attention to the non-zero counts. A row denoted "Missing" will be included in count tabulations where specified on the shells to account for dropouts and missing values.

5.1.3. SARS-CoV-2 status

Baseline SARS-CoV-2 status was the baseline status in Part A, and was determined by virologic and serologic evidence of SARS-CoV-2 infection on or before Day 1 (pre-Dose 1) in Part A, and is defined in SAP Version 2.0.

Positive SARS-CoV-2 status at Baseline in Part A is defined as a positive RT-PCR test for SARS-CoV-2, and/or a positive serology test based on bAb specific to SARS-CoV-2 nucleocapsid (as measured by Roche Elecsys Anti-SARS-CoV-2 assay) on or before Day 1 in Part A.

Negative status at Baseline in Part A is defined as a negative RT-PCR test for SARS-CoV-2 and a negative serology test (as measured by Roche Elecsys Anti-SARS-CoV-2 assay) on or before Day 1 in Part A.

The baseline SARS-CoV-2 status in Part A is defined as missing for subjects with both tests missing, or with one test missing and one test negative.

Subjects with negative Baseline SARS-CoV-2 status will be included in the mITT population; subjects with positive or missing baseline SARS-CoV-2 status will be excluded from the mITT and PP population.

SARS-CoV-2 status at unblinding/PDV is the SARS-CoV-2 status at the start of Part B, determined by virologic and serologic evidence of SARS-CoV-2 infection at unblinding/PDV in Part B.

Positive SARS-CoV-2 status is defined as a positive RT-PCR test for SARS-CoV-2, and/or a positive serology test based on bAb specific to SARS-CoV-2 nucleocapsid (as measured by Roche Elecsys Anti-SARS-CoV-2 assay); negative SARS-CoV-2 status is defined as both negative RT-PCR and negative Elecsys at unblinding/PDV at Part B. If one test is negative and one test is missing, SARS-CoV-2 status would be missing at unblinding /PDV.

Pre-booster SARS-CoV-2 status for Part C, is determined by using virologic and serologic evidence of SARS-CoV-2 infection prior to the booster in Part C.

Positive pre-booster SARS-CoV-2 status is defined as a positive RT-PCR test for SARS-CoV-2, and/or a positive serology test based on bAb specific to SARS-CoV-2 nucleocapsid (as measured by Roche Elecsys Anti-SARS-CoV-2 assay) on or before the date of booster (BD-Day 1).

Negative pre-booster SARS-CoV-2 status is defined as no virologic or serologic evidence of SARS-CoV-2 infection on or before BD-Day 1 (pre-booster), i.e. RT-PCR result is not positive if available at BD-Day 1 and a negative bAb specific to SARS-CoV-2 nucleocapsid (as measured by Roche Elecsys Anti-SARS-CoV-2 assay) on or before BD-Day 1.

The pre-booster SARS-CoV-2 status is defined as missing for subjects with both tests missing, or no Elecsys results on or before BD-Day 1 and RT-PCR not positive at pre-booster (at BD-Day 1). Please note the NP Swab at BD-Day 1 is optional, which is a limitation to detect the infections occurred prior to Part C.

Subjects with positive pre-booster SARS-CoV-2 status will be excluded from the Part C PP Set and Part C PPIS-Neg.

5.1.4. Analysis Strategy and Treatment Groups

Analyses Strategy: The following analyses may be performed by using Part B and Part C data. Please also refer to "Analysis Periods" subsection (Section 5.1.5) in this section. The analysis periods are summarized in Table 1 of Section 5.1.5.

- To assess effectiveness and safety during Part B (OL-phase period), analyses may be conducted using the analysis periods and treatment groups for Efficacy and Safety of Part B based on data collected in Part B.
- To assess the effectiveness and safety after booster (Part C), Part C analyses will be conducted using the analysis periods and treatment groups for Effectiveness and Safety of Part C based on data collected during the booster dose phase (Part C).
- To assess effectiveness of the primary series of mRNA-1273, analyses of effectiveness may be conducted using analysis periods and treatment groups for efficacy using data collected in Part A and Part B. Please note only those who received 2 doses of mRNA-1273 would be included in the analyses.
- To assess effectiveness of a booster, and long term effectiveness (or durability) of mRNA-1273 primary series, analysis of effectiveness may be conducted using analysis periods and treatment groups for efficacy using data collected during the blinded phase (Part A), the open-label phase (Part B), and the booster dose phase (part C).

Treatment cohorts/groups

The following treatment groups have been used in analysis of blinded phase (Part A):

- mRNA-1273
- Placebo

The following treatment groups have been and will be used in analysis of Part B:

- mRNA-1273
- Placebo-mRNA-1273
- Placebo

The above 3 treatment groups may also be used to assess long term effectiveness of mRNA-1273 (Part A and Part B).

If a participant requested to receive mRNA-1273 in Part B (OL phase), the subject will be included to mRNA-1273 cohort as the planned treatment group for efficacy analysis, regardless whether the dose was actually received or not. Participants who were randomized to placebo and planned to receive mRNA-1273 in the open-label phase will be included to the Placebo-mRNA-1273 Cohort for efficacy analysis. Participants who only received placebo in the blinded phase and received mRNA-1273 in the open-label phase will be included in the Placebo-mRNA-1273 Cohort for safety analysis and efficacy analysis for Part B.

The following treatment groups will be used in analysis of Part C:

- Booster 50 µg after mRNA-1273 Primary Series
- Booster 50 µg after Placebo-mRNA-1273 Primary Series

In addition, an overall/total booster group in Part C may be presented in the summaries and analysis of Part C.

The following treatment groups will be used in analyses of mRNA-1273 primary series:

- mRNA-1273
- Placebo-mRNA-1273

All analyses and data summaries/display for efficacy will be provided by corresponding treatment cohorts using appropriate analysis population unless otherwise specified.

5.1.5. Analysis Periods

Analysis periods and treatment groups for analysis of Part B, Part C, and Primary Series are summarized in Table 1. For effectiveness, participants will be included in treatment groups based on ‘as-planned’ or ‘intent-to-treat’ assignments; for safety, participants will be included in treatment groups based on ‘as treated’ groups (treatment actually received), details are provided to Appendix F. For analysis of immunogenicity, ‘per-protocol’ sets will be used as analysis population and ‘per-protocol’ sets require participants receiving planned treatments.

Table 1 Analysis Periods for Analyses of Part B, Part C, and Primary Series

Treatment Group/Cohort	Description	Analysis Period
**Part B (Effectiveness)**		
mRNA-1273	Participants randomized to mRNA-1273 in the Blinded Phase<br>Date of reference: PDV/unblinding	From PDV/unblinding to earlier date of (BD-1 visit, DBL or data cutoff date for efficacy, last observed in study [a])
Placebo	Participants randomized to Placebo in the Blinded Phase and did not request to receive mRNA-1273 in Part B<br>Date of reference: Dose 1 of mRNA-1273	
Placebo-mRNA-1273	Participants randomized to Placebo in the Blinded phase and requested to receive mRNA-1273 in Part B<br>Date of reference: Dose 1 of mRNA-1273 in Part B	
**Part B (Safety)**		
mRNA-1273	Participants received at least one dose of mRNA-1273 in the Blinded Phase	From PDV/unblinding or 1st dose of mRNA-1273 in Part B to earlier date of (BD- Day 1, DBL or data cutoff date for safety, last observed in study [a]).
Placebo	Participants only received Placebo in the Blinded Phase and did not receive mRNA-1273 in Part B	
Placebo-mRNA-1273	Participants only received Placebo in the Blinded phase and received mRNA-1273 in Part B	
**Part C (Effectiveness, Immunogenicity, Safety)**		
Booster 50 µg after mRNA-1273 primary series	Participants randomized to/received mRNA-1273 in the Blinded Phase and received booster 50 µg in Part C<br>Date of reference: date of booster	From dose date in Part C to earlier date of (DBL or data cutoff date for safety, last observed in study [a]).

Booster 50 µg after Placebo-mRNA-1273 Primary Series	Participants randomized to /only received Placebo in the Blinded Phase and received at least one dose of mRNA-1273 in Part B and received booster 50 µg in Part C<br>Date of reference: date of booster	
Booster 50 µg	Participants randomized in the blinded phase and received booster 50 µg in Part C<br>Date of reference: date of booster	From dose date in Part C to earlier date of (DBL or data cutoff date for safety, last observed in study [a]).
Not received booster	Participants randomized to/received mRNA-1273 in Part A or B and did not receive booster in Part C<br>Date of reference: date of BD-visit 1	Up to earlier date of (DBL or data cutoff date for safety, last observed in study [a]).
Placebo	Participants randomized to/only received Placebo in Part A or B and did not receive booster in Part C<br>Date of reference: date of BD-visit 1	From BD-visit 1 in Part C to earlier date of (DBL or data cutoff date for safety, last observed in study [a]).
**Effectiveness, Immunogenicity of mRNA-1273 primary Series**		
mRNA-1273	Participants randomized to mRNA-1273 in the Blinded Phase	From Dose 1 of mRNA-1273 to earlier date of (booster date, DBL or data cutoff date, last observed in study [a]).
Placebo-mRNA-1273	Participants randomized to Placebo and received mRNA-1273 in Part B	
**Safety of mRNA-1273 primary Series**		
mRNA-1273	Participants received mRNA-1273 in the Blinded Phase	From Dose 1 of mRNA-1273 to earlier date of (booster date, DBL or data cutoff date, last observed in study [a]).
Placebo-mRNA-1273	Participants received Placebo in the Blinded phase and received mRNA-1273 in Part B	

[a] last observed in study is the earliest date of study discontinuation, study completion, or death.

All analyses will be conducted using SAS Version 9.4 or higher unless otherwise specified.

5.2. Background Characteristics

5.2.1. Subject Disposition

The number and percentage of subjects (analysis sets defined in Section 5) will be summarized by treatment cohort.

The number and percentage of subjects in each of the following disposition categories will be summarized for Part B:

- Received each dose of IP in the blinded phase
- Unblinded in the open-label phase
- Received each dose of mRNA-1273 in the open-label phase
- Prematurely discontinued study IP and the reason for discontinuation
- Prematurely discontinued the study and the reason for discontinuation

The number and percentage of subjects in each of the following disposition categories will be summarized for Part C based on the Part C Safety Set:

- Received booster dose in the BD phase
- Completed study
- Prematurely discontinued the study and the reason for discontinuation

5.2.2. Demographics and Baseline Characteristics

Descriptive statistics will be calculated for the following continuous demographic and baseline characteristics: age (years), weight (kg), height (cm), body mass index (BMI) (kg/m^2). The number and percentage of subjects will be provided for categorical variables such as age group, sex, race, ethnicity, randomization stratum under which the subject was randomized, and risk factors at Screening (have at least one of the following):

- Chronic lung disease (e.g., emphysema and chronic bronchitis, idiopathic pulmonary fibrosis, and cystic fibrosis) or moderate to severe asthma
- Significant cardiac disease (e.g., heart failure, coronary artery disease, congenital heart disease, cardiomyopathies, and pulmonary hypertension)
- Severe obesity (body mass index ≥ 40 kg/m^2)
- Diabetes (Type 1, Type 2 or gestational)
- Liver disease
- Human Immunodeficiency Virus (HIV) infection

The summaries will be provided by treatment cohort for Safety Set and Part C Safety Set.

5.2.3. Medical History

Medical history data will be coded by system organ class (SOC) and preferred term (PT) using the Medical Dictionary for Regulatory Activities (MedDRA).

The number and percentage of participants with any medical history will be summarized by SOC, PT, and treatment. A participant will be counted only once for multiple events within each SOC and PT. SOC will be displayed in internationally agreed order.

5.2.4. Prior and Concomitant Medications

Prior and concomitant medications and non-study vaccinations will be coded using the WHO drug dictionary (WHODD). The summary of concomitant medications will be based on the Safety Set. Categorization of prior, concomitant, and post medications for Part B and Part C is summarized in Appendix C.

Concomitant Medications for Part C will be summarized in Part C Safety Set.

5.2.5. Study Exposure

For Part B, the number and percentage of subjects receiving 1st and 2nd dose of mRNA-1273 in Part B, the timing of the 2nd dose will be summarized by treatment cohort for Part B using the Safety Set.
For Part C, the number and percentage of subjects receiving booster dose will be summarized by treatment cohort for Part C using Part C Safety Set.
The summary of time between the 2 doses of primary series, time between the primary series and booster dose, and follow up time after booster dose will be provided.

5.2.6. Major Protocol Deviations

Major protocol deviations are a subset of protocol deviations that may significantly impact the completeness, accuracy, or reliability of the study data or that may significantly affect a subject's rights, safety, or well-being. Major protocol deviations rules will be developed and finalized before database lock.

The number and percentage of the subjects with each major protocol deviation type in Part B and Part C will be provided.

Select major protocol deviations impact critical or key study data, and subjects with such deviations will be excluded from the Per-Protocol sets as defined in Section 5 of this SAP. Such major protocol deviations will be determined and documented by Sponsor prior to DBL. Reasons of exclusion from Per-Protocol Set for efficacy will be summarized.

5.3. Effectiveness Analysis

For Part B, PP Set is the primary analysis population for Part B analysis of effectiveness.

For Part C, the Part C PP Set is the primary analysis population for Part C analysis of vaccine effectiveness, unless otherwise specified. Participants will be included in the treatment cohort for effectiveness analysis as specified in Section 5.1.

Surveillance for COVID-19 will be performed starting after enrollment through the end of the study.

An Adjudication Committee (AC) will review potential cases and adjudicate COVID-19 and severe COVID-19.

5.3.1. Analysis of the Primary Efficacy Endpoint for Part B, Part C, and Long-term Analyses

5.3.1.1. Primary Efficacy Endpoint Definition/Derivation

Vaccine efficacy has been demonstrated based on the primary efficacy endpoint in the blinded phase (Part A) of first occurrence of COVID-19 starting 14 days after the 2nd injection in the blinded phase (Part A).

Part B and Part C are open-label and participants are offered an opportunity to receive the primary series and booster of mRNA-1273 respectively.

Throughout the study, COVID-19 is assessed in the same way as the primary efficacy endpoint in the blinded phase (Part A). An independent external Adjudication Committee (AC) have been adjudicating potential COVID-19 and severe COVID-19 cases. COVID-19 based on AC assessments will be used as the primary approach for COVID-19 throughout the study. COVID-19 cases based on the protocol definition requiring at least 2 eligible systemic symptoms or 1 respiratory symptom after the primary series, and after the booster will also be evaluated.

Derivation of COVID-19 cases based on RT-PCR test results and symptoms is the same as COVID-19 cases for the primary efficacy endpoint as specified in Section 8.1 of the protocol and Section 6.3.1.1 of SAP Version 2.0.

In the Par B open-label phase of the study, all participants will have a scheduled NP swab test and blood collected for immunologic analysis at the PDV. Participants who received mRNA-1273 in the open-label phase will have an additional blood sample collected at OL Day 29 or OL Day 57, depending on the planned number of mRNA-1273 doses to be received in the OL phase. For Part B analysis and analysis of the primary series, subjects with early infection, as detected by positive RT-PCR at scheduled visits (including Day 29 and PDV), or positive bAb against SARS-CoV-2 nucleocapsid protein at scheduled visits,

will be censored at the time of early infection. If a participant had eligible symptom(s) within 14 days of positive RT-PCR at scheduled visits, the participant is considered to be a case for COVID-19.

In the Part C booster dose phase of the study, all participants will have scheduled blood sample collected at BD Day 1 visit. NP swab test at this visit is optional. Participants who received the booster dose of mRNA-1273 in Part C will have blood sample collected at BD Day 29 and BD Day 181 (6 months post-booster). For Part C, subjects with early infection, as detected by positive RT-PCR at scheduled visits, or positive bAb against SARS-CoV-2 nucleocapsid protein at the scheduled visits, will be censored at the time of early infection. If a participant had eligible symptom(s) within 14 days of positive RT-PCR at scheduled visits, the participant is considered to be a case for COVID-19.

Subjects who have no documented COVID-19 will be censored at the earlier date of DBL or data cutoff date for efficacy, date of last observed in study defined as the earlier of study completion date, early discontinuation date from study, or date of death. For analysis of Part B or primary series, subjects who have no documented COVID-19 will be censored at the earlier date of date of booster, DBL or data cutoff date for efficacy, or date of last observed in study defined as the earlier of study completion date, early discontinuation date from study, or date of death.

5.3.1.2. Analysis approach for effectiveness of vaccine

COVID-19 will be evaluated using incidence rates. Number of events and incidence rate will be provided in a dynamic approach for each treatment cohort, e.g., a participant without COVID-19 or SARS-COV-2 infection will no longer be considered as “at risk” in the non-booster treatment group from the timepoint when this participant received the booster dose, instead, this participant will be considered as “at risk” in the booster treatment group from that timepoint. Incidence rate will be calculated as the number of

participants with an event divided by the number of subjects at risk adjusted by person-time (years) in each treatment cohort.

5.3.1.2.1. Effectiveness of the primary series

Effectiveness of the primary series of mRNA-1273 in those vaccinated participants, will be assessed in participants initially randomized to the mRNA-1273 group (mRNA-1273, vaccinated from July-December 2020, early vaccinated) and those initially randomized to the placebo group (Placebo-mRNA-1273, vaccinated December 2020-April 2021, recent vaccinated) using the PP Primary Series Set. Part A and Part B data after Dose 1 of mRNA-1273 will be included for this analysis, and date of Dose 1 of mRNA-1273 will be used as the reference. The analysis period will be from Dose 1 of mRNA-1273 to earlier of last study participation, booster date, or data cutoff date for efficacy as presented in Table 1.

Using PP Primary Series Set, participants at risk will be those who did not have evidence of prior infection prior to Dose 1 of mRNA-1273, specifically, those in mRNA-1273 (randomized to mRNA-1273 in Part A (baseline/pre-Day 1 SARS-CoV-2 negative is required for inclusion in PP Primary Series Set), and those in Placebo-mRNA-1273 group who were SARS-CoV-2 negative at PDV/unblinding at the start of Part B.

Incidence rates will be provided over the time from Dose 1 of mRNA-1273 to receipt of booster, last study participation, or data cutoff date for efficacy. Incidence rates will also be provided by calendar quarters, as well as by the following time intervals:

- After Dose 1
    - After Dose 1 of mRNA-1273
    - After Dose 1 to before Dose 2 of mRNA-1273
    - After Dose 2 of mRNA-1273
    - Dose 2 to <14 days after Dose 2
    - ≥14 days after Dose 2

The last time interval "≥14 days after Dose 2" of mRNA-1273 may be further partitioned into shorter intervals. For each interval, person-time is calculated for participants at risk at

the beginning of the interval, as the time from the date of the beginning of the time interval to the date of the event/censoring for subjects with an event/censor if the event/censor occurs in the interval; or the time of the interval for subjects who continue to be at risk at the end of the interval. Incidence rate, and 95% CI for incidence rate will be calculated using the exact method adjusting for person-time will be provided by treatment cohort. Number and incidence rates of COVID-19 starting 14 days after Dose 2 of mRNA-1273 will also be provided by calendar quarters.
Incidence rate will also be provided for the periods defined by emergence and circulation of select variant of interest, e.g. December 2021- data cutoff date (Apr, 2022).
Effectiveness in Part B will be performed, and PP Set will be used with treatment cohorts listed in Table 1 for Part B analysis with similar analysis approach. For Part B, participants at risk at the start of Part B are defined as participants who did not have COVID-19 or SARS-CoV-2 infection and non-positive SARS-CoV-2 status at unblinding/PDV prior to Part B.

5.3.1.2.1.1. Relative Effectiveness of recent vaccinated vs early vaccinated (the Primary Series of mRNA-1273)

Relative effectiveness of recent vaccinated participants (Placebo-mRNA-1273) vs. early vaccinated participants (mRNA-1273) will be assessed using the reduction in incidence rate, calculated as 1- incidence rate ratio (IRR). The 95% CI of reduction in IR will be calculated using the exact method (Poisson distribution) adjusting for person-time.

The above analysis may be performed by calendar quarters and/or time periods.

5.3.1.2.2. Effectiveness of the booster

For Part C, analysis of COVID-19 after booster, Part C PP Set will be used. For Part C, participants at risk at the start of Part C are defined as participants who did not have COVID-19 and had no SARS-CoV-2 infection prior to Part C , i.e. negative pre-booster SARS-CoV-2 status. Similarly, incidence rates after booster, as well as by calendar months (if feasible), and by time periods of the following time intervals will be provided:

- After Booster

- Booster to <14 days after booster
- ≥14 days after Booster

Person-time, incidence rate, and 95% CI for incidence rate based on the exact method adjusting for person-time will be provided by treatment cohort. Number and incidence rates of COVID-19 starting 14 days after Booster will also be provided by calendar months along with 2 additional time intervals associating with pre-omicron and omicron variant waves: 01-Sep-2021 to 30-Nov-2021 and 01-Dec-2021 to data cutoff date. The non-parametric Kaplan-Meier method will be used to estimate cumulative incidences of time to first occurrence of COVID-19 curve in each treatment cohort.

5.3.1.2.3. Effectiveness of booster (50 µg mRNA-1273)

The same analysis approach described above will be used to assess relative effectiveness of a booster based on adjudicated COVID-19 starting 14 days after booster using the Per-Protocol Set (Primary Series), participants in PP primary Series Set with no major PD in Part C that may affect critical data or were a known COVID-19 or infection case before Part C or not pre-booster SARS-CoV-2 negative will be excluded from this analysis. Participants received a booster after primary series will be compared with those who did not receive a booster after primary series (treatment group listed below). Number of cases, incidence rates and 95% CI will be provided for each treatment group, and reduction in IR (1-IRR) and its 95% CI will be provided to assess relative effectiveness of a booster after the primary series.

Booster 50 µg	In PP Set, Participants received primary series of mRNA-1273 per schedule in Part A or Part B and received booster 50 µg in Part C<br>Date of reference: date of booster
Not received booster	Participants randomized to/received mRNA-1273 in Part A or B and did not receive booster in Part C

5.3.1.3. Subgroup Analysis

Subgroup analyses of the efficacy endpoint of COVID-19 will be performed in select subgroups specified below based on the PP Set by treatment cohort. The efficacy endpoint of COVID-19 will be analysed by each of the subgroups using the same methods described for the primary analysis for each category of the following classification variables:

- Age groups: ≥ 18 and < 65 years, ≥ 65 years
- Stratification factor at randomization: ≥ 18 and < 65 years and not at risk, ≥ 18 and < 65 years at risk, ≥ 65 years
- Sex (female, male)
- Race
- Ethnicity
- Race and Ethnicity: non-Hispanic white, communities of color
- Each risk factor for severe COVID-19 illness as listed in Section 6.2.1 of the protocol

5.3.1.4. Variant-specific analysis of effectiveness

New SARS-CoV-2 variants have emerged. COVID-19 cases have been and will continue to be sequenced. Summary of variant cases by variants will be provided in adjudicated COVID-19 cases. Effectiveness of vaccine for specific variants will be assessed based on incidence rates if there are sufficient number of variants cases. In this variant-specific analysis, COVID-19 due to the specific variant will be considered as cases, and COVID-19 due to other variants will be censored at the time of documentation of COVID-19. Analysis of effectiveness against variants may be performed if there are sufficient numbers of variants cases for Primary series, and for booster, respectively.

Number of variant cases, person-time, and incidence rate will be provided. Person-time will be calculated as the total time, for primary series, from dose 1 of primary series; for booster, from booster date, to the earliest of the date of COVID-19, censor, last date of

study participation, or efficacy data cutoff date, whichever was earlier. The incidence rate will be calculated as the number of participants with an event divided by the number of participants at risk and adjusted by person-time (total time at risk) in each treatment group. The 95% CI will be calculated using the exact method (Poisson distribution) and adjusted by person-time.

For primary series, relative effectiveness of recent vaccinated participants (Placebo-mRNA-1273) vs. early vaccinated participants (mRNA-1273) will be assessed using the reduction in incidence rate, calculated as 1- incidence rate ratio (IRR). The 95% CI will be provided for the reduction in IR using the exact method.

5.3.2. Analysis of Other Efficacy Endpoints

The efficacy endpoints included in this section will be evaluated for Part C using Part C PP Set, for the primary series using PP Set with appropriate analysis period.

5.3.2.1. Derivation of Other Efficacy Endpoint

Derivation of other select efficacy endpoints are included in Section 6.3.2.1 of SAP Version 2.0 with modifications as below.

5.3.2.1.1. Derivation of Secondary Efficacy Endpoint: Severe COVID-19

The definition of severe COVID-19 was described in SAP Version 2.0.
For primary series, the time to the first occurrence of severe COVID-19 will be calculated as:

Time to the 1st occurrence of severe COVID-19 = Date of documented severe COVID-19 – Date of first injection of mRNA-1273 +1

For Part C, the time to the first occurrence of severe COVID-19 will be calculated as:

Time to the 1st occurrence of severe COVID-19 = Date of documented severe COVID-19 – Date of booster +1

5.3.2.1.2. Derivation of a secondary definition of COVID-19

The secondary definition of COVID-19 was provided in SAP Version 2.0.

Secondary definition of COVID-19 is derived using the censoring rules for the efficacy endpoint of COVID-19 cases, i.e. subjects with early infection, as detected by positive RT-PCR at scheduled visits (including Day 29 and PDV), or positive bAb against SARS-CoV-2 nucleocapsid protein in blood samples at the scheduled visits (including months 1, 2, 7, 13, 25, PDV, OL Day 29, OL Day 57, and scheduled visits in BD phase), are censored at the time of early infection. If a participant had eligible symptom(s) within 14 days of positive RT-PCR at scheduled visits, the participant is considered to be a case for secondary definition of COVID-19.

5.3.2.1.3. Derivation of serologically confirmed SARS-CoV-2 infection or COVID-19 regardless of symptomatology or severity

SARS-CoV-2 infection or COVID-19 regardless of symptomatology or severity is detected by post-baseline positive RT-PCR results, or seroconversion at scheduled visits applicable to individual treatment cohorts:

- Post-baseline positive RT-PCR in the study, or
- Seroconversion based on bAb specific to SARS-CoV-2 nucleocapsid at scheduled visits, defined by a negative bAb N-protein (*Roche* Elecsys SARS-CoV-2 assay) at respective reference(baseline) and a positive test at post-baseline.

5.3.2.1.4. Derivation of asymptomatic SARS-CoV-2 infection

Asymptomatic SARS-CoV-2 infection is identified by absence of symptoms and infections as detected by seroconversion or positive RT-PCR at scheduled visits applicable to individual treatment cohorts for Part B, Part C, and analysis of primary series with corresponding analysis periods:

- Absent of symptoms (no COVID-19 symptom for either efficacy endpoint of COVID-19, or secondary definition of COVID-19),
- AND at least one from below:

- Post-baseline positive RT-PCR at scheduled visits, or
- Seroconversion (bAb specific to SARS-CoV-2 nucleocapsid) at scheduled visits (PDV, OL Day 29, and OL Day 57 in Part B; BD Day 1, BD Day 29, and BD Day 181 in Part C), when blood samples for immunogenicity are collected.

5.3.2.2.Analysis of Other Efficacy Endpoints

Similar analysis methods as for the endpoint of COVID-19 cases (Section 5.3.1) will be applied to the following efficacy endpoints for analysis of primary series based on the PP set, and for Part C based on the Part C PP Set, unless otherwise specified. Start date to define cases will be similar with that for the endpoint of COVID-19 cases.

- Severe COVID-19
- SARS-CoV-2 infection or COVID-19 regardless of symptomatology or severity
- Asymptomatic SARS-CoV-2 Infection
- COVID-19 using a secondary definition of symptoms
- Death caused by COVID-19

5.4. Safety Analysis

Safety will be assessed by clinical review of all relevant parameters including unsolicited AEs, SAEs, MAAEs, AEs leading to withdrawal from study vaccine and/or study participation, vital signs, and physical examination findings.

Safety analysis of Part B will be based on the Safety Set. Part C safety analyses will be based on the Part C Safety Set. Safety analysis of mRNA-1273 primary series will be based on the Primary Series Safety Set. Analysis period is defined in Section 5.1. All safety analyses will be provided by treatment cohort, unless otherwise specified.

5.4.1. Unsolicited Treatment-emergent Adverse Events

A treatment-emergent AE (TEAE) is defined as any event occurring during the study not present before exposure to the IP or any event already present that worsens after exposure

to study vaccine. Worsening of a pre-existing condition after vaccination will be reported as a new AE.

Adverse events will also be evaluated by the investigator for the coexistence of MAAE which is defined as an AE that leads to an unscheduled visit to a healthcare practitioner.

Unsolicited AEs will be coded by PT and SOC using MedDRA and summarized by vaccination group.

All summary tables (except for the overall summary of AEs) for unsolicited AEs will be presented by SOC and PT or by PT only for TEAEs with counts of subjects included. SOC will be displayed in internationally agreed order.

When summarizing the number and percentage of subjects with an event, subjects with multiple occurrences of the same AE or a continuing AE will be counted once. Only the maximum severity level will be presented in the severity summaries, and the strongest relationship level will be presented in the relationship summaries. Percentages will be based upon the number of subjects in the Safety Set within each treatment cohort.

Unsolicited TEAEs will be summarized for Part B, Part C, and Primary Series using analysis periods as defined in Section 5.1.

5.4.1.1. Overview of Unsolicited TEAEs

An overall summary of unsolicited TEAEs including the number and percentage of subjects by treatment cohort who experience the following will be presented:

- Any unsolicited TEAEs
- Any serious AEs
- Any unsolicited AEs that are medically-attended
- Any unsolicited TEAEs leading to discontinuation from participation in the study
- Any unsolicited TEAEs that are fatal
- Any unsolicited AEs that are Grade 3 or higher

- Any non-serious TEAE
- Any non-serious TEAEs that are Grade 3 or higher

The table will also include number and percentage of subjects with unsolicited TEAEs that are treatment-related in each of the above categories.

In addition, separate listings containing individual subject adverse event data may be provided.

5.4.1.2. TEAEs by System Organ Class and Preferred Term

The following summary tables of TEAEs will be provided by SOC and PT using frequency counts and percentages (i.e. number and percentage of subjects with an event):

- All unsolicited TEAEs
- All unsolicited TEAEs that are treatment-related
- All serious AEs
- All serious AEs that are treatment-related
- All unsolicited TEAEs leading to discontinuation from study vaccine (mRNA-1273 primary series only)
- All unsolicited TEAEs leading to discontinuation from participation in the study
- All unsolicited severe TEAEs
- All unsolicited severe TEAEs that are treatment-related
- All unsolicited AEs that are medically-attended

Summary tables of all unsolicited TEAEs, Serious AEs, treatment-related SAEs, MAAEs, and TEAE leading to discontinuation from participation in the study will also be provided by SOC and PT.

In addition, summary tables of select TEAEs of clinical interests identified by SMQ will be provided by PT.

Summary of AE of special interest per investigator's assessment will also be provided by SOC and PT.

5.4.1.3.Subgroup Analysis of TEAEs

An overview of TEAE, TEAE summaries presented by SOC and PT will be provided for the following subgroups:

- Age group (<65, and ≥ 65 years)
- Sex (male, female)
- Pre-booster SARS-CoV-2 status
- Race
- Ethnicity

These summaries may be provided for subgroups of select baseline characteristics.

5.4.2. Other Safety Endpoints Pregnancies

For Vital sign measurements, including systolic and diastolic blood pressures, heart rate, respiratory rate, and body temperature, the abnormalities meeting the toxicity grading criteria will be summarized .

A listing of pregnancies including outcome will be provided.

5.5. Immunogenicity Analysis

The secondary immunogenicity objective (Part A), to evaluate the immunogenicity of 2 doses of mRNA-1273 given 28 days apart, have been evaluated and summarized in the Part A CSR (dated 03 Aug 2021) using the Per-protocol stratified random subcohort (PPRSI). mRNA-1273 was highly immunogenic as measured by both bAb and nAb in both SARS-CoV-2 baseline-negative and baseline-positive individuals, as indicated by increased bAb and nAb levels 1 month after first injection (Day 29) and 1 month after second injection (Day 57).

This SAP includes analyses of immunogenicity data to evaluate the immunogenicity of mRNA-1273 primary series and booster.

In principle, summary (descriptive statistics) will be provided for available immunogenicity data by timepoints in relative of primary series and booster dose respectively. Comparison or analysis to assess difference between groups (Section 5.5.3) will only be performed using the same assay from the same (central) testing lab.

For immune response to a booster, Part C PPIS (Part C Per-Protocol Immunogenicity Subset), defined in Section 4.3, will be used for summaries of immune response, and data will be presented by pre-booster SARS-CoV-2 status. Part C PPIS-Neg, Part C PPIS – Pre-booster SARS-CoV-2 Negative defined in Section 4.4, will be used as the primary analysis population for comparison of immune response, either as paired-comparison when a subject serves as his/her own control; or between-group comparison compared to post-primary series in Part A using mRNA-1273 subjects in PPRSI with negative baseline SARS-CoV-2 as historical control.

5.5.1. Immunogenicity Data

The following immunogenicity assessments (assays) may be used in this study:

- The MSD-ECL Multiplex Assay (MSD-ECL = meso scale discovery – electrochemiluminescence assay) measures binding antibody to antigens corresponding to: Spike; the Receptor Binding Domain (RBD) of the Spike protein; and nucleocapsid protein, which is not contained in mRNA-1273.
- Pseudovirus nAb assay (PsVNA) for measuring neutralizing antibodies against SARS-CoV-2 Spike-pseudotyped viruses. Please see Appendix H for description of the pseudovirus nAb assay.

With the emergence of variants, neutralizing and/or binding antibodies against specific variants are of interest, and such data will be summarized and analyzed in the same way for that against the prototype strain.

5.5.2. Summary of Antibody-Mediated Immunogenicity Endpoints

Quantitative binding and neutralizing antibodies will be summarized by treatment group and by corresponding "baseline" SARS-CoV-2 status (e.g. baseline/pre-Day1, or pre-booster) if applicable:

- Geometric mean titer (GMT) or value with corresponding 95% CI will be provided at each time point. The 95% CIs will be calculated based on the t-distribution of the log-transformed values then back transformed to the original scale for presentation. The detail of calculation is provided in Appendix G.
- Geometric mean fold rise (GMFR) of titer with corresponding 95% CI will be provided at each post-baseline timepoint over corresponding baseline or reference at Day 1. GMFR may be calculated based on two types of baseline:

    1) pre-Dose 1 baseline prior to the first dose of mRNA-1273
    2) pre-booster baseline (where applicable)

  The 95% CIs will be calculated based on the t-distribution of the log-transformed values then back transformed to the original scale for presentation. GMFR calculated from the pre-vaccination (pre-Dose 1) will be considered as the primary approach.
- Seroresponse rate (SRR), defined as proportion of subjects achieving seroresponse, will be provided with 2-sided 95% Clopper-Pearson CIs.

5.5.2.1.1. Seroresponse Rate (SRR) of the Primary Series and Booster

For each of the antibodies of interest, seroresponse rate (SRR) is defined as the proportion of participants achieving seroresponse.

Seroresponse of the booster is assessed based on two types of baselines (Pre-vaccination /Pre-Dose 1 of the primary series; Pre-booster) as described below. Seroresponse of a booster based on pre-vaccination (pre-Dose 1 of the primary series) value will be considered the primary definition of seroresponse of the booster:

- a 4-fold rise definition compared to Pre-vaccination (Pre-Dose 1 of the primary series) baseline, defined as
    - ≥ 4-foldrise for those with Pre-vaccination /Pre-Dose 1 value ≥ LLOQ
    - ≥ 4*LLOQ for those with Pre-vaccination /Pre-Dose 1 value < LLOQ;
    - If there is no pre-Dose 1 antibody titer information and there was evidence that the participant did not have SARS-CoV-2 infection prior to vaccination, i.e. negative SARS-CoV-2 status at the pre-vaccination/pre-dose 1 of primary series, these participants' pre-vaccination/pre-dose 1 antibody titer may be assumed to be <LLOQ at pre-dose 1 of primary series.
- a 4-fold rise definition compared to Pre-booster value, defined as
    - ≥ 4-foldrise for those with Pre-booster value ≥ LLOQ
    - ≥ 4*LLOQ for those with Pre-booster value < LLOQ;

If an assay-specific definition proposed by the Sponsor is available, assay-specific SRR definitions based on the two types of baselines may be provided. Please refer to Appendix I on assay-specific definitions used for Part A analysis of immune response.

In addition to GMT, GMFR, SRR, the number and percentage of subjects with fold-rise ≥ 2, and fold-rise ≥ 4 from baseline at each post injection time points will be tabulated with 2-sided 95% Clopper Pearson CI. For select nAb and bAb specific to S-protein, number and percentage of subjects with ≥ 4*LLOQ at select timepoints with 2-sided 95% Clopper Pearson CI may also be provided.

For select nAb and bAb specific to S-protein, boxplots of antibodies, and reverse cumulative distribution function curve (RCDF) at timepoint of interest will be provided.

5.5.3. Analysis of Antibody-Mediated Immunogenicity Endpoints

5.5.3.1. Durability of immune response to the Primary Series: Mixed Model for Repeated Measures (MMRM)

At the completion of P301, if immunogenicity data of the primary series beyond Day 57 are deemed to be sufficient, an exploratory analysis using a mixed model for repeated measures (MMRM) may be carried out to o characterize the immune response of the primary series of mRNA-1273 in SARS-CoV-2-specific nAb and S protein-specific bAb. The model may be carried out using SAS PROC MIXED. If performed, this analysis will be based on the participants on mRNA-1273 in the Part C PPIS-Neg (PPIS with negative pre-booster SARS-CoV-2 status).

For each select SARS-CoV-2-specific nAb and S protein-specific bAb of interest, the model will include all available log-transformed antibody titers at each post-Dose 1 of mRNA-1273 timepoints as the dependent variable. No treatment group variable in the model as all subjects in the model have received primary series of mRNA-1273. Visits will be re-parameterized with respect to target days post-Dose of the primary series of mRNA-1273.The model will include Visit (as a class variable) as fixed effects, with adjustment for stratification factor used for randomization, and subject as a random effect. The model may also adjust for other characteristics such as Sex. An unstructured covariance structure will be used to model the within-subject errors. A Kenward-Roger approximation will be used for the denominator degrees of freedom. If there is a convergence issue due to the unstructured covariance matrix, a compound symmetry covariance structure will be used to model the within-subject errors. No imputation of missing data will be done.

GMT at each post-Dose 1 of primary series timepoint will be estimated by the geometric least squares mean (GLSM) and corresponding 2-sided 95% CI estimated from the model. Ratio of GMT (change) between two timepoints may also be estimated by the ratio of GLSM. The corresponding 95% CI results in log-transformed scale estimated from the model will be back-transformed to obtain these estimates in the original scale.

5.5.3.2. Immunobridging of response of a Booster as compared to Primary Series

Immunobridging analyses of a booster as compared to the primary series will be performed using the neutralizing antibody titers (as measured by PsVNA) 28 days following the booster dose (BD-D29) to the corresponding titers 28 days following the second dose of the primary series (Day 57 of the blinded phase for the mRNA-1273 cohort). Immunobridging analyses may also be performed using binding antibody specific to S-protein by MSD.

The following endpoints will be used for this analysis:

- Geometric mean titer (GMT)
- Seroresponse Rate (SRR)

For GMT:

In the situation that subjects in PPIS-Neg Set have the same specific nAb (e.g. PsVNA) at baseline/pre-Dose 1, 28 days after Dose 2 of primary series, pre-booster, 28 days after booster and other timepoints, GMT ratios will be calculated by back transforming the mean of within-subject paired differences of antibody titers on the logarithmic scale between post-booster Day 29 (of the booster phase) and Day 57 of primary series (Day 57 of the blinded phase for the mRNA-1273 cohort). CIs for the GMT ratio will be based on t-distribution of the log-transformed values then back transformed to the original scale for presentation.

Another approach is to compare the post-booster antibody titers in Part C with the post-primary series titers in baseline-SARS-CoV-2 negative mRNA-1273 participants. An analysis of covariance (ANCOVA) model will be performed using the Part C PPIS-Neg Set with baseline-SARS-CoV-2 negative mRNA-1273 participants in PPRSI (considering as historical control). In the model, antibody titers at 28 days post-booster (BD-Day 29) and post-Dose 2 of primary series will be a dependent variable, and a group variable (Booster and mRNA-1273 primary series) will be the fixed effect, adjusting for age groups (< 65, ≥ 65 years). The GMT will be estimated by the geometric least square mean (GLSM) from the model and its corresponding 95% CI will be provided for each group. The GMR (ratio of GMTs) for Booster with respect to mRNA-1273 primary series will be estimated by the ratio of GLSM from the model and the corresponding 95% CI will be provided. The 95% CI for

GMR will be used to assess the between group difference in immune response 28 days post-booster as compared to 28 days post-Dose 2 of the primary series.

For Seroresponse:

Seroresponse of a booster will be compared with that of the primary series based on the of the SRR difference (booster – the primary series) and seroresponse based on change (fold rise) from pre-vaccination (pre-dose 1 of the primary series) would be considered the primary approach.

Using Part C PPIS-Neg, SRR at Day 29 of the booster phase will be compared with the SRR at Day 57 of primary series (Day 57 of the blinded phase for the mRNA-1273 cohort or OL-D57 for the Placebo-mRNA-1273 cohort). The difference in SRR and its corresponding 95% CI based on adjusted Wald method (Bonett et al 2012) will be provided. If feasible, the adjusted Wald method is adopted owing to the fact that the seroresponses at different time points can be paired within each participant.

If within-subject paired-comparison is not feasible, the Miettinen-Nurminen's method will be used to calculate the 95% CI for SRR difference (booster – primary series). Baseline-SARS-CoV-2 negative mRNA-1273 participants in PPRSI, as historical control, will be compared with PPIS-Neg regarding SRR difference (booster – primary series).

The 95% confidence interval (CI) of the ratio of GMT ratio (GMR) and 95% CI of seroresponse rate (SRR) difference will be used to assess the immune response of a booster (50 µg mRNA-1273) as compared to mRNA-1273 100 µg Primary Series (PS). All tests will be performed at nominal alpha level of 0.05 (2-sided). The table below lists possible scenarios of GMR and SRR difference results and corresponding conclusions.

Endpoints	Scenario		Conclusion
GMT Ratio	1	Lower bound of 95% CI of GMR ($GMT_{booster}$/ $GMT_{mRNA\text{-}1273\ PS}$) < 0.67	Non-inferiority of a booster as compared to mRNA-1273 100 µg primary series (PS) based on GMR is not demonstrated

	2	$0.67 \leq$ Lower bound of 95% CI of GMR ($GMT_{booster}$/ $GMT_{mRNA\text{-}1273\ PS}$) ≤ 1	Non-inferior of a booster as compared to mRNA-1273 PS based on GMR is demonstrated but superiority is not demonstrated
	3	Lower bound of 95% CI of GMR ($GMT_{booster}$/ $GMT_{mRNA\text{-}1273\ PS}$)> 1	Superiority of a booster as compared to mRNA-1273 PS based on GMR is demonstrated
SRR Difference	1	Lower bound of 95% CI of SRR difference ($SRR_{booster}$ - $SRR_{mRNA\text{-}1273\ PS}$) $\leq$ -10 %	Non-inferiority of a booster as compared to mRNA-1273 PS based on SRR difference is not demonstrated
	2	-10% < Lower bound of 95% CI of SRR difference ($SRR_{booster}$ - $SRR_{mRNA\text{-}1273\ PS}$) ≤ 0	Non-inferiority of a booster as compared to mRNA-1273 PS based on SRR difference is demonstrated but superiority is not demonstrated
	3	Lower bound of 95% CI of SRR difference ($SRR_{booster}$ - $SRR_{mRNA\text{-}1273\ PS}$) > 0	Superiority of a booster as compared to mRNA-1273 PS based on SRR difference is demonstrated

5.6. Exploratory Analysis of Effectiveness

Exploratory analyses that were not described in SAP Part A will be included in this section. This section also documents modifications or additions to the analysis plan which are resulted from information that was not available at the time of SAP Part A finalization.

5.6.1. Durability of Vaccine Efficacy of Primary Series (including data from both blinded and OL- Phase before boost Data)

The durability of vaccine efficacy may be evaluated to see if the effect of the vaccine is waning over time. These exploratory analyses will include data from both blinded phase (Part A) and OL-phase (Part B) before booster.

5.6.1.1. Methods Proposed by Tsiatis and Davidian (2021a/b)

Tsiatis and Davidian (2021a/b) proposed a framework to conceptualize counting process for infection by making transparent assumptions about biological, behavioral and other phenomena. The method provides a mechanism based on inverse probability weighting (IPW) to account for possible confounding. The confounding is introduced in the process of unblinding and post-treatment choice of cross-over to vaccine for those who are originally assigned to placebo.

In this approach, the VE is modeled via the following infection rate ratio

$$R^b(\tau;\theta) = \exp\{\zeta(\tau)\}I(\tau < \ell) + \exp\{\theta_0 + g(\tau - \ell;\theta_1)\}I(\tau \geq \ell) \quad (1)$$

and VE=1-$R^b(\tau;\theta)$

Here, τ is the time since vaccination, and ℓ is the lag time from vaccination to full immunity achieved, which is approximately 6 weeks (2 doses given 28 days apart, and 14 days for vaccine effect to ramp up) The relevant VE is therefore estimated by two parts:

- The first part is via θ_0, which models the VE at the time when full immunity is achieved ($\tau = \ell$). This is mainly estimated via the data from double-blind period where the infection data from both vaccine arm and placebo arm is available.
- The second part is via θ_1, which models the waning effect as a function of time since vaccination. $\theta_1 = 0$ implies that VE doesn't change with time since vaccination, and $\theta_1 > 0$ implies that VE decreases with increased time since vaccination, meaning waning. And the value of θ_1 indicates the magnitude of waning effect. We will explore different optional form of the g function (e.g. linear, or piece-wise constant) to evaluate how the effect change over time.
    - The data from unblinded phase will contributing in estimating the waning effect θ_1, by evaluating how infection rates changes under vaccine arm after different times since vaccination τ_1 and τ_2. The infection rates can be estimated based on the infection status data at time t from vaccinated individuals who received vaccine at times $t - \tau_1$ and $t - \tau_2$, respectively.

This will reflect information about waning of the vaccine itself while controlling for the possibly different background infection rate at time t.

Data will be censored at the time of booster.

A sensitivity analysis may be performed including the stratification factor (≥ 18 and < 65 years and not at risk, ≥ 18 and < 65 years and at risk, or ≥ 65 years) as covariates for building models for entry, unblinding and censoring mechanism to evaluate the impact of confounding.

5.6.1.2. Methods Proposed by Lin et al (2021, 2022)

Lin et al (2021, 2022) proposed a method to evaluate long-term COVID-19 vaccine efficacy using Cox regression model with a time-varying vaccine effect

$$\lambda(t|S,X) = \lambda_0(t)e^{(\beta X + I(t>S)\eta(t-S))} \quad (2)$$

Where $\eta(t - S)$ is a piece-wise linear function (e.g. every week).

Here, S denote the time when first dose of vaccination takes place and T denote the time when the endpoint events occurs. Both times are measured in days from the start of the clinical trial. We measure each participant's time to disease from the start of the trial rather than from their time of enrollment because the risk of disease depends on community transmission, which varies over the calendar time. The two timescales are different under staggered enrollment. X denote baseline stratification factor (≥ 18 and < 65 years and not at risk, ≥ 18 and < 65 years and at risk, or ≥ 65 years).

Based on the cox regression, vaccine effect is modeled as a function (η) of time since vaccination (t-S) while controlling for the possibly different background infection rate at time t. We may evaluate both the cumulative vaccine effect over the time and instantaneous vaccine effect over time.

5.6.2. The effectiveness of booster

It is important to note that since the variant circulating in the community is changing over time, so the time effect could be partially confounded with boost effect if the infection rate before and post boost is compared without adjustment. To handle this issue, the cox model with time-varying covariates allows the comparison of boosted vs un-boosted at any given time *t* time, and this adjustment estimate the boost effect while controlling for the possibly different background infection ratio at time *t*. In all analysis below, we will analyze the infection caused by any variant. Variant-specific analysis for booster may be explored using competing risk framework (i.e. censoring the other variant which is not of the primary interest) in the future. These analyses may be applied to assess effectiveness of booster on COVID-19, severe COVID-19 and death due to COVID-19.

Specifically, two approaches may be used for exploratory analysis to evaluate the effectiveness of booster.

5.6.2.1 Methods Proposed by Lin et al (2021, 2022)

The effectiveness of booster will be analyzed by a modification of Cox regression model with a time-varying vaccine effect proposed by Lin et al (2021, 2022). The hazard function is modeled by:

$$\lambda(t|S,X)) = \lambda_0(t)e^{(\beta X + I(t>S)\eta(t-S))} \quad (3)$$

Where S is the calendar time of boost, and t is the calendar time of the infection. So η(t-S) is a piece-wise linear function (e.g. every week) which models the booster effect over time as a function of time since boost. It will also provide information on when the booster achieves its full effect.

The data will only include the subject who had completed the full primary series (two doses of mRNA-1273 vaccine, PP Set). And the time will start at the time when the first subject received the 2nd dose of mRNA-1273 (time zero). Each of the subject will enter

the risk set at the time they took the 2nd dose. To control the waning effect of the prime, time of 2nd dose from time zero will be included as a covariate in X. X also include the baseline stratification factor for randomization.

5.6.2.2 Methods Proposed by Fintzi and Follmann (2021)

Another exploratory analysis to One exploratory analysis to evaluate the effectiveness of booster is using cox proportional hazard model with booster as a time-varying covariate (Fintzi and Follmann 2021, also known as step-wedge approach) . Such model compares the infection rate for boosted vs unboosted at a given time t. Time since second dose of vaccination will be included as a continuous covariate. This can be viewed as an analysis of an individual level step-wedge observational study (Brown and Lilford 2006). It is similar to the analyses used to assess booster efficacy of the BNT162b2 vaccine in Israel [Bar-On et al 2021]. Technically, we will use the following model with t as calendar time:

$$h(t) = h0(t) \exp(b1*ZB(t) + b2*TPS + b3*ZB(t)*TPS + b4*X1+b5*X2) \qquad (4)$$

Here, ZB(t) is the time-varying covariate which takes value of 0 before boost, and takes the value of 1, 14 or more days after boost. Since the booster effect may not be fully achieved until 14-days after the injection, subjects and their events (if any) during the 14-day period will be removed from the risk set between the boost dose date and 14 days later. X1 and X2 are covariates coded for stratification factor (≥ 18 and < 65 years and not at risk, ≥ 18 and < 65 years and at risk, or ≥ 65 years).

If we define TP as the time since second dose as of the earliest date of a participant receiving the booster (first date of fist booster dose), TPS is the standardized time since prime defined as TP-min(TP). Min(TP) is the time since prime for the subject who had the shortest time since prime. This standardization (via subtraction) is to facilitate the interpretation of the parameter estimation.

In this case, boost efficacy by standardized time since prime can be estimated as

$$BE(tps) = 1 - \exp(b1 + b3\ tps)$$

where BE at tps=0 is the boost efficacy among those who had the shortest time since prime.

The analysis will be confined within the period between the time when the first subject with booster administrated and the last subject with booster administrated. However, it should be noted that since there is no randomization between boosted and unboosted at a given time t, therefore, boost effect will still be confounded with the potential population difference between those who choose to have booster early vs late. So the results should be interpreted with caution. Sensitivity analysis may be considered to adjust for this confounding effect.

A sensitivity analysis about the impact of time since prime will use a binary variable instead of a continuous covariate. This binary variable will indicate if the subject received vaccine as original randomized treatment (early cohort), or as the cross-over treatment after unblinding when vaccine is approved (late cohort). Another form of sensitivity analysis will include quadratic function of TPS.

It is important to note that since the booster effect reflect the average effect 14 or more days after booster. If the full effect is not achieved by day 14, then this average effect will be smaller than the full effect. This result will be interpreted together with the results from model (3).

Additional exploratory analysis may be performed to assess the impact of covariates in addition to the stratification factor for randomization (age and risk group) that could potentially associated with booster time and COVID risk, such as time since prime , sex, race/ethnicity, or occupational risk.

6 Changes from Planned Analyses in Protocol

Not applicable.

7 References

Andersen, P.K., Borgan, O., Gill, R.D., Keiding, N. Statistical Models Based on Counting Processes. Springer. 1993.

Estimands and Sensitivity Analysis in Clinical Trials. The Guideline on Statistical Principles for Clinical Trials. International Council for Harmonisation (ICH) E9(R1). November 2019.

Fine P.F., Gray R.J. A Proportional Hazards Model for the Subdistribution of a Competing Risk. Journal of the American Statistical Association. December 1999; Vol. 94, No. 446, 496-509.

Gilbert, P.B., Wei, L. J., Kosorok, M. R., Clemens, J. D. Simultaneous Inferences on the Contrast of Two Hazard Functions with Censored Observations. Biometrics. 2002 Dec; 58(4): 773-80.

Guidance for Industry: Toxicity Grading Scale for Healthy Adult and Adolescent Volunteers Enrolled in Preventive Vaccine Clinical Trials. U. S. Department of Health and Human Services (DHHS 2020). Food and Drug Administration. Center for Biologics Evaluation and Research. September 2007.

Miettinen OS, Nurminen M. Comparative analysis of two rates. Statistics in Medicine 1985; 4:213-226.

Prentice, R. A case-cohort design for epidemiologic cohort studies and disease prevention trials. Biometrika. 1986, 73, 1-11.

Zeng D. Estimating marginal survival function by adjusting for dependent censoring using many covariates. The Annals of Statistics. 2004, 32(4), 1533-1555.

P. B. Gilbert et al. Immune Correlates Analysis of the mRNA-1273 COVID-19 Vaccine Efficacy Trial. Science10.1126/science.abm3425 (2021)

R Pajon et al. Initial Analysis of Viral Dynamics and Circulating Viral Variants During the mRNA-1273 Phase 3 COVE Trial, Nature Medicine (Nov 2021)

Tsiatis, AA, Davidian, M. Estimating vaccine efficacy over time after a randomized study is unblinded. Biometrics. 2021a; 1– 14. https://doi.org/10.1111/biom.13509

Tsiatis, AA, Davidian, M. Rejoinder: Estimating vaccine efficacy over time after a randomized study is
unblinded. Biometrics. 2021b; 1– 4. https://doi.org/10.1111/biom.13539

Lin DY, Zeng D, Gilbert PB. Evaluating the Long-term Efficacy of Coronavirus Disease 2019 (COVID-19) Vaccines. Clin Infect Dis. 2021 Nov 16;73(10):1927-1939. doi: 10.1093/cid/ciab226. PMID: 33693529; PMCID: PMC7989522.

Brown, Celia A, and Richard J Lilford. "The stepped wedge trial design: a systematic review." BMC medical research methodology vol. 6 54. 8 Nov. 2006, doi:10.1186/1471-2288-6-54.

Bar-On YM, Goldberg Y, Mandel M, Bodenheimer O, Freedman L, Kalkstein N, Mizrahi B, Alroy-Preis S, Ash N, Milo R, Huppert A. Protection of BNT162b2 Vaccine Booster against Covid-19 in Israel. N Engl J Med. 2021 Oct 7;385(15):1393-1400. doi: 10.1056/NEJMoa2114255. Epub 2021 Sep 15. PMID: 34525275; PMCID: PMC8461568.

Fintzi, J, Follmann, D. Assessing vaccine durability in randomized trials following placebo crossover. Statistics in Medicine. 2021; 40: 5983– 6007. https://doi.org/10.1002/sim.9001

Dan-Yu Lin, Yu Gu, Bradford Wheeler, Hayley Young, Shannon Holloway, Shadia-Khan Sunny, Zack Moore, and Donglin Zeng. Effectiveness of Covid-19 Vaccines over a 9-Month Period in North Carolina. New England Journal of Medicine. January 12, 2022 DOI: 10.1056/NEJMoa2117128

8 List of Appendices

Appendix A Standards for Variable Display in TFLs

Continuous Variables: The precision for continuous variables will be based on the precision of the data itself. The mean and median will be presented to one more significant figure than the original results; the SD will be presented to two more significant figures than the original results; the minimum and maximum will be presented to the same precision as the original results.

Categorical Variables: Percentages will be presented to 1 decimal place. If the count is 0, the percentage will not be displayed. If the count equals the denominator, the percentage will be displayed as 100.

Appendix B Analysis Visit Windows

Analysis will be summarized using the following analysis visit window for post injection assessments:

Step 1: If the assessments are collected at a scheduled visit, the collected data will be mapped to the nominal scheduled visit.

Step 2: If the assessments are collected at an unscheduled visit, the collected data will be mapped using the analysis visit windows described in Table B-1 below. For subjects with confirmed COVID-19, unscheduled assessments will be preferably mapped to the visits with respect to the confirmation of COVID-19 (i.e. Illness Visit Day xx) over nominal scheduled visits (i.e. Day xx).

If a subject has multiple assessments within the same analysis visit, the following rule will be used:

- If multiple assessments occur within a given analysis visit, the assessment closest to the target study day will be used.
- If there are 2 or more assessments equal distance to the target study day, the last assessment will be used.

Table B-1 **Analysis Visit Windows**

Visit	Target Study Day	Visit Window in Study Day
**NP swab (or saliva)**		
Day 1	1 (Date of First Injection)	1, first-dose
Day 29	29 (Date of Second Injection)	
Illness Visit Day 1	X (Date of NP Swab test)	X
Illness Visit Day 3	X+2	[X+1, X+2]

Illness Visit Day 5	X+4	[X+3, X+4]
Illness Visit Day 7	X+6	[X+5, X+6]
Illness Visit Day 9	X+8	[X+7, X+10]
Illness Visit Day 14	X+13	[X+11, X+16]
Illness Visit Day 21	X+20	[X+17, X+23]
Illness Visit Day 28	X+27	[X+24, X+34]
PDV	Y	Y
BD Day 1	Z	Z
**Vital Signs**		
Day 1	1 (Date of First Injection)	1, Pre-first-dose
Day 1	1 (Date of First Injection)	1, Post-first-dose
Day 29	29 (Date of Second Injection)	[2, 43] Pre-second-dose
Day 29	29 (Date of Second Injection)	[2, 43] Post-second-dose
Day 57	57	[44, 133]
Day 209	209	[134, 301]
Day 394	394	[302, 576]
Day 759	759	[577,end of study if did not receive booster]
Illness Visit Day 1	X (Date of COVID-19 Confirmation)	X
Illness Visit Day 28	X+27	[X+2, X+34]
OL Day 1	Y (Date of First Injection in Part B)	Y, Pre-first-dose in Part B

OL Day 1	Y (Date of First Injection in Part B)	Y, Post-first-dose in Part B
OL Day 29	Y+28 (Date of Second Injection in Part B)	[Y+1, Y+42], Pre-second-dose in Part B
OL Day 29	Y+28 (Date of Second Injection in Part B)	[Y+1, Y+42], Post-second-dose in Part B
OL Day 57	Y+56	[Y+43, Y+70]
BD Day 1	Z (Date of Booster Dose in Part C)	Z, Pre-first-dose in Part C
BD Day 1	Z (Date of Booster Dose in Part C)	Z, Post-first-dose in Part C
BD Day 29	Z+28	[Z+1, Z+105]
BD Day 181	Z+180	[Z+106, end of study if received booster]
**Immunogenicity**		
Day 1	1	1, first-dose
Day 29	29 (Date of Second Injection)	[2, 43] Pre-second-dose
Day 57	57	[44, 133]
Day 209	209	[134, 301]
Day 394	394	[302, 576]
Day 759	759	[577, end of study if did not receive booster]
Illness Visit Day 1	X (Date of NP Swab test)	X
Illness Visit Day 28	X+27	[X+2, X+34]

OL Day 1	Y (Date of First Injection in Part B)	Y, first-dose in Part B
OL Day 29	Y+28 (Date of Second Injection in Part B)	[Y+1, Y+42], second-dose in Part B, for participants who will receive 1 dose in Part B
OL Day 57	Y+56	[Y+1, Y+70], for participants who will receive 2 doses in Part B
BD Day 1	Z (Date of Booster Dose in Part C)	Z, Pre-first-dose in Part C
BD Day 29	Z+28	[Z+1, Z+105]
BD Day 181	Z+180	[Z+106, end of study if received booster]

Appendix C Imputation Rules for Missing Dates of Prior/Concomitant Medications and Non-Study Vaccinations

Imputation rules for missing or partial start/stop dates of medication are defined below:

1. Missing or partial medication start date:
   - If only Day is missing, use the first day of the month, unless:
     - The medication end date is on/after the date of first injection or is missing/partial AND the start month and year of the medication coincide with the start month and year of the first injection. In this case, use the date of first injection.
   - If Day and Month are both missing, use the first day of the year, unless:
     - The medication end date is on/after the date of first injection or is missing/partial AND the start year of the medication coincide with the start year of the first injection. In this case, use the date of first injection.
   - If Day, Month, and Year are all missing, the date will not be imputed, but the medication will be treated as though it began prior to the first injection for purposes of determining if status as prior or concomitant.
2. Missing or partial medication stop date:
   a. If only Day is missing, use the earliest date of (last day of the month, study completion, discontinuation from the study, or death).
   b. If Day and Month are both missing, use the earliest date of (last day of the year, study completion, discontinuation from the study, or death).
   c. If Day, Month, and Year are all missing, the date will not be imputed, but the medication will be flagged as a continuing medication.

In summary, the prior, concomitant or post categorization of medications and non-study vaccinations is described in the table below.

Table C-1 Concomitant, and Post Categorization of Medications and Non-study Vaccinations

**Part B**			
	**Medication Stop Date**		
**Medication Start Date**	**< First Injection Date of mRNA-1273 in the open-label phase**	**≥ First Injection Date in the open-label phase and ≤ 28 Days After Last Injection in the open-label phase**	**> 28 Days After Last Injection in the open-label phase [2]**
< First injection date of mRNA-1273 in the open-label phase [1]	-	C	C, A
≥ First injection date in the open-label phase and ≤ 28 days after last injection in the open-label phase	-	C	C, A
> 28 days after last injection in the open-label phase	-	-	A

**Part C**			
**Medication Start Date**	**Medication Stop Date**		

	**< Injection Date of mRNA-1273 in the booster dose phase**	**≥ Injection Date in the booster dose phase and ≤ 28 Days After Injection in the booster dose phase**	**> 28 Days After Injection in the booster dose phase [2]**
< Injection date of mRNA-1273 in the booster dose phase [1]	-	C	C, A
≥ Injection date in the booster dose phase and ≤ 28 days after injection in the booster dose phase	-	C	C, A
> 28 days after injection in the booster dose phase	-	-	A

A: Post; C: Concomitant; P: Prior

[1] includes medications with completely missing start date

[2] includes medications with completely missing end date

Appendix D Imputation Rules for Missing Dates of AEs

Imputation rules for missing or partial start dates and stop dates of AEs are defined below:

1. Missing or partial start date:
   - If only Day is missing, use the first day of the month, unless:
     - The AE end date is on/after the date of first injection or is missing/partial AND the start month and year of the AE coincide with the start month and year of the first injection. In this case, use the date and time of first injection, even if AE time was collected.
   - If Day and Month are both missing, use the first day of the year, unless:
     - The AE end date is on/after the date of first injection or is missing/partial AND the start year of the AE coincides with the start year of the first injection. In this case, use the date and time of first injection, when time is available.
   - If Day, Month, and Year are all missing, the date will not be imputed. However, if the AE end date is prior to the date of first injection, then the AE will be considered a pre-treatment AE. Otherwise, the AE will be considered treatment-emergent.
2. Missing or partial end dates will not be imputed.

Appendix E Schedule of Events

Please refer to Table 16, 17, 18, 19, 21, 22, and 23 in Appendix 1 Schedule of Events in the protocol.

Appendix F Treatment Groups/Cohorts Based on the Protocol and Treatment Groups/Cohorts Based on Actual Treatment

Part A		Part B			Treatment Cohort based on Protocol (Efficacy and Immunogenicity)	Treatment Cohort based on Actual Treatment (Safety)
Planned Treatment	Actual Treatment	Request to Receive mRNA-1273	Planned Treatment	Actual Treatment		
mRNA-1273	mRNA-1273	No			mRNA-1273	mRNA-1273
mRNA-1273	mRNA-1273	Yes	mRNA-1273		mRNA-1273	mRNA-1273
mRNA-1273	mRNA-1273	Yes	mRNA-1273	mRNA-1273	mRNA-1273	mRNA-1273
mRNA-1273	Placebo	No			mRNA-1273	Placebo
mRNA-1273	Placebo	Yes	mRNA-1273		mRNA-1273	Placebo
mRNA-1273	Placebo	Yes	mRNA-1273	mRNA-1273	mRNA-1273	Placebo-mRNA-1273
Placebo	Placebo	No			Placebo	Placebo
Placebo	Placebo	Yes	mRNA-1273		Placebo-mRNA-1273	Placebo
Placebo	Placebo	Yes	mRNA-1273	mRNA-1273	Placebo-mRNA-1273	Placebo-mRNA-1273
Placebo	mRNA-1273	No			Placebo	mRNA-1273

Placebo	mRNA-1273	Yes	mRNA-1273		Placebo-mRNA-1273	mRNA-1273
Placebo	mRNA-1273	Yes	mRNA-1273	mRNA-1273	Placebo-mRNA-1273	mRNA-1273

Appendix F Calculation of observed GMT and GMFR

The GMT and GM levels will be calculated using the following formula:

$$10^{\left\{\frac{\sum_{i=1}^{n}\log_{10}(t_i)}{n}\right\}}$$

where $t_1, t_2, \ldots, t_n$ are n observed immunogenicity titers or levels.

The geometric mean fold rise (GMFR) measures the changes in immunogenicity titers within subjects. The GMFR will be calculated using the following formula:

$$10^{\left\{\frac{\sum_{i=1}^{n}\log_{10}\left(v_{ij}/v_{ik}\right)}{n}\right\}} = 10^{\left\{\frac{\sum_{i=1}^{n}\log_{10}(v_{ij})-\log_{10}(v_{ik})}{n}\right\}}$$

where, for n subjects, v_{ij} and v_{ik} are observed immunogenicity titers or levels for subject i at time points j and k, $j \neq k$.

Appendix H Description of Pseudovirus neutralization assay used in P301

Duke PsVNA:
A pseudovirus neutralization assay from Duke University quantifies SARS-CoV-2 neutralizing antibodies utilizing lentivirus particles expressing SARS-CoV-2 Spike protein on their surface and contains a firefly luciferase (Luc) reporter gene for quantitative measurements of infection by relative luminescence units (RLU). Infection takes place in transduced 293T cells expressing high levels of ACE2 (293T/ACE2 cells).

PPD (VAC62) Assay:
A pseudovirus neutralization assay developed at PPD is designed to assess the ability of anti-SARS CoV-2 spike neutralizing antibodies to inhibit the infection of 293T-ACE2 cells utilizing SARS-CoV2 Spike Reporter Virus Particles (RVP) which express green fluorescent protein (GFP). SARS-CoV-2 infection is enumerated by counting the number of green fluorescent cells.

Appendix I Assay-specific critical fold-rise (CFR) criteria established for select assays

If an assay-specific definition proposed by the Sponsor is available, assay-specific SRR definitions based on the two types of baselines may be provided. The assay-specific definitions are currently only available for specific assays against the original virus strain as outlined here. For PsVNA ID50 titers against the original virus strain, the assay-specific seroresponse definition is based on a 3.3-fold rise which statistically distinguishes titers based on the variability of the assay USP General Chapter <1033> Biological Assay Validation. The two assay-specific definition of SRR based on the 2 types of baselines are:

- An assay-specific definition compared to Pre-vaccination (Pre-Dose 1 of the primary series) baseline. In this definition, the critical fold-rise (CFR) criterion depends on the assay and was established to statistically distinguishes titers based on the variability of the assay. Assay-specific seroresponse is defined as ≥ LLOQ for those with values <LLOQ at pre-vaccination baseline; ≥ CFR-foldrise for those with values ≥ LLOQ at pre-vaccination baseline.
- An assay-specific definition compared to pre-booster value. The same assay-specific CFR criterion is used. Assay-specific seroresponse is defined as ≥ LLOQ for those with values <LLOQ pre-booster; ≥ CFR-foldrise for those with values ≥ LLOQ pre-booster.

Table I-1. Assay-specific critical fold-rise (CFR) criteria established for select assays

Assay Name	Develop/ Testing Lab	Category	Test Name/ Description	Definition of Seroresponse
Pseudovirus	Duke	neutralizing antibodies against	*PsVNA* ID_{50}	Pre-vaccination baseline <LLOQ: >=LLOQ

(PsVNA) against the original virus strain		SARS-CoV-2 S-protein		Pre-vaccination baseline >=LLOQ: 3.3-foldrise
			PsVNA ID_{80}	Pre-vaccination baseline <LLOQ: >=LLOQ<br>Pre-vaccination baseline >=LLOQ: 2.3-foldrise
MSD multiplex (MesoScale Discovery electrochemilum inescence multiplex assay)	VRC	binding antibody levels against SARS-CoV-2	MSD anti-Spike	Pre-vaccination baseline <LLOQ: >=LLOQ<br>Pre-vaccination baseline >=LLOQ: 1.9-foldrise
