# Supplementary material for: Long-term safety and effectiveness of mRNA-1273 vaccine in adults: COVE trial open-label and booster phases
Source: Nat Commun. 2024 Aug 29;15:7469. doi: 10.1038/s41467-024-50376-z (PMC11362294; doi:10.1038/s41467-024-50376-z)
Supplement: Supplementary file 3 — Reporting Summary [file 41467_2024_50376_MOESM3_ESM.pdf]

nature portfolio

Corresponding author(s): Lindsey R Baden

Last updated by author(s): Jun 5, 2024

Reporting Summary

Nature Portfolio wishes to improve the reproducibility of the work that we publish. This form provides structure for consistency and transparency in reporting. For further information on Nature Portfolio policies, see our Editorial Policies and the Editorial Policy Checklist.

Statistics

For all statistical analyses, confirm that the following items are present in the figure legend, table legend, main text, or Methods section.

n/a	Confirmed	
☐	☒	The exact sample size (*n*) for each experimental group/condition, given as a discrete number and unit of measurement
☐	☒	A statement on whether measurements were taken from distinct samples or whether the same sample was measured repeatedly
☐	☒	The statistical test(s) used AND whether they are one- or two-sided<br>*Only common tests should be described solely by name; describe more complex techniques in the Methods section.*
☐	☒	A description of all covariates tested
☒	☐	A description of any assumptions or corrections, such as tests of normality and adjustment for multiple comparisons
☐	☒	A full description of the statistical parameters including central tendency (e.g. means) or other basic estimates (e.g. regression coefficient) AND variation (e.g. standard deviation) or associated estimates of uncertainty (e.g. confidence intervals)
☐	☒	For null hypothesis testing, the test statistic (e.g. *F*, *t*, *r*) with confidence intervals, effect sizes, degrees of freedom and *P* value noted<br>*Give P values as exact values whenever suitable.*
☒	☐	For Bayesian analysis, information on the choice of priors and Markov chain Monte Carlo settings
☐	☒	For hierarchical and complex designs, identification of the appropriate level for tests and full reporting of outcomes
☐	☒	Estimates of effect sizes (e.g. Cohen's *d*, Pearson's *r*), indicating how they were calculated

Our web collection on statistics for biologists contains articles on many of the points above.

Software and code

Policy information about availability of computer code

Data collection	Not applicable.
Data analysis	All analyses were conducted using SAS Version 9.4 or higher.

For manuscripts utilizing custom algorithms or software that are central to the research but not yet described in published literature, software must be made available to editors and reviewers. We strongly encourage code deposition in a community repository (e.g. GitHub). See the Nature Portfolio guidelines for submitting code & software for further information.

Data

Policy information about availability of data

All manuscripts must include a data availability statement. This statement should provide the following information, where applicable:

- Accession codes, unique identifiers, or web links for publicly available datasets
- A description of any restrictions on data availability
- For clinical datasets or third party data, please ensure that the statement adheres to our policy

Data associated with this study are provided in the manuscript and/or Supplementary Appendix; the protocol and statistical analysis plan are provided as Supplementary Information. Individual-level data reported in this study involving human research participants are not publicly shared due to potentially identifying or sensitive patient information. Access to participant-level data and supporting clinical documents by qualified external researchers may be made available upon

April 2023

request and subject to review. A materials transfer and/or data access agreement with the sponsor will be required for accessing shared data. Such requests can be made to Dr. Lindsey Baden, Brigham and Women's Hospital, Boston, MA 02115, USA.

Research involving human participants, their data, or biological material

Policy information about studies with human participants or human data. See also policy information about sex, gender (identity/presentation), and sexual orientation and race, ethnicity and racism.

Reporting on sex and gender	There were 48.0% and 47.3% female participants in the mRNA-1273 and placebo-mRNA-1273 groups, respectively in the study safety set. Gender was self-reported at enrollment.
Reporting on race, ethnicity, or other socially relevant groupings	Demographic information relating to the participant's age, sex (self-reported), race and ethnicity was recorded at Screening in the eCRF.
Population characteristics	Participant demographics and baseline characteristics were generally balanced and were reported previously (Baden, L.R., et al. N Engl J Med 384, 403-416, 2021 and El Sahly, H.M., et al. N Engl J Med 385, 1774-1785, 2021) at the conclusion of the blinded part A of the study. A demographics table summarizes the characteristics (Table S1).
Recruitment	Participants were enrolled and randomized as described previously (Baden, L.R., et al. N Engl J Med 384, 403-416, 2021 and El Sahly, H.M., et al. N Engl J Med 385, 1774-1785, 2021). Participants were recruited to the sites through review of unit past participant logs for eligible individuals based on the inclusion and exclusion criteria, public advertisements using social media, and referrals from primary care physicians. As such, there are not believed to beany self-selection biases that would impact results.
Ethics oversight	The full name of the central IRB was Advarra, Inc., Columbia, MD

Note that full information on the approval of the study protocol must also be provided in the manuscript.

Field-specific reporting

Please select the one below that is the best fit for your research. If you are not sure, read the appropriate sections before making your selection.

☒ Life sciences ☐ Behavioural & social sciences ☐ Ecological, evolutionary & environmental sciences

For a reference copy of the document with all sections, see nature.com/documents/nr-reporting-summary-flat.pdf

Life sciences study design

All studies must disclose on these points even when the disclosure is negative.

Sample size	Determination of sample size was previously reported (Baden, L.R., et al. N Engl J Med 384, 403-416, 2021 and El Sahly, H.M., et al. N Engl J Med 385, 1774-1785, 2021). A description of the immunogenicity subset was previuosly described for the blinded portion of the study (El Sahly, H.M., et al. J Infect Dis 226, 1731-1742 (2022), and details of the part C immunogenicity set are provided in the supplement methods and figure S3. Details of the exploratory efficacy analysis set are also provided in the supplement.
Data exclusions	10 placebo participants who received a booster dose without receiving a primary series vaccination were included in the safety set and excluded from the per-protocol set (Figure S2). Exclusions from analysis sets are described in Figure S2. Analysis set definitions were prespecified.
Replication	In this clinical study, one sample at each time point was collected for trial participants.
Randomization	Randomization of participants in Part A was previously reported (Baden, L.R., et al. N Engl J Med 384, 403-416, 2021 and El Sahly, H.M., et al. N Engl J Med 385, 1774-1785, 2021). Participants who entered Parts B and C were not randomized.
Blinding	Study blinding in Part A was previously reported (Baden, L.R., et al. N Engl J Med 384, 403-416, 2021 and El Sahly, H.M., et al. N Engl J Med 385, 1774-1785, 2021). Parts B and C were open-label studies

Reporting for specific materials, systems and methods

We require information from authors about some types of materials, experimental systems and methods used in many studies. Here, indicate whether each material, system or method listed is relevant to your study. If you are not sure if a list item applies to your research, read the appropriate section before selecting a response.

Materials & experimental systems

n/a	Involved in the study
☐	☒ Antibodies
☒	☐ Eukaryotic cell lines
☒	☐ Palaeontology and archaeology
☒	☐ Animals and other organisms
☐	☒ Clinical data
☒	☐ Dual use research of concern
☒	☐ Plants

Methods

n/a	Involved in the study
☒	☐ ChIP-seq
☒	☐ Flow cytometry
☒	☐ MRI-based neuroimaging

Antibodies

Antibodies used

Neutralizing antibodies and binding antibodies against ancestral SARS-CoV-2 (D614G) were measured at baseline and post-baseline timepoints.

Validation

For the assessment of ancestral SARS-CoV-2 (D614G) neutralizing antibodies, post-vaccination serology samples from participants were tested using a validated reporter virus microneutralization assay (PPD, part of Thermo Fisher Scientific Vaccines Laboratory Services, Richmond, Virginia).
The MSD Multiplex assay for the detection of IgG against SARS-CoV-2 spike (S-2P protein; Wuhan-Hu-1 isolate including D614G) was validated (precision, ruggedness, relative accuracy, dilutional linearity, specificity, and selectivity), by the NIH Vaccine Research Center (Rockville, MD), Vaccine Immunology program for the detection of bAb against the S-2P protein, the receptor binding domain (RBD), and Nucleocapsid (NP).

Clinical data

Policy information about clinical studies
All manuscripts should comply with the ICMJE guidelines for publication of clinical research and a completed CONSORT checklist must be included with all submissions.

Clinical trial registration

NCT04470427

Study protocol

The protocol and SAP are provided in supplementary material.

Data collection

Study was conducted at 99 centers in the US. Data was collected from trial initiation July 27th, 2020 and to study end of January 20th, 2023. Details on analysis periods are described in Supplementary Table S29

Outcomes

The efficacy, safety, and immunogenicity outcomes of the COVE trial were previously reported for the blinded Part A of the study (Baden, L.R., et al. N Engl J Med 384, 403-416, 2021 and El Sahly, H.M., et al. N Engl J Med 385, 1774-1785, 2021). Part B of the study provides longer-term safety follow-up and effectiveness data (primary objectives) following the primary series from unblinding (or participant decision visit [PDV]) to booster dose at day 1 (BD-1). Primary objectives of Part C were safety and effectiveness of a 50-µg booster dose of mRNA-1273; immunogenicity was evaluated as secondary objective.

Safety data for Parts B and C included unsolicited AEs for 28 days after vaccination, and medically-attended (MAAEs), serious (SAEs), and AEs leading to discontinuations. In addition, safety data for Part C included AEs of special interest (AESIs) including cardiac events (myocarditis and pericarditis) and vascular events for Part C through study-end. In Part C, adverse reactions (ARs) were not solicited because the reactogenicity of the 50-µg mRNA-1273 booster dose has been found to be similar to that of the second injection of the primary series; ARs that met the criteria for a MAAE, SAE, or leading to discontinuation were recorded.

Effectiveness endpoints for the mRNA-1273 primary series and booster were assessed using active surveillance and included COVID-19 (COVE and CDC definitions), severe COVID-19 (as defined in the COVE protocol and per FDA guidance), serologically confirmed SARS-CoV-2 infection or COVID-19 regardless of symptomatology or severity, asymptomatic SARS-CoV-2 infection and death caused by COVID-19. Cases of COVID-19 and severe COVID-19 were adjudicated by an independent committee.

Immunogenicity objectives compared immune responses following the mRNA-1273 booster at day 29 (BD-29) with immunological responses following two-injections of the mRNA-1273 primary series at day 57 to infer booster effectiveness. Neutralizing antibodies (nAb) against SARS-CoV-2 were analyzed using a pseudovirus nAb assay for ancestral SARS-CoV-2 (D614G) and SARS-CoV-2 spike-bAbs using a Mesoscale Discovery (MSD) assay.

Plants

Seed stocks

Report on the source of all seed stocks or other plant material used. If applicable, state the seed stock centre and catalogue number. If plant specimens were collected from the field, describe the collection location, date and sampling procedures.

Novel plant genotypes

Describe the methods by which all novel plant genotypes were produced. This includes those generated by transgenic approaches, gene editing, chemical/radiation-based mutagenesis and hybridization. For transgenic lines, describe the transformation method, the number of independent lines analyzed and the generation upon which experiments were performed. For gene-edited lines, describe the editor used, the endogenous sequence targeted for editing, the targeting guide RNA sequence (if applicable) and how the editor was applied.

Authentication

Describe any authentication procedures for each seed stock used or novel genotype generated. Describe any experiments used to assess the effect of a mutation and, where applicable, how potential secondary effects (e.g. second site T-DNA insertions, mosiacism, off-target gene editing) were examined.
